# The Role of *ARHGAP1* in Rho GTPase Inactivation during Metastasizing of Breast Cancer Cell Line MCF-7 after Treatment with Doxorubicin

**DOI:** 10.3390/ijms241411352

**Published:** 2023-07-12

**Authors:** Imrich Géci, Peter Bober, Eva Filová, Evžen Amler, Ján Sabo

**Affiliations:** 1 Department of Medical and Clinical Biophysics, Faculty of Medicine, Pavol Jozef Šafárik University in Košice, Trieda SNP 1, 04011 Košice, Slovakia; 2 Institute of Experimental Medicine, Czech Academy of Sciences, Vídeňská 1083, 142 00 Prague, Czech Republic

**Keywords:** proteomics, mass spectrometry, cell adhesion, breast cancer, doxorubicin, metastases

## Abstract

Breast cancer is the most prevalent cancer type in women worldwide. It proliferates rapidly and can metastasize into farther tissues at any stage due to the gradual invasiveness and motility of the tumor cells. These crucial properties are the outcome of the weakened intercellular adhesion, regulated by small guanosine triphosphatases (GTPases), which hydrolyze to the guanosine diphosphate (GDP)-bound conformation. We investigated the inactivating effect of *ARHGAP1* on Rho GTPases involved signaling pathways after treatment with a high dose of doxorubicin. Label-free quantitative proteomic analysis of the proteome isolated from the MCF-7 breast cancer cell line, treated with 1 μM of doxorubicin, identified *RAC1*, *CDC42*, and *RHOA* GTPases that were inactivated by the *ARHGAP1* protein. Upregulation of the GTPases involved in the transforming growth factor-beta (TGF-beta) signaling pathway initiated epithelial–mesenchymal transitions. These findings demonstrate a key role of the *ARHGAP1* protein in the disruption of the cell adhesion and simultaneously allow for a better understanding of the molecular mechanism of the reduced cell adhesion leading to the subsequent metastasis. The conclusions of this study corroborate the hypothesis that chemotherapy with doxorubicin may increase the risk of metastases in drug-resistant breast cancer cells.

## 1. Introduction

Breast cancer is, with 12.5% of all new annual cases, at the forefront of the global cancer statistics in women. More than 90% of breast cancer deaths are caused by the local invasiveness of these tumor cells, as well as by the metastasis to distant tissues and organs [1]. The formation of metastases is based on the significant usurpation of epithelial–mesenchymal transformations (EMT) by the tumor cells in the process of morphogenesis, of which the typical manifestation is significant cellular motility and migration [2,3,4]. EMT is a dynamic process in which adhesion complexes coexist next to the newly transformed migratory cells. It is evident that, in all steps, starting from the cancer tumorigenesis, through the loss of adhesion and ending in metastasis, there are crucially involved proteins from the group of GTPases known as Rho GTPases [5].

Rho guanosine triphosphatases (Rho GTPases) belong to the Ras-type superfamily with a size of 20–25 kDa. By switching between a bound active and inactive conformation, they enter key biological signaling pathways associated with the EMT process. The affinity for both forms is very high, and GTPases rarely appear in an unbound free form [6,7]. The concentration of GTP in the cell is 10 times higher as compared to GDP; therefore, guanosine triphosphate immediately occupies the active site of GTPase, which is activated within the blink of an eye. Staying in the GTP-bound conformation is a determining factor for triggering signaling processes in the cell. The best-described members are *RAC1*, *RHOA*, and *CDC42* [8].

The negative regulator of these Rho GTPases is the protein *ARHGAP1*, the representative of more than 70 GTPase-activated proteins (GAPs), which initiate their switch to an inactive GDP-bound conformation [9], thereby regulating cellular function. A study of the invasive nature of adenocarcinomas associated with Rho GTPase activation confirmed the relationship between the loss of *ARHGAP1* and the reduction in cell invasion [10]. Next, *ARHGAP1* was identified as an essential regulator of the Rho GTPase activity in the process of germ cell embryogenesis [11]. Generally, the superfamily of GAPs is an important mediator of cancer cell death and immunity [12]. In addition, small GTPases are also regulated by more than 60 guanine nucleotide exchange factors (GEFs), which switch the GDP form back to GTP. Lastly, there are guanine nucleotide dissociation inhibitors (GDIs) which inhibit the release of GDP when specifically bound to GDP [6].

Considering the signaling pathways, GTPases are activated by the TGF-beta signaling pathway in the tumor microenvironment, followed by the destabilization of adherens junctions, leading to actin filament reorganization. This is considered a primary step in the increased cell motility and invasiveness [13]. Adherens junctions between the cells are constructed from the cadherin superfamily, including E-cadherin, as well as by members of the catenin family, including p120-catenin and α-, β-, and γ-catenin [14]. The inactivation of Rho GTPases disrupts E-cadherin localization in epithelial tissues, resulting in the suppression of cellular adherens junctions. This is considered a key step in the process of epithelial–mesenchymal transition [15,16], which can be assessed through upregulation of mesenchymal (alternatively cancer stem cell—CSC) markers such as *CD44* [2,17,18]. CSCs play a major role in tumor progression, metastasis, and drug resistance [19,20].

To better understand the relationship between the metastatic phenotype and the induction of the drug resistance, multiple EMT transcription factors were studied in the breast cancer cell line MCF-7 following a stepwise addition of the chemotherapeutic agent doxorubicin (DOX). Thus, low-dose long-term addition was shown to cause resistance of the MCF-7 cell line to DOX [21,22]. Contrastingly, short-term application of a high dose of DOX led to a considerable increase in abnormal morphology of the MCF-7 cell line, whereby a new population of so-called “spiky cells” was observed. They exhibited similar characteristics to metastatic cells and drug-resistant cells [23].

Doxorubicin, also known under the brand name Adriamycin, is a commonly used anthracycline-class chemotherapy drug for treating breast cancer via intercalation into DNA double helices, where it blocks topoisomerase II, thereby disrupting DNA synthesis. DOX also has an influence on cell proliferation by causing oxidative stress, which may result in apoptosis [24]. Additionally, DOX has an impact on other diverse human cancer types, such as leukemia, myeloma, sarcoma, and lung, ovarian, or gastric cancer. Moreover, it acts not only against cancer, but also germ cells [25].

In our present study on short-term DOX therapy, we decided to use a high dose of DOX (1 µM). We anticipated that, by elucidating the complex mechanism related to the weakening of the cell adhesion in MCF-7/DOX-1, it will be possible to gain a better understanding of the metastatic process during EMT [26]. Proteomic samples isolated from the cells before and after DOX administration were analyzed by label-free quantitative mass spectrometry (MS) and immunohistochemical staining. High-resolution MS-based quantitative proteomics was shown to be an effective tool in the analysis of new biomarkers for the prediction of breast cancer and colorectal cancer metastasis, respectively [27,28].

Here, we report a direct signaling relationship between the upregulation of GTPases *RAC1*, *CDC42*, and *RHOA* in the presence of *ARHGAP1* in MCF-7/DOX-1 cells, leading to the weakening of cell–cell adhesion. We confirmed that a high dose of doxorubicin initiates metastasis of MCF-7 breast tumor cells.

## 2. Results

### 2.1. Qualitative and Quantitative Mass Spectrometry Analysis

To gain deeper insight into the biological pathways related to changes in the breast cancer microenvironment, which modulate the invasiveness, motility, and final metastasis, the detailed protein profiles of both MCF-7 cell lines, wildtype and after treatment with 1 μM doxorubicin (MCF-7/DOX-1), were determined. As more protein entities are identified, especially those with low abundance, the understanding and function of signaling pathways became more straightforward. For this purpose, we applied multidimensional protein separation and identification technology. At first, the protein extracts were enzymatically digested with trypsin, and the observed peptides were subsequently separated by off-gel electromigration fractionation. Sequentially pooled peptidic fractions were submitted for nanoHPLC separation on a C_18_ reversed-phase trap column, followed by separation on a C_18_ analytical column connected online to an ion trap mass spectrometer. The obtained datasets were analyzed using the Mascot search engine, allowing up to two missed cleavages. The final protein identification using the software tool Scaffold 4.4.7. revealed 1803 unique proteins in total (Supplementary Materials, Table S1).

**Figure 1:**
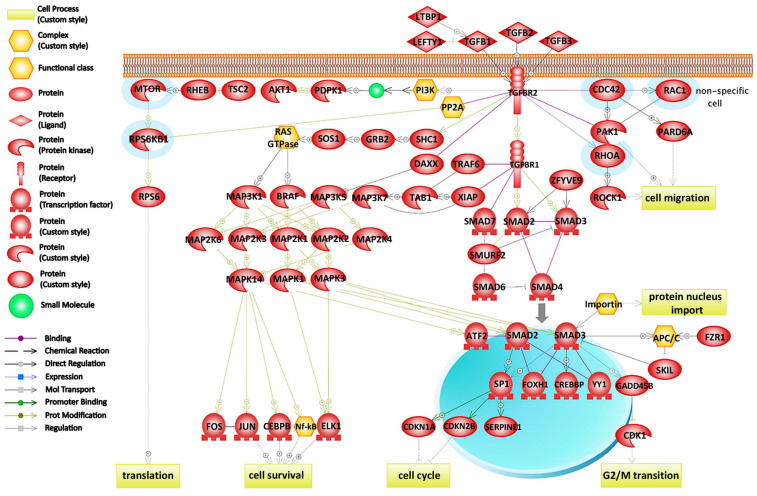
TGF-beta signaling pathway using gene set enrichment analysis algorithm (MWW test, *p* ≤ 0.05). Proteins determined by a label-free quantification (marked with a blue color rim) are involved in this pathway. Whereas *RHOA*, *RAC1*, and *CDC42* GTPases take part in cell migration, *MTOR* and *RPS6KB1* participate in translation. Colored symbols are explained in the legend.

**Figure 2:**
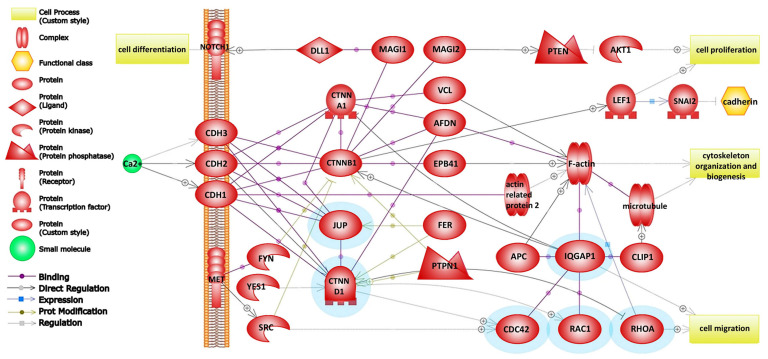
Adherens junction assembly (cadherins) pathway using gene set enrichment analysis algorithm (MWW test, *p* ≤ 0.05). Proteins marked with a blue color rim were determined in this study. Colored symbols are explained in the legend.

To explore the molecular mechanism of the loss of cellular adhesion, we looked for statistically significant changes in the group of identified proteins, employing label-free quantitative proteomic analysis, where two strategies may be applied: signal intensity or spectral counting. In our approach, we estimated the protein expression in datasets from biological triplicates by total spectral counting with the applied *t*-test (*p* ≤ 0.05), which confirmed 370 proteins (Supplementary Materials, Table S1). Using gene set enrichment analysis (GSEA; MWW test, *p* ≤ 0.05), four signal transduction pathways were identified and confirmed according to the identified significant proteins (Table 1). On the basis of the obtained significant changes, five proteins—*RAC1*, *CDC42*, *RHOA*, *MTOR*, and *RPS6KB1*—were detected (Figure 1), which were involved in the TGF-beta signaling pathway, along with six proteins—*RAC1*, *CDC42*, *RHOA*, *IQGAP1*, *JUP*, and *CTNND1* (Figure 2), which were involved in the signaling pathway of adhesive junctions (cadherins). The upregulation of proteins involved in the TGF-beta and adhesive connection signaling pathways is documented in Table 2.

Using the subnetwork enrichment analysis (SNEA) database (MWW test, *p* ≤ 0.05), three groups of proteins were confirmed in the following mechanisms: 916 proteins in chemical regulatory processes, 70 proteins in cell motility, and 53 proteins in epithelial–mesenchymal transitions (Table 3).

### 2.2. Analysis of Morphological Changes and Immunohistochemistry

**Figure 3:**
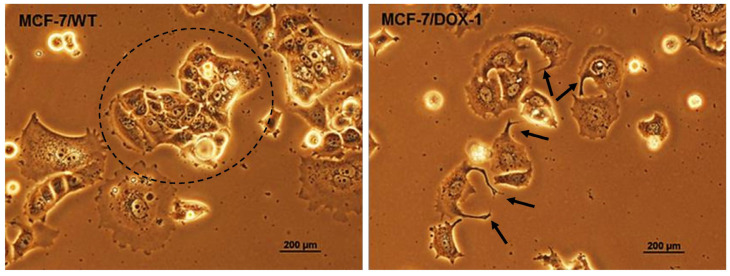
Phase-contrast microscopy images of MCF-7 breast cancer cells. Adherens junctions between MCF-7/WT cells bring them physically closer and cause them to stick to each other, as shown in the circle. These intercellular bridges, preserved by cadherin receptors, are highly dynamic cellular connections, and their disruption causes loosening of cell–cell contacts, leading to disorganization of the cell culture architecture. In the MCF-7/DOX-1 culture, after only 2 days of treatment with 1 μM of doxorubicin, cell separation and shape alteration were observed. The arrows point to spike formation as part of the changed morphology. The suppression of adherens junction integrity is the result of the action of *RHOA*, *RAC1*, and *CDC42* GTPases and their GAPs, which regulate cadherin receptors.

In the MCF-7/DOX-1 cell line, a significant increase in abnormal morphological changes was detected only 2 days after administration of the chemotherapeutic agent DOX at the concentration of 1 μM. One of these abnormal phenotypes was represented by so-called “spiky cells” (Figure 3). According to Dorland (2016), the spiky cell population appears to have properties associated with the drug-resistant cell population and exhibits metastasizing and stem cell-like properties [23].

**Figure 4:**
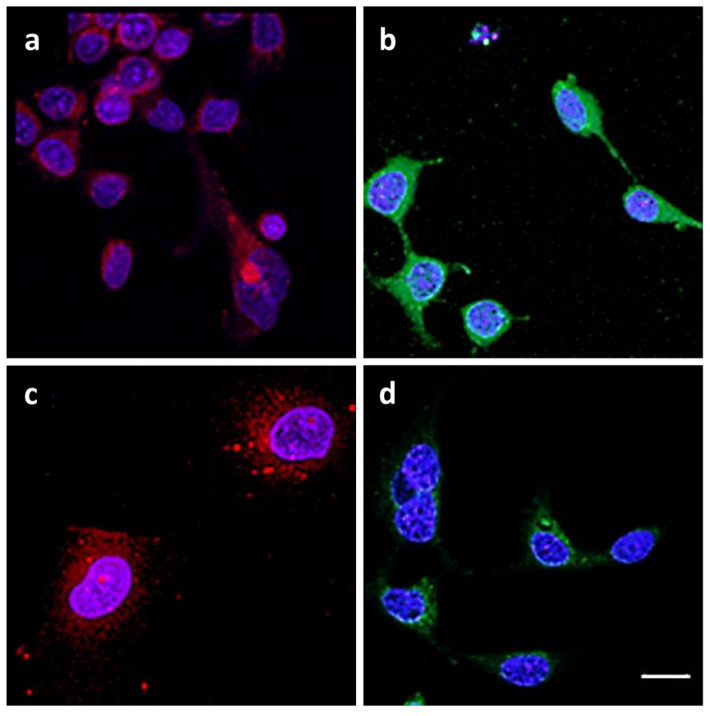
Immunohistochemical staining of MCF-7 breast cancer cell line before and after treatment with doxorubicin for *CD44* and *CD24* markers. Photomicrographs obtained with confocal microscope show MCF-7/wildtype cells (**a**,**b**) and the MCF-7/DOX-1 cell culture treated with 1 µM doxorubicin (**c**,**d**). Both cell cultures were stained with monoclonal antibody anti-CD44 conjugated with R-phycoerythrin (**a**,**c**, red) or with monoclonal antibody anti-CD24 conjugated with fluorescein isothiocyanate (**b**,**d**, green), as described in Section 4. The cell nuclei were stained using Hoechst 34580 (blue). On the basis of the staining intensity in the case of the MCF-7/DOX-1 cell line, upregulation for *CD44* and downregulation for *CD24* were assumed. The scale bar (20 µm) is equal in all photos.

Immunohistochemical staining of the glass-seeded cells with antiCD44–R-PE showed more intense and more discrete staining in case of the MCF-7/DOX-1 cell line as compared to the reference MCF-7/wildtype cell line. Contrarily, the use of CD24–FITC resulted in less intense staining in the DOX treated cell lines (Figure 4). We also attempted to perform an ImageJ quantitative comparison, which was found to be statistically not significant. As expected, after treatment with a high dose of doxorubicin, the cells decreased their proliferation and, thus, were not present in the minimal amount necessary for the quantification. On the basis of the staining intensity in the case of the MCF-7/DOX-1 cell line, we assumed upregulation for *CD44* and downregulation for *CD24*.

### 2.3. Protein–Protein Interaction Network Construction

**Figure 5:**
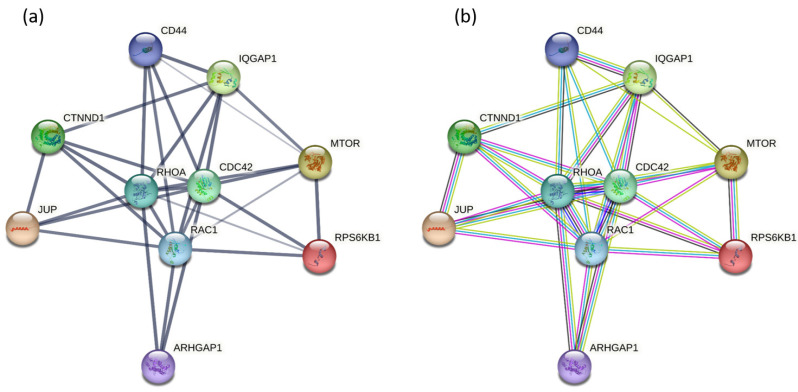
String network analysis of the protein–protein interactions among 10 upregulated proteins. The nodes correspond to proteins, whereas, in (**a**), the thickness of the edges indicates the strength of the interactions, and, in (**b**), the edges show eight differently colored lines representing protein–protein functional associations: turquoise line—presence of curated databases, magenta line—experimental evidence, green line—gene neighborhood evidence, red line—presence of gene fusions, blue line—co-occurrence evidence, lime-green line—text-mining evidence, black line—coexpression evidence, purple line—protein homology evidence.

Ten upregulated proteins from GSEA, shown in Table 2, were submitted to the STRING database to perform protein–protein analysis. As shown in Figure 5a, there was a strong interaction among *RHOA*, *RAC1*, and *CDC42*. Interestingly, no interaction between upregulated GAPs *ARHGAP1* and *IQGAP1* was found. Importantly, there was a strong individual association between all three Rho GTPases and *ARHGAP1*, as well as between GTPases and *IQGAP1*. Significantly more interactions between the protein nodes in Figure 5b indicate that it is not just a random association of random proteins, but such enrichment reflects at least a biological connection. There are two known interactions to be emphasized; the first was from curated databases, and the second one was determined experimentally. Considering the involvement of biological processes, *RHOA*, *RAC1*, and *CDC42* were present in the cell adhesion process, while all three GTPases together with *ARHGAP1* contributed to the cell migration.

## 3. Discussion

Enormous advances of biomedical engineering in molecular medicine over the last decade have allowed mass spectrometry-based cancer proteomics to bring a better understanding of biological processes such as cellular signaling events and related post-translational modifications or interactions of metabolic pathways with protein–protein networks [29]. All these endeavors in clinical proteomics lead to one goal—identifying the disease biomarkers which could serve as indicators of the biological pathological processes. Nevertheless, considering the extraordinarily high “entropy” in the sphere of clinical proteomics, which is represented by the type of the cancer, its stage of development, actual metabolic stage of the patient, associated known and unknown comorbidities, post-translational modifications, mutations, and, in many cases currently, post-COVID-19 syndromes, it might be extremely difficult to elucidate the connection between proteomic predictive identifiers and the signaling pathways in order to define the disease biomarker. Although, in our research, we deal mainly with clinical proteomics, so as to get more straightforward outcomes, we applied the MCF-7 breast cancer cell line in the present study. However, to define the precise functions of Rho GTPases via studying the TGF-beta and adherens junction pathways in such a complex process as epithelial–mesenchymal transition (EMT) associated with cancer progression and metastasis [26,27], as well as the increased number of CSCs and drug resistance, remains a challenging task. The crucial step is to determine when and where precisely do Rho GTPases change their activity, either by a particular GTPase-activating protein (GAP) or a group of proteins (GAPs) that dictate the specific subcellular localization and activity level of these molecular switchers [5]. It is known that GAPs convert *RAC1*, *CDC42*, and *RHOA* to inactive GDP-bound forms. However, to date it has not been possible to determine particular GAP family members and how they influence localized GTPase activity in the process of incapacitating adherens junctions. Therefore, we studied the mechanism related to the weakening of the cell–cell adhesion via inactivation of *RHOA*, *RAC1*, and *CDC42*, to point out the main role of *ARHGAP1* in the disruption of cell–cell adhesion during EMT.

A typical morphology for epithelial MCF-7/WT cells is that they formed close connections to their neighbors. On the contrary, the “spiky cells” developed in the MCF-7/DOX-1 culture did not form as many connections, but rather longer and spindly projections, as depicted in Figure 3. As shown before, in the case of highly metastatic human osteosarcomas, low-metastatic and high-metastatic cancer cells differed in roundness, elongation, and perimeter variability [30]. Whereas low-metastatic cancer cells displayed a more rounded mesenchymal-like morphology and much larger projected area, the high-metastatic cancer cell morphology was changed showing significant elongation and aspect ratio. Moreover, both cell lines could be discerned in terms of nuclear size and nuclear shape. According to these findings, the formed spiky breast cancer cells are unable to create the adhesion junctions, but display such genetic changes that lead to their high invasiveness and metastasis, as we found in our present study.

### 3.1. ARHGAP1 and Other Proteins Involved in EMT

According to gene enrichment analysis (GEA) of the proteins that regulate the cellular processes (MWW test, *p* ≤ 0.05) in MCF-7/DOX-1, the upregulation of several proteins related to EMT, namely, *ARHGAP1*, *FOXA1*, *TGM2*, *ARF1*, *FERMT2*, *MTA1*, and *CD44*, was obtained as a consequence of their significant expression due to genetic activity. Hepatocyte nuclear factor 3-alpha (*FOXA1*) appears to be vital in maintaining epithelial differentiation of MCF-7 cells, by providing a molecular firewall to cease the EMT progression [31]. Overexpression of protein-glutamine gamma-glutamyltransferase 2 (*TGM2*) has an influence on EMT induction, thus potentially contributing to drug resistance development and metastatic competence by means of BCCs [32]. Upregulation of ADP-ribosylation factor 1 (*ARF1*) leads to EMT and resistance to chemotherapeutic agents in triple-negative breast cancer cells [33]. Fermitin family homolog 2 (*FERMT2*) was reported to promote tumor cell adhesion, migration, and invasion in BCCs to induce EMT [34,35]. Overexpression of metastasis-associated protein (*MTA1*) determines EMT [36]. Lastly, antigen *CD44* was also upregulated during EMT [37]. On the other hand, anterior gradient protein 2 homolog (*AGR2*), which plays a role in *AGR2* loss with prediction toward weakened cell–cell junctions [38] via the EMT process [39,40], was downregulated.

Regarding the function of Rho GTPase-activating protein 1 (*ARHGAP1*) related to small GTPases, Clay and Halloran (2013) showed that *ARHGAP1* was expressed in a discrete apical region of pre-migratory neural crest cells [11]. Compared to their study that described the effect of *ARHGAP1* in EMT during embryogenesis, the main goal in our present study was to bring new knowledge in revealing the mechanism of cell–cell weakening during EMT associated with promoting metastasis.

### 3.2. Mechanism of Rho GTPase Inactivation via ARHGAP1

As mentioned above, Rho GTPases, including *RAC1*, *CDC42*, and *RHOA*, can be present in two structurally different conformations: a GTP-bound active and a GDP-bound inactive conformation [41]. In the GTP-bound active form, Rho GTPases are localized at the membranes where they interact with effector molecules, which initiate downstream responses in the EMT process. After treatment with a chemical inhibitor, such as doxorubicin (DOX) in present study, GAP proteins including *ARHGAP1* inactivate Rho GTPases *RAC1*, *CDC42*, and *RHOA* via the hydrolysis of GTP forms, switching them into the GDP-bound inactive forms located in the cytoplasm and, thus, terminating the signal delivery (see Graphical Abstract) [42]. The “switch off” of this signaling communication results in weakening of the cellular adhesion and loosening of the cell–cell junctions, which mitigates cell migration and invasion. Recently, it was found that a high dose of DOX led to significant upregulation of the substrate receptor *DCAF13* with an assumed metastasizing effect in the case of triple-negative breast cancer [43]. Suppression of *DCAF13* expression led to reduced metastasis. At the same time, a direct effect of doxorubicin treatment on EMT was detected by upregulation of beta-catenin and downregulation of fibronectin. Similarly, cancer proliferation was obtained when DOX was replaced by ubiquitin [44]. Hence, it can be concluded that DOX acted in the present study as an effector, initiating cell–cell disruption and cancer metastasis. However, further experiments are needed to verify this influence of DOX.

### 3.3. Role of Inactivated Rho GTPases in Adherens Junction Pathway during Weak Adhesion

Loss of cellular adhesion is accompanied by other cellular changes as part of the EMT. Cell adhesion molecules are divided into several groups—cadhedrins, integrins, selectins, immunoglobulin superfamily members, and others. Cadhedrins play an important role in solid tissues, where they are associated with adhesive bonds between the cells. For their proper adhesive function, calcium ions are essential. However, integrins contain a domain which mediates magnesium ligand binding.

The identified Ras GTPase-activating-like protein (*IQGAP1*), which is bound to actin filaments, is considered to have a positive function in junction formation via stabilizing the actin at the cell–cell adhesion site. It is also bound to β (γ)-catenin and prevents β (γ)-catenin binding to α-catenin, which results in decreased cell–cell adhesion. The identified *RAC1*, *CDC42*, and *IQGAP1* together regulate the cadherin-mediated cell–cell adhesion [45,46]. Being in GDP-bound inactive forms, *CDC42* and *RAC1* cannot interact with *IQGAP1*; thus, *IQGAP1* interacts with β (γ)-catenin to dissociate α-catenin from the cadherin–catenin complex. Here, the ratio of the E-cadherin–β (γ)-catenin—*IQGAP1* complex to the E-cadherin–β (γ)-catenin–α-catenin complex is high, resulting in weak adhesion and high cell–cell dissociation. Therefore, the probability that the inactivation of *RAC1* and *CDC42* is related to disruption of cell–cell adhesion significantly increases (Figure 5b) [47].

The armadillo family protein p120-catenin (p120ctn) plays a central role in the connection of adherens junctions and Rho GTPases [48]. p120ctn is considered to be an indirect regulator to assemble and disassemble adherens junctions via the Rho family of GTPases [49,50]. p120ctn binds the cadherin juxtamembrane domain (JMD), resulting in physical interaction for the stable retention of cadherin at the plasma membrane, which is necessary for strong cell–cell adhesion [51]. The increase in cytoplasmic p120ctn protein results in the formation of a p120/RHOA–GDP complex [52] and subsequent disruption of cell–cell adhesion (Figure 5a, b).

### 3.4. Role of Rho GTPases in TGF-Beta Signaling Pathway during EMT

The TGF-beta signaling pathway plays a key role in different cellular processes by switching from being a tumor suppressor (in normal or dysplastic cells) to a tumor promoter (in advanced cancers) through provoking EMT. We found that upregulated Rho GTPases *RAC1*, *CDC42*, and *RHOA* participated in the TGF-beta signaling pathway in the breast cancer MCF-7 cell line treated with DOX. TGF-beta interaction with surface receptors results in the localization of SMAD proteins, which activates the expression of E-cadherin-suppressing transcription factors. This is the crucial step toward invasion and metastasis. SMAD-dependent pathways are supposedly involved in TGF-beta tumor-suppressive functions, whereas activation of SMAD-independent pathways is connected to the loss of their tumor suppressor features [53].

Regarding the direct regulation of Rho GTPases *RAC1*, *CDC42*, and *RHOA*, transmission of SMAD-independent TGF-beta signals, leading to the reorganization of the cytoskeleton, cell motility, and invasion through their activation, is carried out [54,55]. Taking into account the cell motility, (1) *RAC1* induces focal complexes and polymerization of actin to form lamellipodia, (2) *CDC42* induces filopodia assembly, polymerization of actin and thin actin filaments projecting from the cell membrane, and (3) *RHOA* induces formation of stress fibers [56].

## 4. Materials and Methods

### 4.1. MCF-7/WT and MCF-7/DOX-1 Cultivation

The human breast adenocarcinoma MCF-7 laboratory cell line (American Type Culture Collection, Manassas, Virginia, USA) in a frozen cryotube (app. 8 × 10^5^ MCF-7 cells) was gradually heated in a water bath at 37 °C; after melting, it was immediately transferred into a 75 cm^2^ culture flask (Becton, Dickinson and Co., Franklin Lakes, NJ, USA), where 10 mL of cultivating medium was added. The cultivating medium contained Dulbecco’s modified Eagle medium (BE12-604, DMEM, 4.5 g/L glucose, Lonza-BioWhittaker, Verviers, Belgium), F-12 HAM medium (N6760, Sigma, St. Louis, MO, USA) in a 1:1 (*v*/*v*) ratio with 5% FBS (F4135, GIBCO, Paisley, UK), and 1% sodium pyruvate 100 mM (11-360-070, GIBCO, Grand Islands, NY, USA). Untreated and doxorubicin-treated MCF-7 cell lines were cultured for 2 days, followed by protein extraction and separation, before being evaluated by label-free quantitative proteomic analysis and immunohistochemistry staining (see below).

### 4.2. Cell Lysis and Extraction of Proteins

MCF-7/WT and MCF-7/DOX-1 cells were lysed with a lysis solution of 8 M urea (161-0731, Bio-Rad, USA, 100 mM Tris-HCl, pH 8, 161-0719, Bio-Rad, Hercules, CA, USA). Then, the mixture was centrifuged at 12,000× *g* and 4 °C for 15 min. Next, cold acetone (−20 °C, 1000201000, Merck, Germany) in a ratio of 1:8 (*v*/*v*) was added to the supernatant, and the mixture was vortexed and stored at −20 °C for 60 min. Afterward, the mixture was again centrifuged for 15 min at 12,000× *g* and 4 °C. The obtained pellet was dried in a vacuum concentrator (Labconco, Kansas City, MO, USA).

### 4.3. Determination of the Protein Concentration and In-Solution Digestion

The protein pellets were dissolved in 8 M urea (161-0731, Bio-Rad, USA, 100 mM Tris-HCl, pH 8, 161-0719, Bio-Rad, Hercules, CA, USA), and their concentration was determined using the Bradford method with a UV/Vis spectrophotometer (Shimadzu 3600, Kyoto, Japan). In the next step, 1.1% 0.1 M DTT solution (161-0611, Bio-Rad, USA, 100 mM Tris-HCl, pH 8, 161-0719, Bio-Rad, USA) was added, and the mixture was incubated in the light at 37 °C for 30 min. Subsequently, 0.1% 0.5 M IAA solution (163-2109, Bio-Rad, USA, 100 Mm Tris-HCl, pH 8, 161-0719, Bio-Rad, USA) was added, and the mixture was again incubated in the dark at 37 °C for 30 min. Then, cold acetone (−20 °C, 1000201000, Merck, Darmstadt, Germany) was added and left in a freezer at −20 °C for 60 min. Afterward, the mixture was centrifuged at 4000× *g* for 50 min, and the obtained pellet was dried in a vacuum concentrator (Labconco, USA) and subsequently dissolved in 8 M urea (161-0731, Bio-Rad, USA, 100 Mm Tris-HCl, pH 8, 161-0719, Bio-Rad, USA). Next, 2 mM calcium chloride (1023780500, Merck, Germany, 10 mM Tris-HCl, pH 8, 161-0719, Bio-Rad, USA) and trypsin (T6567, Merck, Germany) in a 1:100 (m/m) ratio were added, and the mixture was incubated at 37 °C for 6 h. Finally, 20% trifluoroacetic acid (TFA, 80457, Merck, Germany) was used to quench the protein digestion. The concentration of proteins was determined to be approximately 1 μg/μL. The mixtures of peptides were desalted via solid-phase extraction using C_18_ matrix cartridges (Agilent Technologies, Santa Clara, CA, USA) and eluted with a solution containing 70% acetonitrile (ACN, 270717, Merck, Germany), 30% distilled water (from own Millipore system, HPLC grade), and 0.1% formic acid (543804, Merck, Germany) (*v*/*v*/*v*); the sample was dried in a vacuum concentrator (Labconco, Kansas City, MO, USA).

### 4.4. Off-Gel Fractionation

Off-gel fractionation was conducted on 12 cm pH 3–10 IPG strips (GE17-6001-11, GE Healthcare Life Sciences, Chicago, IL, USA) using the electromigration-based fractionator (Agilent Technologies, USA). For the fractionation, 1 mg of peptides were dissolved in an off-gel solution possessing 5% glycerol (G6279, Merck, Germany) and 1% ampholyte (1631112, GE Healthcare Life Sciences, USA), and then applied to strips placed in a 12-well frame. After that, the peptide samples were run in default conditions with 24 h runtimes. Fractions were finally isolated and sequentially pooled and concentrated by vacuum centrifugation.

### 4.5. Analysis of Proteins via Nano-HPLC–ESI-MS Assembly

Biological triplicates of MCF-7/WT and MCF-7/DOX-1 were subjected to a nano-HPLC system (Ultimate 3000 RSLC Nano, Thermo Fisher Scientific, Waltham, MA, USA) in online connection with an amaZon speed ETD ion trap mass spectrometer (Bruker Daltonik, Bremen, Germany) equipped with a CaptiveSpray ion source (Bruker Daltonik, Germany). The separation process was initialized by injecting 1 μL of the peptide sample from the autosampler unit. In the first separation step, the peptides (1 µg) were loaded to the Acclaim^®^ PepMap 100 C_18_ trap column (5 μm particles, 100 Å, 300 μmi.d. × 2 cm) via the mobile phase (98% water, 2% ACN, 270717, Merck, Germany, and 0.1% FA, 543804, Merck, Germany) at an 8 μL/min flow-rate. In the second step, the peptides were eluted from a trap column to an Acclaim^®^ PepMap RSLC analytical column C_18_ (2 μm particles, 100 Å, 75 μmi.d. × 15 cm). Lastly, the fractions of peptides from analytical column were sprayed directly into the ion trap of the mass spectrometer by mobile phase A (98% water, 2% ACN and 0.1% FA) and phase B (95% ACN, 5% water and 0.1% FA). The elution gradient started from 96%:4% and reached 65%:35% (A:B) during a 100 min run time at a 0.4 μL·min^−1^ flowrate. The acquisition parameters for MS spectra were set as follows: positive ionization mode, enhanced resolution mode, ion change control (ICC) target up to 400,000 compounds, maximum accumulation time up to 50 ms, and scan range of 300–1300 *m*/*z*. For MS/MS spectra, acquisition parameters were used: Xtreme resolution mode, ICC target up to 500,000 compounds, maximum accumulation time up to 100 ms, and isolation width of 2.2 *m*/*z*. Once the precursor ion was selected for one MS/MS spectrum, its active exclusion was performed within a 0.25 min release time.

### 4.6. Protein Database Search

The acquired raw data were assessed via Compass Data Analysis software (version 4.2, Bruker Daltonik GmbH, Germany). ProteinScape software (version 3.1.2 450, Bruker Daltonik GmbH, Bremen, Gemany) served as the platform for searching the proteins in the SwissProt database (settings: Homo sapiens, SwissProt_2016_03) utilizing the Mascot search engine (version 2.4.0, Matrix Science, London, UK) under the following conditions: taxonomy—Homo sapiens (human); enzyme—trypsin; fixed modifications—carbamidomethyl (C); variable modifications—oxidation (M); allowed missed cleavages—up to 2; peptide charge +2, +3; minimum peptide length—3; protein assessment: false discovery rate (FDR) < 1%, minimum two unique peptides. The data files generated by Mascot were exported and examined by Scaffold 4.4.7 software (see below).

### 4.7. Protein Identification, Quantification, and Pathway Analysis

The bioinformatics tool Scaffold 4.4.7 (trial version, Proteome Software Inc., Portland, OR, USA) and gene ontology database (NCBI_2016_03) were used for the identification of the proteins and for their quantification. To perform the label-free protein quantification, the data were normalized, and a quantitative method referred to as the total spectra; count was used. A *t*-test (*p* ≤ 0.05) was applied to select datasets from biological triplicates of MCF-7/WT cells and MCF-7/DOX-1 cell lines.

Pathway Studio 11.2 software (trial version, Ariadne Genomics, Rockville, MD, USA) that utilizes MedScan as the literature mining program for searching publicly available databases such as PubMed for relationships between entities was used for signaling pathway analysis. The ResNet and ChemEffect databases were utilized for gene set enrichment analysis (GSEA) and subnetwork enrichment analysis (SNEA) using the Mann–Whitney U test (MWW test, *p* ≤ 0.05).

### 4.8. Immunohistochemistry Staining

The MCF-7/WT and MCF-7/DOX-1 cells were fixed onto glass coverslips by 4% paraformaldehyde (P6148, Merck, Germany) for 10 min, and subsequently stained with either monoclonal antibody anti-CD24 conjugated to Milli-Mark™ fluorescein isothiocyanate (FCMAB188F, anti-CD24–FITC; clone SN3, 1:15 (*v*/*v*) dilution, EMD Millipore Corporation, Burlington, MA, USA) or monoclonal antibody anti-human CD44 conjugated to R-phycoerythrin (CBL154P, anti-CD44–R-PE; clone F10-44-2, dilution 1:15, EMD Millipore Corporation, Burlington, MA, USA) at room temperature (RT) for 1 h. After washing with PBS (524650, Merck, Germany) containing 0.05% Tween-20 (P9416, Merck, Germany), the cell nuclei were stained with Hoechst 34580 (H21486, 2 µg/mL, Thermo Fisher Scientific, USA) for 10 min. The cells were visualized using a confocal microscope Zeiss LSM 5 DUO (Zeiss, Germany) at λ_exc_ = 488 nm and λ_em_ = 515–550 nm for anti-CD24-FITC, at λ_exc_ = 560nm and λ_em_ > 573 nm for anti-CD44-R-PE, and at λ_exc_ = 405 nm and λ_em_ = 420–480 nm for Hoechst 34580. The photomicrographs were scanned using a 63× objective.

## 5. Conclusions

In this study, we performed multidimensional mass spectrometry analysis of the proteome isolated from the MCF-7 breast cancer cell line before and after treatment with chemotherapeutic drug doxorubicin, which revealed 1803 unique proteins. The label-free quantification total spectra count method determined 370 proteins. In this case, label-free quantification was advantageous over the labeling method, as it avoided any impurities which might rise from the labeling such that the quantification was more straightforward. The main consequence of applied chemotherapeutic drug DOX was the inactivation of Rho GTPases *RAC1*, *CDC42*, and *RHOA* by *ARHGAP1*, which participated in the TGF-beta signaling pathway and resulted in the weakening of cell adhesion, morphological changes in the cancer cells, increased motility, and metastasis. The proposed mechanism of weakening of the cellular adhesion through the inactivation of Rho GTPases by *ARHGAP1* highlights the essential regulating function of *ARHGAP1* during epithelial–mesenchymal transition. Moreover, this mechanism clarifies the relationship between the treatment with a high dose of doxorubicin and metastasis, as well as the involved TGF-beta signaling pathway. Accordingly, we show that the EMT process is mandatory for metastasis to occur.

Regarding the activity measure and control of upregulated small GTPases in the presence of DOX, in general, the most obvious way to regulate the protein activity is to control its total amount in a sample. However, as the biological activity of proteins is the incarnation of several functions and interactions with other molecules, one could assume that the upregulation of the *RAC1*, *CDC42*, and *RHOA* GTPases does not necessarily reflect their activity. Moreover, as the negative regulator *ARHGAP1*, which promotes GTPase hydrolysis, thereby inactivating them, was coincidently upregulated, it should be emphasized that promoting GTPase activity in this case means their switch into a GDP-inactive conformation. Importantly, simultaneous upregulation of GTPases and *ARHGAP1* definitely plays a role in the TGF-beta and adherens junctions assembly signaling pathways through crosstalk among *RAC1*, *CDC42*, and *RHOA* GTPases, which results in a cellular response by means of morphological remodeling. It should be noted that each GTPase staying separately would have a divergent biological function.

Considering immunohistochemical staining, studying the GTPase activation level could be as challenging, as in the case of statistically low significant quantitative measures of *CD44* or *CD24* expression. Nevertheless, quantitative evaluation of the metastatic potential of MCF-7 cells treated with DOX, together with a further analysis of the upregulated GTPase activation level, will be performed in our future study. To analyze the GTPase activity, one can measure the decrease in GTP form, increase in GDP form, release of Pi, or even the level of *ARHGAP1* [8].

Regardless of the abovementioned simultaneous upregulation, another crucial question arises: How does the upregulation (expression increase) of both GTPases and *ARHGAP1* relate to the switch from GTP to GDP? The answer probably lies in the added high dose of highly cytotoxic doxorubicin, which is known to activate the overexpression of Rho GTPases.

In spite of these promising findings, the use of just one breast cancer cell line is considered constricting. To validate these results, we will use other appropriate breast cancer cell lines in future research. Lastly, the obtained results will be verified in our ongoing clinical proteomics study of the breast cancer proteome isolated from real patients at different cancer stages, with a focus on the crosstalk among different signaling pathways.

## Figures and Tables

**Table 1:** Gene set enrichment analysis of Ariadne cell process pathways and signal transduction pathways (MWW test, *p* ≤ 0.05).

Ariadne Cell Process Pathway	Total Entities	Overlap	Overlapping Entities	*p*-Value	Jaccard Similarity
VEGF signaling	82	6	*RPS6KB1*, *MTOR*, *RAC1*, *IQGAP1*, *CDC42*, *HSPB1*	0.016	0.013
p38 MAPK/MAPK14 signaling	41	4	*RAC1*, *CDC42*, *HSPB1*, *RHOA*	0.020	0.010
Ras–GAP regulation signaling	47	4	*RAC1*, *CDC42*, *DYNC1H1*, *STAT1*	0.032	0.010
TGF-beta signaling	75	5	*RPS6KB1*, *MTOR*, *RAC1*, *CDC42*, *RHOA*	0.041	0.011
**Signal Transduction Pathway**					
Translation	84	15	*EIF4G1*, *EEF1G*, *EEF1D*, *ETF1*, *RARS*, *AARS*, *IARS*, *RPS6KB1*, *EPRS*, *VARS*, *QARS*, *DARS*, *NARS*, *EIF5B*, *EIF4A1*	4.28 × 10^−7^	0.034
Endosomal recycling	50	7	*PDCD6IP*, *ARF1*, *CDC42*, *RAB11A*, *TFRC*, *EHD4*, *DNM2*	0.003	0.017
Adherens junction assembly (cadherins)	39	6	*CTNND1*, *RHOA*, *JUP*, *RAC1*, *CDC42*, *IQGAP1*	0.004	0.015
Golgi to endosome transport	59	7	*VPS35*, *SNX2*, *STX16*, *NSF*, *NAPA*, *ARF1*, *DNM2*	0.008	0.017
SRCAP chromatin remodeling	18	3	*RUVBL2*, *ACTL6A*, *RUVBL1*	0.032	0.008
Secretory pathway: Golgi transport	36	4	*RAB2A*, *SEC24C*, *SEC16A*, *ARF1*	0.052	0.010

**Table 2:** Up-regulated proteins involved in TGF-beta and Adherens Junction Assembly pathways in MCF-7/DOX-1 cells (MWW test, *p* ≤ 0.05).

Accession	Gene	Protein	Fold Change	*t*-Test (*p* ≤ 0.05)	MCF-7/DOX-1 Regulated
Ras-related C3 botulinum toxin substrate1	*RAC1*	RAC1_HUMAN	13.8	0.003	up
Cell division control protein 42 homolog	*CDC42*	CDC42_HUMAN	19.8	0.002	up
Transforming protein RhoA	*RHOA*	RHOA_HUMAN	18.9	0.021	up
Ras GTPase-activating-like protein	*IQGAP1*	IQGA1_HUMAN	2.7	0.014	up
Rho GTPase-activating protein 1	*ARHGAP1*	RHG01_HUMAN	15.6	0.004	up
Catenin gamma	*JUP*	PLAK_HUMAN	2.5	0.023	up
p120 catenin	*CTNND1*	CTND1_HUMAN	13.2	0.004	up
Serine/threonine-pro-tein kinase mTOR	*MTOR*	MTOR_HUMAN	5.1	0.0001	up
Ribosomal protein S6 kinase beta-1	*RPS6KB1*	KS6B1_HUMAN	3.1	0.040	up
CD44 antigen	*CD44*	CD44_HUMAN	4.9	0.012	up

**Table 3:** Gene set enrichment analysis of proteins regulating cell processes (MWW test, *p* ≤ 0.05).

Proteins/Chemicals Regulating Cell Processes	Total Entities	Overlap—%	MCF-7/DOX-1	*p*-Value	Jaccard Similarity
Cell motility	3208	70 2	*STAT1*, *CLIC1*, *FOXA1*, *TGM2*, *NAMPT*, *IPO7*, *TACSTD2*, *CAPN1*, *PKM*, *CDC42*, *AIFM1*, *ARL1*, *GNA13*, *HSPA2*, *MYO1C*, *APEX1*, *MEMO1*, *ATP1A1*, *TRIM16*, *DIAPH1*, *HNRNPK*, *SND1*, *MTA1*, *RUVBL1*, *CYFIP1*, *MYO1B*, *GNAS*, *G6PD*, *FLOT1*, *YWHAZ*, *RHOA*, *NSF*, *HUWE1*, *IQGAP1*, *MYOF*, *DNM2*, *ACLY*, *ATP1B1*, *NPM1*, *KDM1A*, *CD44*, *PRDX1*, *AGR2*, *FAM129B*, *MUC5AC*, *FLII*, *MTOR*, *CTNND1*, *PAPOLA*, *ANXA7*, *RAC1*, *RAB11A*, *DNM1L*, *NARS*, *GNB2L1*, *WDR1*, *HSPB1*, *RPS6KB1*, *S100P*, *TWF1*, *STOML2*, *SLC12A2*, *JUP*, *MARCKS*, *CUL1*, *KHSRP*, *YWHAB*, *GOLPH3*, *ARHGAP1*, *FERMT2*	8.177 × 10^−16^	0.020
Epithelial-to-mesenchymal transition	2423	53 2	*STAT1*, *DNAJB6*, *ACTL6A*, *HYOU1*, *FOXA1*, *TGM2*, *NAMPT*, *TACSTD2*, *BLVRA*, *PKM*, *CDC42*, *GNA13*, *PRMT5*, *RUVBL2*, *MEMO1*, *PRDX2*, *TRIM16*, *GLRX3*, *MTA1*, *KIF5B*, *GNAS*, *ARF1*, *YWHAZ*, *SENP3*, *RHOA*, *IQGAP1*, *MYOF*, *EEF1D*, *ACLY*, *ATP1B1*, *DSP*, *CTBP2*, *KDM1A*, *CD44*, *PRDX1*, *ALDH7A1*, *AGR2*, *MTOR*, *CTNND1*, *IDH2*, *RAC1*, *DNM1L*, *GNB2L1*, *HSPB1*, *RPS6KB1*, *S100P*, *TWF1*, *CKAP4*, *JUP*, *CUL1*, *YWHAB*, *ARHGAP1*, *FERMT2*	0.012	0.019

## Data Availability

The data presented in this study are available in the Supplementary Materials of this article.

## Supplementary Materials

The following supporting information can be downloaded at https://www.mdpi.com/article/10.3390/ijms241411352/s1.
