# Supplementary material for: The Role of ARHGAP1 in Rho GTPase Inactivation during Metastasizing of Breast Cancer Cell Line MCF-7 after Treatment with Doxorubicin
Source: Int J Mol Sci. 2023 Jul 12;24(14):11352. doi: 10.3390/ijms241411352 (PMC10379778; doi:10.3390/ijms241411352)
Supplement: Supplementary file 1 [file ijms-24-11352-s001.zip › ijms-2460125-supplementary.pdf]

Table S1
List of identified and quantified proteins.

#	Visible?	Starred?	Identified Proteins (1803)	Accession Number	Molecular Weight	Protein Grouping Ambiguity	T-Test (P-Value): (p <= 0.05)	Fold Change by Category	MCF-7[1]	MCF-7[2]	MCF-7[3]	MCF-7/DOX[1]	MCF-7/DOX[2]	MCF-7/DOX[3]
1	TRUE	Empty	[Pyruvate dehydrogenase [acetyl-transferring]]-phosphatase 1, mitochondrial OS=Homo sapiens GN=PDP1 PE=1 SV=3	PDP1_HUMAN	?		0.184606477	INF	0	0	0	0.74522	0	1.96
2	TRUE	Empty	1,4-alpha-glucan-branching enzyme OS=Homo sapiens GN=GBE1 PE=1 SV=3	GLGB_HUMAN	80 kDa		0.145322396	INF	0	0	0	1.04	0.84713	0
3	TRUE	Empty	10 kDa heat shock protein, mitochondrial OS=Homo sapiens GN=HSPE1 PE=1 SV=2	CH10_HUMAN	11 kDa		0.211910961	1.3	15.472	13.561	23.939	23.847	21.178	22.555
4	TRUE	Empty	116 kDa U5 small nuclear ribonucleoprotein component OS=Homo sapiens GN=EFTUD2 PE=1 SV=1	U5S1_HUMAN	?	TRUE	.013446306	3.778455175	2.03	2.0863	4.73	10.433	15.248	9.78
5	TRUE	Empty	14 kDa phosphohistidine phosphatase OS=Homo sapiens GN=PHPT1 PE=1 SV=1	PHP14_HUMAN	?		0.6126083	0.4	0	9.86	0	2.57	0	1.96
6	TRUE	Empty	14-3-3 protein beta/alpha OS=Homo sapiens GN=YWHAB PE=1 SV=3	1433B_HUMAN	?	TRUE	.027938477	1.564532112	59.679	77.195	54.86	83.464	108.43	108.08
7	TRUE	Empty	14-3-3 protein epsilon OS=Homo sapiens GN=YWHAE PE=1 SV=1	1433E_HUMAN	?	TRUE	.396415565	1.2	59.679	128.31	112.71	102.09	126.22	139.09
8	TRUE	Empty	14-3-3 protein eta OS=Homo sapiens GN=YWHAH PE=1 SV=4	1433F_HUMAN	28 kDa	TRUE	.066171799	1.7	37.575	70.936	47.878	68.56	92.338	105.26
9	TRUE	Empty	14-3-3 protein gamma OS=Homo sapiens GN=YWHAG PE=1 SV=2	1433G_HUMAN	28 kDa	TRUE	0.06379069	1.6	46.417	76.152	49.873	72.286	104.2	106.2
10	TRUE	Empty	14-3-3 protein sigma OS=Homo sapiens GN=SFN PE=1 SV=1	1433S_HUMAN	?	TRUE	.068282107	1.6	41.996	73.022	57.853	70.796	103.35	104.32
11	TRUE	Empty	14-3-3 protein theta OS=Homo sapiens GN=YWHAQ PE=1 SV=1	1433T_HUMAN	28 kDa	TRUE	.068239458	1.6	41.996	79.281	68.825	80.484	109.28	115.59
12	TRUE	Empty	14-3-3 protein zeta/delta OS=Homo sapiens GN=YWHAZ PE=1 SV=1	1433Z_HUMAN	?	TRUE	.042246178	1.417111594	106.1	101.19	95.756	117.74	166.04	145.67
13	TRUE	Empty	1-phosphatidylinositol 4,5-bisphosphate phosphodiesterase eta-1 OS=Homo sapiens GN=PLCH1 PE=1 SV=1	PLCH1_HUMAN	?	TRUE	.625960051	0.6	2.03	2.0863	0	0	2.14	0
14	TRUE	Empty	2,4-dienoyl-CoA reductase, mitochondrial OS=Homo sapiens GN=DECR1 PE=1 SV=1	DECR_HUMAN	?		0.007360905	4.986205038	0	2.0863	1.49	5.17	5.99	8.81
15	TRUE	Empty	2'-5'-oligoadenylate synthase 1 OS=Homo sapiens GN=OAS1 PE=1 SV=4	OAS1_HUMAN	?	TRUE	.373900966	INF	0	0	0	1.04	0	0
16	TRUE	Empty	26S protease regulatory subunit 10B OS=Homo sapiens GN=PSMC6 PE=1 SV=1	PRS10_HUMAN	44 kDa	TRUE	.315162174	1.3	6.631	11.475	7.97	8.74	11.86	13.157
17	TRUE	Empty	26S protease regulatory subunit 4 OS=Homo sapiens GN=PSMC1 PE=1 SV=1	PRS4_HUMAN	?	TRUE	.038588035	4.698508276	0	1.0432	2.24	3.61	6.71	8.81
18	TRUE	Empty	26S protease regulatory subunit 6A OS=Homo sapiens GN=PSMC3 PE=1 SV=3	PRS6A_HUMAN	49 kDa	TRUE	.881302386	1	8.13	14.604	14.962	9.78	14.401	13.157
19	TRUE	Empty	26S protease regulatory subunit 6B OS=Homo sapiens GN=PSMC4 PE=1 SV=2	PRS6B_HUMAN	?	TRUE	.574786554	0.6	2.03	1.0432	6.22	4.13	0.84713	0.93978
20	TRUE	Empty	26S protease regulatory subunit 7 OS=Homo sapiens GN=PSMC2 PE=1 SV=3	PRS7_HUMAN	?	TRUE	.004910701	22.15988611	0	0	0.99746	9.78	6.71	5.87
21	TRUE	Empty	26S protease regulatory subunit 8 OS=Homo sapiens GN=PSMC5 PE=1 SV=1	PRS8_HUMAN	?	TRUE	.186604005	2	2.03	2.0863	4.73	5.17	3.85	9.78
22	TRUE	Empty	26S proteasome non-ATPase regulatory subunit 1 OS=Homo sapiens GN=PSMD1 PE=1 SV=2	PSMD1_HUMAN	?		0.038342701	3.655538651	4.06	1.0432	4.73	8.74	17.79	12.217

23	TRUE	Empty	26S proteasome non-ATPase regulatory subunit 11 OS=Homo sapiens GN=PSMD11 PE=1 SV=3	PSD11_HUMAN	?		0.038714573	5.189657513	4.06	0	0	10.433	5.99	6.85
24	TRUE	Empty	26S proteasome non-ATPase regulatory subunit 12 OS=Homo sapiens GN=PSMD12 PE=1 SV=3	PSD12_HUMAN	?		0.150165826	INF	0	0	0	0	2.14	4.89
25	TRUE	Empty	26S proteasome non-ATPase regulatory subunit 13 OS=Homo sapiens GN=PSMD13 PE=1 SV=2	PSD13_HUMAN	?		0.00945161	17.95590801	0.99746	0	0	5.65	4.57	8.81
26	TRUE	Empty	26S proteasome non-ATPase regulatory subunit 2 OS=Homo sapiens GN=PSMD2 PE=1 SV=3	PSMD2_HUMAN	?	TRUE	.009110702	4.882133883	0	2.0863	8.71	16.395	16.943	20.675
27	TRUE	Empty	26S proteasome non-ATPase regulatory subunit 3 OS=Homo sapiens GN=PSMD3 PE=1 SV=2	PSMD3_HUMAN	?		0.062928597	5.8	0	1.0432	3.98	8.26	5.0828	15.037
28	TRUE	Empty	26S proteasome non-ATPase regulatory subunit 4 OS=Homo sapiens GN=PSMD4 PE=1 SV=1	PSMD4_HUMAN	?	TRUE	.985320714	1	2.03	9.86	15.959	12.669	7.42	7.83
29	TRUE	Empty	26S proteasome non-ATPase regulatory subunit 5 OS=Homo sapiens GN=PSMD5 PE=1 SV=3	PSMD5_HUMAN	?		0.004670138	26.62482706	0.99746	0	0	5.17	9.85	11.277
30	TRUE	Empty	26S proteasome non-ATPase regulatory subunit 6 OS=Homo sapiens GN=PSMD6 PE=1 SV=1	PSMD6_HUMAN	?		0.050468881	4.9	4.06	2.0863	1.49	6.707	20.331	15.037
31	TRUE	Empty	26S proteasome non-ATPase regulatory subunit 7 OS=Homo sapiens GN=PSMD7 PE=1 SV=2	PSMD7_HUMAN	37 kDa	TRUE	.184903748	5	0	2.0863	2.24	4.13	4.57	16.916
32	TRUE	Empty	26S proteasome non-ATPase regulatory subunit 8 OS=Homo sapiens GN=PSMD8 PE=1 SV=2	PSMD8_HUMAN	40 kDa		0.136121777	INF	0	0	0	1.04	0	0.93978
33	TRUE	Empty	26S proteasome non-ATPase regulatory subunit 9 OS=Homo sapiens GN=PSMD9 PE=1 SV=3	PSMD9_HUMAN	?		0.450690138	0.5	0	2.0863	4.73	0.74522	1.43	0.93978
34	TRUE	Empty	28 kDa heat- and acid-stable phosphoprotein OS=Homo sapiens GN=PDAP1 PE=1 SV=1	HAP28_HUMAN	21 kDa	TRUE	.459398381	0.7	2.03	8.54	12.967	2.09	6.71	5.87
35	TRUE	Empty	28S ribosomal protein S18c, mitochondrial OS=Homo sapiens GN=MRPS18C PE=1 SV=1	RT18C_HUMAN	16 kDa		0.388590446	3	0	1.0432	0	2.57	0	0.93978
36	TRUE	Empty	28S ribosomal protein S22, mitochondrial OS=Homo sapiens GN=MRPS22 PE=1 SV=1	RT22_HUMAN	?		0.373900966	INF	0	0	0	0	0	1.96
37	TRUE	Empty	28S ribosomal protein S23, mitochondrial OS=Homo sapiens GN=MRPS23 PE=1 SV=2	RT23_HUMAN	22 kDa	TRUE	0.1639255	2	2.03	1.0432	0	2.57	2.14	1.96
38	TRUE	Empty	28S ribosomal protein S25, mitochondrial OS=Homo sapiens GN=MRPS25 PE=1 SV=1	RT25_HUMAN	?		0.053421057	INF	0	0	0	2.57	0.84713	3.91
39	TRUE	Empty	28S ribosomal protein S27, mitochondrial OS=Homo sapiens GN=MRPS27 PE=1 SV=3	RT27_HUMAN	?		0.373900966	INF	0	0	0	0	0	3.91
40	TRUE	Empty	28S ribosomal protein S29, mitochondrial OS=Homo sapiens GN=DAP3 PE=1 SV=1	RT29_HUMAN	?		0.136121777	INF	0	0	0	1.04	0	0.93978
41	TRUE	Empty	2-oxoglutarate dehydrogenase, mitochondrial OS=Homo sapiens GN=OGDH PE=1 SV=3	ODO1_HUMAN	?	TRUE	.084566581	INF	0	0	0	2.09	0.84713	0.93978
42	TRUE	Empty	2-oxoisovalerate dehydrogenase subunit alpha, mitochondrial OS=Homo sapiens GN=BCKDHA PE=1 SV=2	ODBA_HUMAN	?		0.192836343	INF	0	0	0	0	2.14	0.93978
43	TRUE	Empty	3'(2'),5'-bisphosphate nucleotidase 1 OS=Homo sapiens GN=BPNT1 PE=1 SV=1	BPNT1_HUMAN	?		0.360920531	4.5	0	1.0432	0	3.61	0	0.93978
44	TRUE	Empty	39S ribosomal protein L12, mitochondrial OS=Homo sapiens GN=MRPL12 PE=1 SV=2	RM12_HUMAN	21 kDa		0.728175975	1.1	2.03	8.54	4.73	5.17	5.99	5.87
45	TRUE	Empty	39S ribosomal protein L46, mitochondrial OS=Homo sapiens GN=MRPL46 PE=1 SV=1	RM46_HUMAN	32 kDa	TRUE	0.03308882	3.48079121	0	0	0.99746	0.74522	0.84713	1.96
46	TRUE	Empty	39S ribosomal protein L53, mitochondrial OS=Homo sapiens GN=MRPL53 PE=1 SV=1	RM53_HUMAN	12 kDa		0.968461748	0.9	0	0	1.49	0	0	1.96
47	TRUE	Empty	3-hydroxyacyl-CoA dehydrogenase type-2 OS=Homo sapiens GN=HSD17B10 PE=1 SV=3	HCD2_HUMAN	?		0.234985525	1.4	35.365	20.863	14.962	31.299	29.65	37.591
48	TRUE	Empty	3-hydroxyisobutyrate dehydrogenase, mitochondrial OS=Homo sapiens GN=HIBADH PE=1 SV=2	3HIDH_HUMAN	35 kDa	TRUE	.800719764	1.1	2.03	11.475	4.73	6.707	7.42	6.85

49	TRUE	Empty	3-ketoacyl-CoA thiolase, mitochondrial OS=Homo sapiens GN=ACAA2 PE=1 SV=2	THIM_HUMAN	42 kDa		0.994822471	1	0	4.1727	1.49	0.74522	1.43	3.91
50	TRUE	Empty	3-ketoacyl-CoA thiolase, peroxisomal OS=Homo sapiens GN=ACAA1 PE=1 SV=2	THIK_HUMAN	?		0.000706428	17.47288112	0	0	0.99746	6.707	5.0828	5.87
51	TRUE	Empty	3-mercaptopyruvate sulfurtransferase OS=Homo sapiens GN=MPST PE=1 SV=3	THTM_HUMAN	?	TRUE	.356219975	1.3	2.03	3.1295	4.73	5.65	4.57	3.91
52	TRUE	Empty	3-oxoacyl-[acyl-carrier-protein] synthase, mitochondrial OS=Homo sapiens GN=OXSM PE=1 SV=1	OXSM_HUMAN	?	TRUE	.373900966	INF	0	0	0	2.57	0	0
53	TRUE	Empty	40S ribosomal protein S10 OS=Homo sapiens GN=RPS10 PE=1 SV=1	RS10_HUMAN	19 kDa	TRUE	.864900777	0.9	26.524	3.1295	5.47	8.26	8.13	14.097
54	TRUE	Empty	40S ribosomal protein S11 OS=Homo sapiens GN=RPS11 PE=1 SV=3	RS11_HUMAN	18 kDa		0.038076528	4.703644293	0	0	1.49	2.57	3.85	3.91
55	TRUE	Empty	40S ribosomal protein S12 OS=Homo sapiens GN=RPS12 PE=1 SV=3	RS12_HUMAN	15 kDa		0.400534658	0.5	2.03	50.072	32.916	12.669	18.637	14.097
56	TRUE	Empty	40S ribosomal protein S13 OS=Homo sapiens GN=RPS13 PE=1 SV=2	RS13_HUMAN	17 kDa	TRUE	.046374213	5.174745601	0	0	1.49	2.57	3.85	4.89
57	TRUE	Empty	40S ribosomal protein S14 OS=Homo sapiens GN=RPS14 PE=1 SV=3	RS14_HUMAN	16 kDa		0.314645447	2.4	0	0	2.24	2.09	3.85	0.93978
58	TRUE	Empty	40S ribosomal protein S15 OS=Homo sapiens GN=RPS15 PE=1 SV=2	RS15_HUMAN	17 kDa	TRUE	.550821271	1.5	0	1.0432	3.98	2.09	1.43	2.94
59	TRUE	Empty	40S ribosomal protein S15a OS=Homo sapiens GN=RPS15A PE=1 SV=2	RS15A_HUMAN	15 kDa		0.236171395	3	6.631	0	0	4.13	4.57	11.277
60	TRUE	Empty	40S ribosomal protein S16 OS=Homo sapiens GN=RPS16 PE=1 SV=2	RS16_HUMAN	16 kDa		0.218969151	2.8	11.052	0	2.24	23.102	9.85	7.83
61	TRUE	Empty	40S ribosomal protein S17 OS=Homo sapiens GN=RPS17 PE=1 SV=2	RS17_HUMAN	16 kDa		0.981470778	1	2.03	11.475	17.954	8.26	12.707	10.338
62	TRUE	Empty	40S ribosomal protein S18 OS=Homo sapiens GN=RPS18 PE=1 SV=3	RS18_HUMAN	18 kDa	TRUE	0.73326517	1.2	8.13	1.0432	9.46	5.65	11.86	6.85
63	TRUE	Empty	40S ribosomal protein S19 OS=Homo sapiens GN=RPS19 PE=1 SV=2	RS19_HUMAN	16 kDa	TRUE	.314449511	0.7	6.631	13.561	11.969	2.57	8.13	10.338
64	TRUE	Empty	40S ribosomal protein S2 OS=Homo sapiens GN=RPS2 PE=1 SV=2	RS2_HUMAN	31 kDa		0.772904383	0.9	8.13	10.432	20.947	13.414	11.013	12.217
65	TRUE	Empty	40S ribosomal protein S20 OS=Homo sapiens GN=RPS20 PE=1 SV=1	RS20_HUMAN	?		0.364088797	0.6	6.631	2.0863	6.22	5.65	3.85	0.93978
66	TRUE	Empty	40S ribosomal protein S21 OS=Homo sapiens GN=RPS21 PE=1 SV=1	RS21_HUMAN	9 kDa		0.865712078	0.9	4.06	17.734	20.947	7.22	5.99	25.374
67	TRUE	Empty	40S ribosomal protein S24 OS=Homo sapiens GN=RPS24 PE=1 SV=1	RS24_HUMAN	?		0.21264754	2.4	4.06	0	4.73	4.13	12.707	5.87
68	TRUE	Empty	40S ribosomal protein S25 OS=Homo sapiens GN=RPS25 PE=1 SV=1	RS25_HUMAN	14 kDa		0.62678517	0.8	22.103	6.259	15.959	11.178	14.401	11.277
69	TRUE	Empty	40S ribosomal protein S26 OS=Homo sapiens GN=RPS26 PE=1 SV=3	RS26_HUMAN	13 kDa	TRUE	.358712293	2.4	8.13	1.0432	6.22	28.318	5.0828	7.83
70	TRUE	Empty	40S ribosomal protein S28 OS=Homo sapiens GN=RPS28 PE=1 SV=1	RS28_HUMAN	8 kDa		0.30958028	0.6	8.13	3.1295	8.71	2.57	6.71	3.91
71	TRUE	Empty	40S ribosomal protein S3 OS=Homo sapiens GN=RPS3 PE=1 SV=2	RS3_HUMAN	?		0.394474769	1.6	33.155	4.1727	7.97	17.885	22.873	33.832
72	TRUE	Empty	40S ribosomal protein S3a OS=Homo sapiens GN=RPS3A PE=1 SV=2	RS3A_HUMAN	30 kDa		0.138939551	2.6	2.03	2.0863	3.98	11.923	4.57	5.87
73	TRUE	Empty	40S ribosomal protein S4, X isoform OS=Homo sapiens GN=RPS4X PE=1 SV=2	RS4X_HUMAN	30 kDa	TRUE	.373104626	1.5	4.06	5.59	13.964	11.178	17.79	7.83
74	TRUE	Empty	40S ribosomal protein S5 OS=Homo sapiens GN=RPS5 PE=1 SV=4	RS5_HUMAN	23 kDa	TRUE	.046990761	6.267678335	0	3.1295	2.24	19.376	6.71	12.217

75	TRUE	Empty	40S ribosomal protein S6 OS=Homo sapiens GN=RPS6 PE=1 SV=1	RS6_HUMAN	29 kDa		0.088101989	3.1	2.03	1.0432	5.47	11.178	12.707	4.89
76	TRUE	Empty	40S ribosomal protein S7 OS=Homo sapiens GN=RPS7 PE=1 SV=1	RS7_HUMAN	22 kDa		0.151551581	0.7	15.472	9.86	9.46	6.707	7.42	9.78
77	TRUE	Empty	40S ribosomal protein S8 OS=Homo sapiens GN=RPS8 PE=1 SV=2	RS8_HUMAN	24 kDa	TRUE	.994000394	1	4.06	5.59	24.936	14.904	16.096	3.91
78	TRUE	Empty	40S ribosomal protein S9 OS=Homo sapiens GN=RPS9 PE=1 SV=3	RS9_HUMAN	23 kDa		0.41505911	2	8.13	0	0	5.17	3.85	8.81
79	TRUE	Empty	40S ribosomal protein SA OS=Homo sapiens GN=RPSA PE=1 SV=4	RSSA_HUMAN	33 kDa		0.261617919	0.6	59.679	18.777	35.908	20.121	27.108	19.735
80	TRUE	Empty	4-aminobutyrate aminotransferase, mitochondrial OS=Homo sapiens GN=ABAT PE=1 SV=3	GABT_HUMAN	56 kDa		0.339979506	1.7	6.631	3.1295	2.24	3.61	5.99	12.217
81	TRUE	Empty	4F2 cell-surface antigen heavy chain OS=Homo sapiens GN=SLC3A2 PE=1 SV=3	4F2_HUMAN	?		0.83444122	1.1	33.155	8.54	2.24	11.178	22.025	17.856
82	TRUE	Empty	4-hydroxyphenylpyruvate dioxygenase-like protein OS=Homo sapiens GN=HPDL PE=1 SV=1	HPDL_HUMAN	39 kDa		0.036639133	5.722164297	0.99746	0	0	2.09	0.84713	1.96
83	TRUE	Empty	4-trimethylaminobutyraldehyde dehydrogenase OS=Homo sapiens GN=ALDH9A1 PE=1 SV=3	AL9A1_HUMAN	?		0.037589604	5.755463957	0	1.0432	0	1.04	1.43	2.94
84	TRUE	Empty	5'-3' exoribonuclease 2 OS=Homo sapiens GN=XRN2 PE=1 SV=1	XRN2_HUMAN	?		0.738892649	0.8	0	3.1295	9.46	3.61	3.85	2.94
85	TRUE	Empty	5'-AMP-activated protein kinase catalytic subunit alpha-1 OS=Homo sapiens GN=PRKAA1 PE=1 SV=4	AAPK1_HUMAN	?	TRUE	.003651919	4.134982856	0	0.99746	0	1.04	1.43	0.93978
86	TRUE	Empty	5'-AMP-activated protein kinase subunit beta-1 OS=Homo sapiens GN=PRKAB1 PE=1 SV=4	AAKB1_HUMAN	30 kDa	TRUE	.373900966	INF	0	0	0	0	0	1.96
87	TRUE	Empty	5'-AMP-activated protein kinase subunit gamma-1 OS=Homo sapiens GN=PRKAG1 PE=1 SV=1	AAKG1_HUMAN	?	TRUE	.458310893	2.3	0	0	0.99746	1.04	0.84713	0
88	TRUE	Empty	5'-nucleotidase domain-containing protein 1 OS=Homo sapiens GN=NT5DC1 PE=1 SV=1	NT5D1_HUMAN	?		0.4598504	1.9	0	0	2.24	2.57	2.14	0.93978
89	TRUE	Empty	60 kDa heat shock protein, mitochondrial OS=Homo sapiens GN=HSPD1 PE=1 SV=2	CH60_HUMAN	?		0.148347207	1.7	172.4	78.238	66.83	219.84	164.34	147.55
90	TRUE	Empty	60 kDa SS-A/Ro ribonucleoprotein OS=Homo sapiens GN=TROVE2 PE=1 SV=2	RO60_HUMAN	?	TRUE	.678870175	1.1	4.06	5.59	7.97	4.13	7.42	7.83
91	TRUE	Empty	60S acidic ribosomal protein P0 OS=Homo sapiens GN=RPLP0 PE=1 SV=1	RLA0_HUMAN	?	TRUE	0.23991139	1.4	11.052	22.95	31.919	24.592	33.885	36.652
92	TRUE	Empty	60S acidic ribosomal protein P1 OS=Homo sapiens GN=RPLP1 PE=1 SV=1	RLA1_HUMAN	?	TRUE	.088915969	0.4	26.524	40.684	27.929	11.923	25.414	0
93	TRUE	Empty	60S acidic ribosomal protein P2 OS=Homo sapiens GN=RPLP2 PE=1 SV=1	RLA2_HUMAN	12 kDa	TRUE	.562490765	0.8	37.575	64.677	25.934	18.63	54.217	25.374
94	TRUE	Empty	60S ribosomal protein L10 OS=Homo sapiens GN=RPL10 PE=1 SV=4	RL10_HUMAN	25 kDa	TRUE	.060356148	4.1	0	0	2.24	4.13	5.0828	2.94
95	TRUE	Empty	60S ribosomal protein L10a OS=Homo sapiens GN=RPL10A PE=1 SV=2	RL10A_HUMAN	25 kDa		0.292851619	2	0	2.0863	6.22	8.74	3.85	6.85
96	TRUE	Empty	60S ribosomal protein L11 OS=Homo sapiens GN=RPL11 PE=1 SV=2	RL11_HUMAN	?	TRUE	.528645098	0.8	11.052	6.259	7.97	3.61	10.166	6.85
97	TRUE	Empty	60S ribosomal protein L12 OS=Homo sapiens GN=RPL12 PE=1 SV=1	RL12_HUMAN	?		0.529566289	0.8	35.365	30.252	18.952	38.006	9.85	16.916
98	TRUE	Empty	60S ribosomal protein L13 OS=Homo sapiens GN=RPL13 PE=1 SV=4	RL13_HUMAN	?	TRUE	.346367754	1.7	2.03	3.1295	15.959	12.669	12.707	10.338
99	TRUE	Empty	60S ribosomal protein L13a OS=Homo sapiens GN=RPL13A PE=1 SV=2	RL13A_HUMAN	24 kDa		0.984363485	1	6.631	0	2.24	0.74522	3.85	5.87
100	TRUE	Empty	60S ribosomal protein L14 OS=Homo sapiens GN=RPL14 PE=1 SV=4	RL14_HUMAN	23 kDa		0.864007608	0.8	0	0	8.71	3.61	1.43	1.96

101	TRUE	Empty	60S ribosomal protein L15 OS=Homo sapiens GN=RPL15 PE=1 SV=2	RL15_HUMAN	?		0.9604117	1	2.03	2.0863	4.73	1.04	4.57	3.91
102	TRUE	Empty	60S ribosomal protein L17 OS=Homo sapiens GN=RPL17 PE=1 SV=3	RL17_HUMAN	?	TRUE	.251980677	2	0	3.1295	0.99746	2.09	3.85	1.96
103	TRUE	Empty	60S ribosomal protein L18 OS=Homo sapiens GN=RPL18 PE=1 SV=2	RL18_HUMAN	?	TRUE	.383730047	1.5	6.631	0	5.47	7.22	5.99	5.87
104	TRUE	Empty	60S ribosomal protein L18a OS=Homo sapiens GN=RPL18A PE=1 SV=2	RL18A_HUMAN	21 kDa	TRUE	.210232473	2.4	0	6.259	6.22	3.61	14.401	13.157
105	TRUE	Empty	60S ribosomal protein L21 OS=Homo sapiens GN=RPL21 PE=1 SV=2	RL21_HUMAN	19 kDa	TRUE	.231293643	2.7	0	0	6.22	3.61	5.99	9.78
106	TRUE	Empty	60S ribosomal protein L22 OS=Homo sapiens GN=RPL22 PE=1 SV=2	RL22_HUMAN	15 kDa	TRUE	.553223062	0.8	15.472	6.259	14.962	11.178	11.013	8.81
107	TRUE	Empty	60S ribosomal protein L23 OS=Homo sapiens GN=RPL23 PE=1 SV=1	RL23_HUMAN	15 kDa		0.53560817	1.4	15.472	1.0432	11.969	8.74	15.248	15.037
108	TRUE	Empty	60S ribosomal protein L23a OS=Homo sapiens GN=RPL23A PE=1 SV=1	RL23A_HUMAN	18 kDa	TRUE	0.16626643	2	8.13	0	4.73	10.433	7.42	9.78
109	TRUE	Empty	60S ribosomal protein L24 OS=Homo sapiens GN=RPL24 PE=1 SV=1	RL24_HUMAN	18 kDa		0.744157408	1.2	2.03	1.0432	7.97	2.57	5.99	5.87
110	TRUE	Empty	60S ribosomal protein L27 OS=Homo sapiens GN=RPL27 PE=1 SV=2	RL27_HUMAN	16 kDa	TRUE	.724509257	1.3	2.03	0	4.73	2.57	5.0828	1.96
111	TRUE	Empty	60S ribosomal protein L27a OS=Homo sapiens GN=RPL27A PE=1 SV=2	RL27A_HUMAN	17 kDa		0.788325767	0.9	6.631	4.1727	11.969	2.57	9.85	8.81
112	TRUE	Empty	60S ribosomal protein L3 OS=Homo sapiens GN=RPL3 PE=1 SV=2	RL3_HUMAN	46 kDa	TRUE	.145654847	2.4	0	2.0863	7.97	8.26	5.99	9.78
113	TRUE	Empty	60S ribosomal protein L30 OS=Homo sapiens GN=RPL30 PE=1 SV=2	RL30_HUMAN	13 kDa		0.413689219	1.8	0	3.1295	17.954	7.22	14.401	15.976
114	TRUE	Empty	60S ribosomal protein L31 OS=Homo sapiens GN=RPL31 PE=1 SV=1	RL31_HUMAN	?		0.730226587	1.2	19.893	0	6.22	11.923	10.166	11.277
115	TRUE	Empty	60S ribosomal protein L32 OS=Homo sapiens GN=RPL32 PE=1 SV=2	RL32_HUMAN	16 kDa		0.166541107	2.4	0	2.0863	4.73	4.13	4.57	8.81
116	TRUE	Empty	60S ribosomal protein L36 OS=Homo sapiens GN=RPL36 PE=1 SV=3	RL36_HUMAN	12 kDa		0.681500123	1.5	0	0	6.22	0.74522	4.57	5.87
117	TRUE	Empty	60S ribosomal protein L38 OS=Homo sapiens GN=RPL38 PE=1 SV=2	RL38_HUMAN	8 kDa		0.196156141	2.1	2.03	0	5.47	4.13	6.71	5.87
118	TRUE	Empty	60S ribosomal protein L4 OS=Homo sapiens GN=RPL4 PE=1 SV=5	RL4_HUMAN	48 kDa	TRUE	.197322838	2.2	0	6.259	23.939	23.102	25.414	16.916
119	TRUE	Empty	60S ribosomal protein L5 OS=Homo sapiens GN=RPL5 PE=1 SV=3	RL5_HUMAN	34 kDa	TRUE	.983841311	1	4.06	12.518	28.926	13.414	16.943	15.037
120	TRUE	Empty	60S ribosomal protein L6 OS=Homo sapiens GN=RPL6 PE=1 SV=3	RL6_HUMAN	33 kDa	TRUE	.857047964	0.9	33.155	2.0863	22.942	20.121	19.484	13.157
121	TRUE	Empty	60S ribosomal protein L7 OS=Homo sapiens GN=RPL7 PE=1 SV=1	RL7_HUMAN	29 kDa		0.631734019	0.7	37.575	4.1727	16.957	11.923	13.554	17.856
122	TRUE	Empty	60S ribosomal protein L7a OS=Homo sapiens GN=RPL7A PE=1 SV=2	RL7A_HUMAN	30 kDa		0.99743936	1	11.052	10.432	20.947	11.923	13.554	16.916
123	TRUE	Empty	60S ribosomal protein L9 OS=Homo sapiens GN=RPL9 PE=1 SV=1	RL9_HUMAN	22 kDa		0.012513274	17.88151906	0	0.99746	0	3.61	8.13	5.87
124	TRUE	Empty	6-phosphogluconate dehydrogenase, decarboxylating OS=Homo sapiens GN=PGD PE=1 SV=3	6PGD_HUMAN	?		0.121859819	1.8	26.524	9.86	10.972	26.083	23.72	34.772
125	TRUE	Empty	6-phosphogluconolactonase OS=Homo sapiens GN=PGLS PE=1 SV=2	6PGL_HUMAN	28 kDa		0.515625178	0.7	2.03	10.432	9.46	2.09	6.71	6.85
126	TRUE	Empty	78 kDa glucose-regulated protein OS=Homo sapiens GN=HSPA5 PE=1 SV=2	GRP78_HUMAN	72 kDa	TRUE	.684131215	1.1	125.99	44.856	63.837	82.719	98.268	86.46

127	TRUE	Empty	7-dehydrocholesterol reductase OS=Homo sapiens GN=DHCR7 PE=1 SV=1	DHCR7_HUMAN	54 kDa		0.0000058929	15.97858561	0	0	0.99746	5.65	5.0828	5.87
128	TRUE	Empty	Abl interactor 1 OS=Homo sapiens GN=ABI1 PE=1 SV=4	ABI1_HUMAN	?	TRUE	.862954764	1.1	0	4.1727	1.49	1.04	1.43	3.91
129	TRUE	Empty	Acetolactate synthase-like protein OS=Homo sapiens GN=ILVBL PE=1 SV=2	ILVBL_HUMAN	68 kDa	TRUE	.022978995	5.774148306	2.03	0	0	2.09	5.0828	4.89
130	TRUE	Empty	Acetyl-CoA acetyltransferase, cytosolic OS=Homo sapiens GN=ACAT2 PE=1 SV=2	THIC_HUMAN	?	TRUE	.548000861	0.6	0	18.777	5.47	3.61	3.85	6.85
131	TRUE	Empty	Acetyl-CoA acetyltransferase, mitochondrial OS=Homo sapiens GN=ACAT1 PE=1 SV=1	THIL_HUMAN	?		0.450926579	1.3	4.06	4.1727	0.99746	3.61	5.0828	3.91
132	TRUE	Empty	Acidic leucine-rich nuclear phosphoprotein 32 family member A OS=Homo sapiens GN=ANP32A PE=1 SV=1	AN32A_HUMAN	29 kDa	TRUE	0.44716148	1.4	4.06	17.734	21.944	25.337	20.331	14.097
133	TRUE	Empty	Acidic leucine-rich nuclear phosphoprotein 32 family member B OS=Homo sapiens GN=ANP32B PE=1 SV=1	AN32B_HUMAN	?	TRUE	.767278136	1.1	11.052	15.648	19.949	18.63	17.79	13.157
134	TRUE	Empty	Acidic leucine-rich nuclear phosphoprotein 32 family member E OS=Homo sapiens GN=ANP32E PE=1 SV=1	AN32E_HUMAN	?	TRUE	.144728695	2.4	0	6.259	1.49	5.65	5.99	8.81
135	TRUE	Empty	Aconitate hydratase, mitochondrial OS=Homo sapiens GN=ACO2 PE=1 SV=2	ACON_HUMAN	85 kDa	TRUE	.020046247	15.41725984	0	0	0.99746	4.13	3.85	7.83
136	TRUE	Empty	Actin, alpha cardiac muscle 1 OS=Homo sapiens GN=ACTC1 PE=1 SV=1	ACTC_HUMAN	42 kDa	TRUE	.121489978	0.7	316.08	230.54	192.51	204.94	149.1	138.15
137	TRUE	Empty	Actin, cytoplasmic 1 OS=Homo sapiens GN=ACTB PE=1 SV=1	ACTB_HUMAN	42 kDa	TRUE	.176650393	0.6	778.03	346.33	409.96	351.74	261.76	237.77
138	TRUE	Empty	Actin-like protein 6A OS=Homo sapiens GN=ACTL6A PE=1 SV=1	ACL6A_HUMAN	?	TRUE	.018699122	3.627208844	0	1.0432	0.99746	2.09	2.14	1.96
139	TRUE	Empty	Actin-related protein 2 OS=Homo sapiens GN=ACTR2 PE=1 SV=1	ARP2_HUMAN	?		0.140292063	1.7	2.03	5.59	9.46	8.26	11.013	10.338
140	TRUE	Empty	Actin-related protein 2/3 complex subunit 1B OS=Homo sapiens GN=ARPC1B PE=1 SV=3	ARC1B_HUMAN	41 kDa	TRUE	.063396291	3.7	0	0	3.98	5.17	4.57	4.89
141	TRUE	Empty	Actin-related protein 2/3 complex subunit 2 OS=Homo sapiens GN=ARPC2 PE=1 SV=1	ARPC2_HUMAN	34 kDa		0.066514209	3	6.631	4.1727	0	14.904	10.166	7.83
142	TRUE	Empty	Actin-related protein 2/3 complex subunit 3 OS=Homo sapiens GN=ARPC3 PE=1 SV=3	ARPC3_HUMAN	21 kDa		0.501412314	1.8	0	1.0432	2.24	4.13	1.43	0.93978
143	TRUE	Empty	Actin-related protein 2/3 complex subunit 4 OS=Homo sapiens GN=ARPC4 PE=1 SV=3	ARPC4_HUMAN	?		0.139596743	2.2	4.06	4.1727	1.49	3.61	8.13	11.277
144	TRUE	Empty	Actin-related protein 2/3 complex subunit 5 OS=Homo sapiens GN=ARPC5 PE=1 SV=3	ARPC5_HUMAN	?	TRUE	.936048318	1	0	6.259	7.97	3.61	4.57	5.87
145	TRUE	Empty	Actin-related protein 2/3 complex subunit 5-like protein OS=Homo sapiens GN=ARPC5L PE=1 SV=1	ARP5L_HUMAN	17 kDa	TRUE	.598991574	0.6	0	2.0863	5.47	1.04	1.43	1.96
146	TRUE	Empty	Actin-related protein 3 OS=Homo sapiens GN=ACTR3 PE=1 SV=3	ARP3_HUMAN	47 kDa	TRUE	.392274922	1.2	30.944	15.648	26.931	35.77	26.261	27.254
147	TRUE	Empty	Activated RNA polymerase II transcriptional coactivator p15 OS=Homo sapiens GN=SUB1 PE=1 SV=3	TCP4_HUMAN	14 kDa		0.483305995	0.7	4.06	23.993	13.964	5.17	9.85	13.157
148	TRUE	Empty	Activator of 90 kDa heat shock protein ATPase homolog 1 OS=Homo sapiens GN=AHSA1 PE=1 SV=1	AHSA1_HUMAN	?		0.09127636	2.4	2.03	0	0.99746	2.57	2.14	2.94
149	TRUE	Empty	Activity-dependent neuroprotector homeobox protein OS=Homo sapiens GN=ADNP PE=1 SV=1	ADNP_HUMAN	124 kDa	TRUE	.002694299	0.18639875	8.13	8.54	5.47	0.74522	1.43	1.96
150	TRUE	Empty	Acyl carrier protein, mitochondrial OS=Homo sapiens GN=NDUFAB1 PE=1 SV=3	ACPM_HUMAN	17 kDa		0.236841901	INF	0	0	0	3.61	0	0.93978
151	TRUE	Empty	Acylamino-acid-releasing enzyme OS=Homo sapiens GN=APEH PE=1 SV=4	ACPH_HUMAN	81 kDa		0.543212036	0.6	0	14.604	7.97	5.17	3.85	4.89
152	TRUE	Empty	Acyl-CoA dehydrogenase family member 9, mitochondrial OS=Homo sapiens GN=ACAD9 PE=1 SV=1	ACAD9_HUMAN	69 kDa		0.019847808	4.183058422	0	1.0432	0.99746	2.57	2.14	3.91
153	TRUE	Empty	Acyl-CoA-binding protein OS=Homo sapiens GN=DBI PE=1 SV=2	ACBP_HUMAN	?		0.758037229	0.8	0	12.518	13.964	6.707	11.86	2.94

154	TRUE	Empty	Acyl-coenzyme A thioesterase 9, mitochondrial OS=Homo sapiens GN=ACOT9 PE=1 SV=2	ACOT9_HUMAN	?		0.840559453	1.3	0	2.0863	0	0	1.43	0.93978
155	TRUE	Empty	Acylphosphatase-1 OS=Homo sapiens GN=ACYP1 PE=1 SV=2	ACYP1_HUMAN	?		0.199673922	0.2	0	2.0863	1.49	0.74522	0	0
156	TRUE	Empty	Acyl-protein thioesterase 1 OS=Homo sapiens GN=LYPLA1 PE=1 SV=1	LYPA1_HUMAN	?		0.884130871	0.9	0	9.86	2.24	2.57	5.99	2.94
157	TRUE	Empty	Adapter molecule crk OS=Homo sapiens GN=CRK PE=1 SV=2	CRK_HUMAN	?	TRUE	.409086063	2.6	0	0	0.99746	0	1.43	0.93978
158	TRUE	Empty	Adenine phosphoribosyltransferase OS=Homo sapiens GN=APRT PE=1 SV=2	APT_HUMAN	?		0.887260184	1	19.893	20.863	23.939	24.592	22.025	16.916
159	TRUE	Empty	Adenosine kinase OS=Homo sapiens GN=ADK PE=1 SV=2	ADK_HUMAN	?		0.139868417	2.4	0	10.432	6.22	9.78	11.86	19.735
160	TRUE	Empty	Adenosylhomocysteinase 2 OS=Homo sapiens GN=AHCYL1 PE=1 SV=2	SAHH2_HUMAN	?	TRUE	.329975308	6.2	2.03	0	0	11.178	2.14	0
161	TRUE	Empty	Adenosylhomocysteinase OS=Homo sapiens GN=AHCY PE=1 SV=4	SAHH_HUMAN	?		0.002600651	1.883231999	15.472	11.475	17.954	28.318	27.108	29.133
162	TRUE	Empty	Adenylate kinase 2, mitochondrial OS=Homo sapiens GN=AK2 PE=1 SV=2	KAD2_HUMAN	?		0.337523635	0.7	6.631	12.518	13.964	4.13	7.42	11.277
163	TRUE	Empty	Adenylate kinase isoenzyme 1 OS=Homo sapiens GN=AK1 PE=1 SV=3	KAD1_HUMAN	22 kDa	TRUE	.602051864	1.5	0	9.86	2.24	3.61	4.57	10.338
164	TRUE	Empty	Adenylosuccinate lyase OS=Homo sapiens GN=ADSL PE=1 SV=2	PUR8_HUMAN	?	TRUE	.136122783	INF	0	0	0	2.09	0	1.96
165	TRUE	Empty	Adenylyl cyclase-associated protein 1 OS=Homo sapiens GN=CAP1 PE=1 SV=5	CAP1_HUMAN	?	TRUE	.019069428	2.151224572	4.06	5.59	2.24	11.178	8.13	7.83
166	TRUE	Empty	Adipocyte plasma membrane-associated protein OS=Homo sapiens GN=APMAP PE=1 SV=2	APMAP_HUMAN	?		0.669298398	1.6	8.13	0	0	2.09	1.43	9.78
167	TRUE	Empty	ADP/ATP translocase 2 OS=Homo sapiens GN=SLC25A5 PE=1 SV=7	ADT2_HUMAN	33 kDa	TRUE	.801185623	1.3	112.73	2.0863	2.24	40.242	47.44	60.146
168	TRUE	Empty	ADP/ATP translocase 3 OS=Homo sapiens GN=SLC25A6 PE=1 SV=4	ADT3_HUMAN	33 kDa	TRUE	.681630302	1.4	75.151	2.0863	0	29.809	35.58	45.11
169	TRUE	Empty	ADP-ribosylation factor 1 OS=Homo sapiens GN=ARF1 PE=1 SV=2	ARF1_HUMAN	21 kDa	TRUE	.026667737	1.499405403	24.314	29.209	17.954	34.28	38.121	34.772
170	TRUE	Empty	ADP-ribosylation factor 4 OS=Homo sapiens GN=ARF4 PE=1 SV=3	ARF4_HUMAN	21 kDa	TRUE	.851401055	0.9	8.13	19.82	8.71	13.414	7.42	14.097
171	TRUE	Empty	ADP-ribosylation factor 6 OS=Homo sapiens GN=ARF6 PE=1 SV=2	ARF6_HUMAN	20 kDa	TRUE	.262949104	3.9	0	0	0.99746	2.57	1.43	0
172	TRUE	Empty	ADP-ribosylation factor-like protein 1 OS=Homo sapiens GN=ARL1 PE=1 SV=1	ARL1_HUMAN	?		0.023730014	6.571491589	0	0	0.99746	2.09	1.43	1.96
173	TRUE	Empty	ADP-ribosylation factor-like protein 3 OS=Homo sapiens GN=ARL3 PE=1 SV=2	ARL3_HUMAN	20 kDa		0.24827618	0.3	0	9.86	7.97	1.04	3.85	0
174	TRUE	Empty	ADP-sugar pyrophosphatase OS=Homo sapiens GN=NUDT5 PE=1 SV=1	NUDT5_HUMAN	24 kDa		0.760233275	1.2	0	4.1727	4.73	4.13	2.14	3.91
175	TRUE	Empty	Aflatoxin B1 aldehyde reductase member 2 OS=Homo sapiens GN=AKR7A2 PE=1 SV=3	ARK72_HUMAN	40 kDa	TRUE	.936285911	1	6.631	15.648	7.97	13.414	8.13	7.83
176	TRUE	Empty	AGO3_HUMAN-DECOY	AGO3_HUMAN-DEC	?	TRUE	.195474009	2.1	0	1.0432	0.99746	0.74522	1.43	1.96
177	TRUE	Empty	AH receptor-interacting protein OS=Homo sapiens GN=AIP PE=1 SV=2	AIP_HUMAN	38 kDa		0.129938577	2.9	0	1.0432	0.99746	2.57	0.84713	2.94
178	TRUE	Empty	A-kinase anchor protein 1, mitochondrial OS=Homo sapiens GN=AKAP1 PE=1 SV=1	AKAP1_HUMAN	?		0.03308882	3.48079121	0	0.99746	0	0.74522	0.84713	1.96
179	TRUE	Empty	A-kinase anchor protein 8 OS=Homo sapiens GN=AKAP8 PE=1 SV=1	AKAP8_HUMAN	76 kDa	TRUE	.185911451	4.6	0	1.0432	0	2.09	0.84713	0.93978
180	TRUE	Empty	A-kinase anchor protein 9 OS=Homo sapiens GN=AKAP9 PE=1 SV=3	AKAP9_HUMAN	?	TRUE	.136121777	INF	0	0	0	1.04	0	0.93978

181	TRUE	Empty	Alanine--tRNA ligase, cytoplasmic OS=Homo sapiens GN=AARS PE=1 SV=2	SYAC_HUMAN	?	TRUE	.015484022	2.17027666	11.052	8.54	10.972	20.866	17.79	27.254
182	TRUE	Empty	Alcohol dehydrogenase [NADP(+)] OS=Homo sapiens GN=AKR1A1 PE=1 SV=3	AK1A1_HUMAN	37 kDa		0.188122049	3	0	4.1727	1.49	10.433	5.0828	2.94
183	TRUE	Empty	Alcohol dehydrogenase class-3 OS=Homo sapiens GN=ADH5 PE=1 SV=4	ADHX_HUMAN	40 kDa	TRUE	0.00485573	6.868646362	0	0	0.99746	1.04	2.14	2.94
184	TRUE	Empty	Aldehyde dehydrogenase family 16 member A1 OS=Homo sapiens GN=ALDH16A1 PE=1 SV=2	A16A1_HUMAN	?	TRUE	.021107574	4.040099178	2.03	1.0432	0.99746	3.61	5.99	7.83
185	TRUE	Empty	Aldehyde dehydrogenase X, mitochondrial OS=Homo sapiens GN=ALDH1B1 PE=1 SV=3	AL1B1_HUMAN	57 kDa	TRUE	0.00734386	8.16784633	0	0	0.99746	3.61	2.14	1.96
186	TRUE	Empty	Aldo-keto reductase family 1 member C1 OS=Homo sapiens GN=AKR1C1 PE=1 SV=1	AK1C1_HUMAN	37 kDa	TRUE	.443867849	1.7	0	9.86	2.24	4.13	5.99	10.338
187	TRUE	Empty	Allograft inflammatory factor 1-like OS=Homo sapiens GN=AIF1L PE=1 SV=1	AIF1L_HUMAN	?		0.373900966	0	0	0	3.98	0	0	0
188	TRUE	Empty	Alpha-2-macroglobulin OS=Homo sapiens GN=A2M PE=1 SV=3	A2MG_HUMAN	163 kDa	TRUE	.856753163	1.2	0	2.0863	11.969	2.57	1.43	13.157
189	TRUE	Empty	Alpha-2-macroglobulin receptor-associated protein OS=Homo sapiens GN=LRPAP1 PE=1 SV=1	AMRP_HUMAN	41 kDa		0.118047874	INF	0	0	0	5.65	1.43	0.93978
190	TRUE	Empty	Alpha-actinin-1 OS=Homo sapiens GN=ACTN1 PE=1 SV=2	ACTN1_HUMAN	?	TRUE	.171008554	0.8	97.254	132.48	140.64	84.21	114.36	91.159
191	TRUE	Empty	Alpha-actinin-4 OS=Homo sapiens GN=ACTN4 PE=1 SV=2	ACTN4_HUMAN	?	TRUE	.244631272	0.8	130.41	218.02	236.4	139.36	164.34	143.79
192	TRUE	Empty	Alpha-aminoadipic semialdehyde dehydrogenase OS=Homo sapiens GN=ALDH7A1 PE=1 SV=5	AL7A1_HUMAN	?		0.005098322	7.635977017	2.03	0	0	5.65	5.0828	6.85
193	TRUE	Empty	Alpha-endosulfine OS=Homo sapiens GN=ENSA PE=1 SV=1	ENSA_HUMAN	?	TRUE	.262180901	0.3	0	9.86	6.22	0.74522	2.14	1.96
194	TRUE	Empty	Alpha-enolase OS=Homo sapiens GN=ENO1 PE=1 SV=2	ENOA_HUMAN	?	TRUE	0.99758219	1	161.35	433.96	358.09	210.9	316.83	426.66
195	TRUE	Empty	Alpha-galactosidase A OS=Homo sapiens GN=GLA PE=1 SV=1	AGAL_HUMAN	49 kDa		0.766585891	1.2	0	10.432	5.47	5.65	11.013	3.91
196	TRUE	Empty	Alpha-N-acetylglucosaminidase OS=Homo sapiens GN=NAGLU PE=1 SV=2	ANAG_HUMAN	82 kDa		0.797694301	0.8	0	3.1295	1.49	0.74522	1.43	1.96
197	TRUE	Empty	Alpha-soluble NSF attachment protein OS=Homo sapiens GN=NAPA PE=1 SV=3	SNAA_HUMAN	33 kDa	TRUE	.015689374	5.687621439	4.06	5.59	1.49	14.904	21.178	30.073
198	TRUE	Empty	Alpha-taxilin OS=Homo sapiens GN=TXLNA PE=1 SV=3	TXLNA_HUMAN	62 kDa	TRUE	.121394667	INF	0	0	0	1.04	0	1.96
199	TRUE	Empty	Aminoacyl tRNA synthase complex-interacting multifunctional protein 1 OS=Homo sapiens GN=AIMP1 PE=1 SV=2	AIMP1_HUMAN	?		0.185495762	5.7	0	0	1.49	2.57	1.43	7.83
200	TRUE	Empty	Aminoacyl tRNA synthase complex-interacting multifunctional protein 2 OS=Homo sapiens GN=AIMP2 PE=1 SV=2	AIMP2_HUMAN	35 kDa		0.590356854	1.7	6.631	1.0432	0	2.57	1.43	9.78
201	TRUE	Empty	Aminopeptidase B OS=Homo sapiens GN=RNPEP PE=1 SV=2	AMPB_HUMAN	73 kDa		0.119448566	INF	0	0	0	0	3.85	2.94
202	TRUE	Empty	Angio-associated migratory cell protein OS=Homo sapiens GN=AAMP PE=1 SV=2	AAMP_HUMAN	47 kDa		0.03048408	9.513707822	0	1.0432	0	4.13	1.43	3.91
203	TRUE	Empty	Ankyrin repeat domain-containing protein 17 OS=Homo sapiens GN=ANKRD17 PE=1 SV=3	ANR17_HUMAN	?	TRUE	.840559453	1.3	0	2.0863	0	0	1.43	0.93978
204	TRUE	Empty	Ankyrin-1 OS=Homo sapiens GN=ANK1 PE=1 SV=3	ANK1_HUMAN	?	TRUE	.373900966	0	0	0	8.71	0	0	0
205	TRUE	Empty	Ankyrin-3 OS=Homo sapiens GN=ANK3 PE=1 SV=3	ANK3_HUMAN	?	TRUE	.515839158	0.5	0	2.0863	6.22	0.74522	3.85	0
206	TRUE	Empty	Annexin A1 OS=Homo sapiens GN=ANXA1 PE=1 SV=2	ANXA1_HUMAN	39 kDa	TRUE	0.33251746	1.3	11.052	13.561	9.46	14.159	20.331	10.338
207	TRUE	Empty	Annexin A11 OS=Homo sapiens GN=ANXA11 PE=1 SV=1	ANX11_HUMAN	?	TRUE	0.05337963	2.9	2.03	2.0863	0.99746	6.707	5.99	2.94
208	TRUE	Empty	Annexin A2 OS=Homo sapiens GN=ANXA2 PE=1 SV=2	ANXA2_HUMAN	?	TRUE	.904484993	0.9	172.4	49.029	50.87	87.936	90.643	78.002
209	TRUE	Empty	Annexin A3 OS=Homo sapiens GN=ANXA3 PE=1 SV=3	ANXA3_HUMAN	36 kDa	TRUE	.379559643	2.6	0	2.0863	0	2.09	2.14	0
210	TRUE	Empty	Annexin A4 OS=Homo sapiens GN=ANXA4 PE=1 SV=4	ANXA4_HUMAN	?	TRUE	.014494473	2.777936447	4.06	1.0432	3.98	7.22	8.13	10.338
211	TRUE	Empty	Annexin A5 OS=Homo sapiens GN=ANXA5 PE=1 SV=2	ANXA5_HUMAN	36 kDa		0.039563618	2.603960706	22.103	6.259	9.46	24.592	33.885	41.35
212	TRUE	Empty	Annexin A7 OS=Homo sapiens GN=ANXA7 PE=1 SV=3	ANXA7_HUMAN	?	TRUE	0.000368	13.54219718	0	0.99746	0	3.61	5.0828	4.89

213	TRUE	Empty	Anterior gradient protein 2 homolog OS=Homo sapiens GN=AGR2 PE=1 SV=1	AGR2_HUMAN	20 kDa	TRUE	.009509825	0.427507102	79.571	62.59	52.865	24.592	29.65	29.133
214	TRUE	Empty	Antithrombin-III OS=Homo sapiens GN=SERPINC1 PE=1 SV=1	ANT3_HUMAN	53 kDa		0.781341676	0.7	0	0	4.73	0.74522	0.84713	1.96
215	TRUE	Empty	AP-1 complex subunit beta-1 OS=Homo sapiens GN=AP1B1 PE=1 SV=2	AP1B1_HUMAN	?	TRUE	.050255883	2.8	6.631	8.54	21.944	23.847	33.885	46.049
216	TRUE	Empty	AP-1 complex subunit gamma-1 OS=Homo sapiens GN=AP1G1 PE=1 SV=5	AP1G1_HUMAN	?	TRUE	.002606621	21.79365589	0.99746	0	0	8.74	5.0828	8.81
217	TRUE	Empty	AP-1 complex subunit mu-2 OS=Homo sapiens GN=AP1M2 PE=1 SV=4	AP1M2_HUMAN	?	TRUE	.173447135	2.3	2.03	1.0432	4.73	2.09	6.71	9.78
218	TRUE	Empty	AP-2 complex subunit alpha-1 OS=Homo sapiens GN=AP2A1 PE=1 SV=3	AP2A1_HUMAN	?	TRUE	.333638339	2.1	0	0	9.46	5.17	6.71	8.81
219	TRUE	Empty	AP-2 complex subunit alpha-2 OS=Homo sapiens GN=AP2A2 PE=1 SV=2	AP2A2_HUMAN	?	TRUE	.174676845	3.3	0	1.0432	1.49	2.57	5.99	1.96
220	TRUE	Empty	AP-2 complex subunit beta OS=Homo sapiens GN=AP2B1 PE=1 SV=1	AP2B1_HUMAN	?	TRUE	.098576866	2	4.06	7.22	15.959	13.414	20.331	21.615
221	TRUE	Empty	AP-2 complex subunit mu OS=Homo sapiens GN=AP2M1 PE=1 SV=2	AP2M1_HUMAN	?		0.600971946	1.5	2.03	0	4.73	5.17	1.43	2.94
222	TRUE	Empty	AP-3 complex subunit beta-1 OS=Homo sapiens GN=AP3B1 PE=1 SV=3	AP3B1_HUMAN	?	TRUE	.041852932	14.87458144	0	0	0.99746	2.57	5.0828	7.83
223	TRUE	Empty	AP-3 complex subunit delta-1 OS=Homo sapiens GN=AP3D1 PE=1 SV=1	AP3D1_HUMAN	?	TRUE	.001790582	12.39558479	0	0	0.99746	5.65	3.85	3.91
224	TRUE	Empty	AP-3 complex subunit mu-1 OS=Homo sapiens GN=AP3M1 PE=1 SV=1	AP3M1_HUMAN	47 kDa	TRUE	.373900966	INF	0	0	0	0	0	1.96
225	TRUE	Empty	Apoptosis inhibitor 5 OS=Homo sapiens GN=API5 PE=1 SV=3	API5_HUMAN	?		0.403926404	2	0	0	2.24	1.04	2.14	1.96
226	TRUE	Empty	Apoptosis regulator BAX OS=Homo sapiens GN=BAX PE=1 SV=1	BAX_HUMAN	?		0.996953168	1	4.06	1.0432	2.24	2.57	3.85	2.94
227	TRUE	Empty	Apoptosis-associated speck-like protein containing a CARD OS=Homo sapiens GN=PYCARD PE=1 SV=2	ASC_HUMAN	?		0.13736875	1.4	4.06	6.259	7.97	8.74	10.166	7.83
228	TRUE	Empty	Apoptosis-inducing factor 1, mitochondrial OS=Homo sapiens GN=AIFM1 PE=1 SV=1	AIFM1_HUMAN	?		0.047075874	5.735665981	0	2.0863	1.49	11.178	8.13	3.91
229	TRUE	Empty	Apoptotic chromatin condensation inducer in the nucleus OS=Homo sapiens GN=ACIN1 PE=1 SV=2	ACINU_HUMAN	?	TRUE	.576584151	0.5	0	1.0432	9.46	1.04	0.84713	2.94
230	TRUE	Empty	Arf-GAP domain and FG repeat-containing protein 1 OS=Homo sapiens GN=AGFG1 PE=1 SV=2	AGFG1_HUMAN	?	TRUE	.373900966	INF	0	0	0	1.04	0	0
231	TRUE	Empty	Arginine--tRNA ligase, cytoplasmic OS=Homo sapiens GN=RARS PE=1 SV=2	SYRC_HUMAN	?	TRUE	.000718645	8.79672299	2.03	2.0863	0	11.178	14.401	12.217
232	TRUE	Empty	ARI1_HUMAN-DECOY	ARI1_HUMAN-DECC	?		0.153594946	0	0	2.0863	3.98	0	0	0
233	TRUE	Empty	Asparagine synthetase [glutamine-hydrolyzing] OS=Homo sapiens GN=ASNS PE=1 SV=4	ASNS_HUMAN	?		0.202194141	3.3	4.06	0	0	7.22	5.0828	1.96
234	TRUE	Empty	Asparagine--tRNA ligase, cytoplasmic OS=Homo sapiens GN=NARS PE=1 SV=1	SYNC_HUMAN	?	TRUE	.033813504	29.25738113	0	1.0432	0	4.13	11.013	15.037
235	TRUE	Empty	Aspartate aminotransferase, cytoplasmic OS=Homo sapiens GN=GOT1 PE=1 SV=3	AATC_HUMAN	?	TRUE	.240827197	0.5	4.06	18.777	16.957	6.707	9.85	4.89
236	TRUE	Empty	Aspartate aminotransferase, mitochondrial OS=Homo sapiens GN=GOT2 PE=1 SV=3	AATM_HUMAN	?	TRUE	.670394191	1.1	6.631	11.475	10.972	12.669	6.71	13.157
237	TRUE	Empty	Aspartate--tRNA ligase, cytoplasmic OS=Homo sapiens GN=DARS PE=1 SV=2	SYDC_HUMAN	?		0.016663436	12.34699603	2.03	0	0.99746	17.885	7.42	14.097
238	TRUE	Empty	Aspartyl aminopeptidase OS=Homo sapiens GN=DNPEP PE=1 SV=1	DNPEP_HUMAN	52 kDa		0.977954822	1	4.06	5.59	5.47	5.17	7.42	1.96
239	TRUE	Empty	Ataxin-2-like protein OS=Homo sapiens GN=ATXN2L PE=1 SV=2	ATX2L_HUMAN	?		0.836436044	0.8	0	4.1727	5.47	5.17	1.43	0.93978

240	TRUE	Empty	ATP synthase F(0) complex subunit B1, mitochondrial OS=Homo sapiens GN=ATP5F1 PE=1 SV=2	AT5F1_HUMAN	29 kDa		0.344258503	2.1	4.06	0	0	2.09	3.85	2.94
241	TRUE	Empty	ATP synthase subunit alpha, mitochondrial OS=Homo sapiens GN=ATP5A1 PE=1 SV=1	ATPA_HUMAN	?		0.592881441	1.4	53.048	8.54	9.46	40.987	29.65	27.254
242	TRUE	Empty	ATP synthase subunit beta, mitochondrial OS=Homo sapiens GN=ATP5B PE=1 SV=3	ATPB_HUMAN	57 kDa		0.972014445	1	145.88	54.245	33.914	64.089	88.949	77.062
243	TRUE	Empty	ATP synthase subunit d, mitochondrial OS=Homo sapiens GN=ATP5H PE=1 SV=3	ATP5H_HUMAN	?	TRUE	.211638856	INF	0	0	0	2.09	0	0.93978
244	TRUE	Empty	ATP synthase subunit delta, mitochondrial OS=Homo sapiens GN=ATP5D PE=1 SV=2	ATPD_HUMAN	17 kDa		0.964742757	1	8.13	1.0432	0	2.57	2.14	4.89
245	TRUE	Empty	ATP synthase subunit e, mitochondrial OS=Homo sapiens GN=ATP5I PE=1 SV=2	ATP5I_HUMAN	8 kDa		0.810945021	0.9	6.631	5.59	0	2.09	2.14	4.89
246	TRUE	Empty	ATP synthase subunit f, mitochondrial OS=Homo sapiens GN=ATP5J2 PE=1 SV=3	ATPK_HUMAN	?		0.23631817	2.6	6.631	0	0	3.61	7.42	5.87
247	TRUE	Empty	ATP synthase subunit gamma, mitochondrial OS=Homo sapiens GN=ATP5C1 PE=1 SV=1	ATPG_HUMAN	?	TRUE	.412671593	1.6	8.13	1.0432	1.49	6.707	4.57	8.81
248	TRUE	Empty	ATP synthase subunit O, mitochondrial OS=Homo sapiens GN=ATP5O PE=1 SV=1	ATPO_HUMAN	23 kDa	TRUE	.575834535	1.4	0	16.691	10.972	11.178	16.943	9.78
249	TRUE	Empty	ATPase ASNA1 OS=Homo sapiens GN=ASNA1 PE=1 SV=2	ASNA_HUMAN	39 kDa		0.08219335	2.9	2.03	1.0432	0	2.57	4.57	2.94
250	TRUE	Empty	ATPase WRNIP1 OS=Homo sapiens GN=WRNIP1 PE=1 SV=2	WRIP1_HUMAN	?	TRUE	.373900966	INF	0	0	0	0	1.43	0
251	TRUE	Empty	ATP-binding cassette sub-family E member 1 OS=Homo sapiens GN=ABCE1 PE=1 SV=1	ABCE1_HUMAN	67 kDa		0.061930926	10	0	0	0.99746	2.09	1.43	5.87
252	TRUE	Empty	ATP-binding cassette sub-family F member 1 OS=Homo sapiens GN=ABCF1 PE=1 SV=2	ABCF1_HUMAN	?	TRUE	.432550548	2	0	0	2.24	0.74522	2.14	2.94
253	TRUE	Empty	ATP-binding cassette sub-family F member 2 OS=Homo sapiens GN=ABCF2 PE=1 SV=2	ABCF2_HUMAN	?	TRUE	.184606477	INF	0	0	0	0.74522	0	1.96
254	TRUE	Empty	ATP-binding cassette sub-family F member 3 OS=Homo sapiens GN=ABCF3 PE=1 SV=2	ABCF3_HUMAN	?		0.005539052	6.571491589	0.99746	0	0	2.09	1.43	1.96
255	TRUE	Empty	ATP-citrate synthase OS=Homo sapiens GN=ACLY PE=1 SV=3	ACLY_HUMAN	?	TRUE	.006589573	8.30722469	4.06	0	1.49	23.102	16.096	14.097
256	TRUE	Empty	ATP-dependent 6-phosphofructokinase, liver type OS=Homo sapiens GN=PFKL PE=1 SV=6	PFKAL_HUMAN	?	TRUE	.076190979	1.9	19.893	11.475	5.47	18.63	27.108	26.314
257	TRUE	Empty	ATP-dependent 6-phosphofructokinase, muscle type OS=Homo sapiens GN=PFKM PE=1 SV=2	PFKAM_HUMAN	?	TRUE	.356080741	2.2	6.631	0	0	2.09	4.57	7.83
258	TRUE	Empty	ATP-dependent 6-phosphofructokinase, platelet type OS=Homo sapiens GN=PFKP PE=1 SV=2	PFKAP_HUMAN	?	TRUE	.175380355	1.7	24.314	6.259	8.71	23.847	22.025	21.615
259	TRUE	Empty	ATP-dependent RNA helicase A OS=Homo sapiens GN=DHX9 PE=1 SV=4	DHX9_HUMAN	?	TRUE	.015840947	2.326152578	24.314	7.22	14.962	38.751	33.885	35.712
260	TRUE	Empty	ATP-dependent RNA helicase DDX1 OS=Homo sapiens GN=DDX1 PE=1 SV=2	DDX1_HUMAN	?		0.205597599	1.5	8.13	14.604	13.964	11.923	26.261	19.735
261	TRUE	Empty	ATP-dependent RNA helicase DDX18 OS=Homo sapiens GN=DDX18 PE=1 SV=2	DDX18_HUMAN	75 kDa	TRUE	.891581636	1.1	4.06	0	0	1.04	1.43	1.96
262	TRUE	Empty	ATP-dependent RNA helicase DDX19A OS=Homo sapiens GN=DDX19A PE=1 SV=1	DD19A_HUMAN	?	TRUE	.005538615	3.285655565	0.99746	0	0	1.04	0.84713	0.93978
263	TRUE	Empty	ATP-dependent RNA helicase DDX39A OS=Homo sapiens GN=DDX39A PE=1 SV=2	DX39A_HUMAN	?	TRUE	.000546845	2.05379188	8.13	8.54	8.71	16.395	19.484	17.856
264	TRUE	Empty	ATP-dependent RNA helicase DDX3X OS=Homo sapiens GN=DDX3X PE=1 SV=3	DDX3X_HUMAN	?	TRUE	.281507001	1.3	33.155	16.691	19.949	29.809	29.65	29.133
265	TRUE	Empty	ATP-dependent RNA helicase DDX42 OS=Homo sapiens GN=DDX42 PE=1 SV=1	DDX42_HUMAN	?	TRUE	.035155885	4.555065417	0	0	1.49	3.61	2.14	2.94
266	TRUE	Empty	BAG family molecular chaperone regulator 3 OS=Homo sapiens GN=BAG3 PE=1 SV=3	BAG3_HUMAN	62 kDa		0.872365376	0.9	0	9.86	3.98	3.61	2.14	5.87

267	TRUE	Empty	Band 3 anion transport protein OS=Homo sapiens GN=SLC4A1 PE=1 SV=3	B3AT_HUMAN	?		0.373900966	0	0	0	2.24	0	0	0
268	TRUE	Empty	Band 4.1-like protein 4B OS=Homo sapiens GN=EPB41L4B PE=2 SV=2	E41LB_HUMAN	?	TRUE	.217218164	INF	0	0	0	0	0.84713	2.94
269	TRUE	Empty	Barrier-to-autointegration factor OS=Homo sapiens GN=BANF1 PE=1 SV=1	BAF_HUMAN	10 kDa		0.337061464	0.8	8.13	18.777	13.964	8.74	11.013	12.217
270	TRUE	Empty	Basic leucine zipper and W2 domain-containing protein 1 OS=Homo sapiens GN=BZW1 PE=1 SV=1	BZW1_HUMAN	?	TRUE	.094658247	5.5	2.03	0	0	1.04	5.99	4.89
271	TRUE	Empty	Basic leucine zipper and W2 domain-containing protein 2 OS=Homo sapiens GN=BZW2 PE=1 SV=1	BZW2_HUMAN	?	TRUE	.169754044	INF	0	0	0	0	1.43	3.91
272	TRUE	Empty	B-cell CLL/lymphoma 9-like protein OS=Homo sapiens GN=BCL9L PE=1 SV=1	BCL9L_HUMAN	?		0.184606477	INF	0	0	0	0.74522	0	1.96
273	TRUE	Empty	B-cell receptor-associated protein 31 OS=Homo sapiens GN=BCAP31 PE=1 SV=3	BAP31_HUMAN	?		0.613977735	0.4	28.734	0	0	6.707	3.85	2.94
274	TRUE	Empty	Bcl-2-associated transcription factor 1 OS=Homo sapiens GN=BCLAF1 PE=1 SV=2	BCLF1_HUMAN	?	TRUE	0.30647747	0.1	0	1.0432	3.98	0.74522	0	0
275	TRUE	Empty	Beta-actin-like protein 2 OS=Homo sapiens GN=ACTBL2 PE=1 SV=2	ACTBL_HUMAN	42 kDa	TRUE	.144748861	0.7	81.782	53.202	82.789	44.713	60.994	53.568
276	TRUE	Empty	Beta-arrestin-1 OS=Homo sapiens GN=ARRB1 PE=1 SV=2	ARRB1_HUMAN	?	TRUE	.053421057	INF	0	0	0	2.57	0.84713	3.91
277	TRUE	Empty	Beta-galactosidase OS=Homo sapiens GN=GLB1 PE=1 SV=2	BGAL_HUMAN	?	TRUE	.195474009	2.1	0	1.0432	0.99746	0.74522	1.43	1.96
278	TRUE	Empty	Beta-hexosaminidase subunit alpha OS=Homo sapiens GN=HEXA PE=1 SV=2	HEXA_HUMAN	?	TRUE	.920175008	0.9	0	6.259	4.73	5.65	2.14	2.94
279	TRUE	Empty	Beta-hexosaminidase subunit beta OS=Homo sapiens GN=HEXB PE=1 SV=3	HEXB_HUMAN	63 kDa		0.052661927	0.3	2.03	1.0432	1.49	0.74522	0.84713	0
280	TRUE	Empty	Beta-lactamase-like protein 2 OS=Homo sapiens GN=LACTB2 PE=1 SV=2	LACB2_HUMAN	33 kDa		0.473019919	3.8	0	0	2.24	10.433	0.84713	0
281	TRUE	Empty	BH3-interacting domain death agonist OS=Homo sapiens GN=BID PE=1 SV=1	BID_HUMAN	?		0.401696177	0.6	11.052	14.604	1.49	6.707	1.43	7.83
282	TRUE	Empty	Bifunctional 3'-phosphoadenosine 5'-phosphosulfate synthase 1 OS=Homo sapiens GN=PAPSS1 PE=1 SV=2	PAPS1_HUMAN	71 kDa	TRUE	.074620032	2.6	2.03	0	1.49	3.61	2.14	4.89
283	TRUE	Empty	Bifunctional 3'-phosphoadenosine 5'-phosphosulfate synthase 2 OS=Homo sapiens GN=PAPSS2 PE=1 SV=2	PAPS2_HUMAN	?	TRUE	.045552312	2.427306816	2.03	6.259	10.972	12.669	19.484	15.037
284	TRUE	Empty	Bifunctional coenzyme A synthase OS=Homo sapiens GN=COASY PE=1 SV=4	COASY_HUMAN	?		0.03308882	3.48079121	0	0.99746	0	0.74522	0.84713	1.96
285	TRUE	Empty	Bifunctional glutamate/proline--tRNA ligase OS=Homo sapiens GN=EPRS PE=1 SV=5	SYEP_HUMAN	171 kDa	TRUE	)000762161	28.37417039	0	0.99746	0	10.433	8.13	9.78
286	TRUE	Empty	Bifunctional methylenetetrahydrofolate dehydrogenase/cyclohydrolase, mitochondrial OS=Homo sapiens GN=MTHFD2 PE=1 SV=2	MTDC_HUMAN	?	TRUE	.117744445	INF	0	0	0	1.04	1.43	0
287	TRUE	Empty	Bifunctional purine biosynthesis protein PURH OS=Homo sapiens GN=ATIC PE=1 SV=3	PUR9_HUMAN	?	TRUE	.252948543	1.3	46.417	26.079	35.908	41.732	41.51	53.568
288	TRUE	Empty	Biliverdin reductase A OS=Homo sapiens GN=BLVRA PE=1 SV=2	BIEA_HUMAN	33 kDa	TRUE	.005206173	4.654333844	0	4.1727	3.98	10.433	14.401	13.157
289	TRUE	Empty	BolA-like protein 2 OS=Homo sapiens GN=BOLA2 PE=1 SV=1	BOLA2_HUMAN	?		0.780084979	0.8	0	5.59	2.24	1.04	3.85	1.96
290	TRUE	Empty	Brain acid soluble protein 1 OS=Homo sapiens GN=BASP1 PE=1 SV=2	BASP1_HUMAN	?		0.17460843	0	2.03	1.0432	8.71	0	0	0
291	TRUE	Empty	Branched-chain-amino-acid aminotransferase, mitochondrial OS=Homo sapiens GN=BCAT2 PE=1 SV=2	BCAT2_HUMAN	?		0.331587669	0.4	0	7.22	6.22	0.74522	2.14	2.94
292	TRUE	Empty	BRCA1-A complex subunit BRE OS=Homo sapiens GN=BRE PE=1 SV=2	BRE_HUMAN	?		0.513864492	2.1	0	1.0432	0.99746	0	3.85	0.93978
293	TRUE	Empty	BRCA1-associated ATM activator 1 OS=Homo sapiens GN=BRAT1 PE=1 SV=2	BRAT1_HUMAN	?		0.063218814	INF	0	0	0	2.09	0.84713	4.89

294	TRUE	Empty	BRCA1-associated protein OS=Homo sapiens GN=BRAP PE=1 SV=2	BRAP_HUMAN	?		0.005538615	3.285655565	0.99746	0	0	1.04	0.84713	0.93978
295	TRUE	Empty	Breast carcinoma-amplified sequence 1 OS=Homo sapiens GN=BCAS1 PE=1 SV=2	BCAS1_HUMAN	?	TRUE	.394250017	0.5	0	7.22	4.73	2.57	0.84713	2.94
296	TRUE	Empty	BRO1 domain-containing protein BROX OS=Homo sapiens GN=BROX PE=1 SV=1	BROX_HUMAN	?		0.053421057	INF	0	0	0	2.57	0.84713	3.91
297	TRUE	Empty	C-1-tetrahydrofolate synthase, cytoplasmic OS=Homo sapiens GN=MTHFD1 PE=1 SV=3	C1TC_HUMAN	102 kDa		0.041818893	2.22074096	35.365	10.432	14.962	41.732	41.51	51.688
298	TRUE	Empty	Cactin OS=Homo sapiens GN=CACTIN PE=1 SV=3	CATIN_HUMAN	?		0.351869907	1.7	0	4.1727	3.98	6.707	4.57	2.94
299	TRUE	Empty	CAD protein OS=Homo sapiens GN=CAD PE=1 SV=3	PYR1_HUMAN	243 kDa	TRUE	.002719629	21.71936719	0.99746	0	0	6.707	9.85	5.87
300	TRUE	Empty	Cadherin EGF LAG seven-pass G-type receptor 2 OS=Homo sapiens GN=CELSR2 PE=1 SV=1	CELR2_HUMAN	317 kDa	TRUE	.163163057	0	0	6.259	2.24	0	0	0
301	TRUE	Empty	Cadherin-1 OS=Homo sapiens GN=CDH1 PE=1 SV=3	CADH1_HUMAN	?	TRUE	.509929123	1.8	2.03	1.0432	0	2.57	0	3.91
302	TRUE	Empty	Calcium-binding mitochondrial carrier protein Aralar2 OS=Homo sapiens GN=SLC25A13 PE=1 SV=2	CMC2_HUMAN	?	TRUE	0.01245054	4.882180739	0	0.99746	0	2.57	1.43	0.93978
303	TRUE	Empty	Calcium-binding mitochondrial carrier protein SCaMC-1 OS=Homo sapiens GN=SLC25A24 PE=1 SV=2	SCMC1_HUMAN	?		0.18202165	4	17.683	0	0	41.732	17.79	11.277
304	TRUE	Empty	Calcium-binding protein 39 OS=Homo sapiens GN=CAB39 PE=1 SV=1	CAB39_HUMAN	40 kDa	TRUE	.082815077	2.7	0	1.0432	2.24	2.09	3.85	4.89
305	TRUE	Empty	Calcyclin-binding protein OS=Homo sapiens GN=CACYBP PE=1 SV=2	CYBP_HUMAN	?		0.79476342	0.8	0	34.425	12.967	14.904	11.86	12.217
306	TRUE	Empty	Calcyphosin OS=Homo sapiens GN=CAPS PE=1 SV=1	CAYP1_HUMAN	?		0.277766028	3.6	0	0	0.99746	0	1.43	1.96
307	TRUE	Empty	Calmodulin OS=Homo sapiens GN=CALM1 PE=1 SV=2	CALM_HUMAN	17 kDa	TRUE	0.62499055	0.8	6.631	9.86	4.73	2.09	3.85	10.338
308	TRUE	Empty	Calmodulin-like protein 5 OS=Homo sapiens GN=CALML5 PE=1 SV=2	CALL5_HUMAN	16 kDa		0.122021578	0.3	6.631	22.95	29.924	3.61	9.85	4.89
309	TRUE	Empty	Calnexin OS=Homo sapiens GN=CANX PE=1 SV=2	CALX_HUMAN	?		0.066967129	3.5	11.052	1.0432	0.99746	20.866	12.707	12.217
310	TRUE	Empty	Calpain small subunit 1 OS=Homo sapiens GN=CAPNS1 PE=1 SV=1	CPNS1_HUMAN	28 kDa	TRUE	.887886748	0.9	0	6.259	8.71	5.65	5.99	2.94
311	TRUE	Empty	Calpain-1 catalytic subunit OS=Homo sapiens GN=CAPN1 PE=1 SV=1	CAN1_HUMAN	82 kDa	TRUE	.006927817	11.29068462	0	2.0863	2.24	12.669	21.178	23.495
312	TRUE	Empty	Calpain-2 catalytic subunit OS=Homo sapiens GN=CAPN2 PE=1 SV=6	CAN2_HUMAN	?	TRUE	0.06853503	INF	0	0	0	0.74522	2.14	0.93978
313	TRUE	Empty	Calpastatin OS=Homo sapiens GN=CAST PE=1 SV=4	ICAL_HUMAN	?	TRUE	.882916777	1.1	0	14.604	11.969	8.26	7.42	12.217
314	TRUE	Empty	Calponin-2 OS=Homo sapiens GN=CNN2 PE=1 SV=4	CNN2_HUMAN	?	TRUE	.118703811	9.5	0	0	0.99746	2.09	0.84713	5.87
315	TRUE	Empty	Calreticulin OS=Homo sapiens GN=CALR PE=1 SV=1	CALR_HUMAN	48 kDa		0.292671775	2	22.103	6.259	1.49	26.083	9.85	24.434
316	TRUE	Empty	cAMP-dependent protein kinase catalytic subunit alpha OS=Homo sapiens GN=PRKACA PE=1 SV=2	KAPCA_HUMAN	?	TRUE	.191631292	3.1	4.06	0	0	2.57	6.71	4.89
317	TRUE	Empty	cAMP-dependent protein kinase type II-alpha regulatory subunit OS=Homo sapiens GN=PRKAR2A PE=1 SV=2	KAP2_HUMAN	?	TRUE	.017814887	5.731437852	0.99746	0	0	2.57	2.14	0.93978
318	TRUE	Empty	Cancer-related nucleoside-triphosphatase OS=Homo sapiens GN=NTPCR PE=1 SV=1	NTPCR_HUMAN	21 kDa		0.374455941	1.7	0	1.0432	0.99746	0.74522	1.43	0.93978
319	TRUE	Empty	CAP-Gly domain-containing linker protein 1 OS=Homo sapiens GN=CLIP1 PE=1 SV=2	CLIP1_HUMAN	?	TRUE	0.76413443	0.8	0	2.0863	1.49	2.57	0	0.93978
320	TRUE	Empty	Caprin-1 OS=Homo sapiens GN=CAPRIN1 PE=1 SV=2	CAPR1_HUMAN	?		0.873156475	0.9	0	9.86	8.71	3.61	3.85	9.78
321	TRUE	Empty	Carbonic anhydrase 2 OS=Homo sapiens GN=CA2 PE=1 SV=2	CAH2_HUMAN	29 kDa		0.146372392	0.5	15.472	37.554	21.944	10.433	14.401	14.097
322	TRUE	Empty	Carbonyl reductase [NADPH] 1 OS=Homo sapiens GN=CBR1 PE=1 SV=3	CBR1_HUMAN	?	TRUE	0.84630378	1.1	6.631	11.475	14.962	8.74	13.554	13.157
323	TRUE	Empty	Carboxymethylenebutenolidase homolog OS=Homo sapiens GN=CMBL PE=1 SV=1	CMBL_HUMAN	28 kDa		0.784057816	0.7	0	0	3.98	0	0.84713	1.96
324	TRUE	Empty	Carcinoembryonic antigen-related cell adhesion molecule 5 OS=Homo sapiens GN=CEACAM5 PE=1 SV=3	CEAM5_HUMAN	?	TRUE	.323128365	3.5	0	2.0863	0	0	2.14	4.89

325	TRUE	Empty	Carnitine O-palmitoyltransferase 1, liver isoform OS=Homo sapiens GN=CPT1A PE=1 SV=2	CPT1A_HUMAN	?		0.279257698	INF	0	0	0	5.65	0.84713	0
326	TRUE	Empty	Casein kinase I isoform alpha OS=Homo sapiens GN=CSNK1A1 PE=1 SV=2	KC1A_HUMAN	?	TRUE	.122761597	3.9	2.03	0	0	1.04	4.57	2.94
327	TRUE	Empty	Casein kinase II subunit alpha OS=Homo sapiens GN=CSNK2A1 PE=1 SV=1	CSK21_HUMAN	?	TRUE	.106968772	5.3	0	0	0.99746	0.74522	1.43	2.94
328	TRUE	Empty	Casein kinase II subunit beta OS=Homo sapiens GN=CSNK2B PE=1 SV=1	CSK2B_HUMAN	25 kDa		0.194668816	2.6	0	2.0863	0.99746	3.61	3.85	0.93978
329	TRUE	Empty	Catalase OS=Homo sapiens GN=CAT PE=1 SV=3	CATA_HUMAN	60 kDa		0.093238996	4.2	0	3.1295	1.49	11.923	5.0828	4.89
330	TRUE	Empty	Catechol O-methyltransferase OS=Homo sapiens GN=COMT PE=1 SV=2	COMT_HUMAN	?		0.090276431	2.4	4.06	3.1295	2.24	12.669	5.99	6.85
331	TRUE	Empty	Catenin alpha-1 OS=Homo sapiens GN=CTNNA1 PE=1 SV=1	CTNA1_HUMAN	?	TRUE	.200546071	1.6	8.13	6.259	14.962	19.376	17.79	10.338
332	TRUE	Empty	Catenin beta-1 OS=Homo sapiens GN=CTNNB1 PE=1 SV=1	CTNB1_HUMAN	?	TRUE	.443363156	1.9	0	0	4.73	2.57	3.85	3.91
333	TRUE	Empty	Catenin delta-1 OS=Homo sapiens GN=CTNND1 PE=1 SV=1	CTND1_HUMAN	?		0.004127847	13.19389443	0	0	1.49	11.178	7.42	7.83
334	TRUE	Empty	Cathepsin B OS=Homo sapiens GN=CTSB PE=1 SV=3	CATB_HUMAN	38 kDa		0.088962811	0.4	4.06	11.475	7.97	2.09	3.85	3.91
335	TRUE	Empty	Cathepsin D OS=Homo sapiens GN=CTSD PE=1 SV=1	CATD_HUMAN	45 kDa		0.090938872	0.7	50.837	49.029	33.914	24.592	35.58	31.953
336	TRUE	Empty	Cathepsin L1 OS=Homo sapiens GN=CTSL PE=1 SV=2	CATL1_HUMAN	38 kDa	TRUE	0.77737516	1.3	0	2.0863	1.49	1.04	0	3.91
337	TRUE	Empty	CCR4-NOT transcription complex subunit 1 OS=Homo sapiens GN=CNOT1 PE=1 SV=2	CNOT1_HUMAN	?	TRUE	.011775165	14.49371403	0	0.99746	0	2.09	6.71	4.89
338	TRUE	Empty	CD166 antigen OS=Homo sapiens GN=ALCAM PE=1 SV=2	CD166_HUMAN	?		0.373900966	INF	0	0	0	2.57	0	0
339	TRUE	Empty	CD2 antigen cytoplasmic tail-binding protein 2 OS=Homo sapiens GN=CD2BP2 PE=1 SV=1	CD2B2_HUMAN	38 kDa		0.675387566	1.4	2.03	1.0432	5.47	8.74	2.14	1.96
340	TRUE	Empty	CD2-associated protein OS=Homo sapiens GN=CD2AP PE=1 SV=1	CD2AP_HUMAN	71 kDa		0.736671358	0.8	0	3.1295	1.49	2.57	0.84713	0.93978
341	TRUE	Empty	CD44 antigen OS=Homo sapiens GN=CD44 PE=1 SV=3	CD44_HUMAN	?		0.01245054	4.882180739	0	0	0.99746	2.57	1.43	0.93978
342	TRUE	Empty	CD59 glycoprotein OS=Homo sapiens GN=CD59 PE=1 SV=1	CD59_HUMAN	14 kDa		0.003020465	5.926453191	0	0	0.99746	1.04	2.14	1.96
343	TRUE	Empty	CD81 antigen OS=Homo sapiens GN=CD81 PE=1 SV=1	CD81_HUMAN	26 kDa		0.944043977	0.9	0	8.54	1.49	3.61	5.99	0
344	TRUE	Empty	CD9 antigen OS=Homo sapiens GN=CD9 PE=1 SV=4	CD9_HUMAN	25 kDa		0.483022542	0.5	0	4.1727	12.967	3.61	3.85	0.93978
345	TRUE	Empty	CDGSH iron-sulfur domain-containing protein 2 OS=Homo sapiens GN=CISD2 PE=1 SV=1	CISD2_HUMAN	15 kDa		0.064414469	INF	0	0	0	1.04	1.43	4.89
346	TRUE	Empty	CDP-diacylglycerol--inositol 3-phosphatidyltransferase OS=Homo sapiens GN=CDIPT PE=1 SV=1	CDIPT_HUMAN	?		0.173372942	5.9	2.03	0	0	2.09	1.43	8.81
347	TRUE	Empty	Cell cycle and apoptosis regulator protein 2 OS=Homo sapiens GN=CCAR2 PE=1 SV=2	CCAR2_HUMAN	?		0.027346516	27.95657592	0	1.0432	0	8.74	5.99	15.037
348	TRUE	Empty	Cell division control protein 42 homolog OS=Homo sapiens GN=CDC42 PE=1 SV=2	CDC42_HUMAN	?	TRUE	.002245391	19.82565717	0.99746	0	0	7.22	7.42	4.89
349	TRUE	Empty	Cellular nucleic acid-binding protein OS=Homo sapiens GN=CNBP PE=1 SV=1	CNBP_HUMAN	?		0.349068061	0.4	0	12.518	18.952	5.65	6.71	0.93978
350	TRUE	Empty	Cellular retinoic acid-binding protein 2 OS=Homo sapiens GN=CRABP2 PE=1 SV=2	RABP2_HUMAN	16 kDa	TRUE	.371969959	1.8	11.052	10.432	14.962	6.707	19.484	37.591
351	TRUE	Empty	Centrin-2 OS=Homo sapiens GN=CETN2 PE=1 SV=1	CETN2_HUMAN	20 kDa	TRUE	.416420095	0.4	0	4.1727	1.49	0	1.43	0.93978
352	TRUE	Empty	Ceramide synthase 2 OS=Homo sapiens GN=CERS2 PE=1 SV=1	CERS2_HUMAN	45 kDa		0.226345874	INF	0	0	0	0	3.85	0.93978
353	TRUE	Empty	Cingulin OS=Homo sapiens GN=CGN PE=1 SV=2	CING_HUMAN	?	TRUE	.577751678	0.7	0	5.59	7.97	2.57	1.43	4.89
354	TRUE	Empty	Citrate synthase, mitochondrial OS=Homo sapiens GN=CS PE=1 SV=2	CISY_HUMAN	52 kDa		0.019646104	21.74352856	0	0	0.99746	3.61	7.42	10.338
355	TRUE	Empty	C-Jun-amino-terminal kinase-interacting protein 4 OS=Homo sapiens GN=SPAG9 PE=1 SV=4	JIP4_HUMAN	?	TRUE	.179911888	2.9	0	3.1295	0.99746	6.707	2.14	2.94
356	TRUE	Empty	Clathrin heavy chain 1 OS=Homo sapiens GN=CLTC PE=1 SV=5	CLH1_HUMAN	?	TRUE	.074816381	2.3	125.99	32.338	22.942	126.69	149.94	146.61
357	TRUE	Empty	Clathrin interactor 1 OS=Homo sapiens GN=CLINT1 PE=1 SV=1	EPN4_HUMAN	?		0.921029287	0.9	0	4.1727	3.98	2.57	1.43	3.91

358	TRUE	Empty	Clathrin light chain B OS=Homo sapiens GN=CLTB PE=1 SV=1	CLCB_HUMAN	?	TRUE	0.37963503	0.2	0	1.0432	8.71	0.74522	0.84713	0
359	TRUE	Empty	Cleavage and polyadenylation specificity factor subunit 1 OS=Homo sapiens GN=CPSF1 PE=1 SV=2	CPSF1_HUMAN	161 kDa	TRUE	.373900966	INF	0	0	0	1.04	0	0
360	TRUE	Empty	Cleavage and polyadenylation specificity factor subunit 3 OS=Homo sapiens GN=CPSF3 PE=1 SV=1	CPSF3_HUMAN	77 kDa	TRUE	.147672906	INF	0	0	0	0	1.43	0.93978
361	TRUE	Empty	Cleavage and polyadenylation specificity factor subunit 5 OS=Homo sapiens GN=NUDT21 PE=1 SV=1	CPSF5_HUMAN	26 kDa		0.582536923	1.2	6.631	6.259	7.97	9.78	11.86	3.91
362	TRUE	Empty	Cleavage and polyadenylation specificity factor subunit 6 OS=Homo sapiens GN=CPSF6 PE=1 SV=2	CPSF6_HUMAN	?		0.963334705	1	0	5.59	8.71	6.707	5.0828	2.94
363	TRUE	Empty	Cleavage and polyadenylation specificity factor subunit 7 OS=Homo sapiens GN=CPSF7 PE=1 SV=1	CPSF7_HUMAN	?	TRUE	.482026799	1.5	0	4.1727	1.49	3.61	2.14	2.94
364	TRUE	Empty	Cleavage stimulation factor subunit 2 tau variant OS=Homo sapiens GN=CSTF2T PE=1 SV=1	CSTFT_HUMAN	64 kDa	TRUE	.067797762	2.7	0	2.0863	1.49	3.61	2.14	4.89
365	TRUE	Empty	Clustered mitochondria protein homolog OS=Homo sapiens GN=CLUH PE=1 SV=2	CLU_HUMAN	147 kDa		0.140218517	INF	0	0	0	0	1.43	2.94
366	TRUE	Empty	C-Myc-binding protein OS=Homo sapiens GN=MYCBP PE=1 SV=3	MYCBP_HUMAN	12 kDa		0.607859167	0.6	0	4.1727	6.22	5.65	1.43	0
367	TRUE	Empty	Coagulation factor V OS=Homo sapiens GN=F5 PE=1 SV=4	FA5_HUMAN	252 kDa		0.069585698	3.2	0	1.0432	4.73	5.17	5.0828	8.81
368	TRUE	Empty	Coatomer subunit alpha OS=Homo sapiens GN=COPA PE=1 SV=2	COPA_HUMAN	?	TRUE	0.00621089	6.024621177	8.13	0	1.49	17.885	22.025	25.374
369	TRUE	Empty	Coatomer subunit beta OS=Homo sapiens GN=COPB1 PE=1 SV=3	COPB_HUMAN	107 kDa	TRUE	.000193602	12.40187333	0	1.0432	2.24	17.885	15.248	16.916
370	TRUE	Empty	Coatomer subunit beta' OS=Homo sapiens GN=COPB2 PE=1 SV=2	COPB2_HUMAN	?		0.015200776	3.359556622	11.052	2.0863	2.24	17.885	20.331	15.976
371	TRUE	Empty	Coatomer subunit delta OS=Homo sapiens GN=ARCN1 PE=1 SV=1	COPD_HUMAN	?	TRUE	.017502731	2.854724882	4.06	2.0863	1.49	8.26	5.99	9.78
372	TRUE	Empty	Coatomer subunit epsilon OS=Homo sapiens GN=COPE PE=1 SV=3	COPE_HUMAN	?		0.004776955	9.305034788	0	0	0.99746	2.09	2.14	3.91
373	TRUE	Empty	Coatomer subunit gamma-1 OS=Homo sapiens GN=COPG1 PE=1 SV=1	COPG1_HUMAN	98 kDa	TRUE	.000914255	5.266562445	4.06	3.1295	4.73	18.63	22.025	25.374
374	TRUE	Empty	Coatomer subunit gamma-2 OS=Homo sapiens GN=COPG2 PE=1 SV=1	COPG2_HUMAN	?	TRUE	.079931037	3.9	4.06	3.1295	2.24	6.707	12.707	21.615
375	TRUE	Empty	Cofilin-1 OS=Homo sapiens GN=CFL1 PE=1 SV=3	COF1_HUMAN	19 kDa	TRUE	.738797939	0.9	70.73	150.22	122.69	93.152	105.89	118.41
376	TRUE	Empty	Coiled-coil and C2 domain-containing protein 1A OS=Homo sapiens GN=CC2D1A PE=1 SV=1	C2D1A_HUMAN	?	TRUE	.026917445	2.358667219	2.03	1.0432	0.99746	3.61	2.14	3.91
377	TRUE	Empty	Coiled-coil domain-containing protein 25 OS=Homo sapiens GN=CCDC25 PE=1 SV=2	CCD25_HUMAN	?		0.189953018	INF	0	0	0	2.57	0.84713	0
378	TRUE	Empty	Coiled-coil domain-containing protein 47 OS=Homo sapiens GN=CCDC47 PE=1 SV=1	CCD47_HUMAN	?	TRUE	.013972038	17.49132797	0.99746	0	0	5.65	8.13	3.91
379	TRUE	Empty	Cold shock domain-containing protein E1 OS=Homo sapiens GN=CSDE1 PE=1 SV=2	CSDE1_HUMAN	?	TRUE	.105071446	2.5	0	3.1295	6.22	7.22	6.71	11.277
380	TRUE	Empty	Cold-inducible RNA-binding protein OS=Homo sapiens GN=CIRBP PE=1 SV=1	CIRBP_HUMAN	?	TRUE	.080788661	0.5	6.631	6.259	8.71	0.74522	5.0828	4.89
381	TRUE	Empty	Complement C3 OS=Homo sapiens GN=C3 PE=1 SV=2	CO3_HUMAN	187 kDa	TRUE	.849337677	0.7	0	0	1.49	1.04	0	0
382	TRUE	Empty	Complement component 1 Q subcomponent-binding protein, mitochondrial OS=Homo sapiens GN=C1QBP PE=1 SV=1	C1QBP_HUMAN	31 kDa		0.133459228	1.8	6.631	2.0863	2.24	6.707	8.13	5.87
383	TRUE	Empty	Condensin complex subunit 1 OS=Homo sapiens GN=NCAPD2 PE=1 SV=3	CND1_HUMAN	157 kDa	TRUE	.043958178	13.18519038	0	0.99746	0	1.04	5.0828	6.85
384	TRUE	Empty	Conserved oligomeric Golgi complex subunit 2 OS=Homo sapiens GN=COG2 PE=1 SV=1	COG2_HUMAN	?		0.373900966	INF	0	0	0	0	1.43	0

385	TRUE	Empty	Conserved oligomeric Golgi complex subunit 8 OS=Homo sapiens GN=COG8 PE=1 SV=2	COG8_HUMAN	68 kDa		0.120323626	INF	0	0	0	1.04	0.84713	4.89
386	TRUE	Empty	Constitutive coactivator of PPAR-gamma-like protein 1 OS=Homo sapiens GN=FAM120A PE=1 SV=2	F120A_HUMAN	?	TRUE	.927527477	1.1	4.06	0	0.99746	2.57	1.43	1.96
387	TRUE	Empty	COP9 signalosome complex subunit 1 OS=Homo sapiens GN=GPS1 PE=1 SV=4	CSN1_HUMAN	?	TRUE	.117184678	INF	0	0	0	0	1.43	1.96
388	TRUE	Empty	COP9 signalosome complex subunit 4 OS=Homo sapiens GN=COPS4 PE=1 SV=1	CSN4_HUMAN	?	TRUE	.016501622	9.949972931	0.99746	0	0	4.13	1.43	3.91
389	TRUE	Empty	COP9 signalosome complex subunit 6 OS=Homo sapiens GN=COPS6 PE=1 SV=1	CSN6_HUMAN	36 kDa		0.177767042	INF	0	0	0	2.57	0	0.93978
390	TRUE	Empty	Copine-1 OS=Homo sapiens GN=CPNE1 PE=1 SV=1	CPNE1_HUMAN	59 kDa		0.140218517	INF	0	0	0	0	1.43	2.94
391	TRUE	Empty	Copine-3 OS=Homo sapiens GN=CPNE3 PE=1 SV=1	CPNE3_HUMAN	60 kDa	TRUE	.229855168	2.3	15.472	1.0432	3.98	13.414	23.72	9.78
392	TRUE	Empty	Copper transport protein ATOX1 OS=Homo sapiens GN=ATOX1 PE=1 SV=1	ATOX1_HUMAN	7 kDa		0.348672532	0.4	0	3.1295	4.73	1.04	1.43	0
393	TRUE	Empty	Core histone macro-H2A.1 OS=Homo sapiens GN=H2AFY PE=1 SV=4	H2AY_HUMAN	?	TRUE	.192496369	2.4	4.06	0	0.99746	5.17	4.57	2.94
394	TRUE	Empty	Coronin-1A OS=Homo sapiens GN=CORO1A PE=1 SV=4	COR1A_HUMAN	51 kDa	TRUE	.063017666	INF	0	0	0	3.61	0.84713	1.96
395	TRUE	Empty	Coronin-1B OS=Homo sapiens GN=CORO1B PE=1 SV=1	COR1B_HUMAN	54 kDa	TRUE	.229574336	7	0	0	0.99746	2.57	0	4.89
396	TRUE	Empty	Coronin-7 OS=Homo sapiens GN=CORO7 PE=1 SV=2	CORO7_HUMAN	?	TRUE	.278146739	3	4.06	0	0	7.22	5.0828	0.93978
397	TRUE	Empty	COUP transcription factor 2 OS=Homo sapiens GN=NR2F2 PE=1 SV=1	COT2_HUMAN	?	TRUE	.094905451	INF	0	0	0	0.74522	0.84713	2.94
398	TRUE	Empty	Crk-like protein OS=Homo sapiens GN=CRKL PE=1 SV=1	CRKL_HUMAN	34 kDa	TRUE	.799101856	1.3	0	4.1727	0.99746	3.61	0	2.94
399	TRUE	Empty	C-terminal-binding protein 2 OS=Homo sapiens GN=CTBP2 PE=1 SV=1	CTBP2_HUMAN	?	TRUE	.024028039	8.799367331	0	1.0432	0	3.61	1.43	3.91
400	TRUE	Empty	CTP synthase 1 OS=Homo sapiens GN=CTPS1 PE=1 SV=2	PYRG1_HUMAN	?	TRUE	.374640578	0.7	2.03	3.1295	1.49	0.74522	1.43	2.94
401	TRUE	Empty	CTP synthase 2 OS=Homo sapiens GN=CTPS2 PE=1 SV=1	PYRG2_HUMAN	66 kDa	TRUE	.687473951	1.3	0	2.0863	1.49	0.74522	1.43	2.94
402	TRUE	Empty	CUGBP Elav-like family member 1 OS=Homo sapiens GN=CELF1 PE=1 SV=2	CELF1_HUMAN	?	TRUE	0.00055667	11.8435827	0	0.99746	0	3.61	3.85	4.89
403	TRUE	Empty	Cullin-1 OS=Homo sapiens GN=CUL1 PE=1 SV=2	CUL1_HUMAN	90 kDa	TRUE	0.03308882	3.48079121	0.99746	0	0	0.74522	0.84713	1.96
404	TRUE	Empty	Cullin-2 OS=Homo sapiens GN=CUL2 PE=1 SV=2	CUL2_HUMAN	?		0.06372838	9.4	0	0	0.99746	1.04	5.0828	2.94
405	TRUE	Empty	Cullin-associated NEDD8-dissociated protein 1 OS=Homo sapiens GN=CAND1 PE=1 SV=2	CAND1_HUMAN	?	TRUE	.019714117	2.98292363	28.734	3.1295	11.969	46.949	41.51	42.29
406	TRUE	Empty	Cyclin-dependent kinase 1 OS=Homo sapiens GN=CDK1 PE=1 SV=3	CDK1_HUMAN	?	TRUE	.084566581	INF	0	0	0	2.09	0.84713	0.93978
407	TRUE	Empty	Cyclin-dependent kinase 9 OS=Homo sapiens GN=CDK9 PE=1 SV=3	CDK9_HUMAN	?	TRUE	.136121777	INF	0	0	0	1.04	0	0.93978
408	TRUE	Empty	Cyclin-dependent-like kinase 5 OS=Homo sapiens GN=CDK5 PE=1 SV=3	CDK5_HUMAN	?	TRUE	.152478611	INF	0	0	0	1.04	0	2.94
409	TRUE	Empty	Cyclin-G-associated kinase OS=Homo sapiens GN=GAK PE=1 SV=2	GAK_HUMAN	?	TRUE	.217218164	INF	0	0	0	0	0.84713	2.94
410	TRUE	Empty	Cystathionine beta-synthase OS=Homo sapiens GN=CBS PE=1 SV=2	CBS_HUMAN	?	TRUE	.310947221	3.2	0	0	0.99746	1.04	1.43	0
411	TRUE	Empty	Cystatin-B OS=Homo sapiens GN=CSTB PE=1 SV=2	CYTB_HUMAN	11 kDa		0.168960308	0.6	22.103	29.209	15.959	15.65	18.637	8.81
412	TRUE	Empty	Cysteine and glycine-rich protein 1 OS=Homo sapiens GN=CSRP1 PE=1 SV=3	CSRP1_HUMAN	21 kDa	TRUE	.370063286	0.5	4.06	12.518	24.936	2.09	10.166	9.78
413	TRUE	Empty	Cysteine and histidine-rich domain-containing protein 1 OS=Homo sapiens GN=CHORDC1 PE=1 SV=2	CHRD1_HUMAN	?		0.136121777	INF	0	0	0	1.04	0	0.93978
414	TRUE	Empty	Cysteine-rich protein 2 OS=Homo sapiens GN=CRIP2 PE=1 SV=1	CRIP2_HUMAN	?	TRUE	.226008646	0.5	4.06	25.036	22.942	8.74	9.85	6.85
415	TRUE	Empty	Cytochrome b-c1 complex subunit 1, mitochondrial OS=Homo sapiens GN=UQCRC1 PE=1 SV=3	QCR1_HUMAN	53 kDa	TRUE	.111135393	1.8	6.631	8.54	5.47	9.78	10.166	17.856
416	TRUE	Empty	Cytochrome b-c1 complex subunit 2, mitochondrial OS=Homo sapiens GN=UQCRC2 PE=1 SV=3	QCR2_HUMAN	48 kDa		0.186328973	1.4	6.631	11.475	5.47	14.159	9.85	11.277

417	TRUE	Empty	Cytochrome b-c1 complex subunit 7 OS=Homo sapiens GN=UQCRB PE=1 SV=2	QCR7_HUMAN	?		0.701916933	1.3	0	4.1727	1.49	4.13	0.84713	2.94
418	TRUE	Empty	Cytochrome b-c1 complex subunit Rieske, mitochondrial OS=Homo sapiens GN=UQCRFS1 PE=1 SV=2	UCRI_HUMAN	30 kDa		0.287811707	1.9	0	12.518	3.98	11.923	6.71	13.157
419	TRUE	Empty	Cytochrome c OS=Homo sapiens GN=CYCS PE=1 SV=2	CYC_HUMAN	12 kDa		0.095090395	2.1	4.06	4.1727	5.47	6.707	9.85	15.037
420	TRUE	Empty	Cytochrome c oxidase subunit 2 OS=Homo sapiens GN=MT-CO2 PE=1 SV=1	COX2_HUMAN	26 kDa		0.585522401	1.7	13.262	0	0	8.74	3.85	10.338
421	TRUE	Empty	Cytochrome c oxidase subunit 4 isoform 1, mitochondrial OS=Homo sapiens GN=COX4I1 PE=1 SV=1	COX41_HUMAN	20 kDa	TRUE	.000650923	14.93423295	0	0.99746	0	5.17	4.57	4.89
422	TRUE	Empty	Cytochrome c oxidase subunit 5A, mitochondrial OS=Homo sapiens GN=COX5A PE=1 SV=2	COX5A_HUMAN	17 kDa		0.207817272	12	2.03	0	0	20.121	3.85	3.91
423	TRUE	Empty	Cytochrome c oxidase subunit 5B, mitochondrial OS=Homo sapiens GN=COX5B PE=1 SV=2	COX5B_HUMAN	14 kDa		0.064300471	3.3	0	4.1727	0.99746	4.13	5.0828	7.83
424	TRUE	Empty	Cytochrome c oxidase subunit 6B1 OS=Homo sapiens GN=COX6B1 PE=1 SV=2	CX6B1_HUMAN	10 kDa		0.499627956	0.6	0	7.22	5.47	3.61	2.14	1.96
425	TRUE	Empty	Cytochrome c oxidase subunit 7A2, mitochondrial OS=Homo sapiens GN=COX7A2 PE=1 SV=1	CX7A2_HUMAN	9 kDa		0.946101728	1	4.06	0	1.49	0.74522	2.14	2.94
426	TRUE	Empty	Cytochrome c1, heme protein, mitochondrial OS=Homo sapiens GN=CYC1 PE=1 SV=3	CY1_HUMAN	35 kDa		0.061088963	INF	0	0	0	2.57	1.43	5.87
427	TRUE	Empty	Cytochrome P450 1B1 OS=Homo sapiens GN=CYP1B1 PE=1 SV=2	CP1B1_HUMAN	61 kDa		0.217218164	INF	0	0	0	0	0.84713	2.94
428	TRUE	Empty	Cytoplasmic aconitate hydratase OS=Homo sapiens GN=ACO1 PE=1 SV=3	ACOC_HUMAN	98 kDa		0.001501257	11.94584244	0.99746	0	0	2.09	4.57	4.89
429	TRUE	Empty	Cytoplasmic dynein 1 heavy chain 1 OS=Homo sapiens GN=DYNC1H1 PE=1 SV=5	DYHC1_HUMAN	532 kDa	TRUE	.019664439	9.149826572	13.262	0	0	26.083	39.815	55.447
430	TRUE	Empty	Cytoplasmic dynein 1 light intermediate chain 1 OS=Homo sapiens GN=DYNC1LI1 PE=1 SV=3	DC1L1_HUMAN	57 kDa	TRUE	.832416383	0.9	0	4.1727	6.22	5.65	3.85	0.93978
431	TRUE	Empty	Cytoplasmic dynein 1 light intermediate chain 2 OS=Homo sapiens GN=DYNC1LI2 PE=1 SV=1	DC1L2_HUMAN	?	TRUE	.750161253	0.8	0	3.1295	2.24	1.04	2.14	0.93978
432	TRUE	Empty	Cytoplasmic FMR1-interacting protein 1 OS=Homo sapiens GN=CYFIP1 PE=1 SV=1	CYFP1_HUMAN	?	TRUE	.000157434	20.215748	0	0	0.99746	5.17	7.42	6.85
433	TRUE	Empty	Cytoskeleton-associated protein 4 OS=Homo sapiens GN=CKAP4 PE=1 SV=2	CKAP4_HUMAN	66 kDa	TRUE	.006165014	18.03420689	0	0.99746	0	7.22	6.71	3.91
434	TRUE	Empty	Cytoskeleton-associated protein 5 OS=Homo sapiens GN=CKAP5 PE=1 SV=3	CKAP5_HUMAN	?	TRUE	.040274969	4.032853448	0	0	0.99746	2.57	0.84713	0.93978
435	TRUE	Empty	Cytosol aminopeptidase OS=Homo sapiens GN=LAP3 PE=1 SV=3	AMPL_HUMAN	?	TRUE	0.90686667	1	11.052	32.338	22.942	18.63	23.72	21.615
436	TRUE	Empty	Cytosolic acyl coenzyme A thioester hydrolase OS=Homo sapiens GN=ACOT7 PE=1 SV=3	BACH_HUMAN	?	TRUE	.177116089	3.5	2.03	2.0863	1.49	11.178	10.166	0.93978
437	TRUE	Empty	Cytosolic Fe-S cluster assembly factor NUBP1 OS=Homo sapiens GN=NUBP1 PE=1 SV=2	NUBP1_HUMAN	?	TRUE	.874205584	0.9	0	5.59	5.47	2.57	5.99	1.96
438	TRUE	Empty	Cytosolic non-specific dipeptidase OS=Homo sapiens GN=CNDP2 PE=1 SV=2	CNDP2_HUMAN	?		0.038788539	3.556178172	0	2.0863	0.99746	3.61	2.14	4.89
439	TRUE	Empty	Cytosolic purine 5'-nucleotidase OS=Homo sapiens GN=NT5C2 PE=1 SV=1	5NTC_HUMAN	?	TRUE	.008306911	22.21884586	0	1.0432	0	5.65	7.42	10.338
440	TRUE	Empty	D-3-phosphoglycerate dehydrogenase OS=Homo sapiens GN=PHGDH PE=1 SV=4	SERA_HUMAN	57 kDa		0.006668773	2.077804611	15.472	18.777	9.46	29.809	33.885	28.194
441	TRUE	Empty	DAZ-associated protein 1 OS=Homo sapiens GN=DAZAP1 PE=1 SV=1	DAZP1_HUMAN	?		0.234163855	0.1	0	5.59	1.49	0.74522	0	0
442	TRUE	Empty	DBIRD complex subunit ZNF326 OS=Homo sapiens GN=ZNF326 PE=1 SV=2	ZN326_HUMAN	?	TRUE	.792670078	0.8	0	1.0432	2.24	1.04	0.84713	0.93978
443	TRUE	Empty	dCTP pyrophosphatase 1 OS=Homo sapiens GN=DCTPP1 PE=1 SV=1	DCTP1_HUMAN	19 kDa		0.989266989	1	8.13	3.1295	5.47	8.26	2.14	6.85

444	TRUE	Empty	DDB1- and CUL4-associated factor 7 OS=Homo sapiens GN=DCAF7 PE=1 SV=1	DCAF7_HUMAN	?	TRUE	.116315977	0	0	2.0863	1.49	0	0	0
445	TRUE	Empty	D-dopachrome decarboxylase OS=Homo sapiens GN=DDT PE=1 SV=3	DOPD_HUMAN	?		0.914994424	1.1	0	14.604	5.47	6.707	5.0828	10.338
446	TRUE	Empty	DDRGK domain-containing protein 1 OS=Homo sapiens GN=DDRGK1 PE=1 SV=2	DDRGK_HUMAN	?	TRUE	0.00734386	8.16784633	0.99746	0	0	3.61	2.14	1.96
447	TRUE	Empty	Dehydrogenase/reductase SDR family member 4 OS=Homo sapiens GN=DHRS4 PE=1 SV=3	DHRS4_HUMAN	?	TRUE	.036639133	5.722164297	0	0.99746	0	2.09	0.84713	1.96
448	TRUE	Empty	Delta(14)-sterol reductase OS=Homo sapiens GN=TM7SF2 PE=2 SV=3	ERG24_HUMAN	?		0.169759736	INF	0	0	0	0	0.84713	1.96
449	TRUE	Empty	Delta(24)-sterol reductase OS=Homo sapiens GN=DHCR24 PE=1 SV=2	DHC24_HUMAN	?	TRUE	0.01245054	4.882180739	0	0	0.99746	2.57	1.43	0.93978
450	TRUE	Empty	Delta(3,5)-Delta(2,4)-dienoyl-CoA isomerase, mitochondrial OS=Homo sapiens GN=ECH1 PE=1 SV=2	ECH1_HUMAN	36 kDa		0.12357825	2.2	8.13	3.1295	1.49	12.669	11.013	6.85
451	TRUE	Empty	Delta-1-pyrroline-5-carboxylate synthase OS=Homo sapiens GN=ALDH18A1 PE=1 SV=2	P5CS_HUMAN	?		0.06534674	4.8	2.03	0	0	4.13	4.57	1.96
452	TRUE	Empty	Delta-aminolevulinic acid dehydratase OS=Homo sapiens GN=ALAD PE=1 SV=1	HEM2_HUMAN	?		0.766616669	0.8	0	6.259	4.73	0	4.57	4.89
453	TRUE	Empty	Deoxyhypusine synthase OS=Homo sapiens GN=DHPS PE=1 SV=1	DHYS_HUMAN	?		0.373900966	0	0	0	1.49	0	0	0
454	TRUE	Empty	Deoxyribonuclease-2-alpha OS=Homo sapiens GN=DNASE2 PE=1 SV=2	DNS2A_HUMAN	?		0.799197877	1.2	0	3.1295	0.99746	1.04	2.14	0.93978
455	TRUE	Empty	Deoxyuridine 5'-triphosphate nucleotidohydrolase, mitochondrial OS=Homo sapiens GN=DUT PE=1 SV=4	DUT_HUMAN	?	TRUE	.818394512	1.1	2.03	3.1295	6.22	4.13	3.85	5.87
456	TRUE	Empty	Derlin-1 OS=Homo sapiens GN=DERL1 PE=1 SV=1	DERL1_HUMAN	?		0.125523833	6.4	2.03	0	0	2.57	3.85	8.81
457	TRUE	Empty	Desmoplakin OS=Homo sapiens GN=DSP PE=1 SV=3	DESP_HUMAN	?	TRUE	.038995226	3.790319591	4.06	2.0863	6.22	23.847	16.943	10.338
458	TRUE	Empty	Destrin OS=Homo sapiens GN=DSTN PE=1 SV=3	DEST_HUMAN	?	TRUE	.136263267	0.5	6.631	18.777	17.954	8.26	5.0828	6.85
459	TRUE	Empty	Developmentally-regulated GTP-binding protein 2 OS=Homo sapiens GN=DRG2 PE=1 SV=1	DRG2_HUMAN	41 kDa	TRUE	.373900966	INF	0	0	0	1.04	0	0
460	TRUE	Empty	Diablo homolog, mitochondrial OS=Homo sapiens GN=DIABLO PE=1 SV=1	DBLOH_HUMAN	?	TRUE	.316446148	0.3	0	3.1295	3.98	2.57	0	0
461	TRUE	Empty	Dihydrolipoyl dehydrogenase, mitochondrial OS=Homo sapiens GN=DLD PE=1 SV=2	DLDH_HUMAN	?	TRUE	.060888089	1.7	13.262	11.475	6.22	15.65	16.096	22.555
462	TRUE	Empty	Dihydrolipoyllysine-residue succinyltransferase component of 2-oxoglutarate dehydrogenase complex, mitochondrial OS=Homo sapiens GN=DLST PE=1 SV=4	ODO2_HUMAN	?	TRUE	.162453876	3.3	0	3.1295	0.99746	2.57	7.42	3.91
463	TRUE	Empty	Dihydropyrimidinase-related protein 2 OS=Homo sapiens GN=DPYSL2 PE=1 SV=1	DPYL2_HUMAN	?	TRUE	0.04347111	3.214422229	0	2.0863	1.49	5.65	5.0828	2.94
464	TRUE	Empty	Dipeptidyl peptidase 2 OS=Homo sapiens GN=DPP7 PE=1 SV=3	DPP2_HUMAN	54 kDa	TRUE	0.96154385	1.1	0	0	2.24	2.57	0	0.93978
465	TRUE	Empty	Dipeptidyl peptidase 3 OS=Homo sapiens GN=DPP3 PE=1 SV=2	DPP3_HUMAN	?		0.008244721	3.870805856	6.631	3.1295	2.24	20.866	14.401	14.097
466	TRUE	Empty	DNA damage-binding protein 1 OS=Homo sapiens GN=DDB1 PE=1 SV=1	DDB1_HUMAN	?		0.180455608	2	0	7.22	12.967	14.904	10.166	15.037
467	TRUE	Empty	DNA fragmentation factor subunit alpha OS=Homo sapiens GN=DFFA PE=1 SV=1	DFFA_HUMAN	?		0.466013214	0.5	0	4.1727	5.47	0	3.85	1.96
468	TRUE	Empty	DNA polymerase delta catalytic subunit OS=Homo sapiens GN=POLD1 PE=1 SV=2	DPOD1_HUMAN	124 kDa	TRUE	.061814651	4.9	0	0	0.99746	2.57	1.43	0.93978
469	TRUE	Empty	DNA repair protein XRCC1 OS=Homo sapiens GN=XRCC1 PE=1 SV=2	XRCC1_HUMAN	69 kDa	TRUE	.313355175	2.7	0	1.0432	1.49	4.13	0	3.91
470	TRUE	Empty	DNA replication licensing factor MCM2 OS=Homo sapiens GN=MCM2 PE=1 SV=4	MCM2_HUMAN	102 kDa		0.037396137	24.01239147	0.99746	0	0	3.61	12.707	7.83

471	TRUE	Empty	DNA replication licensing factor MCM3 OS=Homo sapiens GN=MCM3 PE=1 SV=3	MCM3_HUMAN	?		0.084741888	2.3	11.052	1.0432	4.73	15.65	10.166	14.097
472	TRUE	Empty	DNA replication licensing factor MCM4 OS=Homo sapiens GN=MCM4 PE=1 SV=5	MCM4_HUMAN	97 kDa	TRUE	.001582051	3.578173166	2.03	4.1727	6.22	14.904	16.943	15.976
473	TRUE	Empty	DNA replication licensing factor MCM5 OS=Homo sapiens GN=MCM5 PE=1 SV=5	MCM5_HUMAN	82 kDa	TRUE	.002170477	27.81254386	0.99746	0	0	9.78	6.71	11.277
474	TRUE	Empty	DNA replication licensing factor MCM6 OS=Homo sapiens GN=MCM6 PE=1 SV=1	MCM6_HUMAN	93 kDa	TRUE	.000471505	11.70519839	2.03	0	0	8.26	8.13	8.81
475	TRUE	Empty	DNA replication licensing factor MCM7 OS=Homo sapiens GN=MCM7 PE=1 SV=4	MCM7_HUMAN	?	TRUE	.003108288	20.95350844	0	1.0432	0	5.17	9.85	6.85
476	TRUE	Empty	DNA topoisomerase 1 OS=Homo sapiens GN=TOP1 PE=1 SV=2	TOP1_HUMAN	91 kDa	TRUE	.001858442	9.709068252	0	1.0432	0	2.09	3.85	3.91
477	TRUE	Empty	DNA topoisomerase 2-beta OS=Homo sapiens GN=TOP2B PE=1 SV=3	TOP2B_HUMAN	?	TRUE	.437700045	2	2.03	0	0	0.74522	1.43	1.96
478	TRUE	Empty	DNA-(apurinic or apyrimidinic site) lyase OS=Homo sapiens GN=APEX1 PE=1 SV=2	APEX1_HUMAN	36 kDa		0.022424161	5.632645296	2.03	0	1.49	11.178	5.99	6.85
479	TRUE	Empty	DNA-dependent protein kinase catalytic subunit OS=Homo sapiens GN=PRKDC PE=1 SV=3	PRKDC_HUMAN	?	TRUE	.001886819	9.913680782	11.052	0	0	38.751	31.344	39.471
480	TRUE	Empty	DNA-directed RNA polymerase II subunit RPB2 OS=Homo sapiens GN=POLR2B PE=1 SV=1	RPB2_HUMAN	134 kDa	TRUE	0.63960284	2.2	0	0	0.99746	2.57	0	0
481	TRUE	Empty	DNA-directed RNA polymerase III subunit RPC1 OS=Homo sapiens GN=POLR3A PE=1 SV=2	RPC1_HUMAN	156 kDa	TRUE	0.43108597	0.1	0	6.259	0	0.74522	0	0
482	TRUE	Empty	DNA-directed RNA polymerases I and III subunit RPAC1 OS=Homo sapiens GN=POLR1C PE=1 SV=1	RPAC1_HUMAN	?		0.00485573	6.868646362	0	0	0.99746	1.04	2.14	2.94
483	TRUE	Empty	DnaJ homolog subfamily A member 1 OS=Homo sapiens GN=DNAJA1 PE=1 SV=2	DNJA1_HUMAN	?	TRUE	0.57220724	0.7	0	12.518	9.46	5.17	2.14	6.85
484	TRUE	Empty	DnaJ homolog subfamily B member 1 OS=Homo sapiens GN=DNAJB1 PE=1 SV=4	DNJB1_HUMAN	?	TRUE	.177913222	0.4	4.06	13.561	5.47	4.13	2.14	2.94
485	TRUE	Empty	DnaJ homolog subfamily B member 6 OS=Homo sapiens GN=DNAJB6 PE=1 SV=2	DNJB6_HUMAN	?	TRUE	.041547618	5.170061957	0	0	0.99746	1.04	0.84713	2.94
486	TRUE	Empty	DnaJ homolog subfamily C member 13 OS=Homo sapiens GN=DNAJC13 PE=1 SV=5	DJC13_HUMAN	254 kDa		0.074940238	4.2	0	0	0.99746	1.04	0.84713	1.96
487	TRUE	Empty	DnaJ homolog subfamily C member 9 OS=Homo sapiens GN=DNAJC9 PE=1 SV=1	DNJC9_HUMAN	30 kDa		0.528254655	0.3	0	0	2.24	0	0.84713	0
488	TRUE	Empty	DNL-type zinc finger protein OS=Homo sapiens GN=DNLZ PE=1 SV=1	DNLZ_HUMAN	19 kDa		0.373900966	INF	0	0	0	1.04	0	0
489	TRUE	Empty	Dolichol-phosphate mannosyltransferase subunit 1 OS=Homo sapiens GN=DPM1 PE=1 SV=1	DPM1_HUMAN	30 kDa		0.219982104	4.8	0	0	0.99746	2.57	2.14	0
490	TRUE	Empty	Dolichyl-diphosphooligosaccharide--protein glycosyltransferase 48 kDa subunit OS=Homo sapiens GN=DDOST PE=1 SV=4	OST48_HUMAN	?	TRUE	.201766144	3	15.472	0	0	9.78	12.707	23.495
491	TRUE	Empty	Dolichyl-diphosphooligosaccharide--protein glycosyltransferase subunit 1 OS=Homo sapiens GN=RPN1 PE=1 SV=1	RPN1_HUMAN	69 kDa		0.029045105	4.922366992	11.052	0	0	14.904	16.943	22.555
492	TRUE	Empty	Dolichyl-diphosphooligosaccharide--protein glycosyltransferase subunit 2 OS=Homo sapiens GN=RPN2 PE=1 SV=3	RPN2_HUMAN	?	TRUE	.591379601	1.6	22.103	0	0	12.669	10.166	12.217
493	TRUE	Empty	Dolichyl-diphosphooligosaccharide--protein glycosyltransferase subunit STT3A OS=Homo sapiens GN=STT3A PE=1 SV=2	STT3A_HUMAN	?	TRUE	.226345874	INF	0	0	0	0	3.85	0.93978
494	TRUE	Empty	Dolichyl-diphosphooligosaccharide--protein glycosyltransferase subunit STT3B OS=Homo sapiens GN=STT3B PE=1 SV=1	STT3B_HUMAN	94 kDa	TRUE	.041547618	5.170061957	0.99746	0	0	1.04	0.84713	2.94

495	TRUE	Empty	Double-strand-break repair protein rad21 homolog OS=Homo sapiens GN=RAD21 PE=1 SV=2	RAD21_HUMAN	72 kDa	TRUE	.089717672	4.2	0	1.0432	0.99746	2.57	1.43	4.89
496	TRUE	Empty	Double-stranded RNA-binding protein Staufen homolog 1 OS=Homo sapiens GN=STAU1 PE=1 SV=2	STAU1_HUMAN	?	TRUE	.982802861	1	0	4.1727	8.71	2.57	5.0828	5.87
497	TRUE	Empty	Double-stranded RNA-specific adenosine deaminase OS=Homo sapiens GN=ADAR PE=1 SV=4	DSRAD_HUMAN	?		0.011744795	8.55793716	0	0	0.99746	2.57	2.14	3.91
498	TRUE	Empty	Drebrin-like protein OS=Homo sapiens GN=DBNL PE=1 SV=1	DBNL_HUMAN	?		0.245785844	0.3	0	18.777	13.964	1.04	5.0828	2.94
499	TRUE	Empty	D-tyrosyl-tRNA(Tyr) deacylase 1 OS=Homo sapiens GN=DTD1 PE=1 SV=2	DTD1_HUMAN	23 kDa		0.256806469	0.4	8.13	2.0863	3.98	0.74522	1.43	3.91
500	TRUE	Empty	Dual specificity protein phosphatase 3 OS=Homo sapiens GN=DUSP3 PE=1 SV=1	DUS3_HUMAN	?		0.060312045	2.7	0	2.0863	0.99746	2.57	3.85	2.94
501	TRUE	Empty	Dynactin subunit 1 OS=Homo sapiens GN=DCTN1 PE=1 SV=3	DCTN1_HUMAN	?	TRUE	.000114504	5.077196078	0.99746	0	0	1.04	1.43	1.96
502	TRUE	Empty	Dynactin subunit 2 OS=Homo sapiens GN=DCTN2 PE=1 SV=4	DCTN2_HUMAN	?		0.628370931	1.3	2.03	3.1295	8.71	4.13	5.99	7.83
503	TRUE	Empty	Dynamin-1-like protein OS=Homo sapiens GN=DNM1L PE=1 SV=2	DNM1L_HUMAN	?		0.028213971	5.91725984	0	0.99746	0	2.57	0.84713	2.94
504	TRUE	Empty	Dynamin-2 OS=Homo sapiens GN=DNM2 PE=1 SV=2	DYN2_HUMAN	?	TRUE	.011688212	5.462358141	2.03	3.1295	0	8.26	12.707	7.83
505	TRUE	Empty	Dynamin-like 120 kDa protein, mitochondrial OS=Homo sapiens GN=OPA1 PE=1 SV=3	OPA1_HUMAN	?	TRUE	.373900966	INF	0	0	0	0	0	1.96
506	TRUE	Empty	Dynein assembly factor 5, axonemal OS=Homo sapiens GN=DNAAF5 PE=1 SV=4	DAAF5_HUMAN	?	TRUE	.217218164	INF	0	0	0	0	0.84713	2.94
507	TRUE	Empty	Dynein light chain 2, cytoplasmic OS=Homo sapiens GN=DYNLL2 PE=1 SV=1	DYL2_HUMAN	10 kDa	TRUE	.097375341	0.4	4.06	2.0863	4.73	1.04	2.14	0
508	TRUE	Empty	Dynein light chain roadblock-type 1 OS=Homo sapiens GN=DYNLRB1 PE=1 SV=3	DLRB1_HUMAN	?	TRUE	.756551235	1.3	0	1.0432	2.24	0.74522	0.84713	3.91
509	TRUE	Empty	E2 ubiquitin-conjugating enzyme OS=Homo sapiens GN=UBE2O PE=1 SV=3	sp|Q9C0C9|UBE2O_	141 kDa	TRUE	0.39870266	3.6	0	0	0.99746	0.74522	0	2.94
510	TRUE	Empty	E3 ubiquitin/ISG15 ligase TRIM25 OS=Homo sapiens GN=TRIM25 PE=1 SV=2	TRI25_HUMAN	71 kDa		0.019014979	6.274442696	4.06	1.0432	0	14.904	11.86	7.83
511	TRUE	Empty	E3 ubiquitin-protein ligase BRE1B OS=Homo sapiens GN=RNF40 PE=1 SV=4	BRE1B_HUMAN	?	TRUE	.248726999	INF	0	0	0	0	0.84713	3.91
512	TRUE	Empty	E3 ubiquitin-protein ligase HUWE1 OS=Homo sapiens GN=HUWE1 PE=1 SV=3	HUWE1_HUMAN	?	TRUE	.014132239	6.610296779	11.052	0	0	20.866	21.178	31.013
513	TRUE	Empty	E3 ubiquitin-protein ligase CHIP OS=Homo sapiens GN=STUB1 PE=1 SV=2	CHIP_HUMAN	?		0.136189297	2.3	0	3.1295	1.49	2.09	3.85	5.87
514	TRUE	Empty	E3 ubiquitin-protein ligase LRSAM1 OS=Homo sapiens GN=LRSAM1 PE=1 SV=1	LRSM1_HUMAN	?		0.118629825	INF	0	0	0	2.09	2.14	0
515	TRUE	Empty	E3 ubiquitin-protein ligase RNF114 OS=Homo sapiens GN=RNF114 PE=1 SV=1	RN114_HUMAN	?		0.182857041	0.2	0	4.1727	4.73	0.74522	0.84713	0
516	TRUE	Empty	E3 ubiquitin-protein ligase RNF213 OS=Homo sapiens GN=RNF213 PE=1 SV=3	RN213_HUMAN	?	TRUE	.373900966	INF	0	0	0	0	0	2.94
517	TRUE	Empty	E3 ubiquitin-protein ligase TRIM23 OS=Homo sapiens GN=TRIM23 PE=1 SV=1	TRI23_HUMAN	?		0.373900966	INF	0	0	0	0	1.43	0
518	TRUE	Empty	E3 ubiquitin-protein ligase TRIM33 OS=Homo sapiens GN=TRIM33 PE=1 SV=3	TRI33_HUMAN	?	TRUE	.052971722	16	0	0	0.99746	4.13	2.14	8.81
519	TRUE	Empty	E3 ubiquitin-protein ligase UBR4 OS=Homo sapiens GN=UBR4 PE=1 SV=1	UBR4_HUMAN	?	TRUE	.015162985	19.00016041	0	0	0.99746	4.13	5.0828	9.78
520	TRUE	Empty	E3 ubiquitin-protein ligase UBR5 OS=Homo sapiens GN=UBR5 PE=1 SV=2	UBR5_HUMAN	?	TRUE	.311490087	3.2	0	1.0432	0	1.04	0	1.96
521	TRUE	Empty	E3 UFM1-protein ligase 1 OS=Homo sapiens GN=UFL1 PE=1 SV=2	UFL1_HUMAN	?	TRUE	.224916699	3.4	2.03	0	0	2.57	4.57	0.93978

522	TRUE	Empty	Early endosome antigen 1 OS=Homo sapiens GN=EEA1 PE=1 SV=2	EEA1_HUMAN	162 kDa		0.502582938	0.6	0	4.1727	6.22	2.57	2.14	1.96
523	TRUE	Empty	EH domain-containing protein 1 OS=Homo sapiens GN=EHD1 PE=1 SV=2	EHD1_HUMAN	61 kDa	TRUE	.175558359	2.5	4.06	0	1.49	5.17	2.14	7.83
524	TRUE	Empty	EH domain-containing protein 4 OS=Homo sapiens GN=EHD4 PE=1 SV=1	EHD4_HUMAN	61 kDa	TRUE	.000315223	6.673651074	0	0.99746	0	2.57	2.14	1.96
525	TRUE	Empty	EKC/KEOPS complex subunit LAGE3 OS=Homo sapiens GN=LAGE3 PE=1 SV=2	LAGE3_HUMAN	15 kDa		0.842158589	1.1	2.03	1.0432	0.99746	2.09	0	1.96
526	TRUE	Empty	ELAV-like protein 1 OS=Homo sapiens GN=ELAVL1 PE=1 SV=2	ELAV1_HUMAN	?		0.6617871	0.7	2.03	13.561	30.921	8.26	16.096	9.78
527	TRUE	Empty	Electron transfer flavoprotein subunit alpha, mitochondrial OS=Homo sapiens GN=ETFA PE=1 SV=1	ETFA_HUMAN	?		0.037559261	1.926866259	6.631	13.561	5.47	16.395	15.248	18.796
528	TRUE	Empty	Electron transfer flavoprotein subunit beta OS=Homo sapiens GN=ETFB PE=1 SV=3	ETFB_HUMAN	?		0.003475822	7.54793467	2.03	0	0	5.17	5.0828	5.87
529	TRUE	Empty	Elongation factor 1-alpha 2 OS=Homo sapiens GN=EEF1A2 PE=1 SV=1	EF1A2_HUMAN	50 kDa	TRUE	.199860117	1.6	187.88	134.57	55.858	274.24	153.33	191.72
530	TRUE	Empty	Elongation factor 1-beta OS=Homo sapiens GN=EEF1B2 PE=1 SV=3	EF1B_HUMAN	25 kDa	TRUE	.830889948	0.9	15.472	9.86	14.962	17.14	9.85	11.277
531	TRUE	Empty	Elongation factor 1-delta OS=Homo sapiens GN=EEF1D PE=1 SV=5	EF1D_HUMAN	?	TRUE	.028919631	0.677815631	44.206	56.331	58.85	32.044	35.58	40.411
532	TRUE	Empty	Elongation factor 1-gamma OS=Homo sapiens GN=EEF1G PE=1 SV=3	EF1G_HUMAN	?		0.009524998	2.404486135	22.103	8.54	13.964	34.28	33.038	39.471
533	TRUE	Empty	Elongation factor 2 OS=Homo sapiens GN=EEF2 PE=1 SV=4	EF2_HUMAN	95 kDa	TRUE	.106392674	2.1	128.2	33.382	31.919	150.53	137.24	114.65
534	TRUE	Empty	Elongation factor G, mitochondrial OS=Homo sapiens GN=GFM1 PE=1 SV=2	EFGM_HUMAN	?	TRUE	.373900966	INF	0	0	0	1.04	0	0
535	TRUE	Empty	Elongation factor Ts, mitochondrial OS=Homo sapiens GN=TSFM PE=1 SV=2	EFTS_HUMAN	?	TRUE	.090036366	INF	0	0	0	4.13	0.84713	1.96
536	TRUE	Empty	Elongation factor Tu, mitochondrial OS=Homo sapiens GN=TUFM PE=1 SV=2	EFTU_HUMAN	50 kDa		0.32944426	2	15.472	2.0863	0	17.14	9.85	9.78
537	TRUE	Empty	Endonuclease G, mitochondrial OS=Homo sapiens GN=ENDOG PE=1 SV=4	NUCG_HUMAN	33 kDa	TRUE	.475552494	2.5	0	1.0432	0.99746	0	4.57	0.93978
538	TRUE	Empty	Endophilin-B2 OS=Homo sapiens GN=SH3GLB2 PE=1 SV=1	SHLB2_HUMAN	?		0.817889465	1.1	2.03	14.604	10.972	14.904	7.42	8.81
539	TRUE	Empty	Endoplasmic reticulum mannosyl-oligosaccharide 1,2-alpha-mannosidase OS=Homo sapiens GN=MAN1B1 PE=1 SV=2	MA1B1_HUMAN	80 kDa		0.119086248	INF	0	0	0	2.57	0	1.96
540	TRUE	Empty	Endoplasmic reticulum resident protein 29 OS=Homo sapiens GN=ERP29 PE=1 SV=4	ERP29_HUMAN	?	TRUE	.268333783	1.7	2.03	14.604	9.46	10.433	13.554	22.555
541	TRUE	Empty	Endoplasmic reticulum resident protein 44 OS=Homo sapiens GN=ERP44 PE=1 SV=1	ERP44_HUMAN	47 kDa		0.139470421	3.1	4.06	0	0.99746	7.22	2.14	6.85
542	TRUE	Empty	Endoplasmin OS=Homo sapiens GN=HSP90B1 PE=1 SV=1	ENPL_HUMAN	92 kDa	TRUE	.078555915	3.9	39.786	0	4.73	84.955	38.968	51.688
543	TRUE	Empty	Enhancer of mRNA-decapping protein 3 OS=Homo sapiens GN=EDC3 PE=1 SV=1	EDC3_HUMAN	56 kDa		0.003651919	4.134982856	0.99746	0	0	1.04	1.43	0.93978
544	TRUE	Empty	Enhancer of mRNA-decapping protein 4 OS=Homo sapiens GN=EDC4 PE=1 SV=1	EDC4_HUMAN	?		0.023060505	6.578428268	2.03	0	0	3.61	4.57	6.85
545	TRUE	Empty	Enolase-phosphatase E1 OS=Homo sapiens GN=ENOPH1 PE=1 SV=1	ENOPH_HUMAN	?		0.46135138	2.5	0	1.0432	0	0.74522	0	1.96
546	TRUE	Empty	Enoyl-CoA delta isomerase 1, mitochondrial OS=Homo sapiens GN=ECI1 PE=1 SV=1	ECI1_HUMAN	?	TRUE	.227514196	1.4	4.06	6.259	5.47	8.74	5.0828	9.78
547	TRUE	Empty	Enoyl-CoA hydratase, mitochondrial OS=Homo sapiens GN=ECHS1 PE=1 SV=4	ECHM_HUMAN	31 kDa	TRUE	.005653132	24.22994406	0	0.99746	0	9.78	5.0828	9.78
548	TRUE	Empty	Ensconsin OS=Homo sapiens GN=MAP7 PE=1 SV=1	MAP7_HUMAN	?	TRUE	.367591425	2.1	0	0	1.49	1.04	0.84713	1.96
549	TRUE	Empty	Envoplakin OS=Homo sapiens GN=EVPL PE=1 SV=3	EVPL_HUMAN	232 kDa	TRUE	.251391811	0.3	0	6.259	6.22	2.57	1.43	0

550	TRUE	Empty	Epidermal growth factor receptor kinase substrate 8-like protein 1 OS=Homo sapiens GN=EPS8L1 PE=1 SV=3	ES8L1_HUMAN	?		0.373900966	0	0	0	1.49	0	0	0
551	TRUE	Empty	Epidermal growth factor receptor kinase substrate 8-like protein 2 OS=Homo sapiens GN=EPS8L2 PE=1 SV=2	ES8L2_HUMAN	?	TRUE	.373900966	INF	0	0	0	0	2.14	0
552	TRUE	Empty	Epidermal growth factor receptor substrate 15 OS=Homo sapiens GN=EPS15 PE=1 SV=2	EPS15_HUMAN	?	TRUE	.147672906	INF	0	0	0	0	1.43	0.93978
553	TRUE	Empty	Epidermal growth factor receptor substrate 15-like 1 OS=Homo sapiens GN=EPS15L1 PE=1 SV=1	EP15R_HUMAN	?		0.097636	2.3	0	2.0863	1.49	2.57	3.85	3.91
554	TRUE	Empty	Epiplakin OS=Homo sapiens GN=EPPK1 PE=1 SV=2	EPIPL_HUMAN	556 kDa	TRUE	.075079764	2.5	83.992	12.518	15.959	89.426	102.5	90.219
555	TRUE	Empty	Epithelial cell adhesion molecule OS=Homo sapiens GN=EPCAM PE=1 SV=2	EPCAM_HUMAN	35 kDa		0.817919934	1.4	0	1.0432	0	1.04	0	0
556	TRUE	Empty	Epithelial splicing regulatory protein 1 OS=Homo sapiens GN=ESRP1 PE=1 SV=2	ESRP1_HUMAN	?	TRUE	.497736304	1.5	0	3.1295	2.24	1.04	5.0828	2.94
557	TRUE	Empty	Epithelial splicing regulatory protein 2 OS=Homo sapiens GN=ESRP2 PE=1 SV=1	ESRP2_HUMAN	?	TRUE	.373900966	INF	0	0	0	0	2.14	0
558	TRUE	Empty	Epoxide hydrolase 1 OS=Homo sapiens GN=EPHX1 PE=1 SV=1	HYEP_HUMAN	53 kDa		0.03308882	3.48079121	0	0	0.99746	0.74522	0.84713	1.96
559	TRUE	Empty	ER membrane protein complex subunit 1 OS=Homo sapiens GN=EMC1 PE=1 SV=1	EMC1_HUMAN	?		0.060961291	INF	0	0	0	0.74522	4.57	4.89
560	TRUE	Empty	ER membrane protein complex subunit 2 OS=Homo sapiens GN=EMC2 PE=1 SV=1	EMC2_HUMAN	35 kDa		0.119086248	INF	0	0	0	2.57	0	1.96
561	TRUE	Empty	Erlin-2 OS=Homo sapiens GN=ERLIN2 PE=1 SV=1	ERLN2_HUMAN	?	TRUE	.104344154	4.2	2.03	0	0	2.09	1.43	4.89
562	TRUE	Empty	ERO1-like protein alpha OS=Homo sapiens GN=ERO1A PE=1 SV=2	ERO1A_HUMAN	54 kDa	TRUE	.032392471	7.327792593	0	0	0.99746	2.09	3.85	0.93978
563	TRUE	Empty	Erythrocyte band 7 integral membrane protein OS=Homo sapiens GN=STOM PE=1 SV=3	STOM_HUMAN	?		0.007186925	5.719427639	2.03	3.1295	0.99746	15.65	9.85	11.277
564	TRUE	Empty	ES1 protein homolog, mitochondrial OS=Homo sapiens GN=C21orf33 PE=1 SV=3	ES1_HUMAN	?		0.254431521	0.3	0	4.1727	4.73	0.74522	0	1.96
565	TRUE	Empty	Estradiol 17-beta-dehydrogenase 8 OS=Homo sapiens GN=HSD17B8 PE=1 SV=2	DHB8_HUMAN	27 kDa		0.373900966	INF	0	0	0	1.04	0	0
566	TRUE	Empty	Ethanolamine-phosphate cytidylyltransferase OS=Homo sapiens GN=PCYT2 PE=1 SV=1	PCY2_HUMAN	?		0.373900966	INF	0	0	0	0	1.43	0
567	TRUE	Empty	Eukaryotic initiation factor 4A-I OS=Homo sapiens GN=EIF4A1 PE=1 SV=1	IF4A1_HUMAN	?	TRUE	.043439178	2.619032565	37.575	9.86	9.46	42.477	47.44	59.206
568	TRUE	Empty	Eukaryotic initiation factor 4A-III OS=Homo sapiens GN=EIF4A3 PE=1 SV=4	IF4A3_HUMAN	47 kDa	TRUE	.138340583	1.6	6.631	5.59	1.49	7.22	7.42	6.85
569	TRUE	Empty	Eukaryotic peptide chain release factor GTP-binding subunit ERF3A OS=Homo sapiens GN=GSPT1 PE=1 SV=1	ERF3A_HUMAN	?	TRUE	.554356598	2.1	0	0	5.47	8.26	3.85	0
570	TRUE	Empty	Eukaryotic peptide chain release factor subunit 1 OS=Homo sapiens GN=ETF1 PE=1 SV=3	ERF1_HUMAN	?	TRUE	.007195192	4.987713391	0	2.0863	2.24	6.707	10.166	8.81
571	TRUE	Empty	Eukaryotic translation elongation factor 1 epsilon-1 OS=Homo sapiens GN=EEF1E1 PE=1 SV=1	MCA3_HUMAN	?		0.015910638	0.179725665	2.03	4.1727	2.24	0.74522	0	0.93978
572	TRUE	Empty	Eukaryotic translation initiation factor 1b OS=Homo sapiens GN=EIF1B PE=1 SV=2	EIF1B_HUMAN	13 kDa	TRUE	.074940238	4.2	0	0	0.99746	1.04	0.84713	1.96
573	TRUE	Empty	Eukaryotic translation initiation factor 2 subunit 1 OS=Homo sapiens GN=EIF2S1 PE=1 SV=3	IF2A_HUMAN	36 kDa		0.164168852	1.8	13.262	2.0863	7.97	12.669	11.86	17.856
574	TRUE	Empty	Eukaryotic translation initiation factor 2 subunit 2 OS=Homo sapiens GN=EIF2S2 PE=1 SV=2	IF2B_HUMAN	38 kDa		0.359102554	2	0	1.0432	1.49	0.74522	3.85	1.96
575	TRUE	Empty	Eukaryotic translation initiation factor 2 subunit 3 OS=Homo sapiens GN=EIF2S3 PE=1 SV=3	IF2G_HUMAN	51 kDa		0.550446381	1.3	2.03	3.1295	8.71	5.65	5.99	7.83
576	TRUE	Empty	Eukaryotic translation initiation factor 2A OS=Homo sapiens GN=EIF2A PE=1 SV=3	EIF2A_HUMAN	?		0.146055018	3.4	0	0	0.99746	0.74522	1.43	0.93978

577	TRUE	Empty	Eukaryotic translation initiation factor 3 subunit A OS=Homo sapiens GN=EIF3A PE=1 SV=1	EIF3A_HUMAN	?	TRUE	.053455521	8.9	2.03	1.0432	0	4.13	15.248	9.78
578	TRUE	Empty	Eukaryotic translation initiation factor 3 subunit B OS=Homo sapiens GN=EIF3B PE=1 SV=3	EIF3B_HUMAN	?	TRUE	.148107419	2.5	13.262	1.0432	1.49	9.78	13.554	16.916
579	TRUE	Empty	Eukaryotic translation initiation factor 3 subunit C-like protein OS=Homo sapiens GN=EIF3CL PE=3 SV=1	EIFCL_HUMAN (+1)	105 kDa	TRUE	.174870512	2.7	6.631	0	0	5.65	5.99	6.85
580	TRUE	Empty	Eukaryotic translation initiation factor 3 subunit D OS=Homo sapiens GN=EIF3D PE=1 SV=1	EIF3D_HUMAN	?		0.115134731	3.8	0	1.0432	3.98	3.61	5.0828	10.338
581	TRUE	Empty	Eukaryotic translation initiation factor 3 subunit E OS=Homo sapiens GN=EIF3E PE=1 SV=1	EIF3E_HUMAN	52 kDa		0.140004642	5.8	2.03	1.0432	0	5.17	1.43	11.277
582	TRUE	Empty	Eukaryotic translation initiation factor 3 subunit F OS=Homo sapiens GN=EIF3F PE=1 SV=1	EIF3F_HUMAN	38 kDa		0.045615209	6.267657527	0	0	3.98	10.433	4.57	10.338
583	TRUE	Empty	Eukaryotic translation initiation factor 3 subunit G OS=Homo sapiens GN=EIF3G PE=1 SV=2	EIF3G_HUMAN	36 kDa	TRUE	.366763367	0.5	0	6.259	4.73	1.04	0.84713	2.94
584	TRUE	Empty	Eukaryotic translation initiation factor 3 subunit H OS=Homo sapiens GN=EIF3H PE=1 SV=1	EIF3H_HUMAN	40 kDa		0.507804467	2.4	0	1.0432	0.99746	0.74522	4.57	0
585	TRUE	Empty	Eukaryotic translation initiation factor 3 subunit I OS=Homo sapiens GN=EIF3I PE=1 SV=1	EIF3I_HUMAN	37 kDa	TRUE	.193912178	5.7	0	2.0863	1.49	15.65	5.99	1.96
586	TRUE	Empty	Eukaryotic translation initiation factor 3 subunit L OS=Homo sapiens GN=EIF3L PE=1 SV=1	EIF3L_HUMAN	?		0.00606701	13.64435667	0	0.99746	0	2.09	5.99	4.89
587	TRUE	Empty	Eukaryotic translation initiation factor 3 subunit M OS=Homo sapiens GN=EIF3M PE=1 SV=1	EIF3M_HUMAN	?		0.117185082	INF	0	0	0	0	2.14	2.94
588	TRUE	Empty	Eukaryotic translation initiation factor 4 gamma 1 OS=Homo sapiens GN=EIF4G1 PE=1 SV=4	IF4G1_HUMAN	?	TRUE	0.01782156	5.230113219	8.13	0	0	13.414	17.79	15.037
589	TRUE	Empty	Eukaryotic translation initiation factor 4 gamma 2 OS=Homo sapiens GN=EIF4G2 PE=1 SV=1	IF4G2_HUMAN	?	TRUE	.615399499	1.4	6.631	1.0432	0.99746	2.09	3.85	5.87
590	TRUE	Empty	Eukaryotic translation initiation factor 4B OS=Homo sapiens GN=EIF4B PE=1 SV=2	IF4B_HUMAN	?	TRUE	.065015792	0.2	6.631	19.82	11.969	2.09	2.14	3.91
591	TRUE	Empty	Eukaryotic translation initiation factor 4H OS=Homo sapiens GN=EIF4H PE=1 SV=5	IF4H_HUMAN	?	TRUE	.615576737	0.7	0	7.22	4.73	1.04	4.57	2.94
592	TRUE	Empty	Eukaryotic translation initiation factor 5 OS=Homo sapiens GN=EIF5 PE=1 SV=2	IF5_HUMAN	49 kDa	TRUE	.056487833	2.8	6.631	1.0432	1.49	11.178	9.85	6.85
593	TRUE	Empty	Eukaryotic translation initiation factor 5A-1 OS=Homo sapiens GN=EIF5A PE=1 SV=2	IF5A1_HUMAN	?	TRUE	.118035309	0.7	26.524	31.295	16.957	16.395	17.79	15.037
594	TRUE	Empty	Eukaryotic translation initiation factor 5B OS=Homo sapiens GN=EIF5B PE=1 SV=4	IF2P_HUMAN	139 kDa		0.044590634	4.488576668	0	1.0432	2.24	3.61	5.99	8.81
595	TRUE	Empty	Eukaryotic translation initiation factor 6 OS=Homo sapiens GN=EIF6 PE=1 SV=1	IF6_HUMAN	?		0.66629989	0.7	0	29.209	7.97	8.26	7.42	8.81
596	TRUE	Empty	Exosome complex component MTR3 OS=Homo sapiens GN=EXOSC6 PE=1 SV=1	EXOS6_HUMAN	28 kDa		0.528254655	0.3	0	0	2.24	0	0.84713	0
597	TRUE	Empty	Exosome complex component RRP4 OS=Homo sapiens GN=EXOSC2 PE=1 SV=2	EXOS2_HUMAN	?		0.791076242	1.3	0	1.0432	2.24	0.74522	3.85	0.93978
598	TRUE	Empty	Exosome complex component RRP45 OS=Homo sapiens GN=EXOSC9 PE=1 SV=3	EXOS9_HUMAN	?	TRUE	0.44708438	0.3	0	1.0432	3.98	0	1.43	0
599	TRUE	Empty	Exportin-1 OS=Homo sapiens GN=XPO1 PE=1 SV=1	XPO1_HUMAN	123 kDa	TRUE	.217545426	2.5	26.524	0	0	21.611	21.178	22.555
600	TRUE	Empty	Exportin-2 OS=Homo sapiens GN=CSE1L PE=1 SV=3	XPO2_HUMAN	?		0.072845407	3.4	30.944	1.0432	0.99746	44.713	29.65	37.591
601	TRUE	Empty	Exportin-5 OS=Homo sapiens GN=XPO5 PE=1 SV=1	XPO5_HUMAN	136 kDa	TRUE	.062200909	10	2.03	0	0	4.13	12.707	5.87
602	TRUE	Empty	Exportin-7 OS=Homo sapiens GN=XPO7 PE=1 SV=3	XPO7_HUMAN	124 kDa		0.002281953	6.766587131	0.99746	0	0	2.57	1.43	2.94
603	TRUE	Empty	Exportin-T OS=Homo sapiens GN=XPOT PE=1 SV=2	XPOT_HUMAN	110 kDa		0.033302108	4.818078994	4.06	0	0	5.65	7.42	8.81
604	TRUE	Empty	Extended synaptotagmin-1 OS=Homo sapiens GN=ESYT1 PE=1 SV=1	ESYT1_HUMAN	?		0.117655015	3.1	11.052	0	0	10.433	10.166	13.157
605	TRUE	Empty	Extended synaptotagmin-2 OS=Homo sapiens GN=ESYT2 PE=1 SV=1	ESYT2_HUMAN	?		0.373900966	INF	0	0	0	0	0	1.96

606	TRUE	Empty	Extracellular sulfatase Sulf-2 OS=Homo sapiens GN=SULF2 PE=1 SV=1	SULF2_HUMAN	?		0.117667821	INF	0	0	0	0.74522	3.85	0.93978
607	TRUE	Empty	Ezrin OS=Homo sapiens GN=EZR PE=1 SV=4	EZRI_HUMAN	69 kDa	TRUE	.741568903	1.1	35.365	14.604	20.947	21.611	27.108	29.133
608	TRUE	Empty	FACT complex subunit SPT16 OS=Homo sapiens GN=SUPT16H PE=1 SV=1	SP16H_HUMAN	120 kDa		0.00262192	17.27778558	0.99746	0	0	7.22	5.0828	4.89
609	TRUE	Empty	FACT complex subunit SSRP1 OS=Homo sapiens GN=SSRP1 PE=1 SV=1	SSRP1_HUMAN	81 kDa	TRUE	.709010335	1.4	0	0	5.47	2.57	1.43	4.89
610	TRUE	Empty	F-actin-capping protein subunit alpha-1 OS=Homo sapiens GN=CAPZA1 PE=1 SV=3	CAZA1_HUMAN	33 kDa	TRUE	.283620737	0.6	8.13	23.993	30.921	10.433	11.013	16.916
611	TRUE	Empty	F-actin-capping protein subunit alpha-2 OS=Homo sapiens GN=CAPZA2 PE=1 SV=3	CAZA2_HUMAN	?	TRUE	0.40536834	0.8	6.631	11.475	15.959	6.707	7.42	11.277
612	TRUE	Empty	F-actin-capping protein subunit beta OS=Homo sapiens GN=CAPZB PE=1 SV=4	CAPZB_HUMAN	?		0.75526219	1.2	4.06	7.22	20.947	13.414	15.248	9.78
613	TRUE	Empty	Far upstream element-binding protein 1 OS=Homo sapiens GN=FUBP1 PE=1 SV=3	FUBP1_HUMAN	?	TRUE	.333799196	0.7	15.472	32.338	41.893	18.63	17.79	26.314
614	TRUE	Empty	Far upstream element-binding protein 2 OS=Homo sapiens GN=KHSRP PE=1 SV=4	FUBP2_HUMAN	73 kDa	TRUE	.036955802	0.574790603	26.524	35.468	35.908	15.65	15.248	25.374
615	TRUE	Empty	Far upstream element-binding protein 3 OS=Homo sapiens GN=FUBP3 PE=1 SV=2	FUBP3_HUMAN	?	TRUE	.098930349	0.7	11.052	18.777	13.964	9.78	10.166	9.78
616	TRUE	Empty	Farnesyl pyrophosphate synthase OS=Homo sapiens GN=FDPS PE=1 SV=4	FPPS_HUMAN	?		0.00049079	12.59068033	0.99746	0	0	4.13	3.85	4.89
617	TRUE	Empty	Fascin OS=Homo sapiens GN=FSCN1 PE=1 SV=3	FSCN1_HUMAN	55 kDa		0.142831189	1.6	2.03	1.0432	0.99746	2.57	2.14	1.96
618	TRUE	Empty	Fatty acid synthase OS=Homo sapiens GN=FASN PE=1 SV=3	FAS_HUMAN	273 kDa	TRUE	.494590846	1.7	291.76	7.22	8.71	176.62	176.2	168.22
619	TRUE	Empty	Fatty acid-binding protein, epidermal OS=Homo sapiens GN=FABP5 PE=1 SV=3	FABP5_HUMAN	15 kDa	TRUE	.867045185	1.1	2.03	3.1295	2.24	3.61	4.57	0.93978
620	TRUE	Empty	F-box only protein 22 OS=Homo sapiens GN=FBXO22 PE=1 SV=1	FBX22_HUMAN	?		0.080922552	INF	0	0	0	0.74522	1.43	3.91
621	TRUE	Empty	Fermitin family homolog 2 OS=Homo sapiens GN=FERMT2 PE=1 SV=1	FERM2_HUMAN	?		0.009521936	4.227868787	0	0.99746	0	1.04	0.84713	1.96
622	TRUE	Empty	Filamin-A OS=Homo sapiens GN=FLNA PE=1 SV=4	FLNA_HUMAN	?	TRUE	.108753105	1.3	183.46	125.18	187.52	190.03	219.41	237.77
623	TRUE	Empty	Filamin-B OS=Homo sapiens GN=FLNB PE=1 SV=2	FLNB_HUMAN	?	TRUE	.216535841	0.6	64.099	140.83	158.6	68.56	87.255	78.002
624	TRUE	Empty	FK506-binding protein 15 OS=Homo sapiens GN=FKBP15 PE=1 SV=2	FKB15_HUMAN	?	TRUE	.005904554	5.839042271	0	1.0432	0.99746	2.09	4.57	4.89
625	TRUE	Empty	Flavin reductase (NADPH) OS=Homo sapiens GN=BLVRB PE=1 SV=3	BLVRB_HUMAN	22 kDa		0.121549451	0.4	11.052	38.597	35.908	6.707	12.707	13.157
626	TRUE	Empty	Flotillin-1 OS=Homo sapiens GN=FLOT1 PE=1 SV=3	FLOT1_HUMAN	?	TRUE	.030400164	5.179375614	0.99746	0	0	0.74522	2.14	1.96
627	TRUE	Empty	Four and a half LIM domains protein 2 OS=Homo sapiens GN=FHL2 PE=1 SV=3	FHL2_HUMAN	?		0.137246924	3.1	0	1.0432	0	1.04	0.84713	0.93978
628	TRUE	Empty	Fructose-1,6-bisphosphatase 1 OS=Homo sapiens GN=FBP1 PE=1 SV=5	F16P1_HUMAN	37 kDa	TRUE	.169233844	0.5	59.679	160.65	98.748	44.713	72.854	46.989
629	TRUE	Empty	Fructose-2,6-bisphosphatase TIGAR OS=Homo sapiens GN=TIGAR PE=1 SV=1	TIGAR_HUMAN	30 kDa		0.001127235	10.05213242	0	0.99746	0	3.61	2.14	3.91
630	TRUE	Empty	Fructose-bisphosphate aldolase A OS=Homo sapiens GN=ALDOA PE=1 SV=2	ALDOA_HUMAN	?	TRUE	.109056292	0.7	181.25	275.4	220.44	169.16	182.98	135.33
631	TRUE	Empty	Fructose-bisphosphate aldolase C OS=Homo sapiens GN=ALDOC PE=1 SV=2	ALDOC_HUMAN	39 kDa	TRUE	.492102883	0.6	11.052	3.1295	3.98	6.707	0.84713	3.91
632	TRUE	Empty	Fumarate hydratase, mitochondrial OS=Homo sapiens GN=FH PE=1 SV=3	FUMH_HUMAN	?	TRUE	.058785769	5	0	3.1295	0.99746	3.61	10.166	6.85
633	TRUE	Empty	Fumarylacetoacetase OS=Homo sapiens GN=FAH PE=1 SV=2	FAAA_HUMAN	?		0.88838267	0.8	0	0	3.98	0.74522	1.43	0.93978
634	TRUE	Empty	G1/S-specific cyclin-D1 OS=Homo sapiens GN=CCND1 PE=1 SV=1	CCND1_HUMAN	34 kDa		0.080922552	INF	0	0	0	0.74522	1.43	3.91

635	TRUE	Empty	Galactokinase OS=Homo sapiens GN=GALK1 PE=1 SV=1	GALK1_HUMAN	?	TRUE	.302113716	1.5	4.06	3.1295	6.22	5.65	5.99	11.277
636	TRUE	Empty	GALC_HUMAN-DECOY	GALC_HUMAN-DEC(	?		0.373900966	0	0	0	1.49	0	0	0
637	TRUE	Empty	Galectin-1 OS=Homo sapiens GN=LGALS1 PE=1 SV=2	LEG1_HUMAN	15 kDa		0.925381927	1.1	0	19.82	5.47	8.26	10.166	8.81
638	TRUE	Empty	Galectin-3 OS=Homo sapiens GN=LGALS3 PE=1 SV=5	LEG3_HUMAN	26 kDa		0.102937276	0.4	19.893	56.331	62.84	8.74	16.943	24.434
639	TRUE	Empty	Gamma-glutamyl hydrolase OS=Homo sapiens GN=GGH PE=1 SV=2	GGH_HUMAN	36 kDa		0.335302688	0.2	0	1.0432	4.73	0	0	0.93978
640	TRUE	Empty	Gamma-glutamylcyclotransferase OS=Homo sapiens GN=GGCT PE=1 SV=1	GGCT_HUMAN	?		0.148155676	2.1	0	3.1295	1.49	4.13	3.85	2.94
641	TRUE	Empty	GDP-L-fucose synthase OS=Homo sapiens GN=TSTA3 PE=1 SV=1	FCL_HUMAN	36 kDa		0.895738011	1.1	0	3.1295	3.98	2.57	1.43	3.91
642	TRUE	Empty	GDP-mannose 4,6 dehydratase OS=Homo sapiens GN=GMDS PE=1 SV=1	GMDS_HUMAN	?	TRUE	.373900966	0	0	0	1.49	0	0	0
643	TRUE	Empty	Gelsolin OS=Homo sapiens GN=GSN PE=1 SV=1	GELS_HUMAN	?	TRUE	.112291811	0.5	22.103	35.468	49.873	17.885	18.637	21.615
644	TRUE	Empty	Gem-associated protein 5 OS=Homo sapiens GN=GEMIN5 PE=1 SV=3	GEMI5_HUMAN	169 kDa	TRUE	.119086248	INF	0	0	0	2.57	0	1.96
645	TRUE	Empty	General transcription factor 3C polypeptide 3 OS=Homo sapiens GN=GTF3C3 PE=1 SV=1	TF3C3_HUMAN	?		0.231711402	INF	0	0	0	0.74522	0	2.94
646	TRUE	Empty	General transcription factor 3C polypeptide 4 OS=Homo sapiens GN=GTF3C4 PE=1 SV=2	TF3C4_HUMAN	92 kDa		0.071986351	5.9	0	0	0.99746	2.57	0.84713	2.94
647	TRUE	Empty	General transcription factor II-I OS=Homo sapiens GN=GTF2I PE=1 SV=2	GTF2I_HUMAN	?	TRUE	.828224327	1.2	11.052	0	1.49	6.707	0	9.78
648	TRUE	Empty	General vesicular transport factor p115 OS=Homo sapiens GN=USO1 PE=1 SV=2	USO1_HUMAN	?	TRUE	0.49364667	1.6	2.03	0	0.99746	1.04	0.84713	2.94
649	TRUE	Empty	Gephyrin OS=Homo sapiens GN=GPHN PE=1 SV=1	GEPH_HUMAN	?	TRUE	.271397858	2.3	0	1.0432	1.49	0.74522	2.14	3.91
650	TRUE	Empty	Gigaxonin OS=Homo sapiens GN=GAN PE=1 SV=1	GAN_HUMAN	68 kDa		0.013481328	7.907400307	0	1.0432	0	2.09	3.85	1.96
651	TRUE	Empty	Glia maturation factor beta OS=Homo sapiens GN=GMFB PE=1 SV=2	GMFB_HUMAN	17 kDa	TRUE	.443532528	0.4	0	1.0432	2.24	0.74522	0	0.93978
652	TRUE	Empty	Glucosamine 6-phosphate N-acetyltransferase OS=Homo sapiens GN=GNPNAT1 PE=1 SV=1	GNA1_HUMAN	21 kDa		0.009682446	5.793884152	0	2.0863	1.49	5.17	10.166	7.83
653	TRUE	Empty	Glucosamine-6-phosphate isomerase 1 OS=Homo sapiens GN=GNPDA1 PE=1 SV=1	GNPI1_HUMAN	?	TRUE	.456401571	1.6	0	2.0863	0.99746	1.04	2.14	0.93978
654	TRUE	Empty	Glucose-6-phosphate 1-dehydrogenase OS=Homo sapiens GN=G6PD PE=1 SV=4	G6PD_HUMAN	?		0.003589052	2.393575591	24.314	25.036	18.952	57.382	60.994	45.11
655	TRUE	Empty	Glucose-6-phosphate isomerase OS=Homo sapiens GN=GPI PE=1 SV=4	G6PI_HUMAN	?	TRUE	.549373894	0.8	81.782	34.425	74.809	63.344	62.688	28.194
656	TRUE	Empty	Glucose-induced degradation protein 8 homolog OS=Homo sapiens GN=GID8 PE=1 SV=1	GID8_HUMAN	27 kDa		0.087092203	5.6	0	0	0.99746	2.09	1.43	0.93978
657	TRUE	Empty	Glucosidase 2 subunit beta OS=Homo sapiens GN=PRKCSH PE=1 SV=2	GLU2B_HUMAN	?	TRUE	.563974417	1.4	15.472	1.0432	5.47	11.923	9.85	9.78
658	TRUE	Empty	Glutamate dehydrogenase 1, mitochondrial OS=Homo sapiens GN=GLUD1 PE=1 SV=2	DHE3_HUMAN	?	TRUE	.056327309	INF	0	0	0	17.14	3.85	11.277
659	TRUE	Empty	Glutamate dehydrogenase 2, mitochondrial OS=Homo sapiens GN=GLUD2 PE=1 SV=2	DHE4_HUMAN	61 kDa	TRUE	.037604234	13.8209051	0	0	0.99746	3.61	2.14	7.83
660	TRUE	Empty	Glutamate-rich WD repeat-containing protein 1 OS=Homo sapiens GN=GRWD1 PE=1 SV=1	GRWD1_HUMAN	49 kDa		0.993762925	1	2.03	2.0863	2.24	2.09	3.85	0.93978
661	TRUE	Empty	Glutamine--fructose-6-phosphate aminotransferase [isomerizing] 1 OS=Homo sapiens GN=GFPT1 PE=1 SV=3	GFPT1_HUMAN	?	TRUE	0.06066379	3.7	6.631	0	0	7.22	7.42	9.78
662	TRUE	Empty	Glutamine--tRNA ligase OS=Homo sapiens GN=QARS PE=1 SV=1	SYQ_HUMAN	?	TRUE	.000708106	24.43436328	0	0	0.99746	8.74	6.71	9.78
663	TRUE	Empty	Glutaredoxin-3 OS=Homo sapiens GN=GLRX3 PE=1 SV=2	GLRX3_HUMAN	37 kDa		0.029584164	2.615992063	4.06	1.0432	1.49	5.65	6.71	7.83
664	TRUE	Empty	Glutathione reductase, mitochondrial OS=Homo sapiens GN=GSR PE=1 SV=2	GSHR_HUMAN	?		0.680743732	0.8	0	28.166	17.954	11.178	12.707	11.277

665	TRUE	Empty	Glutathione S-transferase kappa 1 OS=Homo sapiens GN=GSTK1 PE=1 SV=3	GSTK1_HUMAN	?		0.000114504	5.077196078	0.99746	0	0	1.04	1.43	1.96
666	TRUE	Empty	Glutathione S-transferase Mu 3 OS=Homo sapiens GN=GSTM3 PE=1 SV=3	GSTM3_HUMAN	27 kDa	TRUE	0.66798007	1.4	24.314	2.0863	1.49	11.923	12.707	14.097
667	TRUE	Empty	Glutathione S-transferase omega-1 OS=Homo sapiens GN=GSTO1 PE=1 SV=2	GSTO1_HUMAN	?	TRUE	.567429604	0.7	2.03	11.475	22.942	5.65	8.13	11.277
668	TRUE	Empty	Glutathione S-transferase theta-1 OS=Homo sapiens GN=GSTT1 PE=1 SV=4	GSTT1_HUMAN	?		0.116315977	0	0	2.0863	1.49	0	0	0
669	TRUE	Empty	Glutathione synthetase OS=Homo sapiens GN=GSS PE=1 SV=1	GSHB_HUMAN	?		0.065597725	0.4	6.631	9.86	13.964	2.57	5.99	3.91
670	TRUE	Empty	Glyceraldehyde-3-phosphate dehydrogenase OS=Homo sapiens GN=GAPDH PE=1 SV=3	G3P_HUMAN	?	TRUE	.362040537	0.5	243.14	1 000.4	439.88	277.22	282.1	210.51
671	TRUE	Empty	Glycerol-3-phosphate dehydrogenase 1-like protein OS=Homo sapiens GN=GPD1L PE=1 SV=1	GPD1L_HUMAN	38 kDa	TRUE	.187802093	3.2	0	0	5.47	8.74	2.14	8.81
672	TRUE	Empty	Glycerol-3-phosphate dehydrogenase, mitochondrial OS=Homo sapiens GN=GPD2 PE=1 SV=3	GPDM_HUMAN	?		0.014880523	9.299142022	0	2.0863	0	6.707	4.57	8.81
673	TRUE	Empty	Glycerophosphodiester phosphodiesterase domain-containing protein 3 OS=Homo sapiens GN=GDPD3 PE=2 SV=3	GDPD3_HUMAN	?		0.121391969	INF	0	0	0	2.09	0	3.91
674	TRUE	Empty	Glycine--tRNA ligase OS=Homo sapiens GN=GARS PE=1 SV=3	SYG_HUMAN	83 kDa	TRUE	.183523879	0.6	15.472	38.597	29.924	9.78	18.637	19.735
675	TRUE	Empty	Glycogen phosphorylase, brain form OS=Homo sapiens GN=PYGB PE=1 SV=5	PYGB_HUMAN	97 kDa	TRUE	.007375351	4.946791833	4.06	3.1295	0.99746	13.414	11.013	17.856
676	TRUE	Empty	Glycogenin-1 OS=Homo sapiens GN=GYG1 PE=1 SV=4	GLYG_HUMAN	?		0.352779877	0.6	4.06	7.22	5.47	0.74522	2.14	7.83
677	TRUE	Empty	Glyoxalase domain-containing protein 4 OS=Homo sapiens GN=GLOD4 PE=1 SV=1	GLOD4_HUMAN	?		0.173004776	2.8	0	7.22	6.22	8.74	9.85	22.555
678	TRUE	Empty	Glyoxylate reductase/hydroxypyruvate reductase OS=Homo sapiens GN=GRHPR PE=1 SV=1	GRHPR_HUMAN	?	TRUE	.483720568	1.4	0	11.475	5.47	8.74	8.13	8.81
679	TRUE	Empty	GMP synthase [glutamine-hydrolyzing] OS=Homo sapiens GN=GMPS PE=1 SV=1	GUAA_HUMAN	?	TRUE	.084528058	9.4	0	1.0432	1.49	6.707	5.0828	16.916
680	TRUE	Empty	GOGA4_HUMAN-DECOY	GOGA4_HUMAN-DE	?	TRUE	.243940672	0	4.06	1.0432	0	0	0	0
681	TRUE	Empty	Golgi apparatus protein 1 OS=Homo sapiens GN=GLG1 PE=1 SV=2	GSLG1_HUMAN	?	TRUE	0.10854527	5.9	2.03	0	0	2.09	2.14	7.83
682	TRUE	Empty	Golgi phosphoprotein 3 OS=Homo sapiens GN=GOLPH3 PE=1 SV=1	GOLP3_HUMAN	34 kDa	TRUE	.013822492	2.053311993	4.06	2.0863	3.98	8.74	6.71	6.85
683	TRUE	Empty	Golgi reassembly-stacking protein 2 OS=Homo sapiens GN=GORASP2 PE=1 SV=3	GORS2_HUMAN	?		0.736444178	0.9	8.13	3.1295	2.24	2.09	5.0828	4.89
684	TRUE	Empty	Golgi resident protein GCP60 OS=Homo sapiens GN=ACBD3 PE=1 SV=4	GCP60_HUMAN	61 kDa	TRUE	.178702406	4.8	0	0	0.99746	2.09	0.84713	0.93978
685	TRUE	Empty	Golgin subfamily A member 3 OS=Homo sapiens GN=GOLGA3 PE=1 SV=2	GOGA3_HUMAN	?	TRUE	.491737711	0.6	0	2.0863	1.49	0.74522	0.84713	0.93978
686	TRUE	Empty	Golgin subfamily A member 4 OS=Homo sapiens GN=GOLGA4 PE=1 SV=1	GOGA4_HUMAN	?	TRUE	.373900966	0	0	0	2.24	0	0	0
687	TRUE	Empty	Grancalcin OS=Homo sapiens GN=GCA PE=1 SV=2	GRAN_HUMAN	24 kDa		0.017663121	3.387905279	0.99746	0	0	0.74522	1.43	0.93978
688	TRUE	Empty	G-rich sequence factor 1 OS=Homo sapiens GN=GRSF1 PE=1 SV=3	GRSF1_HUMAN	?		0.001071009	8.465101357	0	0.99746	0	2.57	3.85	2.94
689	TRUE	Empty	Growth factor receptor-bound protein 2 OS=Homo sapiens GN=GRB2 PE=1 SV=1	GRB2_HUMAN	?		0.792670078	0.8	0	1.0432	2.24	1.04	0.84713	0.93978
690	TRUE	Empty	GTPase NRas OS=Homo sapiens GN=NRAS PE=1 SV=1	RASN_HUMAN	21 kDa	TRUE	.731633154	0.7	0	1.0432	3.98	0	2.14	0.93978
691	TRUE	Empty	GTPase-activating protein and VPS9 domain-containing protein 1 OS=Homo sapiens GN=GAPVD1 PE=1 SV=2	GAPD1_HUMAN	?		0.02329226	12.87871193	0.99746	0	0	3.61	2.14	6.85
692	TRUE	Empty	GTP-binding nuclear protein Ran OS=Homo sapiens GN=RAN PE=1 SV=3	RAN_HUMAN	24 kDa		0.209114757	1.2	30.944	41.727	35.908	48.439	38.968	40.411

693	TRUE	Empty	GTP-binding protein Rheb OS=Homo sapiens GN=RHEB PE=1 SV=1	RHEB_HUMAN	20 kDa		0.373900966	INF	0	0	0	0	1.43	0
694	TRUE	Empty	GTP-binding protein SAR1b OS=Homo sapiens GN=SAR1B PE=1 SV=1	SAR1B_HUMAN	22 kDa	TRUE	.408813086	1.7	2.03	0	3.98	1.04	4.57	4.89
695	TRUE	Empty	Guanine nucleotide exchange factor VAV2 OS=Homo sapiens GN=VAV2 PE=1 SV=2	VAV2_HUMAN	?		0.355492035	7.7	0	0	0.99746	0	6.71	0.93978
696	TRUE	Empty	Guanine nucleotide-binding protein G(I)/G(S)/G(T) subunit beta-1 OS=Homo sapiens GN=GNB1 PE=1 SV=3	GBB1_HUMAN	?	TRUE	.911579007	1.1	2.03	14.604	9.46	7.22	7.42	13.157
697	TRUE	Empty	Guanine nucleotide-binding protein G(I)/G(S)/G(T) subunit beta-2 OS=Homo sapiens GN=GNB2 PE=1 SV=3	GBB2_HUMAN	?	TRUE	.866332962	0.9	2.03	16.691	7.97	6.707	8.13	9.78
698	TRUE	Empty	Guanine nucleotide-binding protein G(k) subunit alpha OS=Homo sapiens GN=GNAI3 PE=1 SV=3	GNAI3_HUMAN	41 kDa	TRUE	0.33908911	2.4	6.631	0	0.99746	11.178	2.14	4.89
699	TRUE	Empty	Guanine nucleotide-binding protein G(s) subunit alpha isoforms XLas OS=Homo sapiens GN=GNAS PE=1 SV=2	GNAS1_HUMAN	?	TRUE	.009839231	7.420722843	0	0	1.49	5.17	5.0828	3.91
700	TRUE	Empty	Guanine nucleotide-binding protein subunit alpha-13 OS=Homo sapiens GN=GNA13 PE=1 SV=2	GNA13_HUMAN	?	TRUE	.004654004	13.5328735	0	0	0.99746	4.13	3.85	5.87
701	TRUE	Empty	Guanine nucleotide-binding protein subunit beta-2-like 1 OS=Homo sapiens GN=GNB2L1 PE=1 SV=3	GBLP_HUMAN	35 kDa		0.036981082	1.907841342	19.893	12.518	9.46	30.554	21.178	29.133
702	TRUE	Empty	Guanine nucleotide-binding protein-like 1 OS=Homo sapiens GN=GNL1 PE=1 SV=2	GNL1_HUMAN	?		0.06853503	INF	0	0	0	0.74522	2.14	0.93978
703	TRUE	Empty	H/ACA ribonucleoprotein complex subunit 2 OS=Homo sapiens GN=NHP2 PE=1 SV=1	NHP2_HUMAN	17 kDa		0.200796519	0.5	4.06	10.432	5.47	3.61	1.43	5.87
704	TRUE	Empty	H/ACA ribonucleoprotein complex subunit 3 OS=Homo sapiens GN=NOP10 PE=1 SV=1	NOP10_HUMAN	8 kDa		0.896631565	1.2	0	2.0863	0	0.74522	1.43	0
705	TRUE	Empty	H/ACA ribonucleoprotein complex subunit 4 OS=Homo sapiens GN=DKC1 PE=1 SV=3	DKC1_HUMAN	?		0.428752659	1.3	4.06	2.0863	3.98	4.13	6.71	2.94
706	TRUE	Empty	HD domain-containing protein 2 OS=Homo sapiens GN=HDDC2 PE=1 SV=1	HDDC2_HUMAN	?	TRUE	.231724225	0.1	0	2.0863	4.73	0	0.84713	0
707	TRUE	Empty	HEAT repeat-containing protein 6 OS=Homo sapiens GN=HEATR6 PE=1 SV=1	HEAT6_HUMAN	129 kDa		0.007928191	4.529039955	6.631	2.0863	0	11.923	14.401	13.157
708	TRUE	Empty	Heat shock 70 kDa protein 14 OS=Homo sapiens GN=HSPA14 PE=1 SV=1	HSP7E_HUMAN	55 kDa		0.91179265	1.1	4.06	0	0	2.57	0.84713	1.96
709	TRUE	Empty	Heat shock 70 kDa protein 1B OS=Homo sapiens GN=HSPA1B PE=1 SV=1	HS71B_HUMAN (+1)	70 kDa	TRUE	.052352551	0.4	156.93	332.77	270.31	118.49	117.75	98.677
710	TRUE	Empty	Heat shock 70 kDa protein 4 OS=Homo sapiens GN=HSPA4 PE=1 SV=4	HSP74_HUMAN	?	TRUE	.009111815	3.658037131	6.631	3.1295	4.73	14.159	22.873	16.916
711	TRUE	Empty	Heat shock cognate 71 kDa protein OS=Homo sapiens GN=HSPA8 PE=1 SV=1	HSP7C_HUMAN	?	TRUE	.090292806	0.6	179.04	327.56	296.24	183.32	155.03	156.94
712	TRUE	Empty	Heat shock factor-binding protein 1 OS=Homo sapiens GN=HSBP1 PE=1 SV=1	HSBP1_HUMAN	9 kDa		0.870026374	0.9	0	4.1727	10.972	5.65	2.14	5.87
713	TRUE	Empty	Heat shock protein 105 kDa OS=Homo sapiens GN=HSPH1 PE=1 SV=1	HS105_HUMAN	?	TRUE	.004927414	5.901646305	2.03	5.59	1.49	18.63	22.873	14.097
714	TRUE	Empty	Heat shock protein 75 kDa, mitochondrial OS=Homo sapiens GN=TRAP1 PE=1 SV=3	TRAP1_HUMAN	?	TRUE	.760794718	1.3	46.417	1.0432	0	15.65	25.414	21.615
715	TRUE	Empty	Heat shock protein beta-1 OS=Homo sapiens GN=HSPB1 PE=1 SV=2	HSPB1_HUMAN	23 kDa	TRUE	.003991209	0.244920858	176.83	264.97	211.46	68.56	57.605	33.832
716	TRUE	Empty	Heat shock protein HSP 90-alpha OS=Homo sapiens GN=HSP90AA1 PE=1 SV=5	HS90A_HUMAN	?	TRUE	.691283762	1.2	349.23	56.331	139.64	279.46	168.58	216.15
717	TRUE	Empty	Heat shock protein HSP 90-beta OS=Homo sapiens GN=HSP90AB1 PE=1 SV=4	HS90B_HUMAN	83 kDa	TRUE	.537483646	1.3	265.24	37.554	108.72	221.33	155.87	175.74
718	TRUE	Empty	Heat shock-related 70 kDa protein 2 OS=Homo sapiens GN=HSPA2 PE=1 SV=1	HSP72_HUMAN	70 kDa	TRUE	.014238855	0.442871496	90.623	145	119.69	47.694	54.217	55.447

719	TRUE	Empty	Hematological and neurological expressed 1 protein OS=Homo sapiens GN=HN1 PE=1 SV=3	HN1_HUMAN	?		0.247173027	0.3	0	11.475	12.967	3.61	3.85	0
720	TRUE	Empty	Hematological and neurological expressed 1-like protein OS=Homo sapiens GN=HN1L PE=1 SV=1	HN1L_HUMAN	?		0.21281136	0.2	0	8.54	8.71	0.74522	2.14	0.93978
721	TRUE	Empty	Heme-binding protein 1 OS=Homo sapiens GN=HEBP1 PE=1 SV=1	HEBP1_HUMAN	21 kDa		0.562574355	0.6	0	5.59	5.47	4.13	1.43	0.93978
722	TRUE	Empty	Heme-binding protein 2 OS=Homo sapiens GN=HEBP2 PE=1 SV=1	HEBP2_HUMAN	?	TRUE	.613850651	0.7	0	9.86	21.944	8.74	5.0828	7.83
723	TRUE	Empty	Hemoglobin subunit alpha OS=Homo sapiens GN=HBA1 PE=1 SV=2	HBA_HUMAN	15 kDa	TRUE	.727941284	0.7	0	11.475	35.908	8.26	9.85	16.916
724	TRUE	Empty	Hemoglobin subunit beta OS=Homo sapiens GN=HBB PE=1 SV=2	HBB_HUMAN	16 kDa	TRUE	.158199504	0.4	6.631	17.734	19.949	5.65	2.14	11.277
725	TRUE	Empty	Hepatocyte growth factor-regulated tyrosine kinase substrate OS=Homo sapiens GN=HGS PE=1 SV=1	HGS_HUMAN	?	TRUE	.639358056	1.7	0	1.0432	1.49	0	4.57	0.93978
726	TRUE	Empty	Hepatocyte nuclear factor 3-alpha OS=Homo sapiens GN=FOXA1 PE=1 SV=2	FOXA1_HUMAN	?	TRUE	.022343681	5.926453191	0	0	0.99746	1.04	2.14	1.96
727	TRUE	Empty	Hepatoma-derived growth factor OS=Homo sapiens GN=HDGF PE=1 SV=1	HDGF_HUMAN	?	TRUE	0.80255124	1.2	0	8.54	15.959	18.63	4.57	6.85
728	TRUE	Empty	Hepatoma-derived growth factor-related protein 2 OS=Homo sapiens GN=HDGFRP2 PE=1 SV=1	HDGR2_HUMAN	?	TRUE	.741983428	1.3	0	2.0863	1.49	0.74522	0.84713	3.91
729	TRUE	Empty	Heterogeneous nuclear ribonucleoprotein A/B OS=Homo sapiens GN=HNRNPAB PE=1 SV=2	ROAA_HUMAN	?	TRUE	.455741301	0.7	8.13	44.856	33.914	14.904	22.025	23.495
730	TRUE	Empty	Heterogeneous nuclear ribonucleoprotein A0 OS=Homo sapiens GN=HNRNPA0 PE=1 SV=1	ROA0_HUMAN	31 kDa	TRUE	.051228521	0.2	17.683	8.54	8.71	2.57	5.0828	0
731	TRUE	Empty	Heterogeneous nuclear ribonucleoprotein A1 OS=Homo sapiens GN=HNRNPA1 PE=1 SV=5	ROA1_HUMAN	?	TRUE	0.01311536	0.27783188	68.52	59.461	84.784	17.14	37.274	4.89
732	TRUE	Empty	Heterogeneous nuclear ribonucleoprotein A3 OS=Homo sapiens GN=HNRNPA3 PE=1 SV=2	ROA3_HUMAN	?	TRUE	.107699507	0.5	30.944	73.022	73.812	32.79	31.344	24.434
733	TRUE	Empty	Heterogeneous nuclear ribonucleoprotein D0 OS=Homo sapiens GN=HNRNPD PE=1 SV=1	HNRPD_HUMAN	?	TRUE	.708232672	0.8	8.13	35.468	44.886	21.611	33.038	20.675
734	TRUE	Empty	Heterogeneous nuclear ribonucleoprotein D-like OS=Homo sapiens GN=HNRNPDL PE=1 SV=3	HNRDL_HUMAN	?	TRUE	.962853544	1	2.03	14.604	19.949	6.707	16.943	12.217
735	TRUE	Empty	Heterogeneous nuclear ribonucleoprotein F OS=Homo sapiens GN=HNRNPF PE=1 SV=3	HNRPF_HUMAN	46 kDa	TRUE	.531241972	0.7	19.893	41.727	51.868	49.93	18.637	15.037
736	TRUE	Empty	Heterogeneous nuclear ribonucleoprotein H OS=Homo sapiens GN=HNRNPH1 PE=1 SV=4	HNRH1_HUMAN	49 kDa	TRUE	.616450921	0.8	33.155	53.202	68.825	76.757	17.79	26.314
737	TRUE	Empty	Heterogeneous nuclear ribonucleoprotein H2 OS=Homo sapiens GN=HNRNPH2 PE=1 SV=1	HNRH2_HUMAN	49 kDa	TRUE	.937795618	1	26.524	30.252	43.888	72.286	14.401	18.796
738	TRUE	Empty	Heterogeneous nuclear ribonucleoprotein H3 OS=Homo sapiens GN=HNRNPH3 PE=1 SV=2	HNRH3_HUMAN	?	TRUE	.428747465	0.8	13.262	6.259	10.972	9.78	4.57	9.78
739	TRUE	Empty	Heterogeneous nuclear ribonucleoprotein K OS=Homo sapiens GN=HNRNPK PE=1 SV=1	HNRPK_HUMAN	?	TRUE	.041418424	0.658267622	110.52	100.14	89.771	64.089	83.866	49.809
740	TRUE	Empty	Heterogeneous nuclear ribonucleoprotein L OS=Homo sapiens GN=HNRNPL PE=1 SV=2	HNRPL_HUMAN	?	TRUE	.374659478	0.5	0	58.418	36.906	16.395	12.707	15.037
741	TRUE	Empty	Heterogeneous nuclear ribonucleoprotein L-like OS=Homo sapiens GN=HNRNPLL PE=1 SV=1	HNRLL_HUMAN	?	TRUE	.121394667	INF	0	0	0	1.04	0	1.96
742	TRUE	Empty	Heterogeneous nuclear ribonucleoprotein M OS=Homo sapiens GN=HNRNPM PE=1 SV=3	HNRPM_HUMAN	?	TRUE	.052505918	0.6	81.782	94.929	95.756	36.516	73.701	63.905
743	TRUE	Empty	Heterogeneous nuclear ribonucleoprotein Q OS=Homo sapiens GN=SYNCRIP PE=1 SV=2	HNRPQ_HUMAN	?	TRUE	.504097358	0.8	44.206	21.907	29.924	20.121	28.803	31.013
744	TRUE	Empty	Heterogeneous nuclear ribonucleoprotein R OS=Homo sapiens GN=HNRNPR PE=1 SV=1	HNRPR_HUMAN	?	TRUE	.318853558	0.8	22.103	15.648	21.944	9.78	18.637	18.796

745	TRUE	Empty	Heterogeneous nuclear ribonucleoprotein U OS=Homo sapiens GN=HNRNPU PE=1 SV=6	HNRPU_HUMAN	?	TRUE	.814229947	0.9	37.575	12.518	49.873	39.497	27.955	23.495
746	TRUE	Empty	Heterogeneous nuclear ribonucleoprotein U-like protein 1 OS=Homo sapiens GN=HNRNPUL1 PE=1 SV=2	HNRL1_HUMAN	?	TRUE	.024169728	6.268346478	0	0	2.24	5.65	5.0828	8.81
747	TRUE	Empty	Heterogeneous nuclear ribonucleoprotein U-like protein 2 OS=Homo sapiens GN=HNRNPUL2 PE=1 SV=1	HNRL2_HUMAN	85 kDa	TRUE	.543956482	1.3	4.06	18.777	15.959	15.65	12.707	20.675
748	TRUE	Empty	Heterogeneous nuclear ribonucleoproteins A2/B1 OS=Homo sapiens GN=HNRNPA2B1 PE=1 SV=2	ROA2_HUMAN	?	TRUE	.067278605	0.6	66.31	105.36	80.794	64.089	45.745	30.073
749	TRUE	Empty	Heterogeneous nuclear ribonucleoproteins C1/C2 OS=Homo sapiens GN=HNRNPC PE=1 SV=4	HNRPC_HUMAN	?	TRUE	.303197603	0.8	39.786	55.288	63.837	44.713	46.592	42.29
750	TRUE	Empty	Heterochromatin protein 1-binding protein 3 OS=Homo sapiens GN=HP1BP3 PE=1 SV=1	HP1B3_HUMAN	?	TRUE	0.17096993	2.5	0	1.0432	0.99746	0.74522	2.14	1.96
751	TRUE	Empty	Hexokinase-1 OS=Homo sapiens GN=HK1 PE=1 SV=3	HXK1_HUMAN	?	TRUE	.198966639	2.6	19.893	0	0.99746	11.923	20.331	21.615
752	TRUE	Empty	High mobility group protein B1 OS=Homo sapiens GN=HMGB1 PE=1 SV=3	HMGB1_HUMAN	25 kDa	TRUE	.487911839	0.7	41.996	166.91	115.71	90.171	74.548	76.122
753	TRUE	Empty	High mobility group protein B2 OS=Homo sapiens GN=HMGB2 PE=1 SV=2	HMGB2_HUMAN	24 kDa	TRUE	.845973139	1.1	13.262	36.511	48.875	32.044	35.58	37.591
754	TRUE	Empty	High mobility group protein B3 OS=Homo sapiens GN=HMGB3 PE=1 SV=4	HMGB3_HUMAN	23 kDa	TRUE	.978962266	1	6.631	23.993	27.929	16.395	23.72	17.856
755	TRUE	Empty	Hippocalcin-like protein 1 OS=Homo sapiens GN=HPCAL1 PE=1 SV=3	HPCL1_HUMAN	22 kDa	TRUE	.146055018	3.4	0	0	0.99746	0.74522	1.43	0.93978
756	TRUE	Empty	Histidine triad nucleotide-binding protein 1 OS=Homo sapiens GN=HINT1 PE=1 SV=2	HINT1_HUMAN	14 kDa		0.5255283	0.6	0	8.54	9.46	5.65	0	5.87
757	TRUE	Empty	Histidine triad nucleotide-binding protein 2, mitochondrial OS=Homo sapiens GN=HINT2 PE=1 SV=1	HINT2_HUMAN	17 kDa		0.782678988	0.7	0	2.0863	0.99746	2.57	0	0
758	TRUE	Empty	Histidine--tRNA ligase, cytoplasmic OS=Homo sapiens GN=HARS PE=1 SV=2	SYHC_HUMAN	?	TRUE	.841255959	0.8	6.631	0	1.49	3.61	1.43	1.96
759	TRUE	Empty	Histone acetyltransferase type B catalytic subunit OS=Homo sapiens GN=HAT1 PE=1 SV=1	HAT1_HUMAN	?	TRUE	.121394667	INF	0	0	0	1.04	0	1.96
760	TRUE	Empty	Histone deacetylase 1 OS=Homo sapiens GN=HDAC1 PE=1 SV=1	HDAC1_HUMAN	55 kDa	TRUE	0.13481951	3.5	2.03	0	3.98	2.57	9.85	10.338
761	TRUE	Empty	Histone deacetylase 2 OS=Homo sapiens GN=HDAC2 PE=1 SV=2	HDAC2_HUMAN	?	TRUE	.275439385	2.2	8.13	3.1295	0.99746	3.61	15.248	9.78
762	TRUE	Empty	Histone H1.2 OS=Homo sapiens GN=HIST1H1C PE=1 SV=2	H12_HUMAN	21 kDa	TRUE	.549022903	0.5	22.103	2.0863	3.98	6.707	5.99	2.94
763	TRUE	Empty	Histone H1.5 OS=Homo sapiens GN=HIST1H1B PE=1 SV=3	H15_HUMAN	23 kDa	TRUE	.555089633	0.4	22.103	0	0.99746	2.09	3.85	2.94
764	TRUE	Empty	Histone H1x OS=Homo sapiens GN=H1FX PE=1 SV=1	H1X_HUMAN	22 kDa		0.373900966	0	0	0	2.24	0	0	0
765	TRUE	Empty	Histone H2A type 2-C OS=Homo sapiens GN=HIST2H2AC PE=1 SV=4	H2A2C_HUMAN	14 kDa	TRUE	.390882903	0.4	475.22	60.504	99.746	65.579	119.45	65.785
766	TRUE	Empty	Histone H2A.V OS=Homo sapiens GN=H2AFV PE=1 SV=3	H2AV_HUMAN	?	TRUE	.611010306	1.3	6.631	7.22	23.939	9.78	23.72	15.976
767	TRUE	Empty	Histone H2B type 1-C/E/F/G/I OS=Homo sapiens GN=HIST1H2BC PE=1 SV=4	H2B1C_HUMAN	14 kDa	TRUE	.326937254	0.7	327.13	156.48	246.37	174.38	242.28	95.858
768	TRUE	Empty	Histone H2B type 2-E OS=Homo sapiens GN=HIST2H2BE PE=1 SV=3	H2B2E_HUMAN	14 kDa	TRUE	.249702531	0.6	322.71	153.35	234.4	166.93	212.63	73.303
769	TRUE	Empty	Histone H4 OS=Homo sapiens GN=HIST1H4A PE=1 SV=2	H4_HUMAN	11 kDa		0.295929165	2	70.73	3.1295	6.22	49.184	65.229	47.929
770	TRUE	Empty	Histone-arginine methyltransferase CARM1 OS=Homo sapiens GN=CARM1 PE=1 SV=3	CARM1_HUMAN	?		0.189953018	INF	0	0	0	2.57	0.84713	0
771	TRUE	Empty	Histone-binding protein RBBP4 OS=Homo sapiens GN=RBBP4 PE=1 SV=3	RBBP4_HUMAN	?	TRUE	.212239959	1.9	0	4.1727	8.71	7.22	7.42	10.338
772	TRUE	Empty	Histone-binding protein RBBP7 OS=Homo sapiens GN=RBBP7 PE=1 SV=1	RBBP7_HUMAN	?	TRUE	.334575215	1.7	0	4.1727	11.969	8.74	9.85	10.338
773	TRUE	Empty	Histone-lysine N-methyltransferase SETD7 OS=Homo sapiens GN=SETD7 PE=1 SV=1	SETD7_HUMAN	41 kDa		0.225136446	0.1	0	4.1727	1.49	0	0.84713	0

774	TRUE	Empty	HIV Tat-specific factor 1 OS=Homo sapiens GN=HTATSF1 PE=1 SV=1	HTSF1_HUMAN	86 kDa		0.068186251	5	0	0	0.99746	2.57	0.84713	1.96
775	TRUE	Empty	Host cell factor 1 OS=Homo sapiens GN=HCFC1 PE=1 SV=2	HCFC1_HUMAN	?		0.946462977	1	0	7.22	8.71	4.13	6.71	5.87
776	TRUE	Empty	Hsc70-interacting protein OS=Homo sapiens GN=ST13 PE=1 SV=2	F10A1_HUMAN	41 kDa	TRUE	.745969406	1.1	2.03	4.1727	5.47	5.17	5.0828	2.94
777	TRUE	Empty	Hsp70-binding protein 1 OS=Homo sapiens GN=HSPBP1 PE=1 SV=1	HPBP1_HUMAN	?		0.009521936	4.227868787	0	0.99746	0	1.04	0.84713	1.96
778	TRUE	Empty	Hsp90 co-chaperone Cdc37 OS=Homo sapiens GN=CDC37 PE=1 SV=1	CDC37_HUMAN	44 kDa	TRUE	.581189718	0.5	2.03	0	11.969	3.61	0.84713	2.94
779	TRUE	Empty	Huntingtin OS=Homo sapiens GN=HTT PE=1 SV=2	HD_HUMAN	348 kDa	TRUE	.145333264	INF	0	0	0	0.74522	0.84713	3.91
780	TRUE	Empty	Huntingtin-interacting protein 1-related protein OS=Homo sapiens GN=HIP1R PE=1 SV=2	HIP1R_HUMAN	?	TRUE	.014880189	4.330118501	0	0.99746	0	0.74522	1.43	1.96
781	TRUE	Empty	Hydroxyacyl-coenzyme A dehydrogenase, mitochondrial OS=Homo sapiens GN=HADH PE=1 SV=3	HCDH_HUMAN	?		0.775877095	0.7	0	1.0432	0.99746	1.04	0	0
782	TRUE	Empty	Hydroxyacylglutathione hydrolase, mitochondrial OS=Homo sapiens GN=HAGH PE=1 SV=2	GLO2_HUMAN	?		0.575126505	0.6	0	3.1295	5.47	3.61	0.84713	0.93978
783	TRUE	Empty	Hydroxymethylglutaryl-CoA lyase, mitochondrial OS=Homo sapiens GN=HMGCL PE=1 SV=2	HMGCL_HUMAN	?		0.231711402	INF	0	0	0	0.74522	0	2.94
784	TRUE	Empty	Hydroxymethylglutaryl-CoA synthase, cytoplasmic OS=Homo sapiens GN=HMGCS1 PE=1 SV=2	HMCS1_HUMAN	57 kDa	TRUE	.007459224	4.986189983	0	4.1727	0.99746	8.26	9.85	7.83
785	TRUE	Empty	Hydroxysteroid dehydrogenase-like protein 2 OS=Homo sapiens GN=HSDL2 PE=1 SV=1	HSDL2_HUMAN	?		0.184606477	INF	0	0	0	0.74522	0	1.96
786	TRUE	Empty	Hypoxanthine-guanine phosphoribosyltransferase OS=Homo sapiens GN=HPRT1 PE=1 SV=2	HPRT_HUMAN	25 kDa	TRUE	.356492995	1.4	2.03	12.518	6.22	9.78	10.166	11.277
787	TRUE	Empty	Hypoxia up-regulated protein 1 OS=Homo sapiens GN=HYOU1 PE=1 SV=1	HYOU1_HUMAN	?	TRUE	.005591786	5.77791303	2.03	3.1295	0	9.78	12.707	8.81
788	TRUE	Empty	Charged multivesicular body protein 3 OS=Homo sapiens GN=CHMP3 PE=1 SV=3	CHMP3_HUMAN	?		0.84022402	1.2	0	1.0432	1.49	0	1.43	1.96
789	TRUE	Empty	Charged multivesicular body protein 4a OS=Homo sapiens GN=CHMP4A PE=1 SV=3	CHM4A_HUMAN	?		0.593553623	0.6	0	1.0432	2.24	1.04	0.84713	0
790	TRUE	Empty	Charged multivesicular body protein 4b OS=Homo sapiens GN=CHMP4B PE=1 SV=1	CHM4B_HUMAN	25 kDa	TRUE	.404244787	0.4	0	3.1295	5.47	2.57	1.43	0
791	TRUE	Empty	Chloride intracellular channel protein 1 OS=Homo sapiens GN=CLIC1 PE=1 SV=4	CLIC1_HUMAN	27 kDa		0.010303208	0.721061032	19.893	22.95	22.942	16.395	16.943	14.097
792	TRUE	Empty	Chloride intracellular channel protein 3 OS=Homo sapiens GN=CLIC3 PE=1 SV=2	CLIC3_HUMAN	27 kDa	TRUE	.195440388	0.2	0	4.1727	5.47	0	0.84713	0.93978
793	TRUE	Empty	Chloride intracellular channel protein 4 OS=Homo sapiens GN=CLIC4 PE=1 SV=4	CLIC4_HUMAN	29 kDa		0.915011943	1.1	0	3.1295	1.49	3.61	0	1.96
794	TRUE	Empty	Choline transporter-like protein 1 OS=Homo sapiens GN=SLC44A1 PE=1 SV=1	CTL1_HUMAN	?		0.096931939	8.9	0	0	0.99746	0.74522	3.85	4.89
795	TRUE	Empty	Choline transporter-like protein 2 OS=Homo sapiens GN=SLC44A2 PE=1 SV=3	CTL2_HUMAN	?		0.5751267	0.5	0	2.0863	1.49	2.57	0	0
796	TRUE	Empty	Choline-phosphate cytidylyltransferase A OS=Homo sapiens GN=PCYT1A PE=1 SV=2	PCY1A_HUMAN	42 kDa	TRUE	.928726266	0.9	0	3.1295	1.49	2.57	2.14	0
797	TRUE	Empty	Chromatin complexes subunit BAP18 OS=Homo sapiens GN=BAP18 PE=1 SV=1	BAP18_HUMAN	?		0.165289766	0.1	0	10.432	8.71	0	1.43	0.93978
798	TRUE	Empty	Chromobox protein homolog 1 OS=Homo sapiens GN=CBX1 PE=1 SV=1	CBX1_HUMAN	21 kDa	TRUE	.081078498	0.6	11.052	14.604	15.959	11.178	8.13	3.91
799	TRUE	Empty	Chromobox protein homolog 3 OS=Homo sapiens GN=CBX3 PE=1 SV=4	CBX3_HUMAN	21 kDa	TRUE	.544570353	0.8	26.524	15.648	14.962	20.121	16.096	12.217
800	TRUE	Empty	Chromobox protein homolog 5 OS=Homo sapiens GN=CBX5 PE=1 SV=1	CBX5_HUMAN	22 kDa	TRUE	.837586551	1.1	6.631	0	9.46	8.26	5.0828	4.89

801	TRUE	Empty	Chromodomain-helicase-DNA-binding protein 4 OS=Homo sapiens GN=CHD4 PE=1 SV=2	CHD4_HUMAN	?	TRUE	.045359676	3.254356725	4.06	0	0.99746	5.65	6.71	5.87
802	TRUE	Empty	Immunity-related GTPase family Q protein OS=Homo sapiens GN=IRGQ PE=1 SV=1	IRGQ_HUMAN	63 kDa	TRUE	0.27968692	8.6	0	0	0.99746	6.707	0	1.96
803	TRUE	Empty	Importin subunit alpha-1 OS=Homo sapiens GN=KPNA2 PE=1 SV=1	IMA1_HUMAN	58 kDa		0.099813876	3.4	8.13	1.0432	0.99746	12.669	6.71	17.856
804	TRUE	Empty	Importin subunit alpha-3 OS=Homo sapiens GN=KPNA4 PE=1 SV=1	IMA3_HUMAN	58 kDa	TRUE	0.05629162	2.6	2.03	0	3.98	5.65	5.99	4.89
805	TRUE	Empty	Importin subunit alpha-4 OS=Homo sapiens GN=KPNA3 PE=1 SV=2	IMA4_HUMAN	58 kDa	TRUE	.337175177	2.3	2.03	0	0	0.74522	2.14	1.96
806	TRUE	Empty	Importin subunit alpha-7 OS=Homo sapiens GN=KPNA6 PE=1 SV=1	IMA7_HUMAN	60 kDa	TRUE	.005690322	20.05262653	0	1.0432	0	5.17	9.85	5.87
807	TRUE	Empty	Importin subunit beta-1 OS=Homo sapiens GN=KPNB1 PE=1 SV=2	IMB1_HUMAN	?		0.128888016	2.2	35.365	6.259	6.22	36.516	38.968	30.073
808	TRUE	Empty	Importin-4 OS=Homo sapiens GN=IPO4 PE=1 SV=2	IPO4_HUMAN	?	TRUE	0.08007674	3.6	17.683	0	1.49	14.159	28.803	28.194
809	TRUE	Empty	Importin-5 OS=Homo sapiens GN=IPO5 PE=1 SV=4	IPO5_HUMAN	?	TRUE	.014237465	21.20836926	0.99746	0	0	10.433	5.0828	5.87
810	TRUE	Empty	Importin-7 OS=Homo sapiens GN=IPO7 PE=1 SV=1	IPO7_HUMAN	120 kDa	TRUE	.028238364	6.791046464	4.06	0	0	7.22	8.13	14.097
811	TRUE	Empty	Importin-9 OS=Homo sapiens GN=IPO9 PE=1 SV=3	IPO9_HUMAN	116 kDa		0.144191754	INF	0	0	0	0.74522	5.99	1.96
812	TRUE	Empty	Inorganic pyrophosphatase 2, mitochondrial OS=Homo sapiens GN=PPA2 PE=1 SV=2	IPYR2_HUMAN	?		0.212298995	2.2	0	5.59	2.24	2.09	8.13	6.85
813	TRUE	Empty	Inorganic pyrophosphatase OS=Homo sapiens GN=PPA1 PE=1 SV=2	IPYR_HUMAN	33 kDa		0.919632846	1	6.631	23.993	25.934	17.885	16.943	19.735
814	TRUE	Empty	Inosine-5'-monophosphate dehydrogenase 2 OS=Homo sapiens GN=IMPDH2 PE=1 SV=2	IMDH2_HUMAN	56 kDa	TRUE	.097302296	0.5	8.13	18.777	12.967	6.707	7.42	7.83
815	TRUE	Empty	Insulin receptor substrate 1 OS=Homo sapiens GN=IRS1 PE=1 SV=1	IRS1_HUMAN	132 kDa		0.367102274	0.4	0	5.59	2.24	0.74522	0.84713	1.96
816	TRUE	Empty	Integrin alpha-2 OS=Homo sapiens GN=ITGA2 PE=1 SV=1	ITA2_HUMAN	129 kDa	TRUE	.080922552	INF	0	0	0	0.74522	1.43	3.91
817	TRUE	Empty	Integrin alpha-IIb OS=Homo sapiens GN=ITGA2B PE=1 SV=3	ITA2B_HUMAN	?		0.373900966	0	0	0	2.24	0	0	0
818	TRUE	Empty	Integrin beta-1 OS=Homo sapiens GN=ITGB1 PE=1 SV=2	ITB1_HUMAN	?		0.059060944	4.6	0	1.0432	0.99746	2.09	1.43	4.89
819	TRUE	Empty	Inter-alpha-trypsin inhibitor heavy chain H1 OS=Homo sapiens GN=ITIH1 PE=1 SV=3	ITIH1_HUMAN	?	TRUE	.373900966	0	0	0	2.24	0	0	0
820	TRUE	Empty	Inter-alpha-trypsin inhibitor heavy chain H2 OS=Homo sapiens GN=ITIH2 PE=1 SV=2	ITIH2_HUMAN	106 kDa		0.845466072	0.9	0	4.1727	6.22	1.04	2.14	5.87
821	TRUE	Empty	Interferon regulatory factor 2-binding protein 1 OS=Homo sapiens GN=IRF2BP1 PE=1 SV=1	I2BP1_HUMAN	62 kDa	TRUE	.836221794	0.9	2.03	2.0863	1.49	2.57	0.84713	2.94
822	TRUE	Empty	Interferon regulatory factor 2-binding protein 2 OS=Homo sapiens GN=IRF2BP2 PE=1 SV=2	I2BP2_HUMAN	?	TRUE	.178737427	0.4	2.03	10.432	5.47	1.04	2.14	2.94
823	TRUE	Empty	Interferon regulatory factor 2-binding protein-like OS=Homo sapiens GN=IRF2BPL PE=1 SV=1	I2BPL_HUMAN	83 kDa	TRUE	.378886963	0.4	0	9.86	4.73	0.74522	2.14	2.94
824	TRUE	Empty	Interferon-induced, double-stranded RNA-activated protein kinase OS=Homo sapiens GN=EIF2AK2 PE=1 SV=2	E2AK2_HUMAN	?	TRUE	.076246162	7	2.03	0	0	5.17	7.42	1.96
825	TRUE	Empty	Interferon-inducible double-stranded RNA-dependent protein kinase activator A OS=Homo sapiens GN=PRKRA PE=1 SV=1	PRKRA_HUMAN	?		0.401672964	2	0	2.0863	0	1.04	0.84713	1.96
826	TRUE	Empty	Interleukin enhancer-binding factor 2 OS=Homo sapiens GN=ILF2 PE=1 SV=2	ILF2_HUMAN	43 kDa	TRUE	.041416164	1.608464038	8.13	13.561	11.969	21.611	18.637	15.037
827	TRUE	Empty	Interleukin enhancer-binding factor 3 OS=Homo sapiens GN=ILF3 PE=1 SV=3	ILF3_HUMAN	?	TRUE	0.28808888	1.3	19.893	11.475	23.939	26.083	21.178	22.555
828	TRUE	Empty	Intraflagellar transport protein 27 homolog OS=Homo sapiens GN=IFT27 PE=1 SV=1	IFT27_HUMAN	?		0.642647753	0.5	0	2.0863	0	0	0	0.93978
829	TRUE	Empty	Inverted formin-2 OS=Homo sapiens GN=INF2 PE=1 SV=2	INF2_HUMAN	?		0.627500576	1.5	6.631	0	0	2.09	3.85	3.91

830	TRUE	Empty	Isoamyl acetate-hydrolyzing esterase 1 homolog OS=Homo sapiens GN=IAH1 PE=1 SV=1	IAH1_HUMAN	?		0.432326179	0.5	0	7.22	4.73	0.74522	4.57	0.93978
831	TRUE	Empty	Isocitrate dehydrogenase [NAD] subunit alpha, mitochondrial OS=Homo sapiens GN=IDH3A PE=1 SV=1	IDH3A_HUMAN	?		0.00210632	7.720147533	2.03	1.0432	0	8.26	6.71	9.78
832	TRUE	Empty	Isocitrate dehydrogenase [NAD] subunit beta, mitochondrial OS=Homo sapiens GN=IDH3B PE=1 SV=2	IDH3B_HUMAN	?	TRUE	.117744445	INF	0	0	0	1.04	1.43	0
833	TRUE	Empty	Isocitrate dehydrogenase [NADP] cytoplasmic OS=Homo sapiens GN=IDH1 PE=1 SV=2	IDHC_HUMAN	47 kDa	TRUE	.546823868	1.4	2.03	14.604	6.22	17.14	8.13	7.83
834	TRUE	Empty	Isocitrate dehydrogenase [NADP], mitochondrial OS=Homo sapiens GN=IDH2 PE=1 SV=2	IDHP_HUMAN	?	TRUE	0.002494	3.315540516	8.13	6.259	3.98	19.376	19.484	24.434
835	TRUE	Empty	Isochorismatase domain-containing protein 1 OS=Homo sapiens GN=ISOC1 PE=1 SV=3	ISOC1_HUMAN	32 kDa		0.756007367	1.1	11.052	13.561	12.967	8.26	11.013	21.615
836	TRUE	Empty	Isochorismatase domain-containing protein 2, mitochondrial OS=Homo sapiens GN=ISOC2 PE=1 SV=1	ISOC2_HUMAN	?		0.458310893	2.3	0	0	0.99746	1.04	0.84713	0
837	TRUE	Empty	Isoleucine--tRNA ligase, cytoplasmic OS=Homo sapiens GN=IARS PE=1 SV=2	SYIC_HUMAN	145 kDa	TRUE	.001174808	14.12627245	2.03	0	0	9.78	9.85	12.217
838	TRUE	Empty	Isoleucine--tRNA ligase, mitochondrial OS=Homo sapiens GN=IARS2 PE=1 SV=2	SYIM_HUMAN	114 kDa	TRUE	.217218164	INF	0	0	0	0	0.84713	2.94
839	TRUE	Empty	Isopentenyl-diphosphate Delta-isomerase 1 OS=Homo sapiens GN=IDI1 PE=1 SV=2	IDI1_HUMAN	?		0.314718277	2.1	0	3.1295	0.99746	2.57	1.43	4.89
840	TRUE	Empty	Isovaleryl-CoA dehydrogenase, mitochondrial OS=Homo sapiens GN=IVD PE=1 SV=1	IVD_HUMAN	?		0.27968692	8.6	0	0	0.99746	6.707	0	1.96
841	TRUE	Empty	Junction plakoglobin OS=Homo sapiens GN=JUP PE=1 SV=3	PLAK_HUMAN	82 kDa	TRUE	.023146446	2.533366523	6.631	1.0432	5.47	10.433	11.013	13.157
842	TRUE	Empty	Kanadaptin OS=Homo sapiens GN=SLC4A1AP PE=1 SV=1	NADAP_HUMAN	89 kDa		0.146055018	3.4	0	0	0.99746	0.74522	1.43	0.93978
843	TRUE	Empty	Keratin, type I cuticular Ha8 OS=Homo sapiens GN=KRT38 PE=1 SV=3	KRT38_HUMAN	50 kDa	TRUE	.050574492	0.5	4.06	4.1727	5.47	1.04	1.43	3.91
844	TRUE	Empty	Keratin, type I cytoskeletal 10 OS=Homo sapiens GN=KRT10 PE=1 SV=6	K1C10_HUMAN	59 kDa	TRUE	.063042626	0.08	17.683	7.22	5.47	1.04	0	0.93978
845	TRUE	Empty	Keratin, type I cytoskeletal 16 OS=Homo sapiens GN=KRT16 PE=1 SV=4	K1C16_HUMAN	51 kDa	TRUE	.070115658	0.4	61.889	23.993	45.883	12.669	22.025	13.157
846	TRUE	Empty	Keratin, type I cytoskeletal 18 OS=Homo sapiens GN=KRT18 PE=1 SV=2	K1C18_HUMAN	48 kDa	TRUE	.099430858	0.6	1 246.6	868.96	782.01	382.3	661.61	612.74
847	TRUE	Empty	Keratin, type I cytoskeletal 19 OS=Homo sapiens GN=KRT19 PE=1 SV=4	K1C19_HUMAN	44 kDa	TRUE	.051431323	0.7	800.14	817.85	850.83	722.86	584.52	413.5
848	TRUE	Empty	Keratin, type I cytoskeletal 9 OS=Homo sapiens GN=KRT9 PE=1 SV=3	K1C9_HUMAN	62 kDa	TRUE	.373900966	INF	0	0	0	0	0	1.96
849	TRUE	Empty	Keratin, type II cytoskeletal 1 OS=Homo sapiens GN=KRT1 PE=1 SV=6	K2C1_HUMAN	66 kDa	TRUE	0.80854899	1.1	128.2	67.806	68.825	115.51	54.217	117.47
850	TRUE	Empty	Keratin, type II cytoskeletal 2 epidermal OS=Homo sapiens GN=KRT2 PE=1 SV=2	K22E_HUMAN	65 kDa	TRUE	.531837205	0.6	2.03	10.432	22.942	5.17	2.14	13.157
851	TRUE	Empty	Keratin, type II cytoskeletal 5 OS=Homo sapiens GN=KRT5 PE=1 SV=3	K2C5_HUMAN	62 kDa	TRUE	.119578236	0.3	6.631	6.259	15.959	0.74522	3.85	4.89
852	TRUE	Empty	Keratin, type II cytoskeletal 7 OS=Homo sapiens GN=KRT7 PE=1 SV=5	K2C7_HUMAN	51 kDa	TRUE	0.77922981	0.9	114.94	77.195	100.74	108.8	55.064	109.95
853	TRUE	Empty	Keratin, type II cytoskeletal 8 OS=Homo sapiens GN=KRT8 PE=1 SV=7	K2C8_HUMAN	?	TRUE	.278845167	0.6	1 545.0	690.58	938.61	594.68	652.29	757.47
854	TRUE	Empty	Keratin, type II cytoskeletal 80 OS=Homo sapiens GN=KRT80 PE=1 SV=2	K2C80_HUMAN	?	TRUE	.957419353	1	119.36	70.936	95.756	115.51	57.605	109.01
855	TRUE	Empty	KH domain-containing, RNA-binding, signal transduction-associated protein 1 OS=Homo sapiens GN=KHDRBS1 PE=1 SV=1	KHDR1_HUMAN	?	TRUE	.295436989	2.2	0	0	2.24	2.57	2.14	1.96

856	TRUE	Empty	Kinesin light chain 1 OS=Homo sapiens GN=KLC1 PE=1 SV=2	KLC1_HUMAN	?	TRUE	.439254504	1.5	0	6.259	3.98	4.13	5.99	4.89
857	TRUE	Empty	Kinesin light chain 2 OS=Homo sapiens GN=KLC2 PE=1 SV=1	KLC2_HUMAN	?	TRUE	.439933611	1.7	0	5.59	3.98	8.74	1.43	5.87
858	TRUE	Empty	Kinesin light chain 4 OS=Homo sapiens GN=KLC4 PE=1 SV=3	KLC4_HUMAN	?	TRUE	.071986351	5.9	0	0	0.99746	2.57	0.84713	2.94
859	TRUE	Empty	Kinesin-1 heavy chain OS=Homo sapiens GN=KIF5B PE=1 SV=1	KINH_HUMAN	110 kDa	TRUE	.019434362	4.341659233	0	1.0432	2.24	6.707	4.57	6.85
860	TRUE	Empty	KN motif and ankyrin repeat domain-containing protein 2 OS=Homo sapiens GN=KANK2 PE=1 SV=1	KANK2_HUMAN	?	TRUE	.217218164	INF	0	0	0	0	0.84713	2.94
861	TRUE	Empty	Kynureninase OS=Homo sapiens GN=KYNU PE=1 SV=1	KYNU_HUMAN	?		0.114667062	2.1	0	8.54	7.97	11.178	14.401	9.78
862	TRUE	Empty	Lactotransferrin OS=Homo sapiens GN=LTF PE=1 SV=6	TRFL_HUMAN	?	TRUE	.828998755	0.9	2.03	7.22	13.964	5.17	3.85	11.277
863	TRUE	Empty	Lactoylglutathione lyase OS=Homo sapiens GN=GLO1 PE=1 SV=4	LGUL_HUMAN	?		0.593406265	0.8	4.06	20.863	17.954	11.178	11.013	12.217
864	TRUE	Empty	Ladinin-1 OS=Homo sapiens GN=LAD1 PE=1 SV=2	LAD1_HUMAN	57 kDa	TRUE	.264095074	0.2	0	3.1295	6.22	0	0	1.96
865	TRUE	Empty	Lamina-associated polypeptide 2, isoform alpha OS=Homo sapiens GN=TMPO PE=1 SV=2	LAP2A_HUMAN	75 kDa	TRUE	.035125144	6.430665672	0	3.1295	0.99746	7.22	5.99	13.157
866	TRUE	Empty	Lamina-associated polypeptide 2, isoforms beta/gamma OS=Homo sapiens GN=TMPO PE=1 SV=2	LAP2B_HUMAN	?	TRUE	.069935796	INF	0	0	0	5.17	0.84713	6.85
867	TRUE	Empty	Lamin-B1 OS=Homo sapiens GN=LMNB1 PE=1 SV=2	LMNB1_HUMAN	66 kDa	TRUE	.764197007	0.9	6.631	32.338	32.916	17.14	26.261	19.735
868	TRUE	Empty	Lamin-B2 OS=Homo sapiens GN=LMNB2 PE=1 SV=4	LMNB2_HUMAN	70 kDa	TRUE	.507417069	0.7	2.03	10.432	13.964	4.13	8.13	5.87
869	TRUE	Empty	LanC-like protein 1 OS=Homo sapiens GN=LANCL1 PE=1 SV=1	LANC1_HUMAN	45 kDa		0.420249734	0.4	0	7.22	1.49	0.74522	1.43	0.93978
870	TRUE	Empty	Lanosterol synthase OS=Homo sapiens GN=LSS PE=1 SV=1	ERG7_HUMAN	?	TRUE	.071497131	5.3	2.03	2.0863	0.99746	4.13	8.13	15.037
871	TRUE	Empty	La-related protein 1 OS=Homo sapiens GN=LARP1 PE=1 SV=2	LARP1_HUMAN	?	TRUE	.663928764	0.7	6.631	0	7.97	3.61	5.99	0.93978
872	TRUE	Empty	Large proline-rich protein BAG6 OS=Homo sapiens GN=BAG6 PE=1 SV=2	BAG6_HUMAN	?		0.097956695	1.8	4.06	1.0432	5.47	6.707	6.71	7.83
873	TRUE	Empty	Latexin OS=Homo sapiens GN=LXN PE=1 SV=2	LXN_HUMAN	26 kDa		0.831207279	1.1	24.314	8.54	3.98	14.159	13.554	13.157
874	TRUE	Empty	LDLR chaperone MESD OS=Homo sapiens GN=MESDC2 PE=1 SV=2	MESD_HUMAN	?		0.136122783	INF	0	0	0	2.09	0	1.96
875	TRUE	Empty	Lethal(2) giant larvae protein homolog 2 OS=Homo sapiens GN=LLGL2 PE=1 SV=2	L2GL2_HUMAN	?	TRUE	.166918615	21	0	0	0.99746	14.904	1.43	4.89
876	TRUE	Empty	Leucine carboxyl methyltransferase 1 OS=Homo sapiens GN=LCMT1 PE=1 SV=2	LCMT1_HUMAN	?		0.169759736	INF	0	0	0	0	0.84713	1.96
877	TRUE	Empty	Leucine-rich PPR motif-containing protein, mitochondrial OS=Homo sapiens GN=LRPPRC PE=1 SV=3	LPPRC_HUMAN	158 kDa	TRUE	.029866352	6.453249887	6.631	0	0	19.376	9.85	14.097
878	TRUE	Empty	Leucine-rich repeat flightless-interacting protein 1 OS=Homo sapiens GN=LRRFIP1 PE=1 SV=2	LRRF1_HUMAN	?	TRUE	0.46138078	0.6	0	10.432	8.71	5.65	0.84713	4.89
879	TRUE	Empty	Leucine-rich repeat-containing protein 47 OS=Homo sapiens GN=LRRC47 PE=1 SV=1	LRC47_HUMAN	63 kDa		0.294836952	3	0	0	3.98	7.22	1.43	2.94
880	TRUE	Empty	Leucine-rich repeat-containing protein 59 OS=Homo sapiens GN=LRRC59 PE=1 SV=1	LRC59_HUMAN	35 kDa		0.017294729	5.595982446	4.06	0	0	9.78	8.13	6.85
881	TRUE	Empty	Leucine--tRNA ligase, cytoplasmic OS=Homo sapiens GN=LARS PE=1 SV=2	SYLC_HUMAN	?	TRUE	.128315098	3.2	6.631	0	0	7.22	9.85	4.89
882	TRUE	Empty	Leucyl-cystinyl aminopeptidase OS=Homo sapiens GN=LNPEP PE=1 SV=3	LCAP_HUMAN	?	TRUE	.092374389	4.7	2.03	0	0	1.04	4.57	4.89
883	TRUE	Empty	Leukocyte elastase inhibitor OS=Homo sapiens GN=SERPINB1 PE=1 SV=1	ILEU_HUMAN	?	TRUE	0.06853503	INF	0	0	0	0.74522	2.14	0.93978
884	TRUE	Empty	Leukocyte surface antigen CD47 OS=Homo sapiens GN=CD47 PE=1 SV=1	CD47_HUMAN	?		0.373900966	INF	0	0	0	8.74	0	0
885	TRUE	Empty	Leukotriene A-4 hydrolase OS=Homo sapiens GN=LTA4H PE=1 SV=2	LKHA4_HUMAN	?	TRUE	0.12446721	5.8	2.03	0	0	7.22	1.43	3.91

886	TRUE	Empty	LIM and SH3 domain protein 1 OS=Homo sapiens GN=LASP1 PE=1 SV=2	LASP1_HUMAN	?		0.371921772	0.4	0	4.1727	1.49	0.74522	0.84713	0.93978
887	TRUE	Empty	LIM domain and actin-binding protein 1 OS=Homo sapiens GN=LIMA1 PE=1 SV=1	LIMA1_HUMAN	?	TRUE	.934110007	1.1	0	2.0863	3.98	3.61	0.84713	1.96
888	TRUE	Empty	Lipoma-preferred partner OS=Homo sapiens GN=LPP PE=1 SV=1	LPP_HUMAN	66 kDa		0.219562563	0.2	0	3.1295	3.98	0.74522	0	0.93978
889	TRUE	Empty	L-lactate dehydrogenase A chain OS=Homo sapiens GN=LDHA PE=1 SV=2	LDHA_HUMAN	?	TRUE	.627638521	1.3	61.889	2.0863	20.947	39.497	33.885	39.471
890	TRUE	Empty	Lon protease homolog, mitochondrial OS=Homo sapiens GN=LONP1 PE=1 SV=2	LONM_HUMAN	?		0.043065892	10.54438273	0.99746	0	0	1.04	3.85	5.87
891	TRUE	Empty	Long-chain-fatty-acid--CoA ligase 3 OS=Homo sapiens GN=ACSL3 PE=1 SV=3	ACSL3_HUMAN	80 kDa	TRUE	.005539052	6.571491589	0	0.99746	0	2.09	1.43	1.96
892	TRUE	Empty	Low molecular weight phosphotyrosine protein phosphatase OS=Homo sapiens GN=ACP1 PE=1 SV=3	PPAC_HUMAN	?	TRUE	.749617151	0.9	6.631	7.22	7.97	5.65	10.166	4.89
893	TRUE	Empty	Luc7-like protein 3 OS=Homo sapiens GN=LUC7L3 PE=1 SV=2	LC7L3_HUMAN	?	TRUE	.776817561	0.8	0	4.1727	6.22	2.09	2.14	3.91
894	TRUE	Empty	Lupus La protein OS=Homo sapiens GN=SSB PE=1 SV=2	LA_HUMAN	47 kDa		0.396355964	1.5	4.06	2.0863	11.969	6.707	11.86	9.78
895	TRUE	Empty	L-xylulose reductase OS=Homo sapiens GN=DCXR PE=1 SV=2	DCXR_HUMAN	26 kDa		0.037589604	5.755463957	0	1.0432	0	1.04	1.43	2.94
896	TRUE	Empty	Lysine-specific histone demethylase 1A OS=Homo sapiens GN=KDM1A PE=1 SV=2	KDM1A_HUMAN	?	TRUE	.034371444	12.1316143	0	0.99746	0	2.09	2.14	6.85
897	TRUE	Empty	Lysine--tRNA ligase OS=Homo sapiens GN=KARS PE=1 SV=3	SYK_HUMAN	?	TRUE	0.20079034	3.3	2.03	0	3.98	6.707	11.86	1.96
898	TRUE	Empty	Lysophosphatidylcholine acyltransferase 1 OS=Homo sapiens GN=LPCAT1 PE=1 SV=2	PCAT1_HUMAN	59 kDa		0.177767042	INF	0	0	0	2.57	0	0.93978
899	TRUE	Empty	Lysophospholipid acyltransferase 7 OS=Homo sapiens GN=MBOAT7 PE=1 SV=2	MBOA7_HUMAN	?		0.794426248	0.7	15.472	0	0	3.61	1.43	5.87
900	TRUE	Empty	Lysosomal alpha-glucosidase OS=Homo sapiens GN=GAA PE=1 SV=4	LYAG_HUMAN	105 kDa		0.308046881	0.3	0	18.777	10.972	1.04	5.0828	3.91
901	TRUE	Empty	Lysosomal alpha-mannosidase OS=Homo sapiens GN=MAN2B1 PE=1 SV=3	MA2B1_HUMAN	?		0.664926364	0.6	0	3.1295	0.99746	0.74522	0	1.96
902	TRUE	Empty	Lysosome-associated membrane glycoprotein 1 OS=Homo sapiens GN=LAMP1 PE=1 SV=3	LAMP1_HUMAN	?		0.132552182	INF	0	0	0	2.57	3.85	0
903	TRUE	Empty	Lysosome-associated membrane glycoprotein 2 OS=Homo sapiens GN=LAMP2 PE=1 SV=2	LAMP2_HUMAN	?		0.159551185	1.7	6.631	3.1295	1.49	5.65	6.71	8.81
904	TRUE	Empty	m7GpppX diphosphatase OS=Homo sapiens GN=DCPS PE=1 SV=2	DCPS_HUMAN	39 kDa		0.056790379	5.7	0	0	0.99746	2.57	2.14	0.93978
905	TRUE	Empty	Macrophage migration inhibitory factor OS=Homo sapiens GN=MIF PE=1 SV=4	MIF_HUMAN	12 kDa		0.326863011	0.4	8.13	129.35	70.82	23.847	31.344	36.652
906	TRUE	Empty	Macrophage-capping protein OS=Homo sapiens GN=CAPG PE=1 SV=2	CAPG_HUMAN	?		0.755173895	1.4	0	0	4.73	1.04	0.84713	4.89
907	TRUE	Empty	Major vault protein OS=Homo sapiens GN=MVP PE=1 SV=4	MVP_HUMAN	99 kDa	TRUE	.184606477	INF	0	0	0	0.74522	0	1.96
908	TRUE	Empty	Malate dehydrogenase, cytoplasmic OS=Homo sapiens GN=MDH1 PE=1 SV=4	MDHC_HUMAN	?		0.349939865	0.8	13.262	15.648	9.46	13.414	9.85	9.78
909	TRUE	Empty	Malate dehydrogenase, mitochondrial OS=Homo sapiens GN=MDH2 PE=1 SV=3	MDHM_HUMAN	?		0.877803289	1	72.941	116.84	70.82	70.796	111.82	87.4
910	TRUE	Empty	Malectin OS=Homo sapiens GN=MLEC PE=1 SV=1	MLEC_HUMAN	32 kDa	TRUE	.121392661	INF	0	0	0	2.57	0	2.94
911	TRUE	Empty	Maleylacetoacetate isomerase OS=Homo sapiens GN=GSTZ1 PE=1 SV=3	MAAI_HUMAN	?		0.373824557	2.7	4.06	0	0	5.65	6.71	0
912	TRUE	Empty	Malignant T-cell-amplified sequence 1 OS=Homo sapiens GN=MCTS1 PE=1 SV=1	MCTS1_HUMAN	?	TRUE	.754650149	0.9	2.03	10.432	10.972	10.433	5.0828	4.89

913	TRUE	Empty	Manganese-transporting ATPase 13A1 OS=Homo sapiens GN=ATP13A1 PE=1 SV=2	AT131_HUMAN	?	TRUE	.030400164	5.179375614	0.99746	0	0	0.74522	2.14	1.96
914	TRUE	Empty	Mannose-1-phosphate guanyltransferase alpha OS=Homo sapiens GN=GMPPA PE=1 SV=1	GMPPA_HUMAN	?		0.201105011	2.2	2.03	1.0432	0.99746	1.04	5.0828	2.94
915	TRUE	Empty	Mannosyl-oligosaccharide glucosidase OS=Homo sapiens GN=MOGS PE=1 SV=5	MOGS_HUMAN	?	TRUE	.064585291	INF	0	0	0	0.74522	5.0828	4.89
916	TRUE	Empty	Matrin-3 OS=Homo sapiens GN=MATR3 PE=1 SV=2	MATR3_HUMAN	?		0.449446767	1.1	19.893	33.382	32.916	35.025	35.58	28.194
917	TRUE	Empty	Melanophilin OS=Homo sapiens GN=MLPH PE=1 SV=1	MELPH_HUMAN	?	TRUE	.091068278	0.08	2.03	11.475	5.47	1.04	0	0
918	TRUE	Empty	Membrane-associated progesterone receptor component 2 OS=Homo sapiens GN=PGRMC2 PE=1 SV=1	PGRC2_HUMAN	?	TRUE	.337215899	2.2	8.13	0	0	5.17	8.13	4.89
919	TRUE	Empty	Mesencephalic astrocyte-derived neurotrophic factor OS=Homo sapiens GN=MANF PE=1 SV=3	MANF_HUMAN	21 kDa		0.15359	0	0	1.0432	1.49	0	0	0
920	TRUE	Empty	Metalloproteinase inhibitor 3 OS=Homo sapiens GN=TIMP3 PE=1 SV=2	TIMP3_HUMAN	24 kDa	TRUE	0.15359	0	0	1.0432	1.49	0	0	0
921	TRUE	Empty	Metastasis-associated protein MTA1 OS=Homo sapiens GN=MTA1 PE=1 SV=2	MTA1_HUMAN	?	TRUE	.017663121	3.387905279	0	0.99746	0	0.74522	1.43	0.93978
922	TRUE	Empty	Methionine adenosyltransferase 2 subunit beta OS=Homo sapiens GN=MAT2B PE=1 SV=1	MAT2B_HUMAN	?		0.82088714	1.1	0	4.1727	2.24	3.61	2.14	1.96
923	TRUE	Empty	Methionine aminopeptidase 1 OS=Homo sapiens GN=METAP1 PE=1 SV=2	MAP11_HUMAN	43 kDa		0.62105497	0.7	0	8.54	7.97	6.707	4.57	0
924	TRUE	Empty	Methionine aminopeptidase 2 OS=Homo sapiens GN=METAP2 PE=1 SV=1	MAP2_HUMAN	?	TRUE	.699734897	1.9	0	0	1.49	0	0	3.91
925	TRUE	Empty	Methionine--tRNA ligase, cytoplasmic OS=Homo sapiens GN=MARS PE=1 SV=2	SYMC_HUMAN	?	TRUE	.102840484	7.4	0	0	0.99746	2.57	4.57	0.93978
926	TRUE	Empty	Methylated-DNA--protein-cysteine methyltransferase OS=Homo sapiens GN=MGMT PE=1 SV=1	MGMT_HUMAN	22 kDa		0.331247655	0.2	0	1.0432	3.98	0	0	0.93978
927	TRUE	Empty	Methyl-CpG-binding domain protein 3 OS=Homo sapiens GN=MBD3 PE=1 SV=1	MBD3_HUMAN	?	TRUE	.373900966	INF	0	0	0	0	0	1.96
928	TRUE	Empty	Methylcrotonoyl-CoA carboxylase beta chain, mitochondrial OS=Homo sapiens GN=MCCC2 PE=1 SV=1	MCCB_HUMAN	?	TRUE	.001800746	15.33364746	0	0.99746	0	3.61	5.99	5.87
929	TRUE	Empty	Methylosome protein 50 OS=Homo sapiens GN=WDR77 PE=1 SV=1	MEP50_HUMAN	?		0.32385374	2.4	0	3.1295	4.73	4.13	2.14	12.217
930	TRUE	Empty	Methylthioribose-1-phosphate isomerase OS=Homo sapiens GN=MRI1 PE=1 SV=1	MTNA_HUMAN	?		0.34494292	2.4	2.03	0	0	0.74522	1.43	2.94
931	TRUE	Empty	Mevalonate kinase OS=Homo sapiens GN=MVK PE=1 SV=1	KIME_HUMAN	42 kDa		0.571575755	1.6	0	0	1.49	1.04	0.84713	0.93978
932	TRUE	Empty	MICOS complex subunit MIC19 OS=Homo sapiens GN=CHCHD3 PE=1 SV=1	MIC19_HUMAN	26 kDa		0.922942221	1.1	2.03	3.1295	2.24	8.74	0	0.93978
933	TRUE	Empty	MICOS complex subunit MIC60 OS=Homo sapiens GN=IMMT PE=1 SV=1	MIC60_HUMAN	?	TRUE	.012256656	6.743654708	2.03	0	0	5.65	5.99	3.91
934	TRUE	Empty	Microsomal glutathione S-transferase 1 OS=Homo sapiens GN=MGST1 PE=1 SV=1	MGST1_HUMAN	?		0.064408264	INF	0	0	0	1.04	4.57	7.83
935	TRUE	Empty	Microtubule-associated protein 4 OS=Homo sapiens GN=MAP4 PE=1 SV=3	MAP4_HUMAN	?	TRUE	.778794658	0.8	0	8.54	2.24	1.04	1.43	5.87
936	TRUE	Empty	Microtubule-associated protein RP/EB family member 1 OS=Homo sapiens GN=MAPRE1 PE=1 SV=3	MARE1_HUMAN	30 kDa	TRUE	.741285641	1.3	0	1.0432	3.98	2.09	2.14	0.93978
937	TRUE	Empty	Mitogen-activated protein kinase 14 OS=Homo sapiens GN=MAPK14 PE=1 SV=3	MK14_HUMAN	?	TRUE	0.07687344	INF	0	0	0	4.13	0.84713	6.85
938	TRUE	Empty	Mitogen-activated protein kinase 3 OS=Homo sapiens GN=MAPK3 PE=1 SV=4	MK03_HUMAN	?	TRUE	.122043508	5.2	0	0	1.49	5.17	2.14	1.96
939	TRUE	Empty	Mitochondrial 2-oxoglutarate/malate carrier protein OS=Homo sapiens GN=SLC25A11 PE=1 SV=3	M2OM_HUMAN	?		0.041547618	5.170061957	0.99746	0	0	1.04	0.84713	2.94
940	TRUE	Empty	Mitochondrial antiviral-signaling protein OS=Homo sapiens GN=MAVS PE=1 SV=2	MAVS_HUMAN	?		0.220019143	INF	0	0	0	0.74522	2.14	0

941	TRUE	Empty	Mitochondrial carrier homolog 2 OS=Homo sapiens GN=MTCH2 PE=1 SV=1	MTCH2_HUMAN	33 kDa		0.019977028	18.26248672	0	0.99746	0	2.09	6.71	8.81
942	TRUE	Empty	Mitochondrial dicarboxylate carrier OS=Homo sapiens GN=SLC25A10 PE=1 SV=2	DIC_HUMAN	?	TRUE	.005353347	7.522908187	0	0	0.99746	2.57	3.85	1.96
943	TRUE	Empty	Mitochondrial fission 1 protein OS=Homo sapiens GN=FIS1 PE=1 SV=2	FIS1_HUMAN	17 kDa		0.445736559	0.7	4.06	7.22	12.967	6.707	5.99	5.87
944	TRUE	Empty	Mitochondrial import inner membrane translocase subunit Tim13 OS=Homo sapiens GN=TIMM13 PE=1 SV=1	TIM13_HUMAN	11 kDa		0.734949237	1.2	0	3.1295	4.73	4.13	2.14	2.94
945	TRUE	Empty	Mitochondrial import inner membrane translocase subunit TIM50 OS=Homo sapiens GN=TIMM50 PE=1 SV=2	TIM50_HUMAN	?		0.642280054	1.5	4.06	0	0	2.57	2.14	1.96
946	TRUE	Empty	Mitochondrial import inner membrane translocase subunit Tim8 A OS=Homo sapiens GN=TIMM8A PE=1 SV=1	TIM8A_HUMAN	11 kDa		0.715736046	0.7	0	8.54	2.24	1.04	0.84713	5.87
947	TRUE	Empty	Mitochondrial import receptor subunit TOM22 homolog OS=Homo sapiens GN=TOMM22 PE=1 SV=3	TOM22_HUMAN	16 kDa		0.911150022	1	4.06	3.1295	1.49	4.13	0.84713	3.91
948	TRUE	Empty	Mitochondrial import receptor subunit TOM34 OS=Homo sapiens GN=TOMM34 PE=1 SV=2	TOM34_HUMAN	35 kDa		0.664294556	0.7	0	7.22	1.49	0.74522	2.14	2.94
949	TRUE	Empty	Mitochondrial import receptor subunit TOM40 homolog OS=Homo sapiens GN=TOMM40 PE=1 SV=1	TOM40_HUMAN	?		0.171563696	INF	0	0	0	0.74522	1.43	6.85
950	TRUE	Empty	Mitochondrial import receptor subunit TOM70 OS=Homo sapiens GN=TOMM70A PE=1 SV=1	TOM70_HUMAN	67 kDa		0.043064785	5.272311672	0	0.99746	0	0.74522	1.43	2.94
951	TRUE	Empty	Mitochondrial-processing peptidase subunit alpha OS=Homo sapiens GN=PMPCA PE=1 SV=2	MPPA_HUMAN	?		0.564332138	1.9	2.03	0	0	2.57	0	1.96
952	TRUE	Empty	Mitotic checkpoint protein BUB3 OS=Homo sapiens GN=BUB3 PE=1 SV=1	BUB3_HUMAN	?	TRUE	.109007107	2.4	2.03	1.0432	0.99746	2.57	5.0828	2.94
953	TRUE	Empty	Mitotic-spindle organizing protein 2B OS=Homo sapiens GN=MZT2B PE=1 SV=1	MZT2B_HUMAN	16 kDa	TRUE	.341617822	0.4	0	5.59	2.24	1.04	0.84713	0.93978
954	TRUE	Empty	MMS19 nucleotide excision repair protein homolog OS=Homo sapiens GN=MMS19 PE=1 SV=2	MMS19_HUMAN	?	TRUE	.004145238	11.65791109	0.99746	0	0	3.61	5.0828	2.94
955	TRUE	Empty	Monoglyceride lipase OS=Homo sapiens GN=MGLL PE=1 SV=2	MGLL_HUMAN	?		0.724984579	0.6	0	0	2.24	0	1.43	0
956	TRUE	Empty	mRNA cap guanine-N7 methyltransferase OS=Homo sapiens GN=RNMT PE=1 SV=1	MCES_HUMAN	?		0.373900966	INF	0	0	0	1.04	0	0
957	TRUE	Empty	mRNA export factor OS=Homo sapiens GN=RAE1 PE=1 SV=1	RAE1L_HUMAN	41 kDa		0.696449592	1.2	0	6.259	6.22	3.61	5.99	6.85
958	TRUE	Empty	Mucin-5AC OS=Homo sapiens GN=MUC5AC PE=1 SV=4	MUC5A_HUMAN	586 kDa	TRUE	.000408349	3.897928144	6.631	4.1727	3.98	18.63	21.178	17.856
959	TRUE	Empty	Mucin-5B OS=Homo sapiens GN=MUC5B PE=1 SV=3	MUC5B_HUMAN	596 kDa	TRUE	0.20307663	5.4	0	0	0.99746	0	2.14	2.94
960	TRUE	Empty	Multifunctional methyltransferase subunit TRM112-like protein OS=Homo sapiens GN=TRMT112 PE=1 SV=1	TR112_HUMAN	?		0.889953256	1.1	0	2.0863	0.99746	0.74522	1.43	0.93978
961	TRUE	Empty	Multifunctional protein ADE2 OS=Homo sapiens GN=PAICS PE=1 SV=3	PUR6_HUMAN	?	TRUE	0.06781201	3	0	3.1295	5.47	12.669	8.13	6.85
962	TRUE	Empty	Multiple myeloma tumor-associated protein 2 OS=Homo sapiens GN=MMTAG2 PE=1 SV=1	MMTA2_HUMAN	?	TRUE	.699728631	1.9	0	0	0.99746	0	0	1.96
963	TRUE	Empty	Muscleblind-like protein 1 OS=Homo sapiens GN=MBNL1 PE=1 SV=2	MBNL1_HUMAN	?	TRUE	.336428859	0.4	0	4.1727	2.24	0	0.84713	1.96
964	TRUE	Empty	Muscleblind-like protein 2 OS=Homo sapiens GN=MBNL2 PE=1 SV=2	MBNL2_HUMAN	?	TRUE	.304738405	0.3	0	4.1727	1.49	0	0.84713	0.93978
965	TRUE	Empty	Myb-binding protein 1A OS=Homo sapiens GN=MYBBP1A PE=1 SV=2	MBB1A_HUMAN	?		0.06853503	INF	0	0	0	0.74522	2.14	0.93978
966	TRUE	Empty	Myelin expression factor 2 OS=Homo sapiens GN=MYEF2 PE=1 SV=3	MYEF2_HUMAN	?	TRUE	.867329491	0.9	0	1.0432	1.49	0	1.43	0.93978
967	TRUE	Empty	Myeloid-derived growth factor OS=Homo sapiens GN=MYDGF PE=1 SV=1	MYDGF_HUMAN	19 kDa	TRUE	0.53228718	1.9	0	5.59	0	2.09	5.99	0.93978
968	TRUE	Empty	Myoferlin OS=Homo sapiens GN=MYOF PE=1 SV=1	MYOF_HUMAN	?	TRUE	.015463896	6.924766429	4.06	0	0.99746	11.178	16.943	9.78

969	TRUE	Empty	Myoglobin OS=Homo sapiens GN=MB PE=1 SV=2	MYG_HUMAN	17 kDa		0.485767229	0.4	0	5.59	0.99746	0.74522	0.84713	0.93978
970	TRUE	Empty	Myosin light polypeptide 6 OS=Homo sapiens GN=MYL6 PE=1 SV=2	MYL6_HUMAN	?	TRUE	.188235244	0.7	44.206	51.115	81.792	33.535	45.745	40.411
971	TRUE	Empty	Myosin regulatory light chain 12B OS=Homo sapiens GN=MYL12B PE=1 SV=2	ML12B_HUMAN	20 kDa		0.362589523	0.5	6.631	17.734	39.898	14.904	11.013	7.83
972	TRUE	Empty	Myosin-10 OS=Homo sapiens GN=MYH10 PE=1 SV=3	MYH10_HUMAN	?	TRUE	.159157955	3	8.13	2.0863	4.73	27.573	7.42	13.157
973	TRUE	Empty	Myosin-14 OS=Homo sapiens GN=MYH14 PE=1 SV=2	MYH14_HUMAN	?	TRUE	.108512691	3	28.734	4.1727	8.71	55.891	19.484	50.748
974	TRUE	Empty	Myosin-9 OS=Homo sapiens GN=MYH9 PE=1 SV=4	MYH9_HUMAN	?	TRUE	.409542825	1.7	101.67	5.59	18.952	78.248	53.369	80.821
975	TRUE	Empty	Myotrophin OS=Homo sapiens GN=MTPN PE=1 SV=2	MTPN_HUMAN	13 kDa		0.259791678	1.7	2.03	2.0863	2.24	5.17	4.57	1.96
976	TRUE	Empty	Myotubularin-related protein 14 OS=Homo sapiens GN=MTMR14 PE=1 SV=2	MTMRE_HUMAN	?	TRUE	.189953018	INF	0	0	0	2.57	0.84713	0
977	TRUE	Empty	Myristoylated alanine-rich C-kinase substrate OS=Homo sapiens GN=MARCKS PE=1 SV=4	MARCS_HUMAN	32 kDa		0.041547618	5.170061957	0	0	0.99746	1.04	0.84713	2.94
978	TRUE	Empty	N(G),N(G)-dimethylarginine dimethylaminohydrolase 2 OS=Homo sapiens GN=DDAH2 PE=1 SV=1	DDAH2_HUMAN	30 kDa	TRUE	.490425624	0.6	0	5.59	5.47	2.09	0.84713	2.94
979	TRUE	Empty	Na(+)/H(+) exchange regulatory cofactor NHE-RF1 OS=Homo sapiens GN=SLC9A3R1 PE=1 SV=4	NHRF1_HUMAN	?	TRUE	.244573065	0.5	11.052	27.122	46.881	16.395	11.86	14.097
980	TRUE	Empty	Na(+)/H(+) exchange regulatory cofactor NHE-RF2 OS=Homo sapiens GN=SLC9A3R2 PE=1 SV=2	NHRF2_HUMAN	?		0.540731665	0.3	0	5.59	0	0.74522	0	0.93978
981	TRUE	Empty	N-acetyl-D-glucosamine kinase OS=Homo sapiens GN=NAGK PE=1 SV=4	NAGK_HUMAN	?		0.306830256	0.6	4.06	10.432	10.972	8.26	4.57	2.94
982	TRUE	Empty	N-acetylglucosamine-6-sulfatase OS=Homo sapiens GN=GNS PE=1 SV=3	GNS_HUMAN	?		0.919600883	0.9	0	5.59	4.73	1.04	3.85	4.89
983	TRUE	Empty	N-acetylserotonin O-methyltransferase-like protein OS=Homo sapiens GN=ASMTL PE=1 SV=3	ASML_HUMAN	?		0.123359563	3	2.03	0	0	2.57	2.14	1.96
984	TRUE	Empty	NAD(P)H dehydrogenase [quinone] 1 OS=Homo sapiens GN=NQO1 PE=1 SV=1	NQO1_HUMAN	?		0.01397539	21.53910934	0	0	0.99746	5.65	5.99	10.338
985	TRUE	Empty	NAD(P)H-hydrate epimerase OS=Homo sapiens GN=APOA1BP PE=1 SV=2	NNRE_HUMAN	?		0.892714961	1.1	0	3.1295	3.98	0.74522	4.57	2.94
986	TRUE	Empty	NADH dehydrogenase [ubiquinone] 1 alpha subcomplex subunit 10, mitochondrial OS=Homo sapiens GN=NDUFA10 PE=1 SV=1	NDUAA_HUMAN	?		0.00025994	5.824393961	0	0.99746	0	2.57	1.43	1.96
987	TRUE	Empty	NADH dehydrogenase [ubiquinone] 1 alpha subcomplex subunit 5 OS=Homo sapiens GN=NDUFA5 PE=1 SV=3	NDUA5_HUMAN	?		0.005539052	6.571491589	0	0	0.99746	2.09	1.43	1.96
988	TRUE	Empty	NADH dehydrogenase [ubiquinone] 1 alpha subcomplex subunit 8 OS=Homo sapiens GN=NDUFA8 PE=1 SV=3	NDUA8_HUMAN	20 kDa		0.612541843	1.6	0	2.0863	0	1.04	0.84713	0.93978
989	TRUE	Empty	NADH dehydrogenase [ubiquinone] 1 alpha subcomplex subunit 9, mitochondrial OS=Homo sapiens GN=NDUFA9 PE=1 SV=2	NDUA9_HUMAN	43 kDa		0.063218814	INF	0	0	0	2.09	0.84713	4.89
990	TRUE	Empty	NADH dehydrogenase [ubiquinone] 1 beta subcomplex subunit 10 OS=Homo sapiens GN=NDUFB10 PE=1 SV=3	NDUBA_HUMAN	?		0.405649664	3	2.03	4.1727	2.24	22.357	2.14	2.94
991	TRUE	Empty	NADH dehydrogenase [ubiquinone] flavoprotein 2, mitochondrial OS=Homo sapiens GN=NDUFV2 PE=1 SV=2	NDUV2_HUMAN	27 kDa		0.028435388	7.379505368	0	1.0432	0	1.04	3.85	2.94
992	TRUE	Empty	NADH dehydrogenase [ubiquinone] iron-sulfur protein 8, mitochondrial OS=Homo sapiens GN=NDUFS8 PE=1 SV=1	NDUS8_HUMAN	24 kDa		0.111295643	3.4	2.03	0	0	2.57	3.85	1.96
993	TRUE	Empty	NADH-cytochrome b5 reductase 1 OS=Homo sapiens GN=CYB5R1 PE=1 SV=1	NB5R1_HUMAN	34 kDa	TRUE	.257234701	2.6	2.03	0	0	2.57	2.14	0.93978
994	TRUE	Empty	NADH-cytochrome b5 reductase 3 OS=Homo sapiens GN=CYB5R3 PE=1 SV=3	NB5R3_HUMAN	?	TRUE	.009521936	4.227868787	0.99746	0	0	1.04	0.84713	1.96
995	TRUE	Empty	NADH-ubiquinone oxidoreductase 75 kDa subunit, mitochondrial OS=Homo sapiens GN=NDUFS1 PE=1 SV=3	NDUS1_HUMAN	?		0.005921119	6.873637063	2.03	0	0	4.13	5.0828	5.87
996	TRUE	Empty	NADP-dependent malic enzyme OS=Homo sapiens GN=ME1 PE=1 SV=1	MAOX_HUMAN	?	TRUE	.366913381	0.4	0	5.59	4.73	0	0	3.91

997	TRUE	Empty	NADPH:adrenodoxin oxidoreductase, mitochondrial OS=Homo sapiens GN=FDXR PE=1 SV=3	ADRO_HUMAN	?	TRUE	.025516134	6.47853548	0	0.99746	0	2.09	2.14	0.93978
998	TRUE	Empty	NADPH--cytochrome P450 reductase OS=Homo sapiens GN=POR PE=1 SV=2	NCPR_HUMAN	77 kDa		0.041670966	27.09943256	0	0	0.99746	5.65	6.71	15.037
999	TRUE	Empty	N-alpha-acetyltransferase 10 OS=Homo sapiens GN=NAA10 PE=1 SV=1	NAA10_HUMAN	?	TRUE	.693507807	1.6	0	0	1.49	2.57	0	0.93978
1000	TRUE	Empty	N-alpha-acetyltransferase 15, NatA auxiliary subunit OS=Homo sapiens GN=NAA15 PE=1 SV=1	NAA15_HUMAN	?	TRUE	.782182663	1.4	2.03	0	0	2.57	0.84713	0
1001	TRUE	Empty	N-alpha-acetyltransferase 50 OS=Homo sapiens GN=NAA50 PE=1 SV=1	NAA50_HUMAN	?		0.023730014	6.571491589	0	0	0.99746	2.09	1.43	1.96
1002	TRUE	Empty	Nascent polypeptide-associated complex subunit alpha, muscle-specific form OS=Homo sapiens GN=NACA PE=1 SV=1	NACAM_HUMAN	?	TRUE	.391425709	0.8	13.262	31.295	28.926	17.885	19.484	19.735
1003	TRUE	Empty	Nck-associated protein 1 OS=Homo sapiens GN=NCKAP1 PE=1 SV=1	NCKP1_HUMAN	?	TRUE	.140218517	INF	0	0	0	0	1.43	2.94
1004	TRUE	Empty	NEDD8-activating enzyme E1 catalytic subunit OS=Homo sapiens GN=UBA3 PE=1 SV=2	UBA3_HUMAN	?		0.147672906	INF	0	0	0	0	1.43	0.93978
1005	TRUE	Empty	NEDD8-conjugating enzyme Ubc12 OS=Homo sapiens GN=UBE2M PE=1 SV=1	UBC12_HUMAN	21 kDa	TRUE	.894417051	0.9	6.631	2.0863	1.49	2.09	4.57	2.94
1006	TRUE	Empty	Negative elongation factor B OS=Homo sapiens GN=NELFB PE=1 SV=1	NELFB_HUMAN	66 kDa	TRUE	0.03308591	6.961482165	0	0.99746	0	1.04	1.43	3.91
1007	TRUE	Empty	Negative elongation factor C/D OS=Homo sapiens GN=NELFCD PE=1 SV=2	NELFD_HUMAN	?		0.250318529	INF	0	0	0	0	4.57	0.93978
1008	TRUE	Empty	Nesprin-2 OS=Homo sapiens GN=SYNE2 PE=1 SV=3	SYNE2_HUMAN	?	TRUE	.388590446	3	0	1.0432	0	2.57	0	0.93978
1009	TRUE	Empty	Neuroblast differentiation-associated protein AHNAK OS=Homo sapiens GN=AHNAK PE=1 SV=2	AHNK_HUMAN	?	TRUE	.306890307	0.5	11.052	197.16	175.55	61.108	38.968	74.243
1010	TRUE	Empty	Neurochondrin OS=Homo sapiens GN=NCDN PE=1 SV=1	NCDN_HUMAN	?		0.080922552	INF	0	0	0	0.74522	1.43	3.91
1011	TRUE	Empty	Neuronal cell adhesion molecule OS=Homo sapiens GN=NRCAM PE=1 SV=3	NRCAM_HUMAN	?	TRUE	.228035754	0	0	14.604	3.98	0	0	0
1012	TRUE	Empty	Neutral alpha-glucosidase AB OS=Homo sapiens GN=GANAB PE=1 SV=3	GANAB_HUMAN	?		0.454141594	1.8	37.575	2.0863	0.99746	29.064	15.248	28.194
1013	TRUE	Empty	Neutral amino acid transporter B(0) OS=Homo sapiens GN=SLC1A5 PE=1 SV=2	AAAT_HUMAN	?		0.014795304	4.798428497	4.06	3.1295	1.49	10.433	20.331	15.037
1014	TRUE	Empty	NF-kappa-B essential modulator OS=Homo sapiens GN=IKBKG PE=1 SV=2	NEMO_HUMAN	?	TRUE	0.2341866	0.2	0	4.1727	1.49	0	0	0.93978
1015	TRUE	Empty	NHP2-like protein 1 OS=Homo sapiens GN=SNU13 PE=1 SV=3	NH2L1_HUMAN	14 kDa		0.751566327	1.2	0	9.86	9.46	8.74	2.14	13.157
1016	TRUE	Empty	Niban-like protein 1 OS=Homo sapiens GN=FAM129B PE=1 SV=3	NIBL1_HUMAN	?		0.001659387	9.831750423	2.03	1.0432	0	8.26	12.707	10.338
1017	TRUE	Empty	Nicotinamide phosphoribosyltransferase OS=Homo sapiens GN=NAMPT PE=1 SV=1	NAMPT_HUMAN	56 kDa	TRUE	.001501257	11.94584244	0	0	0.99746	2.09	4.57	4.89
1018	TRUE	Empty	Nicotinate phosphoribosyltransferase OS=Homo sapiens GN=NAPRT PE=1 SV=2	PNCB_HUMAN	?	TRUE	.189605526	0.1	0	5.59	8.71	0	0.84713	0.93978
1019	TRUE	Empty	NIF3-like protein 1 OS=Homo sapiens GN=NIF3L1 PE=1 SV=2	NIF3L_HUMAN	?	TRUE	.506553997	0.2	0	0	2.24	0.74522	0	0
1020	TRUE	Empty	Nitric oxide synthase-interacting protein OS=Homo sapiens GN=NOSIP PE=1 SV=1	NOSIP_HUMAN	33 kDa		0.67700455	0.7	0	5.59	4.73	5.17	0.84713	0
1021	TRUE	Empty	Nodal modulator 1 OS=Homo sapiens GN=NOMO1 PE=1 SV=5	NOMO1_HUMAN	134 kDa		0.043064785	5.272311672	0.99746	0	0	0.74522	1.43	2.94
1022	TRUE	Empty	Non-POU domain-containing octamer-binding protein OS=Homo sapiens GN=NONO PE=1 SV=4	NONO_HUMAN	?	TRUE	.837073983	1.1	15.472	18.777	7.97	14.904	11.86	17.856
1023	TRUE	Empty	Non-specific lipid-transfer protein OS=Homo sapiens GN=SCP2 PE=1 SV=2	NLTP_HUMAN	?		0.92145333	1.1	0	11.475	7.97	5.65	4.57	11.277

1024	TRUE	Empty	Notchless protein homolog 1 OS=Homo sapiens GN=NLE1 PE=1 SV=4	NLE1_HUMAN	?		0.303239214	0.3	0	3.1295	2.24	0	0	1.96
1025	TRUE	Empty	NSFL1 cofactor p47 OS=Homo sapiens GN=NSFL1C PE=1 SV=2	NSF1C_HUMAN	?	TRUE	.298120696	2.3	0	0	2.24	1.04	2.14	2.94
1026	TRUE	Empty	N-sulphoglucosamine sulphohydrolase OS=Homo sapiens GN=SGSH PE=1 SV=1	SPHM_HUMAN	57 kDa		0.373900966	0	0	2.0863	0	0	0	0
1027	TRUE	Empty	N-terminal kinase-like protein OS=Homo sapiens GN=SCYL1 PE=1 SV=1	NTKL_HUMAN	?	TRUE	.373900966	INF	0	0	0	2.57	0	0
1028	TRUE	Empty	Nuclear autoantigenic sperm protein OS=Homo sapiens GN=NASP PE=1 SV=2	NASP_HUMAN	?		0.658389285	0.8	13.262	14.604	10.972	18.63	3.85	10.338
1029	TRUE	Empty	Nuclear cap-binding protein subunit 1 OS=Homo sapiens GN=NCBP1 PE=1 SV=1	NCBP1_HUMAN	92 kDa		0.002480722	29.91117438	0	0	0.99746	7.22	10.166	12.217
1030	TRUE	Empty	Nuclear factor NF-kappa-B p105 subunit OS=Homo sapiens GN=NFKB1 PE=1 SV=2	NFKB1_HUMAN	?	TRUE	.884250073	1.2	0	2.0863	0.99746	3.61	0	0
1031	TRUE	Empty	Nuclear migration protein nudC OS=Homo sapiens GN=NUDC PE=1 SV=1	NUDC_HUMAN	38 kDa		0.4812811	1.6	0	1.0432	4.73	4.13	2.14	2.94
1032	TRUE	Empty	Nuclear mitotic apparatus protein 1 OS=Homo sapiens GN=NUMA1 PE=1 SV=2	NUMA1_HUMAN	?	TRUE	.202792374	2.9	6.631	1.0432	2.24	18.63	6.71	5.87
1033	TRUE	Empty	Nuclear pore complex protein Nup133 OS=Homo sapiens GN=NUP133 PE=1 SV=2	NU133_HUMAN	129 kDa	TRUE	.003020465	5.926453191	0	0.99746	0	1.04	2.14	1.96
1034	TRUE	Empty	Nuclear pore complex protein Nup153 OS=Homo sapiens GN=NUP153 PE=1 SV=2	NU153_HUMAN	?		0.985771666	1	0	1.0432	4.73	5.65	0	0.93978
1035	TRUE	Empty	Nuclear pore complex protein Nup160 OS=Homo sapiens GN=NUP160 PE=1 SV=3	NU160_HUMAN	?		0.033085386	10.4423235	0	0.99746	0	2.57	2.14	5.87
1036	TRUE	Empty	Nuclear pore complex protein Nup205 OS=Homo sapiens GN=NUP205 PE=1 SV=3	NU205_HUMAN	228 kDa		0.12361009	INF	0	0	0	2.57	1.43	0
1037	TRUE	Empty	Nuclear pore complex protein Nup214 OS=Homo sapiens GN=NUP214 PE=1 SV=2	NU214_HUMAN	?		0.119448566	INF	0	0	0	0	3.85	2.94
1038	TRUE	Empty	Nuclear pore complex protein Nup93 OS=Homo sapiens GN=NUP93 PE=1 SV=2	NUP93_HUMAN	?		0.082651893	INF	0	0	0	2.57	0.84713	4.89
1039	TRUE	Empty	Nuclear pore glycoprotein p62 OS=Homo sapiens GN=NUP62 PE=1 SV=3	NUP62_HUMAN	53 kDa		0.976785061	1	0	3.1295	0.99746	1.04	0.84713	1.96
1040	TRUE	Empty	Nuclear pore membrane glycoprotein 210 OS=Homo sapiens GN=NUP210 PE=1 SV=3	PO210_HUMAN	?	TRUE	.184606477	INF	0	0	0	0.74522	0	1.96
1041	TRUE	Empty	Nuclear receptor-binding protein OS=Homo sapiens GN=NRBP1 PE=1 SV=1	NRBP_HUMAN	60 kDa		0.003020465	5.926453191	0.99746	0	0	1.04	2.14	1.96
1042	TRUE	Empty	Nuclear RNA export factor 1 OS=Homo sapiens GN=NXF1 PE=1 SV=1	NXF1_HUMAN	?		0.140218517	INF	0	0	0	0	1.43	2.94
1043	TRUE	Empty	Nuclear transport factor 2 OS=Homo sapiens GN=NUTF2 PE=1 SV=1	NTF2_HUMAN	14 kDa		0.920331629	0.9	0	6.259	11.969	2.09	10.166	3.91
1044	TRUE	Empty	Nuclease-sensitive element-binding protein 1 OS=Homo sapiens GN=YBX1 PE=1 SV=3	YBOX1_HUMAN	36 kDa	TRUE	.300866003	0.7	26.524	28.166	47.878	32.79	24.567	14.097
1045	TRUE	Empty	Nucleobindin-1 OS=Homo sapiens GN=NUCB1 PE=1 SV=4	NUCB1_HUMAN	54 kDa	TRUE	.172538338	INF	0	0	0	0.74522	1.43	0
1046	TRUE	Empty	Nucleolar and coiled-body phosphoprotein 1 OS=Homo sapiens GN=NOLC1 PE=1 SV=2	NOLC1_HUMAN	?	TRUE	.387501309	0.7	13.262	10.432	4.73	6.707	10.166	1.96
1047	TRUE	Empty	Nucleolar GTP-binding protein 1 OS=Homo sapiens GN=GTPBP4 PE=1 SV=3	NOG1_HUMAN	?	TRUE	.017661144	6.775710304	0	0.99746	0	1.04	3.85	1.96
1048	TRUE	Empty	Nucleolar protein 11 OS=Homo sapiens GN=NOL11 PE=1 SV=1	NOL11_HUMAN	?	TRUE	.094905451	INF	0	0	0	0.74522	0.84713	2.94
1049	TRUE	Empty	Nucleolar protein 16 OS=Homo sapiens GN=NOP16 PE=1 SV=2	NOP16_HUMAN	?		0.373900966	INF	0	0	0	0	0	1.96
1050	TRUE	Empty	Nucleolar protein 56 OS=Homo sapiens GN=NOP56 PE=1 SV=4	NOP56_HUMAN	66 kDa	TRUE	.145067648	4	0	0	0.99746	2.57	0.84713	0.93978

1051	TRUE	Empty	Nucleolar protein 58 OS=Homo sapiens GN=NOP58 PE=1 SV=1	NOP58_HUMAN	60 kDa		0.980885008	1	8.13	1.0432	3.98	6.707	5.0828	1.96
1052	TRUE	Empty	Nucleolar protein 9 OS=Homo sapiens GN=NOP9 PE=1 SV=1	NOP9_HUMAN	?	TRUE	.178905938	7.8	0	1.0432	0	0	3.85	4.89
1053	TRUE	Empty	Nucleolar RNA helicase 2 OS=Homo sapiens GN=DDX21 PE=1 SV=5	DDX21_HUMAN	?	TRUE	.140218517	INF	0	0	0	0	1.43	2.94
1054	TRUE	Empty	Nucleolar transcription factor 1 OS=Homo sapiens GN=UBTF PE=1 SV=1	UBF1_HUMAN	?		0.497773146	2.2	0	1.0432	3.98	8.74	1.43	0.93978
1055	TRUE	Empty	Nucleolin OS=Homo sapiens GN=NCL PE=1 SV=3	NUCL_HUMAN	77 kDa	TRUE	.300530592	0.8	66.31	56.331	77.802	40.242	67.771	56.387
1056	TRUE	Empty	Nucleolysin TIAR OS=Homo sapiens GN=TIAL1 PE=1 SV=1	TIAR_HUMAN	?	TRUE	.348977262	0.7	4.06	3.1295	5.47	1.04	3.85	4.89
1057	TRUE	Empty	Nucleophosmin OS=Homo sapiens GN=NPM1 PE=1 SV=2	NPM_HUMAN	?	TRUE	.004724601	0.292608822	64.099	90.756	81.792	28.318	29.65	11.277
1058	TRUE	Empty	Nucleoplasmin-3 OS=Homo sapiens GN=NPM3 PE=1 SV=3	NPM3_HUMAN	19 kDa		0.145326302	2.3	0	1.0432	1.49	1.04	2.14	2.94
1059	TRUE	Empty	Nucleoporin Nup43 OS=Homo sapiens GN=NUP43 PE=1 SV=1	NUP43_HUMAN	?		0.32064321	0.2	0	3.1295	0.99746	0	0.84713	0
1060	TRUE	Empty	Nucleoprotein TPR OS=Homo sapiens GN=TPR PE=1 SV=3	TPR_HUMAN	?	TRUE	.503739509	0.6	0	25.036	10.972	6.707	8.13	4.89
1061	TRUE	Empty	Nucleoside diphosphate kinase A OS=Homo sapiens GN=NME1 PE=1 SV=1	NDKA_HUMAN	?	TRUE	.308117282	0.8	88.413	158.56	95.756	77.503	88.102	96.798
1062	TRUE	Empty	Nucleoside diphosphate kinase B OS=Homo sapiens GN=NME2 PE=1 SV=1	NDKB_HUMAN	?	TRUE	.300726417	0.7	90.623	185.68	156.6	86.445	126.22	111.83
1063	TRUE	Empty	Nucleosome assembly protein 1-like 1 OS=Homo sapiens GN=NAP1L1 PE=1 SV=1	NP1L1_HUMAN	?	TRUE	.461330116	0.9	13.262	14.604	22.942	15.65	14.401	13.157
1064	TRUE	Empty	Nucleosome assembly protein 1-like 4 OS=Homo sapiens GN=NAP1L4 PE=1 SV=1	NP1L4_HUMAN	?	TRUE	.097169685	0.3	4.06	9.86	13.964	2.57	5.0828	0.93978
1065	TRUE	Empty	O-acetyl-ADP-ribose deacetylase MACROD1 OS=Homo sapiens GN=MACROD1 PE=1 SV=2	MACD1_HUMAN	36 kDa	TRUE	.642647753	0.5	0	2.0863	0	0	0	0.93978
1066	TRUE	Empty	Obg-like ATPase 1 OS=Homo sapiens GN=OLA1 PE=1 SV=2	OLA1_HUMAN	?		0.999376516	1	15.472	16.691	28.926	13.414	27.955	19.735
1067	TRUE	Empty	OCIA domain-containing protein 1 OS=Homo sapiens GN=OCIAD1 PE=1 SV=1	OCAD1_HUMAN	?	TRUE	.024909464	5.666602761	0	1.0432	0	1.04	2.14	1.96
1068	TRUE	Empty	Omega-amidase NIT2 OS=Homo sapiens GN=NIT2 PE=1 SV=1	NIT2_HUMAN	31 kDa		0.079448412	3	0	2.0863	2.24	3.61	4.57	7.83
1069	TRUE	Empty	Opioid growth factor receptor OS=Homo sapiens GN=OGFR PE=1 SV=3	OGFR_HUMAN	?	TRUE	0.01245054	4.882180739	0	0.99746	0	2.57	1.43	0.93978
1070	TRUE	Empty	Ornithine aminotransferase, mitochondrial OS=Homo sapiens GN=OAT PE=1 SV=1	OAT_HUMAN	?	TRUE	.003651021	8.270005815	0	0	0.99746	2.09	3.85	1.96
1071	TRUE	Empty	Osteoclast-stimulating factor 1 OS=Homo sapiens GN=OSTF1 PE=1 SV=2	OSTF1_HUMAN	24 kDa		0.60051185	0.7	0	3.1295	6.22	2.57	2.14	1.96
1072	TRUE	Empty	OTU domain-containing protein 6B OS=Homo sapiens GN=OTUD6B PE=1 SV=1	OTU6B_HUMAN	?	TRUE	.373900966	INF	0	0	0	1.04	0	0
1073	TRUE	Empty	Oxysterol-binding protein 1 OS=Homo sapiens GN=OSBP PE=1 SV=1	OSBP1_HUMAN	89 kDa	TRUE	.373900966	INF	0	0	0	2.57	0	0
1074	TRUE	Empty	Palladin OS=Homo sapiens GN=PALLD PE=1 SV=3	PALLD_HUMAN	?	TRUE	.351961382	0.3	0	2.0863	0.99746	0	0	0.93978
1075	TRUE	Empty	Parafibromin OS=Homo sapiens GN=CDC73 PE=1 SV=1	CDC73_HUMAN	61 kDa		0.028213971	5.91725984	0.99746	0	0	2.57	0.84713	2.94
1076	TRUE	Empty	Paraneoplastic antigen-like protein 6B OS=Homo sapiens GN=PNMA6B PE=3 SV=1	PNM6B_HUMAN	44 kDa		0.373900966	INF	0	0	0	0	0	1.96
1077	TRUE	Empty	Paraspeckle component 1 OS=Homo sapiens GN=PSPC1 PE=1 SV=1	PSPC1_HUMAN	?	TRUE	.640270065	0.7	0	7.22	2.24	2.57	0.84713	3.91
1078	TRUE	Empty	Parkinson disease 7 domain-containing protein 1 OS=Homo sapiens GN=PDDC1 PE=1 SV=1	PDDC1_HUMAN	?		0.644277704	0.7	0	5.59	1.49	2.57	1.43	0.93978
1079	TRUE	Empty	Partitioning defective 6 homolog beta OS=Homo sapiens GN=PARD6B PE=1 SV=1	PAR6B_HUMAN	?	TRUE	.940562373	1.1	0	1.0432	0.99746	2.57	0	0
1080	TRUE	Empty	Partner of Y14 and mago OS=Homo sapiens GN=WIBG PE=1 SV=1	WIBG_HUMAN	?		0.588834218	0.4	0	0	3.98	1.04	0	0
1081	TRUE	Empty	Paxillin OS=Homo sapiens GN=PXN PE=1 SV=3	PAXI_HUMAN	?		0.572783299	0.5	0	2.0863	10.972	2.57	4.57	0

1082	TRUE	Empty	PCTP-like protein OS=Homo sapiens GN=STARD10 PE=1 SV=2	PCTL_HUMAN	33 kDa	TRUE	.879304869	1.1	4.06	7.22	6.22	2.57	10.166	7.83
1083	TRUE	Empty	PDZ and LIM domain protein 1 OS=Homo sapiens GN=PDLIM1 PE=1 SV=4	PDLI1_HUMAN	36 kDa		0.32624249	0.4	0	35.468	18.952	8.26	4.57	6.85
1084	TRUE	Empty	PDZ and LIM domain protein 5 OS=Homo sapiens GN=PDLIM5 PE=1 SV=5	PDLI5_HUMAN	?		0.261032363	0.2	0	11.475	4.73	0.74522	1.43	0.93978
1085	TRUE	Empty	PDZ and LIM domain protein 7 OS=Homo sapiens GN=PDLIM7 PE=1 SV=1	PDLI7_HUMAN	?		0.211887259	0.6	4.06	8.54	12.967	3.61	5.0828	5.87
1086	TRUE	Empty	PDZ domain-containing protein GIPC1 OS=Homo sapiens GN=GIPC1 PE=1 SV=2	GIPC1_HUMAN	?	TRUE	.317180775	0.7	2.03	5.59	5.47	2.09	2.14	3.91
1087	TRUE	Empty	Peptidyl-prolyl cis-trans isomerase A OS=Homo sapiens GN=PPIA PE=1 SV=2	PPIA_HUMAN	?	TRUE	.360411011	0.7	66.31	231.58	185.53	104.33	127.92	95.858
1088	TRUE	Empty	Peptidyl-prolyl cis-trans isomerase B OS=Homo sapiens GN=PPIB PE=1 SV=2	PPIB_HUMAN	24 kDa	TRUE	.202725881	1.7	17.683	14.604	3.98	13.414	22.025	27.254
1089	TRUE	Empty	Peptidyl-prolyl cis-trans isomerase D OS=Homo sapiens GN=PPID PE=1 SV=3	PPID_HUMAN	41 kDa	TRUE	.575026099	1.5	0	2.0863	0.99746	2.09	0.84713	0.93978
1090	TRUE	Empty	Peptidyl-prolyl cis-trans isomerase F, mitochondrial OS=Homo sapiens GN=PPIF PE=1 SV=1	PPIF_HUMAN	?	TRUE	.198389237	0.1	0	6.259	3.98	1.04	0	0
1091	TRUE	Empty	Peptidyl-prolyl cis-trans isomerase FKBP1A OS=Homo sapiens GN=FKBP1A PE=1 SV=2	FKB1A_HUMAN	12 kDa	TRUE	.213967812	0.2	0	21.907	14.962	3.61	2.14	1.96
1092	TRUE	Empty	Peptidyl-prolyl cis-trans isomerase FKBP2 OS=Homo sapiens GN=FKBP2 PE=1 SV=2	FKBP2_HUMAN	16 kDa		0.635423566	0.5	0	5.59	0	0.74522	1.43	0
1093	TRUE	Empty	Peptidyl-prolyl cis-trans isomerase FKBP3 OS=Homo sapiens GN=FKBP3 PE=1 SV=1	FKBP3_HUMAN	25 kDa		0.496614633	0.6	4.06	8.54	23.939	8.74	7.42	7.83
1094	TRUE	Empty	Peptidyl-prolyl cis-trans isomerase FKBP4 OS=Homo sapiens GN=FKBP4 PE=1 SV=3	FKBP4_HUMAN	52 kDa	TRUE	.309074375	1.4	19.893	19.82	13.964	16.395	33.885	22.555
1095	TRUE	Empty	Peptidyl-prolyl cis-trans isomerase G OS=Homo sapiens GN=PPIG PE=1 SV=2	PPIG_HUMAN	?	TRUE	.699728631	1.9	0	0	0.99746	0	0	1.96
1096	TRUE	Empty	Peptidyl-prolyl cis-trans isomerase NIMA-interacting 1 OS=Homo sapiens GN=PIN1 PE=1 SV=1	PIN1_HUMAN	18 kDa		0.230321601	0.2	0	5.59	2.24	0.74522	0	0.93978
1097	TRUE	Empty	Peptidyl-prolyl cis-trans isomerase NIMA-interacting 4 OS=Homo sapiens GN=PIN4 PE=1 SV=1	PIN4_HUMAN	?		0.153594946	0	0	2.0863	3.98	0	0	0
1098	TRUE	Empty	Peptidyl-prolyl cis-trans isomerase-like 1 OS=Homo sapiens GN=PPIL1 PE=1 SV=1	PPIL1_HUMAN	18 kDa		0.119086248	INF	0	0	0	2.57	0	1.96
1099	TRUE	Empty	Peptidyl-prolyl cis-trans isomerase-like 3 OS=Homo sapiens GN=PPIL3 PE=1 SV=1	PPIL3_HUMAN	?		0.499754298	0.2	0	3.1295	0	0.74522	0	0
1100	TRUE	Empty	Perilipin-3 OS=Homo sapiens GN=PLIN3 PE=1 SV=3	PLIN3_HUMAN	?	TRUE	.606713185	0.7	2.03	38.597	17.954	9.78	16.096	15.037
1101	TRUE	Empty	Periplakin OS=Homo sapiens GN=PPL PE=1 SV=4	PEPL_HUMAN	205 kDa	TRUE	0.70937403	0.8	4.06	19.82	14.962	5.65	15.248	12.217
1102	TRUE	Empty	Peroxiredoxin-1 OS=Homo sapiens GN=PRDX1 PE=1 SV=1	PRDX1_HUMAN	22 kDa	TRUE	.022128042	0.536773751	101.67	100.14	86.779	29.809	67.771	57.327
1103	TRUE	Empty	Peroxiredoxin-2 OS=Homo sapiens GN=PRDX2 PE=1 SV=5	PRDX2_HUMAN	?	TRUE	.001159416	0.506422972	50.837	50.072	54.86	21.611	26.261	31.013
1104	TRUE	Empty	Peroxiredoxin-4 OS=Homo sapiens GN=PRDX4 PE=1 SV=1	PRDX4_HUMAN	31 kDa	TRUE	.043593552	2.292194529	8.13	3.1295	3.98	9.78	11.86	15.037
1105	TRUE	Empty	Peroxiredoxin-5, mitochondrial OS=Homo sapiens GN=PRDX5 PE=1 SV=4	PRDX5_HUMAN	?		0.395939046	0.7	8.13	45.9	38.901	19.376	22.873	18.796
1106	TRUE	Empty	Peroxiredoxin-6 OS=Homo sapiens GN=PRDX6 PE=1 SV=3	PRDX6_HUMAN	25 kDa		0.861128577	1	26.524	38.597	36.906	31.299	38.121	30.073
1107	TRUE	Empty	Peroxisomal multifunctional enzyme type 2 OS=Homo sapiens GN=HSD17B4 PE=1 SV=3	DHB4_HUMAN	?	TRUE	.212372811	2.3	17.683	3.1295	0.99746	23.847	15.248	11.277
1108	TRUE	Empty	PERQ amino acid-rich with GYF domain-containing protein 2 OS=Homo sapiens GN=GIGYF2 PE=1 SV=1	PERQ2_HUMAN	?	TRUE	.484287573	1.7	0	3.1295	0.99746	0.74522	3.85	2.94
1109	TRUE	Empty	PEST proteolytic signal-containing nuclear protein OS=Homo sapiens GN=PCNP PE=1 SV=2	PCNP_HUMAN	?		0.591766252	2	0	0	3.98	5.17	0	1.96
1110	TRUE	Empty	PHD finger protein 14 OS=Homo sapiens GN=PHF14 PE=1 SV=2	PHF14_HUMAN	?		0.199673922	0.2	0	2.0863	1.49	0.74522	0	0

1111	TRUE	Empty	PHD finger-like domain-containing protein 5A OS=Homo sapiens GN=PHF5A PE=1 SV=1	PHF5A_HUMAN	12 kDa		0.218050734	0.2	0	8.54	5.47	2.57	0.84713	0
1112	TRUE	Empty	Phenylalanine--tRNA ligase alpha subunit OS=Homo sapiens GN=FARSA PE=1 SV=3	SYFA_HUMAN	?	TRUE	.094905451	INF	0	0	0	0.74522	0.84713	2.94
1113	TRUE	Empty	Phenylalanine--tRNA ligase beta subunit OS=Homo sapiens GN=FARSB PE=1 SV=3	SYFB_HUMAN	?		0.213714872	2.9	6.631	0	0	2.09	8.13	7.83
1114	TRUE	Empty	Phosducin-like protein 3 OS=Homo sapiens GN=PDCL3 PE=1 SV=1	PDCL3_HUMAN	28 kDa		0.362513878	0.4	0	5.59	3.98	2.57	1.43	0
1115	TRUE	Empty	Phosphate carrier protein, mitochondrial OS=Homo sapiens GN=SLC25A3 PE=1 SV=2	MPCP_HUMAN	?	TRUE	.079724614	4.1	11.052	0	0	11.178	12.707	21.615
1116	TRUE	Empty	Phosphatidylethanolamine-binding protein 1 OS=Homo sapiens GN=PEBP1 PE=1 SV=3	PEBP1_HUMAN	21 kDa		0.132389414	0.3	8.13	37.554	26.931	9.78	9.85	6.85
1117	TRUE	Empty	Phosphatidylinositol 5-phosphate 4-kinase type-2 gamma OS=Homo sapiens GN=PIP4K2C PE=1 SV=3	PI42C_HUMAN	?	TRUE	0.06853503	INF	0	0	0	0.74522	2.14	0.93978
1118	TRUE	Empty	Phosphatidylinositol-binding clathrin assembly protein OS=Homo sapiens GN=PICALM PE=1 SV=2	PICAL_HUMAN	?	TRUE	.664166928	0.7	0	3.1295	6.22	1.04	5.0828	0
1119	TRUE	Empty	Phosphoacetylglucosamine mutase OS=Homo sapiens GN=PGM3 PE=1 SV=1	AGM1_HUMAN	?		0.152478611	INF	0	0	0	1.04	0	2.94
1120	TRUE	Empty	Phosphoenolpyruvate carboxykinase [GTP], mitochondrial OS=Homo sapiens GN=PCK2 PE=1 SV=3	PCKGM_HUMAN	?	TRUE	.023355892	5.071204548	0	2.0863	1.49	8.26	4.57	7.83
1121	TRUE	Empty	Phosphoglucomutase-2 OS=Homo sapiens GN=PGM2 PE=1 SV=4	PGM2_HUMAN	?		0.673326908	0.7	0	3.1295	5.47	4.13	1.43	0
1122	TRUE	Empty	Phosphoglycerate kinase 1 OS=Homo sapiens GN=PGK1 PE=1 SV=3	PGK1_HUMAN	?	TRUE	.341500131	0.7	72.941	166.91	152.61	84.955	129.61	64.845
1123	TRUE	Empty	Phosphoglycerate mutase 1 OS=Homo sapiens GN=PGAM1 PE=1 SV=2	PGAM1_HUMAN	29 kDa	TRUE	.310998784	0.7	8.13	16.691	17.954	13.414	6.71	11.277
1124	TRUE	Empty	Phosphoglycolate phosphatase OS=Homo sapiens GN=PGP PE=1 SV=1	PGP_HUMAN	34 kDa	TRUE	.765369014	1.4	0	0	4.73	0.74522	4.57	1.96
1125	TRUE	Empty	Phosphomannomutase 2 OS=Homo sapiens GN=PMM2 PE=1 SV=1	PMM2_HUMAN	?	TRUE	.754117582	1.3	0	1.0432	1.49	2.57	1.43	0
1126	TRUE	Empty	Phosphoribosyl pyrophosphate synthase-associated protein 1 OS=Homo sapiens GN=PRPSAP1 PE=1 SV=2	KPRA_HUMAN	?	TRUE	.110633814	INF	0	0	0	2.57	0.84713	5.87
1127	TRUE	Empty	Phosphoribosyl pyrophosphate synthase-associated protein 2 OS=Homo sapiens GN=PRPSAP2 PE=1 SV=1	KPRB_HUMAN	?	TRUE	.047804212	16.65670804	0	0.99746	0	2.09	4.57	9.78
1128	TRUE	Empty	Phosphoribosylformylglycinamidine synthase OS=Homo sapiens GN=PFAS PE=1 SV=4	PUR4_HUMAN	145 kDa	TRUE	.117667821	INF	0	0	0	0.74522	3.85	0.93978
1129	TRUE	Empty	Pinin OS=Homo sapiens GN=PNN PE=1 SV=4	PININ_HUMAN	?	TRUE	.912639237	1.1	0	6.259	8.71	4.13	2.14	9.78
1130	TRUE	Empty	Pirin OS=Homo sapiens GN=PIR PE=1 SV=1	PIR_HUMAN	32 kDa	TRUE	.699728631	1.9	0	0	0.99746	0	0	1.96
1131	TRUE	Empty	PITH domain-containing protein 1 OS=Homo sapiens GN=PITHD1 PE=1 SV=1	PITH1_HUMAN	?		0.792670078	0.8	0	1.0432	2.24	1.04	0.84713	0.93978
1132	TRUE	Empty	Plasma membrane calcium-transporting ATPase 1 OS=Homo sapiens GN=ATP2B1 PE=1 SV=3	AT2B1_HUMAN	?	TRUE	.094905451	INF	0	0	0	0.74522	0.84713	2.94
1133	TRUE	Empty	Plasminogen activator inhibitor 1 RNA-binding protein OS=Homo sapiens GN=SERBP1 PE=1 SV=2	PAIRB_HUMAN	?	TRUE	.822227713	0.9	0	10.432	11.969	5.65	5.99	8.81
1134	TRUE	Empty	Plastin-1 OS=Homo sapiens GN=PLS1 PE=1 SV=2	PLSI_HUMAN	70 kDa	TRUE	.014300716	7.708680047	0.99746	0	0	2.57	1.43	3.91
1135	TRUE	Empty	Platelet-activating factor acetylhydrolase IB subunit alpha OS=Homo sapiens GN=PAFAH1B1 PE=1 SV=2	LIS1_HUMAN	?		0.445779042	0.5	0	3.1295	5.47	0.74522	0.84713	2.94
1136	TRUE	Empty	Platelet-activating factor acetylhydrolase IB subunit beta OS=Homo sapiens GN=PAFAH1B2 PE=1 SV=1	PA1B2_HUMAN	?		0.767579459	0.8	0	6.259	5.47	2.57	5.0828	2.94
1137	TRUE	Empty	Platelet-activating factor acetylhydrolase IB subunit gamma OS=Homo sapiens GN=PAFAH1B3 PE=1 SV=1	PA1B3_HUMAN	26 kDa		0.953673017	1	0	6.259	6.22	3.61	3.85	6.85
1138	TRUE	Empty	Plectin OS=Homo sapiens GN=PLEC PE=1 SV=3	PLEC_HUMAN	?	TRUE	.063192899	3.1	88.413	5.59	9.46	122.22	99.962	96.798

1139	TRUE	Empty	Pleiotropic regulator 1 OS=Homo sapiens GN=PLRG1 PE=1 SV=1	PLRG1_HUMAN	?		0.657298369	0.5	0	0	3.98	0	0	1.96
1140	TRUE	Empty	Poly [ADP-ribose] polymerase 1 OS=Homo sapiens GN=PARP1 PE=1 SV=4	PARP1_HUMAN	113 kDa		0.080292299	4.1	2.03	0	0.99746	2.57	4.57	6.85
1141	TRUE	Empty	Poly(A) polymerase alpha OS=Homo sapiens GN=PAPOLA PE=1 SV=4	PAPOA_HUMAN	?	TRUE	.008380466	6.019389249	0	0.99746	0	1.04	1.43	2.94
1142	TRUE	Empty	Poly(A)-specific ribonuclease PARN OS=Homo sapiens GN=PARN PE=1 SV=1	PARN_HUMAN	?		0.817919934	1.4	0	1.0432	0	1.04	0	0
1143	TRUE	Empty	Poly(rC)-binding protein 1 OS=Homo sapiens GN=PCBP1 PE=1 SV=2	PCBP1_HUMAN	37 kDa	TRUE	.926750344	1	15.472	40.684	51.868	32.79	45.745	32.892
1144	TRUE	Empty	Poly(rC)-binding protein 2 OS=Homo sapiens GN=PCBP2 PE=1 SV=1	PCBP2_HUMAN	?	TRUE	.659157707	1.1	13.262	27.122	25.934	20.121	27.955	25.374
1145	TRUE	Empty	Poly(U)-binding-splicing factor PUF60 OS=Homo sapiens GN=PUF60 PE=1 SV=1	PUF60_HUMAN	?		0.757340368	0.8	8.13	2.0863	2.24	4.13	2.14	4.89
1146	TRUE	Empty	Polyadenylate-binding protein 1 OS=Homo sapiens GN=PABPC1 PE=1 SV=2	PABP1_HUMAN	?	TRUE	0.48075554	0.8	24.314	55.288	67.827	32.79	44.898	38.531
1147	TRUE	Empty	Polyadenylate-binding protein 2 OS=Homo sapiens GN=PABPN1 PE=1 SV=3	PABP2_HUMAN	?		0.147672906	INF	0	0	0	0	1.43	0.93978
1148	TRUE	Empty	Polyadenylate-binding protein 4 OS=Homo sapiens GN=PABPC4 PE=1 SV=1	PABP4_HUMAN	?	TRUE	.607004199	0.7	0	21.907	26.931	12.669	14.401	7.83
1149	TRUE	Empty	Polymerase delta-interacting protein 3 OS=Homo sapiens GN=POLDIP3 PE=1 SV=2	PDIP3_HUMAN	?		0.373900966	INF	0	0	0	1.04	0	0
1150	TRUE	Empty	Polypeptide N-acetylgalactosaminyltransferase 2 OS=Homo sapiens GN=GALNT2 PE=1 SV=1	GALT2_HUMAN	?	TRUE	.094905451	INF	0	0	0	0.74522	0.84713	2.94
1151	TRUE	Empty	Polypyrimidine tract-binding protein 1 OS=Homo sapiens GN=PTBP1 PE=1 SV=1	PTBP1_HUMAN	?	TRUE	.633994976	0.8	15.472	39.641	60.845	36.516	28.803	30.073
1152	TRUE	Empty	Porphobilinogen deaminase OS=Homo sapiens GN=HMBS PE=1 SV=2	HEM3_HUMAN	?		0.817346147	1.1	2.03	6.259	7.97	4.13	6.71	6.85
1153	TRUE	Empty	Prefoldin subunit 2 OS=Homo sapiens GN=PFDN2 PE=1 SV=1	PFD2_HUMAN	17 kDa	TRUE	.089706545	0.4	8.13	22.95	13.964	4.13	3.85	8.81
1154	TRUE	Empty	Prefoldin subunit 3 OS=Homo sapiens GN=VBP1 PE=1 SV=3	PFD3_HUMAN	23 kDa	TRUE	.619745948	0.7	0	7.22	8.71	2.09	1.43	6.85
1155	TRUE	Empty	Prefoldin subunit 4 OS=Homo sapiens GN=PFDN4 PE=1 SV=1	PFD4_HUMAN	15 kDa	TRUE	.364200795	0.3	0	7.22	3.98	0	0	3.91
1156	TRUE	Empty	Prefoldin subunit 5 OS=Homo sapiens GN=PFDN5 PE=1 SV=2	PFD5_HUMAN	?		0.354643453	0.5	0	11.475	9.46	2.09	3.85	3.91
1157	TRUE	Empty	Prefoldin subunit 6 OS=Homo sapiens GN=PFDN6 PE=1 SV=1	PFD6_HUMAN	15 kDa		0.216957858	0.4	2.03	8.54	2.24	1.04	1.43	1.96
1158	TRUE	Empty	Prelamin-A/C OS=Homo sapiens GN=LMNA PE=1 SV=1	LMNA_HUMAN	?	TRUE	.149203928	0.6	57.468	122.05	118.7	66.324	48.287	66.725
1159	TRUE	Empty	Pre-mRNA 3'-end-processing factor FIP1 OS=Homo sapiens GN=FIP1L1 PE=1 SV=1	FIP1_HUMAN	?	TRUE	0.44053688	0.4	0	10.432	3.98	2.09	2.14	0.93978
1160	TRUE	Empty	Pre-mRNA-processing factor 19 OS=Homo sapiens GN=PRPF19 PE=1 SV=1	PRP19_HUMAN	55 kDa		0.529332167	1.4	0	5.59	7.97	8.26	4.57	5.87
1161	TRUE	Empty	Pre-mRNA-processing factor 40 homolog A OS=Homo sapiens GN=PRPF40A PE=1 SV=2	PR40A_HUMAN	?	TRUE	.081452559	2.3	0	2.0863	2.24	3.61	3.85	4.89
1162	TRUE	Empty	Pre-mRNA-processing factor 6 OS=Homo sapiens GN=PRPF6 PE=1 SV=1	PRP6_HUMAN	?	TRUE	.189953018	INF	0	0	0	2.57	0.84713	0
1163	TRUE	Empty	Pre-mRNA-processing-splicing factor 8 OS=Homo sapiens GN=PRPF8 PE=1 SV=2	PRP8_HUMAN	274 kDa		0.006005601	5.259592846	8.13	0	1.49	19.376	16.943	20.675
1164	TRUE	Empty	Pre-mRNA-splicing factor ATP-dependent RNA helicase DHX15 OS=Homo sapiens GN=DHX15 PE=1 SV=2	DHX15_HUMAN	91 kDa	TRUE	.143342374	2.5	8.13	2.0863	1.49	5.17	11.013	15.976
1165	TRUE	Empty	Pre-mRNA-splicing factor ISY1 homolog OS=Homo sapiens GN=ISY1 PE=1 SV=3	ISY1_HUMAN	?	TRUE	.589707208	0.6	0	3.1295	0.99746	1.04	0.84713	0

1166	TRUE	Empty	Pre-mRNA-splicing factor SPF27 OS=Homo sapiens GN=BCAS2 PE=1 SV=1	SPF27_HUMAN	26 kDa		0.057553063	3.2	0	2.0863	0.99746	4.13	2.14	2.94
1167	TRUE	Empty	Pre-mRNA-splicing regulator WTAP OS=Homo sapiens GN=WTAP PE=1 SV=2	FL2D_HUMAN	?		0.351961382	0.3	0	2.0863	0.99746	0	0	0.93978
1168	TRUE	Empty	Prenylcysteine oxidase 1 OS=Homo sapiens GN=PCYOX1 PE=1 SV=3	PCYOX_HUMAN	?		0.19309281	3	4.06	0	0	3.61	6.71	2.94
1169	TRUE	Empty	Presequence protease, mitochondrial OS=Homo sapiens GN=PITRM1 PE=1 SV=3	PREP_HUMAN	?	TRUE	.002281953	6.766587131	0	0	0.99746	2.57	1.43	2.94
1170	TRUE	Empty	PRKC apoptosis WT1 regulator protein OS=Homo sapiens GN=PAWR PE=1 SV=1	PAWR_HUMAN	37 kDa		0.232907743	0	0	1.0432	3.98	0	0	0
1171	TRUE	Empty	Probable 2-oxoglutarate dehydrogenase E1 component DHKTD1, mitochondrial OS=Homo sapiens GN=DHTKD1 PE=1 SV=2	DHTK1_HUMAN	103 kDa	TRUE	.084566581	INF	0	0	0	2.09	0.84713	0.93978
1172	TRUE	Empty	Probable aminopeptidase NPEPL1 OS=Homo sapiens GN=NPEPL1 PE=1 SV=3	PEPL1_HUMAN	?		0.600113173	0.7	0	13.561	16.957	2.57	5.99	12.217
1173	TRUE	Empty	Probable ATP-dependent RNA helicase DDX17 OS=Homo sapiens GN=DDX17 PE=1 SV=2	DDX17_HUMAN	?	TRUE	.345327057	0.7	26.524	61.547	64.835	45.458	33.038	32.892
1174	TRUE	Empty	Probable ATP-dependent RNA helicase DDX23 OS=Homo sapiens GN=DDX23 PE=1 SV=3	DDX23_HUMAN	?	TRUE	.417424624	1.7	2.03	0	2.24	1.04	2.14	4.89
1175	TRUE	Empty	Probable ATP-dependent RNA helicase DDX46 OS=Homo sapiens GN=DDX46 PE=1 SV=2	DDX46_HUMAN	117 kDa	TRUE	.351986425	1.9	11.052	0	3.98	5.65	8.13	15.037
1176	TRUE	Empty	Probable ATP-dependent RNA helicase DDX5 OS=Homo sapiens GN=DDX5 PE=1 SV=1	DDX5_HUMAN	?	TRUE	.097451013	0.7	41.996	56.331	54.86	25.337	45.745	33.832
1177	TRUE	Empty	Probable ATP-dependent RNA helicase DDX6 OS=Homo sapiens GN=DDX6 PE=1 SV=2	DDX6_HUMAN	54 kDa		0.032878705	1.802925646	4.06	3.1295	4.73	6.707	9.85	6.85
1178	TRUE	Empty	Probable cytosolic iron-sulfur protein assembly protein CIAO1 OS=Homo sapiens GN=CIAO1 PE=1 SV=1	CIAO1_HUMAN	38 kDa		0.693507807	1.6	0	0	1.49	2.57	0	0.93978
1179	TRUE	Empty	Probable global transcription activator SNF2L2 OS=Homo sapiens GN=SMARCA2 PE=1 SV=2	SMCA2_HUMAN	?	TRUE	.043064785	5.272311672	0	0	0.99746	0.74522	1.43	2.94
1180	TRUE	Empty	Probable rRNA-processing protein EBP2 OS=Homo sapiens GN=EBNA1BP2 PE=1 SV=2	EBP2_HUMAN	35 kDa	TRUE	.957772143	1	0	1.0432	3.98	0.74522	1.43	2.94
1181	TRUE	Empty	Probable tRNA N6-adenosine threonylcarbamoyltransferase OS=Homo sapiens GN=OSGEP PE=1 SV=1	OSGEP_HUMAN	36 kDa		0.043064785	5.272311672	0.99746	0	0	0.74522	1.43	2.94
1182	TRUE	Empty	Probable ubiquitin carboxyl-terminal hydrolase FAF-X OS=Homo sapiens GN=USP9X PE=1 SV=3	USP9X_HUMAN	?	TRUE	.282095346	3.4	4.06	0	0	0.74522	5.0828	9.78
1183	TRUE	Empty	Pro-cathepsin H OS=Homo sapiens GN=CTSH PE=1 SV=4	CATH_HUMAN	37 kDa		0.351961382	0.3	0	2.0863	0.99746	0	0	0.93978
1184	TRUE	Empty	Procollagen-lysine,2-oxoglutarate 5-dioxygenase 2 OS=Homo sapiens GN=PLOD2 PE=1 SV=2	PLOD2_HUMAN	?		0.130757249	INF	0	0	0	0	2.14	3.91
1185	TRUE	Empty	Profilin-1 OS=Homo sapiens GN=PFN1 PE=1 SV=2	PROF1_HUMAN	15 kDa		0.370335681	0.6	53.048	285.83	215.45	84.955	140.62	114.65
1186	TRUE	Empty	Profilin-2 OS=Homo sapiens GN=PFN2 PE=1 SV=3	PROF2_HUMAN	?		0.662805573	0.8	2.03	5.59	10.972	2.09	6.71	4.89
1187	TRUE	Empty	Programmed cell death 6-interacting protein OS=Homo sapiens GN=PDCD6IP PE=1 SV=1	PDC6I_HUMAN	?		0.007705435	5.047353903	6.631	2.0863	2.24	25.337	17.79	15.976
1188	TRUE	Empty	Programmed cell death protein 10 OS=Homo sapiens GN=PDCD10 PE=1 SV=1	PDC10_HUMAN	25 kDa	TRUE	.674725081	0.5	0	0	2.24	0.74522	0.84713	0
1189	TRUE	Empty	Programmed cell death protein 5 OS=Homo sapiens GN=PDCD5 PE=1 SV=3	PDCD5_HUMAN	?	TRUE	0.24624684	0.3	2.03	18.777	8.71	2.57	4.57	3.91
1190	TRUE	Empty	Programmed cell death protein 6 OS=Homo sapiens GN=PDCD6 PE=1 SV=1	PDCD6_HUMAN	?		0.148471018	3.3	4.06	1.0432	5.47	3.61	14.401	19.735
1191	TRUE	Empty	Prohibitin OS=Homo sapiens GN=PHB PE=1 SV=1	PHB_HUMAN	?	TRUE	.383003249	1.5	8.13	13.561	16.957	32.79	12.707	14.097
1192	TRUE	Empty	Prohibitin-2 OS=Homo sapiens GN=PHB2 PE=1 SV=2	PHB2_HUMAN	?		0.321284871	2.2	19.893	0	0.99746	17.14	20.331	8.81
1193	TRUE	Empty	Proliferating cell nuclear antigen OS=Homo sapiens GN=PCNA PE=1 SV=1	PCNA_HUMAN	29 kDa		0.163965276	2	0	10.432	8.71	11.923	10.166	16.916

1194	TRUE	Empty	Proliferation-associated protein 2G4 OS=Homo sapiens GN=PA2G4 PE=1 SV=3	PA2G4_HUMAN	?	TRUE	.824188082	0.9	19.893	47.986	34.911	46.949	29.65	17.856
1195	TRUE	Empty	Proline synthase co-transcribed bacterial homolog protein OS=Homo sapiens GN=PROSC PE=1 SV=1	PROSC_HUMAN	30 kDa		0.702826551	0.7	0	2.0863	4.73	1.04	0.84713	2.94
1196	TRUE	Empty	Proline-, glutamic acid- and leucine-rich protein 1 OS=Homo sapiens GN=PELP1 PE=1 SV=2	PELP1_HUMAN	120 kDa		0.107896076	12	0	1.0432	0	2.09	1.43	7.83
1197	TRUE	Empty	Proline-rich protein PRCC OS=Homo sapiens GN=PRCC PE=1 SV=1	PRCC_HUMAN	52 kDa		0.373900966	0	0	0	1.49	0	0	0
1198	TRUE	Empty	Prolyl endopeptidase OS=Homo sapiens GN=PREP PE=1 SV=2	PPCE_HUMAN	81 kDa		0.172534037	INF	0	0	0	2.57	5.0828	0
1199	TRUE	Empty	Prosaposin OS=Homo sapiens GN=PSAP PE=1 SV=2	SAP_HUMAN	?		0.10411851	1.5	8.13	4.1727	4.73	8.74	9.85	10.338
1200	TRUE	Empty	Prostaglandin E synthase 2 OS=Homo sapiens GN=PTGES2 PE=1 SV=1	PGES2_HUMAN	42 kDa		0.12361009	INF	0	0	0	2.57	1.43	0
1201	TRUE	Empty	Prostaglandin E synthase 3 OS=Homo sapiens GN=PTGES3 PE=1 SV=1	TEBP_HUMAN	?		0.751920473	0.8	0	18.777	23.939	5.65	14.401	15.037
1202	TRUE	Empty	Prostaglandin reductase 1 OS=Homo sapiens GN=PTGR1 PE=1 SV=2	PTGR1_HUMAN	?	TRUE	.306886912	2.8	0	2.0863	2.24	5.17	0	8.81
1203	TRUE	Empty	Prostaglandin reductase 2 OS=Homo sapiens GN=PTGR2 PE=1 SV=1	PTGR2_HUMAN	?		0.232907743	0	0	1.0432	3.98	0	0	0
1204	TRUE	Empty	Proteasomal ATPase-associated factor 1 OS=Homo sapiens GN=PAAF1 PE=1 SV=2	PAAF1_HUMAN	?	TRUE	.319930661	0.4	0	2.0863	1.49	0.74522	0.84713	0
1205	TRUE	Empty	Proteasome activator complex subunit 1 OS=Homo sapiens GN=PSME1 PE=1 SV=1	PSME1_HUMAN	?		0.002120875	2.178926293	11.052	9.86	8.71	23.847	18.637	21.615
1206	TRUE	Empty	Proteasome activator complex subunit 2 OS=Homo sapiens GN=PSME2 PE=1 SV=4	PSME2_HUMAN	27 kDa	TRUE	0.7966199	1.2	11.052	1.0432	3.98	5.65	6.71	6.85
1207	TRUE	Empty	Proteasome activator complex subunit 3 OS=Homo sapiens GN=PSME3 PE=1 SV=1	PSME3_HUMAN	?	TRUE	.184637389	2	0	2.0863	4.73	5.65	4.57	4.89
1208	TRUE	Empty	Proteasome subunit alpha type-1 OS=Homo sapiens GN=PSMA1 PE=1 SV=1	PSA1_HUMAN	?		0.198971499	0.5	4.06	15.648	12.967	7.22	5.99	2.94
1209	TRUE	Empty	Proteasome subunit alpha type-2 OS=Homo sapiens GN=PSMA2 PE=1 SV=2	PSA2_HUMAN	26 kDa		0.580429758	0.7	0	47.986	40.896	17.14	27.955	15.976
1210	TRUE	Empty	Proteasome subunit alpha type-3 OS=Homo sapiens GN=PSMA3 PE=1 SV=2	PSA3_HUMAN	?		0.428553478	0.8	17.683	9.86	18.952	10.433	11.86	15.037
1211	TRUE	Empty	Proteasome subunit alpha type-4 OS=Homo sapiens GN=PSMA4 PE=1 SV=1	PSA4_HUMAN	?	TRUE	0.183114	0.3	2.03	22.95	24.936	5.65	5.99	3.91
1212	TRUE	Empty	Proteasome subunit alpha type-5 OS=Homo sapiens GN=PSMA5 PE=1 SV=3	PSA5_HUMAN	?		0.480403508	0.7	22.103	58.418	26.931	22.357	33.038	24.434
1213	TRUE	Empty	Proteasome subunit alpha type-6 OS=Homo sapiens GN=PSMA6 PE=1 SV=1	PSA6_HUMAN	?		0.150508864	0.4	13.262	53.202	34.911	15.65	9.85	14.097
1214	TRUE	Empty	Proteasome subunit alpha type-7 OS=Homo sapiens GN=PSMA7 PE=1 SV=1	PSA7_HUMAN	?	TRUE	.051893585	0.3	13.262	22.95	33.914	7.22	5.99	7.83
1215	TRUE	Empty	Proteasome subunit beta type-1 OS=Homo sapiens GN=PSMB1 PE=1 SV=2	PSB1_HUMAN	26 kDa		0.311300197	0.6	8.13	40.684	41.893	8.26	25.414	15.976
1216	TRUE	Empty	Proteasome subunit beta type-2 OS=Homo sapiens GN=PSMB2 PE=1 SV=1	PSB2_HUMAN	23 kDa		0.062919861	0.5	13.262	27.122	25.934	11.923	11.013	8.81
1217	TRUE	Empty	Proteasome subunit beta type-3 OS=Homo sapiens GN=PSMB3 PE=1 SV=2	PSB3_HUMAN	23 kDa		0.008184827	0.494381006	17.683	22.95	17.954	10.433	11.013	7.83
1218	TRUE	Empty	Proteasome subunit beta type-4 OS=Homo sapiens GN=PSMB4 PE=1 SV=4	PSB4_HUMAN	29 kDa		0.316093054	0.6	4.06	19.82	13.964	5.65	7.42	9.78
1219	TRUE	Empty	Proteasome subunit beta type-5 OS=Homo sapiens GN=PSMB5 PE=1 SV=3	PSB5_HUMAN	?		0.433877791	0.6	8.13	30.252	14.962	3.61	8.13	20.675
1220	TRUE	Empty	Proteasome subunit beta type-6 OS=Homo sapiens GN=PSMB6 PE=1 SV=4	PSB6_HUMAN	25 kDa		0.474864947	0.9	6.631	5.59	6.22	5.17	5.99	5.87

1221	TRUE	Empty	Proteasome subunit beta type-7 OS=Homo sapiens GN=PSMB7 PE=1 SV=1	PSB7_HUMAN	?		0.368127352	0.3	0	10.432	2.24	0	3.85	0
1222	TRUE	Empty	Proteasome-associated protein ECM29 homolog OS=Homo sapiens GN=ECM29 PE=1 SV=2	ECM29_HUMAN	204 kDa		0.071487659	3.7	6.631	0	0	10.433	6.71	7.83
1223	TRUE	Empty	Protein arginine N-methyltransferase 1 OS=Homo sapiens GN=PRMT1 PE=1 SV=2	ANM1_HUMAN	?	TRUE	.061023822	4.2	4.06	2.0863	0.99746	11.178	5.0828	15.037
1224	TRUE	Empty	Protein arginine N-methyltransferase 5 OS=Homo sapiens GN=PRMT5 PE=1 SV=4	ANM5_HUMAN	?		0.003318656	7.53164807	0	1.0432	1.49	6.707	6.71	9.78
1225	TRUE	Empty	Protein argonaute-2 OS=Homo sapiens GN=AGO2 PE=1 SV=3	AGO2_HUMAN	?	TRUE	.472817258	0.5	0	2.0863	6.22	1.04	1.43	0.93978
1226	TRUE	Empty	Protein BRICK1 OS=Homo sapiens GN=BRK1 PE=1 SV=1	BRK1_HUMAN	?		0.245979005	0.3	0	3.1295	2.24	0	0.84713	0.93978
1227	TRUE	Empty	Protein BUD31 homolog OS=Homo sapiens GN=BUD31 PE=1 SV=2	BUD31_HUMAN	?	TRUE	.424502508	0.4	0	1.0432	2.24	0.74522	0.84713	0
1228	TRUE	Empty	Protein C10 OS=Homo sapiens GN=C12orf57 PE=1 SV=1	C10_HUMAN	13 kDa		0.312325166	0.4	0	7.22	9.46	1.04	2.14	2.94
1229	TRUE	Empty	Protein canopy homolog 2 OS=Homo sapiens GN=CNPY2 PE=1 SV=1	CNPY2_HUMAN	?		0.279251548	4	0	0	0.99746	1.04	2.14	0
1230	TRUE	Empty	Protein CDV3 homolog OS=Homo sapiens GN=CDV3 PE=1 SV=1	CDV3_HUMAN	?	TRUE	.154337022	0.1	0	4.1727	3.98	0	0.84713	0
1231	TRUE	Empty	Protein CutA OS=Homo sapiens GN=CUTA PE=1 SV=2	CUTA_HUMAN	?		0.38199956	0.4	0	29.209	14.962	5.17	8.13	4.89
1232	TRUE	Empty	Protein deglycase DJ-1 OS=Homo sapiens GN=PARK7 PE=1 SV=2	PARK7_HUMAN	20 kDa	TRUE	.676168381	0.7	2.03	111.62	44.886	56.637	26.261	31.013
1233	TRUE	Empty	Protein DEK OS=Homo sapiens GN=DEK PE=1 SV=1	DEK_HUMAN	?		0.123705651	0.6	4.06	6.259	4.73	3.61	4.57	0.93978
1234	TRUE	Empty	Protein diaphanous homolog 1 OS=Homo sapiens GN=DIAPH1 PE=1 SV=2	DIAP1_HUMAN	?	TRUE	.028810692	15.72383855	0.99746	0	0	2.57	5.99	7.83
1235	TRUE	Empty	Protein disulfide-isomerase A3 OS=Homo sapiens GN=PDIA3 PE=1 SV=4	PDIA3_HUMAN	57 kDa	TRUE	0.05178919	3.1	15.472	3.1295	2.24	29.809	18.637	18.796
1236	TRUE	Empty	Protein disulfide-isomerase A4 OS=Homo sapiens GN=PDIA4 PE=1 SV=2	PDIA4_HUMAN	73 kDa	TRUE	.113987506	3.7	13.262	0	0	9.78	16.096	23.495
1237	TRUE	Empty	Protein disulfide-isomerase A6 OS=Homo sapiens GN=PDIA6 PE=1 SV=1	PDIA6_HUMAN	?		0.551065016	1.5	37.575	4.1727	1.49	15.65	27.108	24.434
1238	TRUE	Empty	Protein disulfide-isomerase OS=Homo sapiens GN=P4HB PE=1 SV=3	PDIA1_HUMAN	57 kDa		0.158188948	2.1	39.786	6.259	7.97	43.223	30.497	40.411
1239	TRUE	Empty	Protein dopey-2 OS=Homo sapiens GN=DOPEY2 PE=1 SV=5	DOP2_HUMAN	?	TRUE	.009521936	4.227868787	0.99746	0	0	1.04	0.84713	1.96
1240	TRUE	Empty	Protein dpy-30 homolog OS=Homo sapiens GN=DPY30 PE=1 SV=1	DPY30_HUMAN	11 kDa		0.051417907	0.4	19.893	39.641	26.931	11.923	13.554	13.157
1241	TRUE	Empty	Protein FAM49B OS=Homo sapiens GN=FAM49B PE=1 SV=1	FA49B_HUMAN	?	TRUE	.005165388	8.735511017	2.03	0	0	6.707	5.0828	7.83
1242	TRUE	Empty	Protein FAM83H OS=Homo sapiens GN=FAM83H PE=1 SV=3	FA83H_HUMAN	127 kDa	TRUE	.040067787	18.10849558	0	0	0.99746	8.26	2.14	6.85
1243	TRUE	Empty	Protein FAM91A1 OS=Homo sapiens GN=FAM91A1 PE=1 SV=3	F91A1_HUMAN	94 kDa	TRUE	.172538338	INF	0	0	0	0.74522	1.43	0
1244	TRUE	Empty	Protein FAM98A OS=Homo sapiens GN=FAM98A PE=1 SV=1	FA98A_HUMAN	?	TRUE	.373900966	0	0	0	1.49	0	0	0
1245	TRUE	Empty	Protein FAM98B OS=Homo sapiens GN=FAM98B PE=1 SV=1	FA98B_HUMAN	?	TRUE	.207751452	2.9	0	0	1.49	2.57	2.14	0.93978
1246	TRUE	Empty	Protein flightless-1 homolog OS=Homo sapiens GN=FLII PE=1 SV=2	FLII_HUMAN	?		0.007619046	10.91081347	0	0.99746	0	2.09	5.0828	2.94
1247	TRUE	Empty	Protein HID1 OS=Homo sapiens GN=HID1 PE=1 SV=1	HID1_HUMAN	?		0.152633534	INF	0	0	0	2.57	4.57	0
1248	TRUE	Empty	Protein Hikeshi OS=Homo sapiens GN=C11orf73 PE=1 SV=2	HIKES_HUMAN	22 kDa		0.010315957	7.717903475	0	0.99746	0	1.04	3.85	2.94
1249	TRUE	Empty	Protein kinase C and casein kinase substrate in neurons protein 2 OS=Homo sapiens GN=PACSIN2 PE=1 SV=2	PACN2_HUMAN	?	TRUE	.387537566	3	0	1.0432	0.99746	1.04	0	4.89

1250	TRUE	Empty	Protein kinase C delta type OS=Homo sapiens GN=PRKCD PE=1 SV=2	KPCD_HUMAN	?	TRUE	.063437116	INF	0	0	0	3.61	1.43	0.93978
1251	TRUE	Empty	Protein kinase C-binding protein 1 OS=Homo sapiens GN=ZMYND8 PE=1 SV=2	PKCB1_HUMAN	?	TRUE	.003020465	5.926453191	0	0.99746	0	1.04	2.14	1.96
1252	TRUE	Empty	Protein lin-7 homolog C OS=Homo sapiens GN=LIN7C PE=1 SV=1	LIN7C_HUMAN	22 kDa	TRUE	.914036816	1.1	0	2.0863	1.49	0.74522	0.84713	2.94
1253	TRUE	Empty	Protein LYRIC OS=Homo sapiens GN=MTDH PE=1 SV=2	LYRIC_HUMAN	64 kDa		0.231711402	INF	0	0	0	0.74522	0	2.94
1254	TRUE	Empty	Protein MAK16 homolog OS=Homo sapiens GN=MAK16 PE=1 SV=2	MAK16_HUMAN	35 kDa		0.657291243	0.5	0	0	1.49	0	0	0.93978
1255	TRUE	Empty	Protein MEMO1 OS=Homo sapiens GN=MEMO1 PE=1 SV=1	MEMO1_HUMAN	?		0.001524133	7.420748702	0	0.99746	0	2.09	2.14	1.96
1256	TRUE	Empty	Protein MON2 homolog OS=Homo sapiens GN=MON2 PE=1 SV=3	MON2_HUMAN	?	TRUE	.009521936	4.227868787	0.99746	0	0	1.04	0.84713	1.96
1257	TRUE	Empty	Protein NDRG1 OS=Homo sapiens GN=NDRG1 PE=1 SV=1	NDRG1_HUMAN	?		0.741876826	1.3	0	7.22	1.49	8.74	3.85	0.93978
1258	TRUE	Empty	Protein NOXP20 OS=Homo sapiens GN=FAM114A1 PE=1 SV=2	NXP20_HUMAN	?	TRUE	.388505473	3.5	0	1.0432	0	0	0.84713	2.94
1259	TRUE	Empty	Protein O-GlcNAcase OS=Homo sapiens GN=MGEA5 PE=1 SV=2	OGA_HUMAN	?		0.03308882	3.48079121	0	0.99746	0	0.74522	0.84713	1.96
1260	TRUE	Empty	Protein PAXX OS=Homo sapiens GN=C9orf142 PE=1 SV=2	PAXX_HUMAN	?		0.465991698	1.5	0	2.0863	1.49	1.04	1.43	2.94
1261	TRUE	Empty	Protein PBDC1 OS=Homo sapiens GN=PBDC1 PE=1 SV=1	PBDC1_HUMAN	26 kDa		0.431683163	1.9	2.03	1.0432	1.49	0.74522	2.14	6.85
1262	TRUE	Empty	Protein phosphatase 1 regulatory subunit 12A OS=Homo sapiens GN=PPP1R12A PE=1 SV=1	MYPT1_HUMAN	?	TRUE	.649199059	0.7	0	4.1727	1.49	0.74522	1.43	1.96
1263	TRUE	Empty	Protein phosphatase 1 regulatory subunit 7 OS=Homo sapiens GN=PPP1R7 PE=1 SV=1	PP1R7_HUMAN	?	TRUE	.030278029	7.7411342	0	2.0863	0.99746	11.178	4.57	8.81
1264	TRUE	Empty	Protein phosphatase 1A OS=Homo sapiens GN=PPM1A PE=1 SV=1	PPM1A_HUMAN	?	TRUE	.887561457	1.2	0	0	2.24	0.74522	0.84713	1.96
1265	TRUE	Empty	Protein phosphatase 1B OS=Homo sapiens GN=PPM1B PE=1 SV=1	PPM1B_HUMAN	?	TRUE	0.37532752	3.2	0	0	0.99746	2.57	0	0.93978
1266	TRUE	Empty	Protein phosphatase 1G OS=Homo sapiens GN=PPM1G PE=1 SV=1	PPM1G_HUMAN	59 kDa		0.536766153	1.2	6.631	4.1727	5.47	10.433	6.71	3.91
1267	TRUE	Empty	Protein phosphatase 1H OS=Homo sapiens GN=PPM1H PE=1 SV=2	PPM1H_HUMAN	56 kDa	TRUE	.373900966	INF	0	0	0	1.04	0	0
1268	TRUE	Empty	Protein phosphatase inhibitor 2-like protein 3 OS=Homo sapiens GN=PPP1R2P3 PE=1 SV=1	IPP2M_HUMAN	23 kDa	TRUE	.364606702	3.6	0	0	1.49	5.65	0	1.96
1269	TRUE	Empty	Protein phosphatase methylesterase 1 OS=Homo sapiens GN=PPME1 PE=1 SV=3	PPME1_HUMAN	?		0.009491394	8.260782387	0	0.99746	0	3.61	1.43	2.94
1270	TRUE	Empty	Protein phosphatase Slingshot homolog 3 OS=Homo sapiens GN=SSH3 PE=1 SV=2	SSH3_HUMAN	?	TRUE	0.00734386	8.16784633	0	0	0.99746	3.61	2.14	1.96
1271	TRUE	Empty	Protein PML OS=Homo sapiens GN=PML PE=1 SV=3	PML_HUMAN	?	TRUE	.298511407	INF	0	0	0	7.22	0	0.93978
1272	TRUE	Empty	Protein PRRC1 OS=Homo sapiens GN=PRRC1 PE=1 SV=1	PRRC1_HUMAN	?		0.339350852	0.3	2.03	22.95	3.98	3.61	0.84713	2.94
1273	TRUE	Empty	Protein PRRC2A OS=Homo sapiens GN=PRRC2A PE=1 SV=3	PRC2A_HUMAN	?	TRUE	.373900966	0	0	0	1.49	0	0	0
1274	TRUE	Empty	Protein PRRC2B OS=Homo sapiens GN=PRRC2B PE=1 SV=2	PRC2B_HUMAN	?	TRUE	.999738023	1	0	2.0863	0.99746	2.57	0.84713	0
1275	TRUE	Empty	Protein RCC2 OS=Homo sapiens GN=RCC2 PE=1 SV=2	RCC2_HUMAN	56 kDa		0.245505962	1.8	17.683	2.0863	6.22	14.159	20.331	13.157
1276	TRUE	Empty	Protein RTF2 homolog OS=Homo sapiens GN=RTFDC1 PE=1 SV=3	RTF2_HUMAN	34 kDa		0.506553997	0.2	0	0	2.24	0.74522	0	0
1277	TRUE	Empty	Protein S100-A11 OS=Homo sapiens GN=S100A11 PE=1 SV=2	S10AB_HUMAN	12 kDa		0.623086548	0.8	110.52	232.63	224.43	299.58	70.312	57.327
1278	TRUE	Empty	Protein S100-A13 OS=Homo sapiens GN=S100A13 PE=1 SV=1	S10AD_HUMAN	11 kDa		0.87014305	0.9	2.03	10.432	15.959	9.78	10.166	6.85
1279	TRUE	Empty	Protein S100-A16 OS=Homo sapiens GN=S100A16 PE=1 SV=1	S10AG_HUMAN	12 kDa		0.885364353	1.1	2.03	2.0863	4.73	6.707	2.14	0.93978
1280	TRUE	Empty	Protein S100-P OS=Homo sapiens GN=S100P PE=1 SV=2	S100P_HUMAN	10 kDa		0.005108112	0.290191484	15.472	15.648	20.947	3.61	7.42	3.91
1281	TRUE	Empty	Protein SEC13 homolog OS=Homo sapiens GN=SEC13 PE=1 SV=3	SEC13_HUMAN	?		0.292665146	26	0	0	0.99746	22.357	1.43	1.96

1282	TRUE	Empty	Protein sel-1 homolog 1 OS=Homo sapiens GN=SEL1L PE=1 SV=3	SE1L1_HUMAN	?		0.184606477	INF	0	0	0	0.74522	0	1.96
1283	TRUE	Empty	Protein SET OS=Homo sapiens GN=SET PE=1 SV=3	SET_HUMAN	?	TRUE	.533267968	1.2	17.683	30.252	24.936	17.885	35.58	32.892
1284	TRUE	Empty	Protein SGT1 homolog OS=Homo sapiens GN=SUGT1 PE=1 SV=3	SGT1_HUMAN	?		0.092150156	2.9	0	2.0863	0.99746	2.09	4.57	1.96
1285	TRUE	Empty	Protein SON OS=Homo sapiens GN=SON PE=1 SV=4	SON_HUMAN	?	TRUE	.177767042	INF	0	0	0	2.57	0	0.93978
1286	TRUE	Empty	Protein TBRG4 OS=Homo sapiens GN=TBRG4 PE=1 SV=1	TBRG4_HUMAN	?		0.136122783	INF	0	0	0	2.09	0	1.96
1287	TRUE	Empty	Protein TFG OS=Homo sapiens GN=TFG PE=1 SV=2	TFG_HUMAN	?		0.153594946	0	0	2.0863	3.98	0	0	0
1288	TRUE	Empty	Protein transport protein Sec16A OS=Homo sapiens GN=SEC16A PE=1 SV=3	SC16A_HUMAN	?	TRUE	.024287925	4.98423997	0	0.99746	0	1.04	2.14	0.93978
1289	TRUE	Empty	Protein transport protein Sec23A OS=Homo sapiens GN=SEC23A PE=1 SV=2	SC23A_HUMAN	?	TRUE	.671687192	1.4	19.893	4.1727	0.99746	7.22	7.42	19.735
1290	TRUE	Empty	Protein transport protein Sec23B OS=Homo sapiens GN=SEC23B PE=1 SV=2	SC23B_HUMAN	86 kDa	TRUE	.001900304	17.56581718	0	0.99746	0	6.707	4.57	6.85
1291	TRUE	Empty	Protein transport protein Sec24C OS=Homo sapiens GN=SEC24C PE=1 SV=3	SC24C_HUMAN	?	TRUE	.002199069	21.04655826	0	0.99746	0	7.22	5.0828	8.81
1292	TRUE	Empty	Protein transport protein Sec31A OS=Homo sapiens GN=SEC31A PE=1 SV=3	SC31A_HUMAN	?	TRUE	.087730141	4.8	2.03	0	1.49	2.57	8.13	9.78
1293	TRUE	Empty	Protein unc-45 homolog A OS=Homo sapiens GN=UNC45A PE=1 SV=1	UN45A_HUMAN	?		0.931959935	1.1	13.262	0	0.99746	2.57	7.42	5.87
1294	TRUE	Empty	Protein VPRBP OS=Homo sapiens GN=VPRBP PE=1 SV=3	VPRBP_HUMAN	?	TRUE	.136121777	INF	0	0	0	1.04	0	0.93978
1295	TRUE	Empty	Protein-glutamate O-methyltransferase OS=Homo sapiens GN=ARMT1 PE=1 SV=1	ARMT1_HUMAN	51 kDa		0.136121777	INF	0	0	0	1.04	0	0.93978
1296	TRUE	Empty	Protein-glutamine gamma-glutamyltransferase 2 OS=Homo sapiens GN=TGM2 PE=1 SV=2	TGM2_HUMAN	?	TRUE	.036726114	4.302764331	4.06	0	0	7.22	5.99	5.87
1297	TRUE	Empty	Protein-L-isoaspartate(D-aspartate) O-methyltransferase OS=Homo sapiens GN=PCMT1 PE=1 SV=4	PIMT_HUMAN	?		0.529563838	0.8	4.06	7.22	4.73	6.707	5.0828	0.93978
1298	TRUE	Empty	Pseudouridine-5'-phosphatase OS=Homo sapiens GN=HDHD1 PE=1 SV=3	HDHD1_HUMAN	?		0.84210842	1.1	0	1.0432	1.49	0.74522	0.84713	1.96
1299	TRUE	Empty	Pseudouridylate synthase 7 homolog OS=Homo sapiens GN=PUS7 PE=1 SV=2	PUS7_HUMAN	75 kDa		0.335303639	1.6	2.03	0	1.49	2.57	1.43	2.94
1300	TRUE	Empty	Pterin-4-alpha-carbinolamine dehydratase OS=Homo sapiens GN=PCBD1 PE=1 SV=2	PHS_HUMAN	12 kDa		0.443382579	0.5	0	6.259	5.47	2.09	3.85	0
1301	TRUE	Empty	Purine nucleoside phosphorylase OS=Homo sapiens GN=PNP PE=1 SV=2	PNPH_HUMAN	32 kDa	TRUE	.909133008	0.9	4.06	31.295	17.954	12.669	22.025	15.976
1302	TRUE	Empty	Puromycin-sensitive aminopeptidase OS=Homo sapiens GN=NPEPPS PE=1 SV=2	PSA_HUMAN	?	TRUE	.154484089	2.1	19.893	2.0863	4.73	16.395	22.025	18.796
1303	TRUE	Empty	Putative deoxyribonuclease TATDN1 OS=Homo sapiens GN=TATDN1 PE=1 SV=2	TATD1_HUMAN	?		0.895168508	1.1	0	3.1295	6.22	2.09	4.57	3.91
1304	TRUE	Empty	Putative elongation factor 1-alpha-like 3 OS=Homo sapiens GN=EEF1A1P5 PE=5 SV=1	EF1A3_HUMAN	50 kDa	TRUE	.394209207	1.4	258.61	163.78	57.853	315.23	168.58	204.87
1305	TRUE	Empty	Putative helicase MOV-10 OS=Homo sapiens GN=MOV10 PE=1 SV=2	MOV10_HUMAN	?		0.08423303	7.9	0	0	0.99746	0.74522	3.85	3.91
1306	TRUE	Empty	Putative oxidoreductase GLYR1 OS=Homo sapiens GN=GLYR1 PE=1 SV=3	GLYR1_HUMAN	?	TRUE	.373900966	INF	0	0	0	0	3.85	0
1307	TRUE	Empty	Putative RNA-binding protein 15 OS=Homo sapiens GN=RBM15 PE=1 SV=2	RBM15_HUMAN	?	TRUE	.273088108	0.4	2.03	2.0863	3.98	0	3.85	0
1308	TRUE	Empty	Putative RNA-binding protein Luc7-like 1 OS=Homo sapiens GN=LUC7L PE=1 SV=1	LUC7L_HUMAN	?	TRUE	.836458914	0.9	2.03	2.0863	5.47	2.57	3.85	3.91
1309	TRUE	Empty	Putative RNA-binding protein Luc7-like 2 OS=Homo sapiens GN=LUC7L2 PE=1 SV=2	LC7L2_HUMAN	?	TRUE	.590404329	1.3	2.03	10.432	5.47	5.65	6.71	12.217
1310	TRUE	Empty	Putative small nuclear ribonucleoprotein G-like protein 15 OS=Homo sapiens GN=SNRPGP15 PE=5 SV=2	RUXGL_HUMAN	9 kDa	TRUE	.409914111	0.6	2.03	10.432	3.98	2.57	5.0828	1.96

1311	TRUE	Empty	Pyridoxal kinase OS=Homo sapiens GN=PDXK PE=1 SV=1	PDXK_HUMAN	?		0.053434523	0.8	15.472	17.734	19.949	12.669	15.248	12.217
1312	TRUE	Empty	Pyridoxal-dependent decarboxylase domain-containing protein 1 OS=Homo sapiens GN=PDXDC1 PE=1 SV=2	PDXD1_HUMAN	?		0.058249636	16	2.03	0	0	6.707	8.13	19.735
1313	TRUE	Empty	Pyridoxine-5'-phosphate oxidase OS=Homo sapiens GN=PNPO PE=1 SV=1	PNPO_HUMAN	?		0.282866176	0.2	0	9.86	23.939	2.09	2.14	1.96
1314	TRUE	Empty	Pyrroline-5-carboxylate reductase 3 OS=Homo sapiens GN=PYCRL PE=1 SV=3	P5CR3_HUMAN	?		0.75684189	1.2	0	5.59	2.24	4.13	1.43	3.91
1315	TRUE	Empty	Pyruvate carboxylase, mitochondrial OS=Homo sapiens GN=PC PE=1 SV=2	PYC_HUMAN	?	TRUE	.117185082	INF	0	0	0	0	2.14	2.94
1316	TRUE	Empty	Pyruvate dehydrogenase E1 component subunit alpha, somatic form, mitochondrial OS=Homo sapiens GN=PDHA1 PE=1 SV=3	ODPA_HUMAN	?	TRUE	.036881472	1.81383015	2.03	3.1295	1.49	5.65	3.85	4.89
1317	TRUE	Empty	Pyruvate dehydrogenase E1 component subunit beta, mitochondrial OS=Homo sapiens GN=PDHB PE=1 SV=3	ODPB_HUMAN	?		0.349076148	2.3	4.06	1.0432	0	2.09	7.42	1.96
1318	TRUE	Empty	Pyruvate kinase PKM OS=Homo sapiens GN=PKM PE=1 SV=4	KPYM_HUMAN	?	TRUE	.009968533	1.716801796	174.62	109.53	116.7	234	229.57	224.61
1319	TRUE	Empty	Rab GDP dissociation inhibitor alpha OS=Homo sapiens GN=GDI1 PE=1 SV=2	GDIA_HUMAN	51 kDa	TRUE	.944735626	1	37.575	9.86	22.942	20.121	26.261	25.374
1320	TRUE	Empty	Rab GDP dissociation inhibitor beta OS=Homo sapiens GN=GDI2 PE=1 SV=2	GDIB_HUMAN	?	TRUE	0.16259127	1.6	50.837	16.691	36.906	46.204	64.382	51.688
1321	TRUE	Empty	Rab GTPase-activating protein 1 OS=Homo sapiens GN=RABGAP1 PE=1 SV=3	RBGP1_HUMAN	?	TRUE	.059107988	INF	0	0	0	1.04	3.85	0.93978
1322	TRUE	Empty	Rab9 effector protein with kelch motifs OS=Homo sapiens GN=RABEPK PE=1 SV=1	RABEK_HUMAN	?		0.373900966	0	0	5.59	0	0	0	0
1323	TRUE	Empty	Rab-like protein 6 OS=Homo sapiens GN=RABL6 PE=1 SV=2	RABL6_HUMAN	?		0.491737711	0.6	0	2.0863	1.49	0.74522	0.84713	0.93978
1324	TRUE	Empty	RAC-alpha serine/threonine-protein kinase OS=Homo sapiens GN=AKT1 PE=1 SV=2	AKT1_HUMAN	?	TRUE	.373900966	INF	0	0	0	2.57	0	0
1325	TRUE	Empty	Radixin OS=Homo sapiens GN=RDX PE=1 SV=1	RADI_HUMAN	?	TRUE	.614564278	0.9	33.155	14.604	24.936	15.65	26.261	20.675
1326	TRUE	Empty	Ragulator complex protein LAMTOR1 OS=Homo sapiens GN=LAMTOR1 PE=1 SV=2	LTOR1_HUMAN	18 kDa		0.261417449	INF	0	0	0	0.74522	0	3.91
1327	TRUE	Empty	Ragulator complex protein LAMTOR2 OS=Homo sapiens GN=LAMTOR2 PE=1 SV=1	LTOR2_HUMAN	?		0.605622517	0.7	0	4.1727	5.47	1.04	1.43	3.91
1328	TRUE	Empty	Ran GTPase-activating protein 1 OS=Homo sapiens GN=RANGAP1 PE=1 SV=1	RAGP1_HUMAN	64 kDa	TRUE	.000505265	1.862973854	2.03	2.0863	1.49	3.61	4.57	3.91
1329	TRUE	Empty	Ran-specific GTPase-activating protein OS=Homo sapiens GN=RANBP1 PE=1 SV=1	RANG_HUMAN	?		0.269361398	0.7	4.06	8.54	4.73	3.61	2.14	5.87
1330	TRUE	Empty	Ras GTPase-activating protein-binding protein 1 OS=Homo sapiens GN=G3BP1 PE=1 SV=1	G3BP1_HUMAN	?	TRUE	.399202426	0.6	2.03	25.036	16.957	7.22	10.166	7.83
1331	TRUE	Empty	Ras GTPase-activating protein-binding protein 2 OS=Homo sapiens GN=G3BP2 PE=1 SV=2	G3BP2_HUMAN	?	TRUE	.596029125	0.6	0	2.0863	1.49	0	1.43	0.93978
1332	TRUE	Empty	Ras GTPase-activating-like protein IQGAP1 OS=Homo sapiens GN=IQGAP1 PE=1 SV=1	IQGA1_HUMAN	189 kDa	TRUE	.014424286	2.662446077	11.052	3.1295	7.97	16.395	22.873	19.735
1333	TRUE	Empty	Ras-related C3 botulinum toxin substrate 1 OS=Homo sapiens GN=RAC1 PE=1 SV=1	RAC1_HUMAN	?	TRUE	.002569388	13.84930982	0	1.0432	0	3.61	5.0828	5.87
1334	TRUE	Empty	Ras-related GTP-binding protein A OS=Homo sapiens GN=RRAGA PE=1 SV=1	RRAGA_HUMAN	37 kDa	TRUE	.889987491	0.9	0	3.1295	3.98	2.09	1.43	1.96
1335	TRUE	Empty	Ras-related protein Rab-10 OS=Homo sapiens GN=RAB10 PE=1 SV=1	RAB10_HUMAN	23 kDa	TRUE	.514775693	1.8	17.683	0	1.49	5.17	7.42	22.555
1336	TRUE	Empty	Ras-related protein Rab-11A OS=Homo sapiens GN=RAB11A PE=1 SV=3	RB11A_HUMAN	?		0.005441067	2.233254261	4.06	2.0863	3.98	7.22	8.13	7.83
1337	TRUE	Empty	Ras-related protein Rab-14 OS=Homo sapiens GN=RAB14 PE=1 SV=4	RAB14_HUMAN	24 kDa	TRUE	.192368921	3.3	19.893	0	0.99746	13.414	16.943	39.471

1338	TRUE	Empty	Ras-related protein Rab-18 OS=Homo sapiens GN=RAB18 PE=1 SV=1	RAB18_HUMAN	?		0.055057501	INF	0	0	0	0.74522	2.14	3.91
1339	TRUE	Empty	Ras-related protein Rab-1A OS=Homo sapiens GN=RAB1A PE=1 SV=3	RAB1A_HUMAN	?	TRUE	.371623559	2.2	15.472	0	0.99746	7.22	8.13	19.735
1340	TRUE	Empty	Ras-related protein Rab-1B OS=Homo sapiens GN=RAB1B PE=1 SV=1	RAB1B_HUMAN	22 kDa	TRUE	.251700357	2.7	15.472	0	0	12.669	7.42	21.615
1341	TRUE	Empty	Ras-related protein Rab-21 OS=Homo sapiens GN=RAB21 PE=1 SV=3	RAB21_HUMAN	24 kDa	TRUE	.082848332	INF	0	0	0	1.04	0.84713	3.91
1342	TRUE	Empty	Ras-related protein Rab-2A OS=Homo sapiens GN=RAB2A PE=1 SV=1	RAB2A_HUMAN	?	TRUE	.033087169	13.92306458	0	0.99746	0	2.09	3.85	7.83
1343	TRUE	Empty	Ras-related protein Rab-5A OS=Homo sapiens GN=RAB5A PE=1 SV=2	RAB5A_HUMAN	?	TRUE	.293072575	2.5	6.631	0	0	2.09	5.0828	8.81
1344	TRUE	Empty	Ras-related protein Rab-5C OS=Homo sapiens GN=RAB5C PE=1 SV=2	RAB5C_HUMAN	?	TRUE	0.22467013	2.7	8.13	0	0	4.13	11.013	8.81
1345	TRUE	Empty	Ras-related protein Rab-6A OS=Homo sapiens GN=RAB6A PE=1 SV=3	RAB6A_HUMAN	?	TRUE	.410600111	2.9	11.052	0	0	3.61	4.57	24.434
1346	TRUE	Empty	Ras-related protein Rab-7a OS=Homo sapiens GN=RAB7A PE=1 SV=1	RAB7A_HUMAN	23 kDa		0.013735804	6.749129078	4.06	0	0	7.22	10.166	12.217
1347	TRUE	Empty	Ras-related protein Rap-1b OS=Homo sapiens GN=RAP1B PE=1 SV=1	RAP1B_HUMAN	?	TRUE	.689973084	1.2	8.13	2.0863	7.97	4.13	9.85	8.81
1348	TRUE	Empty	Receptor expression-enhancing protein 5 OS=Homo sapiens GN=REEP5 PE=1 SV=3	REEP5_HUMAN	?	TRUE	.204979476	3.2	4.06	0	0	6.707	1.43	5.87
1349	TRUE	Empty	Receptor expression-enhancing protein 6 OS=Homo sapiens GN=REEP6 PE=1 SV=1	REEP6_HUMAN	21 kDa		0.983254095	1	4.06	0	0	0.74522	1.43	1.96
1350	TRUE	Empty	Receptor-type tyrosine-protein phosphatase F OS=Homo sapiens GN=PTPRF PE=1 SV=2	PTPRF_HUMAN	?	TRUE	.178329558	0	0	2.0863	4.73	0	0	0
1351	TRUE	Empty	Receptor-type tyrosine-protein phosphatase kappa OS=Homo sapiens GN=PTPRK PE=1 SV=2	PTPRK_HUMAN	?	TRUE	0.32064321	0.2	0	3.1295	0.99746	0	0.84713	0
1352	TRUE	Empty	Regulation of nuclear pre-mRNA domain-containing protein 1B OS=Homo sapiens GN=RPRD1B PE=1 SV=1	RPR1B_HUMAN	37 kDa	TRUE	.112328326	2.2	0	2.0863	3.98	3.61	5.0828	4.89
1353	TRUE	Empty	Regulation of nuclear pre-mRNA domain-containing protein 2 OS=Homo sapiens GN=RPRD2 PE=1 SV=1	RPRD2_HUMAN	?		0.002281953	6.766587131	0	0.99746	0	2.57	1.43	2.94
1354	TRUE	Empty	Regulator of chromosome condensation OS=Homo sapiens GN=RCC1 PE=1 SV=1	RCC1_HUMAN	?		0.509594459	0.8	4.06	7.22	6.22	6.707	5.99	2.94
1355	TRUE	Empty	Regulator of microtubule dynamics protein 1 OS=Homo sapiens GN=RMDN1 PE=1 SV=1	RMD1_HUMAN	?	TRUE	.036028473	3.329176746	2.03	0	0.99746	4.13	3.85	2.94
1356	TRUE	Empty	Regulator of nonsense transcripts 1 OS=Homo sapiens GN=UPF1 PE=1 SV=2	RENT1_HUMAN	?		0.060183159	2.6	0	9.86	11.969	14.159	22.873	18.796
1357	TRUE	Empty	RelA-associated inhibitor OS=Homo sapiens GN=PPP1R13L PE=1 SV=4	IASPP_HUMAN	89 kDa	TRUE	.116315977	0	0	2.0863	1.49	0	0	0
1358	TRUE	Empty	Replication factor C subunit 5 OS=Homo sapiens GN=RFC5 PE=1 SV=1	RFC5_HUMAN	?	TRUE	.136121777	INF	0	0	0	1.04	0	0.93978
1359	TRUE	Empty	Replication protein A 14 kDa subunit OS=Homo sapiens GN=RPA3 PE=1 SV=1	RFA3_HUMAN	14 kDa		0.73851057	0.8	0	15.648	5.47	5.65	6.71	4.89
1360	TRUE	Empty	Replication protein A 70 kDa DNA-binding subunit OS=Homo sapiens GN=RPA1 PE=1 SV=2	RFA1_HUMAN	68 kDa	TRUE	.285577572	INF	0	0	0	0	0.84713	5.87
1361	TRUE	Empty	Reticulocalbin-2 OS=Homo sapiens GN=RCN2 PE=1 SV=1	RCN2_HUMAN	?		0.177767042	INF	0	0	0	2.57	0	0.93978
1362	TRUE	Empty	Reticulon-4 OS=Homo sapiens GN=RTN4 PE=1 SV=2	RTN4_HUMAN	?		0.108419887	3.6	6.631	0	0	11.178	5.99	6.85
1363	TRUE	Empty	Retinoblastoma-binding protein 5 OS=Homo sapiens GN=RBBP5 PE=1 SV=2	RBBP5_HUMAN	?		0.098392517	0.4	2.03	4.1727	2.24	0.74522	2.14	0
1364	TRUE	Empty	RHG30_HUMAN-DECOY	RHG30_HUMAN-DE	?	TRUE	.440918012	0.6	4.06	2.0863	1.49	0	3.85	1.96
1365	TRUE	Empty	Rho GDP-dissociation inhibitor 1 OS=Homo sapiens GN=ARHGDIA PE=1 SV=3	GDIR1_HUMAN	?		0.510421604	0.7	13.262	4.1727	8.71	2.57	8.13	8.81

1366	TRUE	Empty	Rho GTPase-activating protein 1 OS=Homo sapiens GN=ARHGAP1 PE=1 SV=1	RHG01_HUMAN	50 kDa	TRUE	.003698302	15.58849478	0	0.99746	0	6.707	5.0828	3.91
1367	TRUE	Empty	Rho-associated protein kinase 2 OS=Homo sapiens GN=ROCK2 PE=1 SV=4	ROCK2_HUMAN	161 kDa	TRUE	.169759736	INF	0	0	0	0	0.84713	1.96
1368	TRUE	Empty	Rhomboid domain-containing protein 2 OS=Homo sapiens GN=RHBDD2 PE=2 SV=2	RHBD2_HUMAN	?	TRUE	.896568956	0.8	6.631	0	0	2.57	3.85	0
1369	TRUE	Empty	Ribonuclease inhibitor OS=Homo sapiens GN=RNH1 PE=1 SV=2	RINI_HUMAN	50 kDa	TRUE	.008070508	1.805263787	11.052	10.432	6.22	15.65	16.943	18.796
1370	TRUE	Empty	Ribonuclease P protein subunit p40 OS=Homo sapiens GN=RPP40 PE=1 SV=3	RPP40_HUMAN	?		0.172538338	INF	0	0	0	0.74522	1.43	0
1371	TRUE	Empty	Ribonuclease UK114 OS=Homo sapiens GN=HRSP12 PE=1 SV=1	UK114_HUMAN	14 kDa		0.003809927	0.3238427	6.631	6.259	7.97	1.04	3.85	1.96
1372	TRUE	Empty	Ribonucleoprotein PTB-binding 1 OS=Homo sapiens GN=RAVER1 PE=1 SV=1	RAVR1_HUMAN	?		0.151042322	2.4	0	4.1727	1.49	2.09	5.0828	6.85
1373	TRUE	Empty	Ribonucleoside-diphosphate reductase large subunit OS=Homo sapiens GN=RRM1 PE=1 SV=1	RIR1_HUMAN	90 kDa	TRUE	.147672906	INF	0	0	0	0	1.43	0.93978
1374	TRUE	Empty	Ribonucleoside-diphosphate reductase subunit M2 B OS=Homo sapiens GN=RRM2B PE=1 SV=1	RIR2B_HUMAN	?	TRUE	.652238853	1.4	0	3.1295	7.97	2.57	8.13	4.89
1375	TRUE	Empty	Ribonucleoside-diphosphate reductase subunit M2 OS=Homo sapiens GN=RRM2 PE=1 SV=1	RIR2_HUMAN	?	TRUE	0.69841102	1.4	0	1.0432	6.22	1.04	5.99	3.91
1376	TRUE	Empty	Ribose-phosphate pyrophosphokinase 1 OS=Homo sapiens GN=PRPS1 PE=1 SV=2	PRPS1_HUMAN	?	TRUE	.080775509	INF	0	0	0	5.65	2.14	0.93978
1377	TRUE	Empty	Ribose-phosphate pyrophosphokinase 2 OS=Homo sapiens GN=PRPS2 PE=1 SV=2	PRPS2_HUMAN	?	TRUE	.029630168	15.38407555	0	0	0.99746	8.74	3.85	3.91
1378	TRUE	Empty	Ribosomal L1 domain-containing protein 1 OS=Homo sapiens GN=RSL1D1 PE=1 SV=3	RL1D1_HUMAN	?	TRUE	.088050338	3.3	0	0	1.49	2.57	2.14	1.96
1379	TRUE	Empty	Ribosomal protein S6 kinase beta-1 OS=Homo sapiens GN=RPS6KB1 PE=1 SV=2	KS6B1_HUMAN	?	TRUE	.040108886	3.081788843	2.03	1.0432	0	3.61	2.14	3.91
1380	TRUE	Empty	Ribosome biogenesis protein BRX1 homolog OS=Homo sapiens GN=BRIX1 PE=1 SV=2	BRX1_HUMAN	41 kDa		0.121394667	INF	0	0	0	1.04	0	1.96
1381	TRUE	Empty	Ribosome-binding protein 1 OS=Homo sapiens GN=RRBP1 PE=1 SV=4	RRBP1_HUMAN	?	TRUE	0.21599088	5.1	0	0	0.99746	2.57	0	2.94
1382	TRUE	Empty	RING finger protein 113A OS=Homo sapiens GN=RNF113A PE=1 SV=1	R113A_HUMAN	39 kDa	TRUE	.943452794	0.9	0	2.0863	1.49	2.09	0.84713	0
1383	TRUE	Empty	RNA-binding motif protein, X chromosome OS=Homo sapiens GN=RBMX PE=1 SV=3	RBMX_HUMAN	?	TRUE	.312246472	1.7	2.03	5.59	20.947	14.159	17.79	16.916
1384	TRUE	Empty	RNA-binding motif, single-stranded-interacting protein 1 OS=Homo sapiens GN=RBMS1 PE=1 SV=3	RBMS1_HUMAN	?	TRUE	.443532528	0.4	0	1.0432	2.24	0.74522	0	0.93978
1385	TRUE	Empty	RNA-binding protein 10 OS=Homo sapiens GN=RBM10 PE=1 SV=3	RBM10_HUMAN	?	TRUE	.527021212	0.6	0	3.1295	4.73	2.09	0.84713	0.93978
1386	TRUE	Empty	RNA-binding protein 12 OS=Homo sapiens GN=RBM12 PE=1 SV=1	RBM12_HUMAN	97 kDa		0.997985727	1	0	5.59	0.99746	0.74522	1.43	3.91
1387	TRUE	Empty	RNA-binding protein 14 OS=Homo sapiens GN=RBM14 PE=1 SV=2	RBM14_HUMAN	?		0.837727931	0.9	0	15.648	17.954	10.433	11.86	7.83
1388	TRUE	Empty	RNA-binding protein 25 OS=Homo sapiens GN=RBM25 PE=1 SV=3	RBM25_HUMAN	?	TRUE	0.03298905	4.932903028	2.03	1.0432	0	3.61	7.42	4.89
1389	TRUE	Empty	RNA-binding protein 3 OS=Homo sapiens GN=RBM3 PE=1 SV=1	RBM3_HUMAN	17 kDa	TRUE	.867356704	1.1	6.631	19.82	0.99746	8.26	9.85	12.217
1390	TRUE	Empty	RNA-binding protein 39 OS=Homo sapiens GN=RBM39 PE=1 SV=2	RBM39_HUMAN	?	TRUE	.847477452	1.1	4.06	12.518	11.969	14.904	5.99	10.338
1391	TRUE	Empty	RNA-binding protein 4 OS=Homo sapiens GN=RBM4 PE=1 SV=1	RBM4_HUMAN	?	TRUE	.383231094	1.7	0	6.259	6.22	11.178	5.0828	5.87

1392	TRUE	Empty	RNA-binding protein 8A OS=Homo sapiens GN=RBM8A PE=1 SV=1	RBM8A_HUMAN	?		0.745622251	0.8	0	4.1727	4.73	2.09	1.43	2.94
1393	TRUE	Empty	RNA-binding protein EWS OS=Homo sapiens GN=EWSR1 PE=1 SV=1	EWS_HUMAN	?	TRUE	.454951932	0.7	4.06	5.59	9.46	1.04	8.13	2.94
1394	TRUE	Empty	RNA-binding protein FUS OS=Homo sapiens GN=FUS PE=1 SV=1	FUS_HUMAN	?	TRUE	.366023187	0.5	6.631	30.252	48.875	18.63	8.13	19.735
1395	TRUE	Empty	RNA-binding protein Musashi homolog 2 OS=Homo sapiens GN=MSI2 PE=1 SV=1	MSI2H_HUMAN	?	TRUE	.386382921	0.4	0	6.259	3.98	0.74522	3.85	0
1396	TRUE	Empty	RNA-binding protein Raly OS=Homo sapiens GN=RALY PE=1 SV=1	RALY_HUMAN	?		0.093700402	1.2	4.06	6.259	5.47	7.22	6.71	6.85
1397	TRUE	Empty	RNA-binding protein with serine-rich domain 1 OS=Homo sapiens GN=RNPS1 PE=1 SV=1	RNPS1_HUMAN	?	TRUE	.589536314	0.6	0	6.259	2.24	1.04	3.85	0.93978
1398	TRUE	Empty	rRNA 2'-O-methyltransferase fibrillarin OS=Homo sapiens GN=FBL PE=1 SV=2	FBRL_HUMAN	34 kDa	TRUE	.150322159	3.5	0	1.0432	0.99746	0.74522	2.14	3.91
1399	TRUE	Empty	RUN and FYVE domain-containing protein 1 OS=Homo sapiens GN=RUFY1 PE=1 SV=2	RUFY1_HUMAN	?	TRUE	.373900966	INF	0	0	0	0	0	3.91
1400	TRUE	Empty	RuvB-like 1 OS=Homo sapiens GN=RUVBL1 PE=1 SV=1	RUVB1_HUMAN	?	TRUE	.009126578	1.85236109	13.262	14.604	10.972	20.121	24.567	27.254
1401	TRUE	Empty	RuvB-like 2 OS=Homo sapiens GN=RUVBL2 PE=1 SV=3	RUVB2_HUMAN	?		0.018063231	3.414961062	6.631	1.0432	1.49	8.26	11.86	12.217
1402	TRUE	Empty	Saccharopine dehydrogenase-like oxidoreductase OS=Homo sapiens GN=SCCPDH PE=1 SV=1	SCPDL_HUMAN	47 kDa	TRUE	.868541699	1.1	0	1.0432	1.49	0.74522	1.43	0.93978
1403	TRUE	Empty	S-adenosylmethionine synthase isoform type-2 OS=Homo sapiens GN=MAT2A PE=1 SV=1	METK2_HUMAN	?	TRUE	.312675811	1.4	6.631	4.1727	3.98	5.65	10.166	5.87
1404	TRUE	Empty	SAP domain-containing ribonucleoprotein OS=Homo sapiens GN=SARNP PE=1 SV=3	SARNP_HUMAN	24 kDa		0.647890202	0.6	0	2.0863	7.97	0	3.85	2.94
1405	TRUE	Empty	SAP30-binding protein OS=Homo sapiens GN=SAP30BP PE=1 SV=1	S30BP_HUMAN	?	TRUE	.297675591	0.2	0	2.0863	0.99746	0.74522	0	0
1406	TRUE	Empty	Sarcoplasmic/endoplasmic reticulum calcium ATPase 2 OS=Homo sapiens GN=ATP2A2 PE=1 SV=1	AT2A2_HUMAN	?	TRUE	.123070893	5.2	4.06	0	0	8.74	2.14	12.217
1407	TRUE	Empty	Sarcoplasmic/endoplasmic reticulum calcium ATPase 3 OS=Homo sapiens GN=ATP2A3 PE=1 SV=2	AT2A3_HUMAN	?	TRUE	.289763189	2.5	4.06	0	0	4.13	1.43	4.89
1408	TRUE	Empty	Scaffold attachment factor B1 OS=Homo sapiens GN=SAFB PE=1 SV=4	SAFB1_HUMAN	?	TRUE	.516029961	0.6	0	16.691	9.46	3.61	6.71	5.87
1409	TRUE	Empty	Scaffold attachment factor B2 OS=Homo sapiens GN=SAFB2 PE=1 SV=1	SAFB2_HUMAN	?	TRUE	.426409312	0.5	0	8.54	4.73	2.09	0.84713	2.94
1410	TRUE	Empty	SCY1-like protein 2 OS=Homo sapiens GN=SCYL2 PE=1 SV=1	SCYL2_HUMAN	104 kDa	TRUE	.147672906	INF	0	0	0	0	1.43	0.93978
1411	TRUE	Empty	SEC23-interacting protein OS=Homo sapiens GN=SEC23IP PE=1 SV=1	S23IP_HUMAN	?	TRUE	.358410794	9.9	0	0	0.99746	8.26	0	0.93978
1412	TRUE	Empty	Secernin-1 OS=Homo sapiens GN=SCRN1 PE=1 SV=2	SCRN1_HUMAN	?		0.698039406	1.4	0	2.0863	1.49	2.09	2.14	0
1413	TRUE	Empty	Secretory carrier-associated membrane protein 3 OS=Homo sapiens GN=SCAMP3 PE=1 SV=3	SCAM3_HUMAN	?		0.010315957	7.717903475	0	0	0.99746	1.04	3.85	2.94
1414	TRUE	Empty	Selenide, water dikinase 1 OS=Homo sapiens GN=SEPHS1 PE=1 SV=2	SPS1_HUMAN	?	TRUE	.290484135	0.3	0	23.993	14.962	4.13	4.57	4.89
1415	TRUE	Empty	Selenide, water dikinase 2 OS=Homo sapiens GN=SEPHS2 PE=1 SV=3	SPS2_HUMAN	47 kDa	TRUE	.483821997	0.5	0	6.259	2.24	2.57	0.84713	1.96
1416	TRUE	Empty	Selenium-binding protein 1 OS=Homo sapiens GN=SELENBP1 PE=1 SV=2	SBP1_HUMAN	?		0.178972218	0.3	4.06	58.418	56.855	11.178	8.13	13.157
1417	TRUE	Empty	Sentrin-specific protease 3 OS=Homo sapiens GN=SENP3 PE=1 SV=2	SENP3_HUMAN	65 kDa	TRUE	.040274969	4.032853448	0.99746	0	0	2.57	0.84713	0.93978
1418	TRUE	Empty	Sepiapterin reductase OS=Homo sapiens GN=SPR PE=1 SV=1	SPRE_HUMAN	28 kDa		0.127250026	4	0	1.0432	3.98	3.61	5.0828	11.277
1419	TRUE	Empty	Septin-11 OS=Homo sapiens GN=SEPT11 PE=1 SV=3	SEP11_HUMAN	?	TRUE	.805278135	0.8	0	5.59	2.24	2.57	0.84713	3.91
1420	TRUE	Empty	Septin-2 OS=Homo sapiens GN=SEPT2 PE=1 SV=1	SEPT2_HUMAN	?		0.010632285	0.437212309	11.052	13.561	10.972	5.17	6.71	2.94

1421	TRUE	Empty	Septin-7 OS=Homo sapiens GN=SEPT7 PE=1 SV=2	SEPT7_HUMAN	?	TRUE	.776559456	1.2	0	7.22	9.46	9.78	5.99	4.89
1422	TRUE	Empty	Septin-8 OS=Homo sapiens GN=SEPT8 PE=1 SV=4	SEPT8_HUMAN	?	TRUE	.400765353	1.8	0	2.0863	2.24	4.13	0.84713	3.91
1423	TRUE	Empty	Septin-9 OS=Homo sapiens GN=SEPT9 PE=1 SV=2	SEPT9_HUMAN	?	TRUE	.177837452	2.2	0	7.22	4.73	9.78	5.0828	12.217
1424	TRUE	Empty	Sequestosome-1 OS=Homo sapiens GN=SQSTM1 PE=1 SV=1	SQSTM_HUMAN	?	TRUE	.943452794	0.9	0	2.0863	1.49	2.09	0.84713	0
1425	TRUE	Empty	Serine beta-lactamase-like protein LACTB, mitochondrial OS=Homo sapiens GN=LACTB PE=1 SV=2	LACTB_HUMAN	?		0.050597357	INF	0	0	0	2.57	6.71	2.94
1426	TRUE	Empty	Serine dehydratase-like OS=Homo sapiens GN=SDSL PE=1 SV=1	SDSL_HUMAN	35 kDa	TRUE	.211482915	0.2	0	7.22	4.73	0.74522	1.43	0
1427	TRUE	Empty	Serine hydroxymethyltransferase, cytosolic OS=Homo sapiens GN=SHMT1 PE=1 SV=1	GLYC_HUMAN	?	TRUE	.001799291	3.165816141	2.03	3.1295	0.99746	6.707	6.71	6.85
1428	TRUE	Empty	Serine hydroxymethyltransferase, mitochondrial OS=Homo sapiens GN=SHMT2 PE=1 SV=3	GLYM_HUMAN	?	TRUE	.013656834	4.391084199	4.06	2.0863	4.73	12.669	15.248	22.555
1429	TRUE	Empty	Serine palmitoyltransferase 2 OS=Homo sapiens GN=SPTLC2 PE=1 SV=1	SPTC2_HUMAN	63 kDa	TRUE	.117184678	INF	0	0	0	0	1.43	1.96
1430	TRUE	Empty	Serine protease 23 OS=Homo sapiens GN=PRSS23 PE=1 SV=1	PRS23_HUMAN	?	TRUE	0.24820164	0.04	0	4.1727	15.959	0.74522	0	0
1431	TRUE	Empty	Serine/arginine repetitive matrix protein 1 OS=Homo sapiens GN=SRRM1 PE=1 SV=2	SRRM1_HUMAN	?	TRUE	.846250418	0.9	0	5.59	8.71	4.13	3.85	4.89
1432	TRUE	Empty	Serine/arginine repetitive matrix protein 2 OS=Homo sapiens GN=SRRM2 PE=1 SV=2	SRRM2_HUMAN	?	TRUE	.485858436	0.7	2.03	17.734	16.957	8.74	7.42	9.78
1433	TRUE	Empty	Serine/arginine-rich splicing factor 1 OS=Homo sapiens GN=SRSF1 PE=1 SV=2	SRSF1_HUMAN	?	TRUE	.133908648	0.6	41.996	78.238	39.898	24.592	33.038	31.013
1434	TRUE	Empty	Serine/arginine-rich splicing factor 11 OS=Homo sapiens GN=SRSF11 PE=1 SV=1	SRS11_HUMAN	?	TRUE	.555485241	0.6	0	1.0432	1.49	0	0.84713	0.93978
1435	TRUE	Empty	Serine/arginine-rich splicing factor 3 OS=Homo sapiens GN=SRSF3 PE=1 SV=1	SRSF3_HUMAN	?	TRUE	.331126616	0.4	0	28.166	40.896	11.923	10.166	6.85
1436	TRUE	Empty	Serine/arginine-rich splicing factor 6 OS=Homo sapiens GN=SRSF6 PE=1 SV=2	SRSF6_HUMAN	?	TRUE	.684915003	1.3	0	4.1727	8.71	5.17	5.99	4.89
1437	TRUE	Empty	Serine/arginine-rich splicing factor 7 OS=Homo sapiens GN=SRSF7 PE=1 SV=1	SRSF7_HUMAN	?	TRUE	.006240727	0.196308743	22.103	20.863	32.916	5.17	4.57	4.89
1438	TRUE	Empty	Serine/arginine-rich splicing factor 9 OS=Homo sapiens GN=SRSF9 PE=1 SV=1	SRSF9_HUMAN	26 kDa	TRUE	.257173348	0.5	2.03	10.432	8.71	1.04	3.85	5.87
1439	TRUE	Empty	Serine/threonine-protein kinase 24 OS=Homo sapiens GN=STK24 PE=1 SV=1	STK24_HUMAN	?	TRUE	.067655025	4.6	0	0	1.49	2.09	4.57	1.96
1440	TRUE	Empty	Serine/threonine-protein kinase MARK2 OS=Homo sapiens GN=MARK2 PE=1 SV=2	MARK2_HUMAN	?	TRUE	.373900966	INF	0	0	0	2.57	0	0
1441	TRUE	Empty	Serine/threonine-protein kinase MRCK beta OS=Homo sapiens GN=CDC42BPB PE=1 SV=2	MRCKB_HUMAN	194 kDa	TRUE	.373900966	INF	0	0	0	2.57	0	0
1442	TRUE	Empty	Serine/threonine-protein kinase mTOR OS=Homo sapiens GN=MTOR PE=1 SV=1	MTOR_HUMAN	289 kDa	TRUE	.000114504	5.077196078	0	0.99746	0	1.04	1.43	1.96
1443	TRUE	Empty	Serine/threonine-protein kinase OSR1 OS=Homo sapiens GN=OXSR1 PE=1 SV=1	OXSR1_HUMAN	58 kDa	TRUE	.111295643	3.4	2.03	0	0	2.57	3.85	1.96
1444	TRUE	Empty	Serine/threonine-protein kinase PAK 2 OS=Homo sapiens GN=PAK2 PE=1 SV=3	PAK2_HUMAN	58 kDa	TRUE	0.1240126	3.9	2.03	3.1295	0.99746	14.159	6.71	3.91
1445	TRUE	Empty	Serine/threonine-protein kinase PRP4 homolog OS=Homo sapiens GN=PRPF4B PE=1 SV=3	PRP4B_HUMAN	117 kDa	TRUE	0.06853503	INF	0	0	0	0.74522	2.14	0.93978
1446	TRUE	Empty	Serine/threonine-protein phosphatase 1 regulatory subunit 10 OS=Homo sapiens GN=PPP1R10 PE=1 SV=1	PP1RA_HUMAN	99 kDa	TRUE	.373900966	0	0	0	2.24	0	0	0
1447	TRUE	Empty	Serine/threonine-protein phosphatase 2A 55 kDa regulatory subunit B alpha isoform OS=Homo sapiens GN=PPP2R2A PE=1 SV=1	2ABA_HUMAN	?	TRUE	.106985915	5.2	0	0	0.99746	1.04	0.84713	2.94

1448	TRUE	Empty	Serine/threonine-protein phosphatase 2A 56 kDa regulatory subunit delta isoform OS=Homo sapiens GN=PPP2R5D PE=1 SV=1	2A5D_HUMAN	?	TRUE	.116540141	INF	0	0	0	0.74522	1.43	4.89
1449	TRUE	Empty	Serine/threonine-protein phosphatase 2A 65 kDa regulatory subunit A alpha isoform OS=Homo sapiens GN=PPP2R1A PE=1 SV=4	2AAA_HUMAN	65 kDa	TRUE	.068567243	3.3	35.365	3.1295	0.99746	33.535	46.592	49.809
1450	TRUE	Empty	Serine/threonine-protein phosphatase 2A activator OS=Homo sapiens GN=PPP2R4 PE=1 SV=3	PTPA_HUMAN	?		0.092355765	2.8	4.06	2.0863	0	3.61	7.42	6.85
1451	TRUE	Empty	Serine/threonine-protein phosphatase 2A catalytic subunit alpha isoform OS=Homo sapiens GN=PPP2CA PE=1 SV=1	PP2AA_HUMAN	?	TRUE	0.56982463	0.7	2.03	19.82	14.962	8.26	12.707	4.89
1452	TRUE	Empty	Serine/threonine-protein phosphatase 2B catalytic subunit alpha isoform OS=Homo sapiens GN=PPP3CA PE=1 SV=1	PP2BA_HUMAN	?	TRUE	0.29541841	2.8	0	0	2.24	1.04	5.0828	1.96
1453	TRUE	Empty	Serine/threonine-protein phosphatase 5 OS=Homo sapiens GN=PPP5C PE=1 SV=1	PPP5_HUMAN	57 kDa	TRUE	.028710087	2.846922074	0	1.0432	0.99746	2.57	1.43	1.96
1454	TRUE	Empty	Serine/threonine-protein phosphatase 6 catalytic subunit OS=Homo sapiens GN=PPP6C PE=1 SV=1	PPP6_HUMAN	?		0.929780221	1.1	0	2.0863	5.47	2.57	1.43	4.89
1455	TRUE	Empty	Serine/threonine-protein phosphatase 6 regulatory subunit 3 OS=Homo sapiens GN=PPP6R3 PE=1 SV=2	PP6R3_HUMAN	?	TRUE	)000678736	10.90138953	0	0.99746	0	3.61	3.85	3.91
1456	TRUE	Empty	Serine/threonine-protein phosphatase CPPED1 OS=Homo sapiens GN=CPPED1 PE=1 SV=3	CPPED_HUMAN	?		0.954378702	1	0	2.0863	1.49	1.04	0.84713	1.96
1457	TRUE	Empty	Serine/threonine-protein phosphatase PP1-alpha catalytic subunit OS=Homo sapiens GN=PPP1CA PE=1 SV=1	PP1A_HUMAN	?	TRUE	.447182751	0.8	22.103	21.907	28.926	14.159	20.331	27.254
1458	TRUE	Empty	Serine/threonine-protein phosphatase PP1-beta catalytic subunit OS=Homo sapiens GN=PPP1CB PE=1 SV=3	PP1B_HUMAN	37 kDa	TRUE	.704986412	0.9	15.472	14.604	27.929	12.669	15.248	23.495
1459	TRUE	Empty	Serine-threonine kinase receptor-associated protein OS=Homo sapiens GN=STRAP PE=1 SV=1	STRAP_HUMAN	?		0.79694798	1.2	0	4.1727	3.98	1.04	4.57	3.91
1460	TRUE	Empty	Serine--tRNA ligase, cytoplasmic OS=Homo sapiens GN=SARS PE=1 SV=3	SYSC_HUMAN	59 kDa		0.075797828	5.6	2.03	2.0863	1.49	19.376	10.166	5.87
1461	TRUE	Empty	Serine--tRNA ligase, mitochondrial OS=Homo sapiens GN=SARS2 PE=1 SV=1	SYSM_HUMAN	?	TRUE	.373900966	INF	0	0	0	2.57	0	0
1462	TRUE	Empty	Serpin B6 OS=Homo sapiens GN=SERPINB6 PE=1 SV=3	SPB6_HUMAN	43 kDa		0.018109053	5.077196078	0	0	0.99746	1.04	1.43	1.96
1463	TRUE	Empty	Serpin H1 OS=Homo sapiens GN=SERPINH1 PE=1 SV=2	SERPH_HUMAN	46 kDa		0.340897831	2.9	41.996	4.1727	0	93.898	17.79	24.434
1464	TRUE	Empty	Serrate RNA effector molecule homolog OS=Homo sapiens GN=SRRT PE=1 SV=1	SRRT_HUMAN	?	TRUE	.169759736	INF	0	0	0	0	0.84713	1.96
1465	TRUE	Empty	Serum albumin OS=Homo sapiens GN=ALB PE=1 SV=2	ALBU_HUMAN	?		0.156396892	0.3	148.09	37.554	226.42	34.28	19.484	63.905
1466	TRUE	Empty	SET1B_HUMAN-DECOY	SET1B_HUMAN-DEC	?	TRUE	.862024923	0.8	2.03	0	0	0	1.43	0
1467	TRUE	Empty	S-formylglutathione hydrolase OS=Homo sapiens GN=ESD PE=1 SV=2	ESTD_HUMAN	31 kDa		0.365410258	1.8	0	4.1727	5.47	4.13	10.166	3.91
1468	TRUE	Empty	SH3 domain-binding glutamic acid-rich-like protein OS=Homo sapiens GN=SH3BGRL PE=1 SV=1	SH3L1_HUMAN	13 kDa		0.359997146	0.5	0	25.036	19.949	3.61	7.42	9.78
1469	TRUE	Empty	SHC-transforming protein 1 OS=Homo sapiens GN=SHC1 PE=1 SV=4	SHC1_HUMAN	?	TRUE	.198391549	0.1	0	3.1295	1.49	0.74522	0	0
1470	TRUE	Empty	Shootin-1 OS=Homo sapiens GN=SHTN1 PE=1 SV=4	SHOT1_HUMAN	?	TRUE	.675517486	1.7	0	2.0863	0	0.74522	0	2.94
1471	TRUE	Empty	Short/branched chain specific acyl-CoA dehydrogenase, mitochondrial OS=Homo sapiens GN=ACADSB PE=1 SV=1	ACDSB_HUMAN	?		0.036450182	6.580694965	0	0	0.99746	2.57	3.85	0.93978
1472	TRUE	Empty	Sialate O-acetylesterase OS=Homo sapiens GN=SIAE PE=1 SV=1	SIAE_HUMAN	?		0.128620701	0	0	2.0863	2.24	0	0	0
1473	TRUE	Empty	Sialic acid synthase OS=Homo sapiens GN=NANS PE=1 SV=2	SIAS_HUMAN	40 kDa		0.434433138	1.2	8.13	14.604	17.954	20.866	15.248	14.097
1474	TRUE	Empty	Sideroflexin-1 OS=Homo sapiens GN=SFXN1 PE=1 SV=4	SFXN1_HUMAN	36 kDa	TRUE	.003860013	4.173344125	4.06	0	2.24	10.433	10.166	10.338

1475	TRUE	Empty	Signal peptidase complex catalytic subunit SEC11A OS=Homo sapiens GN=SEC11A PE=1 SV=1	SC11A_HUMAN	?	TRUE	.935581317	0.9	6.631	0	0	2.57	0	3.91
1476	TRUE	Empty	Signal recognition particle 14 kDa protein OS=Homo sapiens GN=SRP14 PE=1 SV=2	SRP14_HUMAN	15 kDa		0.482338413	1.4	2.03	7.22	7.97	3.61	11.013	9.78
1477	TRUE	Empty	Signal recognition particle 19 kDa protein OS=Homo sapiens GN=SRP19 PE=1 SV=3	SRP19_HUMAN	?		0.412765041	1.9	0	1.0432	4.73	1.04	4.57	5.87
1478	TRUE	Empty	Signal recognition particle 54 kDa protein OS=Homo sapiens GN=SRP54 PE=1 SV=1	SRP54_HUMAN	?		0.117184738	INF	0	0	0	0	3.85	3.91
1479	TRUE	Empty	Signal recognition particle 9 kDa protein OS=Homo sapiens GN=SRP9 PE=1 SV=2	SRP09_HUMAN	?		0.287079299	0.3	0	17.734	9.46	1.04	3.85	3.91
1480	TRUE	Empty	Signal recognition particle receptor subunit beta OS=Homo sapiens GN=SRPRB PE=1 SV=3	SRPRB_HUMAN	30 kDa		0.447938824	1.9	2.03	0	0	1.04	0.84713	1.96
1481	TRUE	Empty	Signal recognition particle subunit SRP68 OS=Homo sapiens GN=SRP68 PE=1 SV=2	SRP68_HUMAN	?	TRUE	.084566581	INF	0	0	0	2.09	0.84713	0.93978
1482	TRUE	Empty	Signal recognition particle subunit SRP72 OS=Homo sapiens GN=SRP72 PE=1 SV=3	SRP72_HUMAN	?	TRUE	.008125151	20.59651515	0	0	0.99746	5.65	5.99	9.78
1483	TRUE	Empty	Signal transducer and activator of transcription 1-alpha/beta OS=Homo sapiens GN=STAT1 PE=1 SV=2	STAT1_HUMAN	?		0.017884039	5.360126678	6.631	0	0	10.433	11.013	14.097
1484	TRUE	Empty	Signal transducer and activator of transcription 3 OS=Homo sapiens GN=STAT3 PE=1 SV=2	STAT3_HUMAN	?		0.06534674	4.8	2.03	0	0	4.13	4.57	1.96
1485	TRUE	Empty	Single-stranded DNA-binding protein, mitochondrial OS=Homo sapiens GN=SSBP1 PE=1 SV=1	SSBP_HUMAN	17 kDa		0.136394625	2.4	0	3.1295	2.24	6.707	5.0828	2.94
1486	TRUE	Empty	Small glutamine-rich tetratricopeptide repeat-containing protein alpha OS=Homo sapiens GN=SGTA PE=1 SV=1	SGTA_HUMAN	34 kDa		0.334828316	0.4	0	12.518	6.22	2.57	3.85	1.96
1487	TRUE	Empty	Small nuclear ribonucleoprotein E OS=Homo sapiens GN=SNRPE PE=1 SV=1	RUXE_HUMAN	11 kDa		0.41336916	0.5	30.944	1.0432	13.964	3.61	7.42	10.338
1488	TRUE	Empty	Small nuclear ribonucleoprotein F OS=Homo sapiens GN=SNRPF PE=1 SV=1	RUXF_HUMAN	10 kDa		0.277803163	0.6	2.03	6.259	7.97	3.61	3.85	2.94
1489	TRUE	Empty	Small nuclear ribonucleoprotein Sm D1 OS=Homo sapiens GN=SNRPD1 PE=1 SV=1	SMD1_HUMAN	13 kDa		0.262058897	0.6	4.06	7.22	4.73	4.13	5.0828	0
1490	TRUE	Empty	Small nuclear ribonucleoprotein Sm D2 OS=Homo sapiens GN=SNRPD2 PE=1 SV=1	SMD2_HUMAN	?		0.021425574	2.545556805	4.06	1.0432	6.22	11.178	10.166	10.338
1491	TRUE	Empty	Small nuclear ribonucleoprotein Sm D3 OS=Homo sapiens GN=SNRPD3 PE=1 SV=1	SMD3_HUMAN	?	TRUE	.183979726	0.6	26.524	11.475	13.964	7.22	10.166	11.277
1492	TRUE	Empty	Small nuclear ribonucleoprotein-associated protein N OS=Homo sapiens GN=SNRPN PE=1 SV=1	RSMN_HUMAN	?	TRUE	.625608496	0.8	6.631	18.777	11.969	8.26	9.85	13.157
1493	TRUE	Empty	SMC5-SMC6 complex localization factor protein 1 OS=Homo sapiens GN=SLF1 PE=1 SV=2	SLF1_HUMAN	?	TRUE	.373900966	INF	0	0	0	0	1.43	0
1494	TRUE	Empty	SNARE-associated protein Snapin OS=Homo sapiens GN=SNAPIN PE=1 SV=1	SNAPN_HUMAN	15 kDa		0.064564664	2.5	0	1.0432	1.49	2.57	2.14	2.94
1495	TRUE	Empty	Sodium/hydrogen exchanger 2 OS=Homo sapiens GN=SLC9A2 PE=2 SV=1	SL9A2_HUMAN	92 kDa	TRUE	.373900966	0	0	2.0863	0	0	0	0
1496	TRUE	Empty	Sodium/potassium-transporting ATPase subunit alpha-1 OS=Homo sapiens GN=ATP1A1 PE=1 SV=1	AT1A1_HUMAN	?	TRUE	.015973542	12.22558891	4.06	1.0432	1.49	42.477	18.637	30.073
1497	TRUE	Empty	Sodium/potassium-transporting ATPase subunit beta-1 OS=Homo sapiens GN=ATP1B1 PE=1 SV=1	AT1B1_HUMAN	?	TRUE	.006625109	10.24722796	0	0.99746	0	2.09	2.14	4.89
1498	TRUE	Empty	Solute carrier family 12 member 2 OS=Homo sapiens GN=SLC12A2 PE=1 SV=1	S12A2_HUMAN	?	TRUE	.036450182	6.580694965	0	0.99746	0	2.57	3.85	0.93978
1499	TRUE	Empty	Solute carrier family 35 member E1 OS=Homo sapiens GN=SLC35E1 PE=1 SV=2	S35E1_HUMAN	?		0.373900966	INF	0	0	0	0	1.43	0
1500	TRUE	Empty	Sorbitol dehydrogenase OS=Homo sapiens GN=SORD PE=1 SV=4	DHSO_HUMAN	?		0.9752922	1	2.03	2.0863	4.73	2.09	3.85	2.94
1501	TRUE	Empty	Sorcin OS=Homo sapiens GN=SRI PE=1 SV=1	SORCN_HUMAN	?		0.575795448	0.7	4.06	30.252	10.972	5.17	5.99	17.856

1502	TRUE	Empty	Sorting nexin-1 OS=Homo sapiens GN=SNX1 PE=1 SV=3	SNX1_HUMAN	?	TRUE	.118047874	INF	0	0	0	5.65	1.43	0.93978
1503	TRUE	Empty	Sorting nexin-2 OS=Homo sapiens GN=SNX2 PE=1 SV=2	SNX2_HUMAN	?	TRUE	.018397496	14.17781164	0	0	0.99746	5.17	2.14	5.87
1504	TRUE	Empty	Sorting nexin-3 OS=Homo sapiens GN=SNX3 PE=1 SV=3	SNX3_HUMAN	?	TRUE	.866351863	1.2	0	0	1.49	0.74522	1.43	0
1505	TRUE	Empty	Sorting nexin-5 OS=Homo sapiens GN=SNX5 PE=1 SV=1	SNX5_HUMAN	?	TRUE	.092878935	5.5	0	1.0432	0	2.09	0.84713	1.96
1506	TRUE	Empty	Sorting nexin-6 OS=Homo sapiens GN=SNX6 PE=1 SV=1	SNX6_HUMAN	?	TRUE	.155248768	INF	0	0	0	4.13	0.84713	0.93978
1507	TRUE	Empty	Spectrin alpha chain, erythrocytic 1 OS=Homo sapiens GN=SPTA1 PE=1 SV=5	SPTA1_HUMAN	?	TRUE	.373900966	0	0	0	6.22	0	0	0
1508	TRUE	Empty	Spectrin alpha chain, non-erythrocytic 1 OS=Homo sapiens GN=SPTAN1 PE=1 SV=3	SPTN1_HUMAN	?	TRUE	.471977501	0.7	11.052	64.677	72.814	32.79	33.885	35.712
1509	TRUE	Empty	Spectrin beta chain, erythrocytic OS=Homo sapiens GN=SPTB PE=1 SV=5	SPTB1_HUMAN	?	TRUE	.587433298	0.4	0	0	3.98	0.74522	0.84713	0
1510	TRUE	Empty	Spectrin beta chain, non-erythrocytic 1 OS=Homo sapiens GN=SPTBN1 PE=1 SV=2	SPTB2_HUMAN	?	TRUE	.066532934	3.1	0	3.1295	7.97	14.904	7.42	12.217
1511	TRUE	Empty	Spectrin beta chain, non-erythrocytic 2 OS=Homo sapiens GN=SPTBN2 PE=1 SV=3	SPTN2_HUMAN	?	TRUE	.001524133	7.420748702	0	0.99746	0	2.09	2.14	1.96
1512	TRUE	Empty	Spermatid perinuclear RNA-binding protein OS=Homo sapiens GN=STRBP PE=1 SV=1	STRBP_HUMAN	?	TRUE	.384338753	1.4	2.03	3.1295	1.49	3.61	1.43	4.89
1513	TRUE	Empty	Spermatogenesis-defective protein 39 homolog OS=Homo sapiens GN=VIPAS39 PE=1 SV=1	SPE39_HUMAN	?		0.373900966	INF	0	0	0	2.09	0	0
1514	TRUE	Empty	Spermidine synthase OS=Homo sapiens GN=SRM PE=1 SV=1	SPEE_HUMAN	34 kDa		0.022694969	6.069357101	2.03	0	0	3.61	5.99	3.91
1515	TRUE	Empty	S-phase kinase-associated protein 1 OS=Homo sapiens GN=SKP1 PE=1 SV=2	SKP1_HUMAN	?	TRUE	.215983346	2.7	0	3.1295	0	2.09	1.43	3.91
1516	TRUE	Empty	Sphingosine-1-phosphate lyase 1 OS=Homo sapiens GN=SGPL1 PE=1 SV=3	SGPL1_HUMAN	64 kDa		0.822789008	1.2	6.631	0	0	2.09	3.85	1.96
1517	TRUE	Empty	Spliceosome RNA helicase DDX39B OS=Homo sapiens GN=DDX39B PE=1 SV=1	DX39B_HUMAN	?	TRUE	.018571105	1.771415362	19.893	13.561	11.969	24.592	30.497	25.374
1518	TRUE	Empty	Splicing factor 1 OS=Homo sapiens GN=SF1 PE=1 SV=4	SF01_HUMAN	?	TRUE	.844297138	0.9	4.06	13.561	11.969	7.22	9.85	11.277
1519	TRUE	Empty	Splicing factor 3A subunit 1 OS=Homo sapiens GN=SF3A1 PE=1 SV=1	SF3A1_HUMAN	?	TRUE	.732683096	1.1	2.03	11.475	10.972	10.433	10.166	7.83
1520	TRUE	Empty	Splicing factor 3A subunit 3 OS=Homo sapiens GN=SF3A3 PE=1 SV=1	SF3A3_HUMAN	59 kDa		0.01159133	5.148480958	0	1.0432	1.49	6.707	4.57	4.89
1521	TRUE	Empty	Splicing factor 3B subunit 1 OS=Homo sapiens GN=SF3B1 PE=1 SV=3	SF3B1_HUMAN	?	TRUE	.012864893	8.909612939	2.03	0	0.99746	10.433	5.99	12.217
1522	TRUE	Empty	Splicing factor 3B subunit 2 OS=Homo sapiens GN=SF3B2 PE=1 SV=2	SF3B2_HUMAN	100 kDa		0.211640432	0.8	11.052	15.648	15.959	7.22	12.707	12.217
1523	TRUE	Empty	Splicing factor 3B subunit 3 OS=Homo sapiens GN=SF3B3 PE=1 SV=4	SF3B3_HUMAN	?	TRUE	.285563888	1.7	26.524	6.259	8.71	15.65	23.72	31.013
1524	TRUE	Empty	Splicing factor 3B subunit 4 OS=Homo sapiens GN=SF3B4 PE=1 SV=1	SF3B4_HUMAN	44 kDa		0.239842021	0.6	6.631	9.86	10.972	2.09	10.166	1.96
1525	TRUE	Empty	Splicing factor 3B subunit 5 OS=Homo sapiens GN=SF3B5 PE=1 SV=1	SF3B5_HUMAN	10 kDa		0.974219573	1	0	3.1295	0	2.09	0	0
1526	TRUE	Empty	Splicing factor 3B subunit 6 OS=Homo sapiens GN=SF3B6 PE=1 SV=1	SF3B6_HUMAN	15 kDa		0.503679695	2.2	0	0	1.49	1.04	0	2.94
1527	TRUE	Empty	Splicing factor U2AF 35 kDa subunit OS=Homo sapiens GN=U2AF1 PE=1 SV=3	U2AF1_HUMAN	?	TRUE	.227372921	0.3	0	5.59	4.73	0	1.43	0.93978
1528	TRUE	Empty	Splicing factor U2AF 65 kDa subunit OS=Homo sapiens GN=U2AF2 PE=1 SV=4	U2AF2_HUMAN	?		0.444435751	0.7	6.631	21.907	22.942	11.178	10.166	15.976
1529	TRUE	Empty	Splicing factor, proline- and glutamine-rich OS=Homo sapiens GN=SFPQ PE=1 SV=2	SFPQ_HUMAN	?	TRUE	.112783319	0.5	11.052	27.122	30.921	9.78	11.86	10.338
1530	TRUE	Empty	Squamous cell carcinoma antigen recognized by T-cells 3 OS=Homo sapiens GN=SART3 PE=1 SV=1	SART3_HUMAN	?	TRUE	.074940238	4.2	0	0	0.99746	1.04	0.84713	1.96

1531	TRUE	Empty	SRA stem-loop-interacting RNA-binding protein, mitochondrial OS=Homo sapiens GN=SLIRP PE=1 SV=1	SLIRP_HUMAN	?	TRUE	.056361213	1.8	2.03	1.0432	1.49	2.09	2.14	3.91
1532	TRUE	Empty	Src substrate cortactin OS=Homo sapiens GN=CTTN PE=1 SV=2	SRC8_HUMAN	?	TRUE	.887759393	1.1	6.631	28.166	43.888	52.911	15.248	17.856
1533	TRUE	Empty	Staphylococcal nuclease domain-containing protein 1 OS=Homo sapiens GN=SND1 PE=1 SV=1	SND1_HUMAN	102 kDa		0.004245828	17.81681266	2.03	1.0432	0	15.65	16.943	25.374
1534	TRUE	Empty	Stathmin OS=Homo sapiens GN=STMN1 PE=1 SV=3	STMN1_HUMAN	?	TRUE	0.17222739	0.4	4.06	23.993	19.949	4.13	3.85	9.78
1535	TRUE	Empty	STE20/SPS1-related proline-alanine-rich protein kinase OS=Homo sapiens GN=STK39 PE=1 SV=3	STK39_HUMAN	?	TRUE	.010897438	12.04800192	0.99746	0	0	2.57	5.0828	4.89
1536	TRUE	Empty	Stomatin-like protein 2, mitochondrial OS=Homo sapiens GN=STOML2 PE=1 SV=1	STML2_HUMAN	?		0.014996461	12.14093798	0	0.99746	0	2.57	4.57	5.87
1537	TRUE	Empty	Stress-70 protein, mitochondrial OS=Homo sapiens GN=HSPA9 PE=1 SV=2	GRP75_HUMAN	74 kDa	TRUE	.747671868	0.9	110.52	87.626	50.87	66.324	82.172	81.761
1538	TRUE	Empty	Stress-induced-phosphoprotein 1 OS=Homo sapiens GN=STIP1 PE=1 SV=1	STIP1_HUMAN	?		0.102834031	0.7	37.575	54.245	48.875	35.025	39.815	30.073
1539	TRUE	Empty	Striatin OS=Homo sapiens GN=STRN PE=1 SV=4	STRN_HUMAN	?	TRUE	.318527546	0.3	0	1.0432	1.49	0	0.84713	0
1540	TRUE	Empty	Striatin-4 OS=Homo sapiens GN=STRN4 PE=1 SV=2	STRN4_HUMAN	?	TRUE	.544033882	0.4	0	1.0432	3.98	2.57	0	0
1541	TRUE	Empty	Structural maintenance of chromosomes protein 1A OS=Homo sapiens GN=SMC1A PE=1 SV=2	SMC1A_HUMAN	143 kDa		0.610797588	1.9	0	0	1.49	2.09	0.84713	0
1542	TRUE	Empty	Succinate dehydrogenase [ubiquinone] flavoprotein subunit, mitochondrial OS=Homo sapiens GN=SDHA PE=1 SV=2	SDHA_HUMAN	?		0.21589594	1.9	8.13	2.0863	0.99746	8.26	7.42	6.85
1543	TRUE	Empty	Succinate-semialdehyde dehydrogenase, mitochondrial OS=Homo sapiens GN=ALDH5A1 PE=1 SV=2	SSDH_HUMAN	?		0.035403751	7.37068635	0	1.0432	0	2.57	1.43	3.91
1544	TRUE	Empty	Succinyl-CoA ligase [ADP/GDP-forming] subunit alpha, mitochondrial OS=Homo sapiens GN=SUCLG1 PE=1 SV=4	SUCA_HUMAN	36 kDa		0.295651198	1.8	0	1.0432	2.24	2.09	2.14	1.96
1545	TRUE	Empty	Succinyl-CoA ligase [GDP-forming] subunit beta, mitochondrial OS=Homo sapiens GN=SUCLG2 PE=1 SV=2	SUCB2_HUMAN	?		0.184058619	4	2.03	0	0	0.74522	3.85	4.89
1546	TRUE	Empty	Sulfatase-modifying factor 2 OS=Homo sapiens GN=SUMF2 PE=1 SV=2	SUMF2_HUMAN	?	TRUE	.373900966	INF	0	0	0	1.04	0	0
1547	TRUE	Empty	SUMO-activating enzyme subunit 1 OS=Homo sapiens GN=SAE1 PE=1 SV=1	SAE1_HUMAN	?	TRUE	.061814651	4.9	0	0	0.99746	2.57	1.43	0.93978
1548	TRUE	Empty	SUMO-activating enzyme subunit 2 OS=Homo sapiens GN=UBA2 PE=1 SV=2	SAE2_HUMAN	?	TRUE	.047982325	2.596305764	2.03	0	2.24	3.61	5.0828	4.89
1549	TRUE	Empty	SUMO-conjugating enzyme UBC9 OS=Homo sapiens GN=UBE2I PE=1 SV=1	UBC9_HUMAN	18 kDa		0.802212827	1.1	2.03	6.259	3.98	4.13	4.57	4.89
1550	TRUE	Empty	Superkiller viralicidic activity 2-like 2 OS=Homo sapiens GN=SKIV2L2 PE=1 SV=3	SK2L2_HUMAN	118 kDa	TRUE	.040274969	4.032853448	0	0.99746	0	2.57	0.84713	0.93978
1551	TRUE	Empty	Superoxide dismutase [Cu-Zn] OS=Homo sapiens GN=SOD1 PE=1 SV=2	SODC_HUMAN	16 kDa		0.06598773	0.4	13.262	18.777	8.71	8.74	3.85	5.87
1552	TRUE	Empty	Surfeit locus protein 4 OS=Homo sapiens GN=SURF4 PE=1 SV=3	SURF4_HUMAN	?	TRUE	.114689101	4.2	8.13	0	0	8.74	9.85	19.735
1553	TRUE	Empty	Survival motor neuron protein OS=Homo sapiens GN=SMN1 PE=1 SV=1	SMN_HUMAN	?		0.44897503	0.4	0	6.259	1.49	2.09	0	0
1554	TRUE	Empty	SWI/SNF complex subunit SMARCC2 OS=Homo sapiens GN=SMARCC2 PE=1 SV=1	SMRC2_HUMAN	?	TRUE	0.37532752	3.2	0	0	0.99746	2.57	0	0.93978
1555	TRUE	Empty	SWI/SNF-related matrix-associated actin-dependent regulator of chromatin subfamily A member 5 OS=Homo sapiens GN=SMARCA5 PE=1 SV=1	SMCA5_HUMAN	122 kDa	TRUE	.077979801	INF	0	0	0	8.74	3.85	1.96
1556	TRUE	Empty	Symplekin OS=Homo sapiens GN=SYMPK PE=1 SV=2	SYMPK_HUMAN	?	TRUE	.189953018	INF	0	0	0	2.57	0.84713	0
1557	TRUE	Empty	Synapse-associated protein 1 OS=Homo sapiens GN=SYAP1 PE=1 SV=1	SYAP1_HUMAN	40 kDa		0.369663378	1.7	0	3.1295	4.73	2.57	5.99	5.87

1558	TRUE	Empty	Synaptic vesicle membrane protein VAT-1 homolog OS=Homo sapiens GN=VAT1 PE=1 SV=2	VAT1_HUMAN	?		0.03651796	3.680294278	0	3.1295	1.49	4.13	5.99	8.81
1559	TRUE	Empty	Synaptosomal-associated protein 29 OS=Homo sapiens GN=SNAP29 PE=1 SV=1	SNP29_HUMAN	29 kDa		0.948205042	1	0	6.259	3.98	3.61	4.57	1.96
1560	TRUE	Empty	Syntaxin-12 OS=Homo sapiens GN=STX12 PE=1 SV=1	STX12_HUMAN	32 kDa		0.373900966	INF	0	0	0	1.04	0	0
1561	TRUE	Empty	Syntaxin-16 OS=Homo sapiens GN=STX16 PE=1 SV=3	STX16_HUMAN	?		0.043064785	5.272311672	0.99746	0	0	0.74522	1.43	2.94
1562	TRUE	Empty	Syntaxin-17 OS=Homo sapiens GN=STX17 PE=1 SV=2	STX17_HUMAN	33 kDa	TRUE	.028213971	5.91725984	0.99746	0	0	2.57	0.84713	2.94
1563	TRUE	Empty	Syntaxin-binding protein 3 OS=Homo sapiens GN=STXBP3 PE=1 SV=2	STXB3_HUMAN	68 kDa		0.0398156	11.35133238	0	0	0.99746	5.17	2.14	2.94
1564	TRUE	Empty	Syntenin-1 OS=Homo sapiens GN=SDCBP PE=1 SV=1	SDCB1_HUMAN	?		0.068305638	4	0	1.0432	0	1.04	1.43	0.93978
1565	TRUE	Empty	Talin-1 OS=Homo sapiens GN=TLN1 PE=1 SV=3	TLN1_HUMAN	270 kDa	TRUE	.050972045	3	4.06	3.1295	15.959	20.866	18.637	31.953
1566	TRUE	Empty	Taperin OS=Homo sapiens GN=TPRN PE=1 SV=2	TPRN_HUMAN	?	TRUE	0.15359	0	0	1.0432	1.49	0	0	0
1567	TRUE	Empty	TAR DNA-binding protein 43 OS=Homo sapiens GN=TARDBP PE=1 SV=1	TADBP_HUMAN	?		0.096356337	1.7	6.631	7.22	2.24	8.74	11.86	8.81
1568	TRUE	Empty	Tax1-binding protein 3 OS=Homo sapiens GN=TAX1BP3 PE=1 SV=2	TX1B3_HUMAN	14 kDa		0.319927212	0.4	0	4.1727	3.98	1.04	1.43	0
1569	TRUE	Empty	T-complex protein 1 subunit alpha OS=Homo sapiens GN=TCP1 PE=1 SV=1	TCPA_HUMAN	60 kDa	TRUE	.017626406	2.307611931	6.631	4.1727	4.73	14.904	9.85	12.217
1570	TRUE	Empty	T-complex protein 1 subunit beta OS=Homo sapiens GN=CCT2 PE=1 SV=4	TCPB_HUMAN	?	TRUE	0.13827394	1.6	48.627	16.691	23.939	47.694	45.745	49.809
1571	TRUE	Empty	T-complex protein 1 subunit delta OS=Homo sapiens GN=CCT4 PE=1 SV=4	TCPD_HUMAN	?		0.073112599	2.1	19.893	8.54	7.97	27.573	17.79	29.133
1572	TRUE	Empty	T-complex protein 1 subunit epsilon OS=Homo sapiens GN=CCT5 PE=1 SV=1	TCPE_HUMAN	?		0.489256994	1.2	30.944	14.604	20.947	23.102	24.567	31.013
1573	TRUE	Empty	T-complex protein 1 subunit eta OS=Homo sapiens GN=CCT7 PE=1 SV=2	TCPH_HUMAN	?		0.068476522	2.7	8.13	0	7.97	11.178	14.401	20.675
1574	TRUE	Empty	T-complex protein 1 subunit gamma OS=Homo sapiens GN=CCT3 PE=1 SV=4	TCPG_HUMAN	?	TRUE	.918010276	0.9	64.099	10.432	7.97	28.318	23.72	24.434
1575	TRUE	Empty	T-complex protein 1 subunit theta OS=Homo sapiens GN=CCT8 PE=1 SV=4	TCPQ_HUMAN	?		0.059221701	2.3	17.683	10.432	6.22	20.121	23.72	35.712
1576	TRUE	Empty	T-complex protein 1 subunit zeta OS=Homo sapiens GN=CCT6A PE=1 SV=3	TCPZ_HUMAN	?	TRUE	.347861381	1.5	17.683	10.432	16.957	33.535	22.025	11.277
1577	TRUE	Empty	Telomeric repeat-binding factor 2-interacting protein 1 OS=Homo sapiens GN=TERF2IP PE=1 SV=1	TE2IP_HUMAN	44 kDa		0.181120449	0.2	0	3.1295	2.24	0	0	0.93978
1578	TRUE	Empty	Testin OS=Homo sapiens GN=TES PE=1 SV=1	TES_HUMAN	?		0.518941455	1.4	0	9.86	4.73	5.65	7.42	7.83
1579	TRUE	Empty	Testis-expressed sequence 10 protein OS=Homo sapiens GN=TEX10 PE=1 SV=2	TEX10_HUMAN	?	TRUE	0.06853503	INF	0	0	0	0.74522	2.14	0.93978
1580	TRUE	Empty	Tether containing UBX domain for GLUT4 OS=Homo sapiens GN=ASPSCR1 PE=1 SV=1	ASPC1_HUMAN	?		0.668228187	0.6	0	2.0863	0.99746	0	0	1.96
1581	TRUE	Empty	Tetratricopeptide repeat protein 1 OS=Homo sapiens GN=TTC1 PE=1 SV=1	TTC1_HUMAN	34 kDa		0.377741859	0.4	0	8.54	4.73	0	4.57	0.93978
1582	TRUE	Empty	Tetratricopeptide repeat protein 37 OS=Homo sapiens GN=TTC37 PE=1 SV=1	TTC37_HUMAN	175 kDa	TRUE	.184606477	INF	0	0	0	0.74522	0	1.96
1583	TRUE	Empty	Thioredoxin domain-containing protein 12 OS=Homo sapiens GN=TXNDC12 PE=1 SV=1	TXD12_HUMAN	19 kDa		0.388505473	3.5	0	1.0432	0	0	0.84713	2.94
1584	TRUE	Empty	Thioredoxin domain-containing protein 17 OS=Homo sapiens GN=TXNDC17 PE=1 SV=1	TXD17_HUMAN	14 kDa		0.605837867	1.4	0	7.22	10.972	3.61	8.13	13.157
1585	TRUE	Empty	Thioredoxin domain-containing protein 5 OS=Homo sapiens GN=TXNDC5 PE=1 SV=2	TXND5_HUMAN	?		0.075412414	2.4	0	4.1727	8.71	10.433	11.013	10.338
1586	TRUE	Empty	Thioredoxin OS=Homo sapiens GN=TXN PE=1 SV=3	THIO_HUMAN	?		0.960421376	1	11.052	86.583	57.853	45.458	68.618	45.11
1587	TRUE	Empty	Thioredoxin reductase 1, cytoplasmic OS=Homo sapiens GN=TXNRD1 PE=1 SV=3	TRXR1_HUMAN	?	TRUE	.828342834	1.1	4.06	22.95	5.47	12.669	13.554	11.277

1588	TRUE	Empty	Thioredoxin-dependent peroxide reductase, mitochondrial OS=Homo sapiens GN=PRDX3 PE=1 SV=3	PRDX3_HUMAN	?	TRUE	.026384816	1.77125276	4.06	9.86	5.47	11.923	11.86	11.277
1589	TRUE	Empty	Thioredoxin-like protein 1 OS=Homo sapiens GN=TXNL1 PE=1 SV=3	TXNL1_HUMAN	32 kDa		0.950197635	1.1	2.03	2.0863	0	0	1.43	2.94
1590	TRUE	Empty	Thioredoxin-related transmembrane protein 1 OS=Homo sapiens GN=TMX1 PE=1 SV=1	TMX1_HUMAN	32 kDa		0.1959113	INF	0	0	0	10.433	0	3.91
1591	TRUE	Empty	Thiosulfate sulfurtransferase/rhodanese-like domain-containing protein 1 OS=Homo sapiens GN=TSTD1 PE=1 SV=3	TSTD1_HUMAN	?		0.373900966	0	0	2.0863	0	0	0	0
1592	TRUE	Empty	THO complex subunit 4 OS=Homo sapiens GN=ALYREF PE=1 SV=3	THOC4_HUMAN	27 kDa	TRUE	.433898909	1.5	2.03	1.0432	1.49	0.74522	3.85	3.91
1593	TRUE	Empty	Threonine--tRNA ligase, cytoplasmic OS=Homo sapiens GN=TARS PE=1 SV=3	SYTC_HUMAN	?	TRUE	.180029522	2.3	0	5.59	9.46	5.17	13.554	15.976
1594	TRUE	Empty	Thrombomodulin OS=Homo sapiens GN=THBD PE=1 SV=2	TRBM_HUMAN	60 kDa		0.373900966	INF	0	0	0	1.04	0	0
1595	TRUE	Empty	Thrombospondin-1 OS=Homo sapiens GN=THBS1 PE=1 SV=2	TSP1_HUMAN	?	TRUE	.373900966	0	0	0	2.24	0	0	0
1596	TRUE	Empty	THUMP domain-containing protein 1 OS=Homo sapiens GN=THUMPD1 PE=1 SV=2	THUM1_HUMAN	39 kDa		0.803629916	0.9	0	2.0863	2.24	0.74522	1.43	1.96
1597	TRUE	Empty	Thymidine kinase, cytosolic OS=Homo sapiens GN=TK1 PE=1 SV=2	KITH_HUMAN	25 kDa		0.295249008	6.6	0	0	0.99746	1.04	5.0828	0
1598	TRUE	Empty	Thymidylate kinase OS=Homo sapiens GN=DTYMK PE=1 SV=4	KTHY_HUMAN	?		0.228112223	2.1	0	3.1295	0.99746	2.57	2.14	3.91
1599	TRUE	Empty	Thymosin beta-4 OS=Homo sapiens GN=TMSB4X PE=1 SV=2	TYB4_HUMAN	5 kDa		0.321611456	0.1	8.13	5.59	80.794	8.74	0	2.94
1600	TRUE	Empty	Thyroid hormone receptor-associated protein 3 OS=Homo sapiens GN=THRAP3 PE=1 SV=2	TR150_HUMAN	109 kDa	TRUE	.839829928	0.9	0	5.59	4.73	4.13	1.43	2.94
1601	TRUE	Empty	Thyroid receptor-interacting protein 11 OS=Homo sapiens GN=TRIP11 PE=1 SV=3	TRIPB_HUMAN	228 kDa	TRUE	.204964682	4.4	0	0	0.99746	0.74522	0.84713	2.94
1602	TRUE	Empty	Thyroid receptor-interacting protein 6 OS=Homo sapiens GN=TRIP6 PE=1 SV=3	TRIP6_HUMAN	?		0.924061527	0.9	0	10.432	4.73	8.26	2.14	2.94
1603	TRUE	Empty	Thyroxine-binding globulin OS=Homo sapiens GN=SERPINA7 PE=1 SV=2	THBG_HUMAN	46 kDa		0.373900966	0	0	0	2.24	0	0	0
1604	TRUE	Empty	Tight junction protein ZO-1 OS=Homo sapiens GN=TJP1 PE=1 SV=3	ZO1_HUMAN	?	TRUE	.145035574	0.3	2.03	11.475	12.967	2.09	3.85	1.96
1605	TRUE	Empty	Tight junction protein ZO-2 OS=Homo sapiens GN=TJP2 PE=1 SV=2	ZO2_HUMAN	?	TRUE	.446758635	2	0	0	1.49	2.57	0.84713	0.93978
1606	TRUE	Empty	Tissue alpha-L-fucosidase OS=Homo sapiens GN=FUCA1 PE=1 SV=4	FUCO_HUMAN	54 kDa	TRUE	.289681027	3.4	0	1.0432	0	0	1.43	1.96
1607	TRUE	Empty	Titin OS=Homo sapiens GN=TTN PE=1 SV=4	TITIN_HUMAN	?	TRUE	.660953421	0.5	0	3.1295	0	1.04	0	0
1608	TRUE	Empty	TITIN_HUMAN-DECOY	TITIN_HUMAN-DEC(	?	TRUE	.003651919	4.134982856	0	0	0.99746	1.04	1.43	0.93978
1609	TRUE	Empty	Torsin-1A-interacting protein 1 OS=Homo sapiens GN=TOR1AIP1 PE=1 SV=2	TOIP1_HUMAN	?	TRUE	.141140807	INF	0	0	0	2.57	0	3.91
1610	TRUE	Empty	Trafficking protein particle complex subunit 3 OS=Homo sapiens GN=TRAPPC3 PE=1 SV=1	TPPC3_HUMAN	?		0.373900966	INF	0	0	0	0	2.14	0
1611	TRUE	Empty	TRAF-type zinc finger domain-containing protein 1 OS=Homo sapiens GN=TRAFD1 PE=1 SV=1	TRAD1_HUMAN	?		0.373900966	INF	0	0	0	2.57	0	0
1612	TRUE	Empty	Transaldolase OS=Homo sapiens GN=TALDO1 PE=1 SV=2	TALDO_HUMAN	38 kDa	TRUE	.038552668	2.471274364	2.03	5.59	10.972	14.904	12.707	17.856
1613	TRUE	Empty	Transcription elongation factor A protein 1 OS=Homo sapiens GN=TCEA1 PE=1 SV=2	TCEA1_HUMAN	?	TRUE	.743633189	0.7	0	1.0432	8.71	1.04	1.43	3.91
1614	TRUE	Empty	Transcription elongation factor A protein-like 4 OS=Homo sapiens GN=TCEAL4 PE=1 SV=2	TCAL4_HUMAN	?	TRUE	.660925434	0.7	0	3.1295	1.49	0	1.43	1.96
1615	TRUE	Empty	Transcription elongation factor SPT5 OS=Homo sapiens GN=SUPT5H PE=1 SV=1	SPT5H_HUMAN	?	TRUE	.005538615	3.285655565	0	0	0.99746	1.04	0.84713	0.93978

1616	TRUE	Empty	Transcription elongation factor SPT6 OS=Homo sapiens GN=SUPT6H PE=1 SV=2	SPT6H_HUMAN	?	TRUE	.145322396	INF	0	0	0	1.04	0.84713	0
1617	TRUE	Empty	Transcription elongation regulator 1 OS=Homo sapiens GN=TCERG1 PE=1 SV=2	TCRG1_HUMAN	?	TRUE	0.91323029	0.9	0	3.1295	2.24	3.61	0	1.96
1618	TRUE	Empty	Transcription factor 25 OS=Homo sapiens GN=TCF25 PE=1 SV=1	TCF25_HUMAN	77 kDa	TRUE	.023730014	6.571491589	0	0	0.99746	2.09	1.43	1.96
1619	TRUE	Empty	Transcription factor BTF3 homolog 4 OS=Homo sapiens GN=BTF3L4 PE=1 SV=1	BT3L4_HUMAN	?	TRUE	.373900966	INF	0	0	0	1.04	0	0
1620	TRUE	Empty	Transcription factor BTF3 OS=Homo sapiens GN=BTF3 PE=1 SV=1	BTF3_HUMAN	?	TRUE	0.71542278	0.8	0	8.54	5.47	4.13	0.84713	5.87
1621	TRUE	Empty	Transcription factor jun-B OS=Homo sapiens GN=JUNB PE=1 SV=1	JUNB_HUMAN	36 kDa		0.163162035	0	0	2.0863	0.99746	0	0	0
1622	TRUE	Empty	Transcription intermediary factor 1-beta OS=Homo sapiens GN=TRIM28 PE=1 SV=5	TIF1B_HUMAN	?		0.05275731	2.2	28.734	10.432	7.97	33.535	31.344	38.531
1623	TRUE	Empty	Transcriptional activator protein Pur-alpha OS=Homo sapiens GN=PURA PE=1 SV=2	PURA_HUMAN	35 kDa	TRUE	0.41446157	0.5	2.03	1.0432	5.47	1.04	2.14	0.93978
1624	TRUE	Empty	Transcriptional repressor p66-alpha OS=Homo sapiens GN=GATAD2A PE=1 SV=1	P66A_HUMAN	?	TRUE	.397222029	0.2	0	1.0432	4.73	1.04	0	0
1625	TRUE	Empty	Transducin beta-like protein 2 OS=Homo sapiens GN=TBL2 PE=1 SV=1	TBL2_HUMAN	50 kDa	TRUE	.005538615	3.285655565	0.99746	0	0	1.04	0.84713	0.93978
1626	TRUE	Empty	Transferrin receptor protein 1 OS=Homo sapiens GN=TFRC PE=1 SV=2	TFR1_HUMAN	85 kDa		0.001271201	13.99396773	6.631	0	0	33.535	33.885	25.374
1627	TRUE	Empty	Transformer-2 protein homolog alpha OS=Homo sapiens GN=TRA2A PE=1 SV=1	TRA2A_HUMAN	?	TRUE	.485393711	0.7	2.03	5.59	1.49	1.04	2.14	2.94
1628	TRUE	Empty	Transformer-2 protein homolog beta OS=Homo sapiens GN=TRA2B PE=1 SV=1	TRA2B_HUMAN	?	TRUE	.010181353	0,0	2.03	2.0863	0.99746	0	0.84713	0
1629	TRUE	Empty	Transforming protein RhoA OS=Homo sapiens GN=RHOA PE=1 SV=1	RHOA_HUMAN	22 kDa	TRUE	.021213739	18.89800092	0	0	0.99746	5.65	4.57	9.78
1630	TRUE	Empty	Transgelin-2 OS=Homo sapiens GN=TAGLN2 PE=1 SV=3	TAGL2_HUMAN	?	TRUE	.805582053	1.1	19.893	76.152	66.83	44.713	55.911	78.002
1631	TRUE	Empty	Transitional endoplasmic reticulum ATPase OS=Homo sapiens GN=VCP PE=1 SV=4	TERA_HUMAN	89 kDa	TRUE	.260783834	0.6	53.048	156.48	113.71	59.617	83.866	57.327
1632	TRUE	Empty	Transketolase OS=Homo sapiens GN=TKT PE=1 SV=3	TKT_HUMAN	?	TRUE	.182938374	0.5	72.941	187.77	155.6	111.78	69.465	45.11
1633	TRUE	Empty	Translation initiation factor eIF-2B subunit alpha OS=Homo sapiens GN=EIF2B1 PE=1 SV=1	EI2BA_HUMAN	?	TRUE	.009521936	4.227868787	0	0.99746	0	1.04	0.84713	1.96
1634	TRUE	Empty	Translation initiation factor eIF-2B subunit epsilon OS=Homo sapiens GN=EIF2B5 PE=1 SV=3	EI2BE_HUMAN	80 kDa	TRUE	.000114504	5.077196078	0.99746	0	0	1.04	1.43	1.96
1635	TRUE	Empty	Translational activator GCN1 OS=Homo sapiens GN=GCN1L1 PE=1 SV=6	GCN1L_HUMAN	293 kDa	TRUE	.007958061	9.413228375	11.052	0	0	29.064	44.898	30.073
1636	TRUE	Empty	Translational activator of cytochrome c oxidase 1 OS=Homo sapiens GN=TACO1 PE=1 SV=1	TACO1_HUMAN	32 kDa		0.06335845	INF	0	0	0	5.65	0.84713	3.91
1637	TRUE	Empty	Translationally-controlled tumor protein OS=Homo sapiens GN=TPT1 PE=1 SV=1	TCTP_HUMAN	?	TRUE	.962596211	1	8.13	8.54	11.969	5.17	5.0828	18.796
1638	TRUE	Empty	Translin OS=Homo sapiens GN=TSN PE=1 SV=1	TSN_HUMAN	?		0.241857663	5.7	0	1.0432	0	0.74522	4.57	0.93978
1639	TRUE	Empty	Translin-associated protein X OS=Homo sapiens GN=TSNAX PE=1 SV=1	TSNAX_HUMAN	33 kDa		0.270866	2.3	2.03	0	0	1.04	1.43	1.96
1640	TRUE	Empty	Translocon-associated protein subunit delta OS=Homo sapiens GN=SSR4 PE=1 SV=1	SSRD_HUMAN	19 kDa		0.016503714	4.975066669	0	0.99746	0	2.57	0.84713	1.96
1641	TRUE	Empty	Transmembrane emp24 domain-containing protein 10 OS=Homo sapiens GN=TMED10 PE=1 SV=2	TMEDA_HUMAN	25 kDa	TRUE	0.0263936	8.865402886	2.03	0	0	5.17	4.57	9.78
1642	TRUE	Empty	Transmembrane emp24 domain-containing protein 2 OS=Homo sapiens GN=TMED2 PE=1 SV=1	TMED2_HUMAN	23 kDa		0.003020465	5.926453191	0	0	0.99746	1.04	2.14	1.96
1643	TRUE	Empty	Transmembrane emp24 domain-containing protein 7 OS=Homo sapiens GN=TMED7 PE=1 SV=2	TMED7_HUMAN	?	TRUE	.291574741	2.6	2.03	0	0	2.09	0.84713	1.96

1644	TRUE	Empty	Transmembrane emp24 domain-containing protein 9 OS=Homo sapiens GN=TMED9 PE=1 SV=2	TMED9_HUMAN	27 kDa	TRUE	.013989976	7.724019364	2.03	0	0	4.13	5.0828	7.83
1645	TRUE	Empty	Transmembrane protein 14C OS=Homo sapiens GN=TMEM14C PE=1 SV=1	TM14C_HUMAN	12 kDa		0.000720974	12.60000401	0.99746	0	0	3.61	5.0828	3.91
1646	TRUE	Empty	Transmembrane protein 205 OS=Homo sapiens GN=TMEM205 PE=1 SV=1	TM205_HUMAN	21 kDa		0.103134221	3.9	2.03	0	0	1.04	3.85	3.91
1647	TRUE	Empty	Transmembrane protein 263 OS=Homo sapiens GN=TMEM263 PE=1 SV=1	TM263_HUMAN	12 kDa		0.215724507	0.1	0	4.1727	1.49	0.74522	0	0
1648	TRUE	Empty	Transmembrane protein 33 OS=Homo sapiens GN=TMEM33 PE=1 SV=2	TMM33_HUMAN	28 kDa		0.009521936	4.227868787	0	0.99746	0	1.04	0.84713	1.96
1649	TRUE	Empty	Transportin-1 OS=Homo sapiens GN=TNPO1 PE=1 SV=2	TNPO1_HUMAN	?	TRUE	0.15131275	INF	0	0	0	1.04	0.84713	5.87
1650	TRUE	Empty	Transportin-2 OS=Homo sapiens GN=TNPO2 PE=1 SV=3	TNPO2_HUMAN	?	TRUE	.152473369	INF	0	0	0	2.09	0	5.87
1651	TRUE	Empty	Transportin-3 OS=Homo sapiens GN=TNPO3 PE=1 SV=3	TNPO3_HUMAN	?		0.008380466	6.019389249	0	0.99746	0	1.04	1.43	2.94
1652	TRUE	Empty	Treacle protein OS=Homo sapiens GN=TCOF1 PE=1 SV=3	TCOF_HUMAN	?	TRUE	.474214831	0.5	0	5.59	3.98	0	0.84713	3.91
1653	TRUE	Empty	Tricarboxylate transport protein, mitochondrial OS=Homo sapiens GN=SLC25A1 PE=1 SV=2	TXTP_HUMAN	34 kDa	TRUE	0000034285	20.11368877	0	0.99746	0	6.707	6.71	6.85
1654	TRUE	Empty	Trifunctional enzyme subunit alpha, mitochondrial OS=Homo sapiens GN=HADHA PE=1 SV=2	ECHA_HUMAN	?		0.096816221	3.2	17.683	0	0	18.63	21.178	16.916
1655	TRUE	Empty	Trifunctional enzyme subunit beta, mitochondrial OS=Homo sapiens GN=HADHB PE=1 SV=3	ECHB_HUMAN	?		0.122067973	2.8	6.631	1.0432	1.49	11.178	4.57	11.277
1656	TRUE	Empty	Trifunctional purine biosynthetic protein adenosine-3 OS=Homo sapiens GN=GART PE=1 SV=1	PUR2_HUMAN	?	TRUE	.000558724	23.78942514	0	0.99746	0	6.707	7.42	9.78
1657	TRUE	Empty	Triokinase/FMN cyclase OS=Homo sapiens GN=TKFC PE=1 SV=2	TKFC_HUMAN	?		0.006836668	17.97445512	0	0.99746	0	3.61	7.42	6.85
1658	TRUE	Empty	Triosephosphate isomerase OS=Homo sapiens GN=TPI1 PE=1 SV=3	TPIS_HUMAN	?		0.210925012	0.6	112.73	306.69	197.5	105.08	122.83	135.33
1659	TRUE	Empty	Tripartite motif-containing protein 16 OS=Homo sapiens GN=TRIM16 PE=1 SV=3	TRI16_HUMAN	?	TRUE	.000114422	7.615844244	0	0.99746	0	2.57	2.14	2.94
1660	TRUE	Empty	tRNA (cytosine(34)-C(5))-methyltransferase OS=Homo sapiens GN=NSUN2 PE=1 SV=2	NSUN2_HUMAN	?		0.532669165	1.7	2.03	1.0432	1.49	0.74522	1.43	6.85
1661	TRUE	Empty	tRNA pseudouridine synthase A, mitochondrial OS=Homo sapiens GN=PUS1 PE=1 SV=3	TRUA_HUMAN	?		0.136121777	INF	0	0	0	1.04	0	0.93978
1662	TRUE	Empty	tRNA-splicing ligase RtcB homolog OS=Homo sapiens GN=RTCB PE=1 SV=1	RTCB_HUMAN	55 kDa		0.524532376	0.7	2.03	13.561	17.954	4.13	8.13	10.338
1663	TRUE	Empty	Trophoblast glycoprotein OS=Homo sapiens GN=TPBG PE=1 SV=1	TPBG_HUMAN	46 kDa	TRUE	.311098858	3	2.03	5.59	0	5.65	15.248	1.96
1664	TRUE	Empty	Tropomodulin-3 OS=Homo sapiens GN=TMOD3 PE=1 SV=1	TMOD3_HUMAN	40 kDa	TRUE	.890400991	1.1	0	3.1295	7.97	1.04	3.85	7.83
1665	TRUE	Empty	Tropomyosin alpha-1 chain OS=Homo sapiens GN=TPM1 PE=1 SV=2	TPM1_HUMAN	?	TRUE	.313354953	0.6	13.262	14.604	39.898	11.923	12.707	13.157
1666	TRUE	Empty	Tropomyosin alpha-3 chain OS=Homo sapiens GN=TPM3 PE=1 SV=2	TPM3_HUMAN	?	TRUE	.486832596	0.6	15.472	10.432	51.868	14.904	17.79	15.037
1667	TRUE	Empty	Tropomyosin alpha-4 chain OS=Homo sapiens GN=TPM4 PE=1 SV=3	TPM4_HUMAN	?	TRUE	0.20339762	0.4	41.996	32.338	107.73	27.573	26.261	19.735
1668	TRUE	Empty	Tropomyosin beta chain OS=Homo sapiens GN=TPM2 PE=1 SV=1	TPM2_HUMAN	?	TRUE	.206851653	0.4	26.524	25.036	79.797	18.63	18.637	12.217
1669	TRUE	Empty	Tryptophan--tRNA ligase, cytoplasmic OS=Homo sapiens GN=WARS PE=1 SV=2	SYWC_HUMAN	?		0.427578962	1.7	0	14.604	2.24	11.923	9.85	8.81
1670	TRUE	Empty	Tubulin alpha-1A chain OS=Homo sapiens GN=TUBA1A PE=1 SV=1	TBA1A_HUMAN	?	TRUE	.722320238	1.2	243.14	56.331	62.84	128.92	177.05	128.75
1671	TRUE	Empty	Tubulin alpha-1B chain OS=Homo sapiens GN=TUBA1B PE=1 SV=1	TBA1B_HUMAN	?	TRUE	.997543402	1	495.11	67.806	69.822	176.62	284.64	172.92
1672	TRUE	Empty	Tubulin beta chain OS=Homo sapiens GN=TUBB PE=1 SV=2	TBB5_HUMAN	50 kDa	TRUE	.930302619	1	320.5	167.95	179.54	251.14	218.56	212.39

1673	TRUE	Empty	Tubulin beta-2B chain OS=Homo sapiens GN=TUBB2B PE=1 SV=1	TBB2B_HUMAN	50 kDa	TRUE	.966707905	1	249.77	129.35	126.68	210.9	160.96	140.03
1674	TRUE	Empty	Tubulin beta-3 chain OS=Homo sapiens GN=TUBB3 PE=1 SV=2	TBB3_HUMAN	?	TRUE	.913679859	1	134.83	119.96	108.72	107.31	129.61	123.11
1675	TRUE	Empty	Tubulin beta-4B chain OS=Homo sapiens GN=TUBB4B PE=1 SV=1	TBB4B_HUMAN	50 kDa	TRUE	.975531506	1	305.02	172.12	183.53	242.94	216.02	197.35
1676	TRUE	Empty	Tubulin beta-6 chain OS=Homo sapiens GN=TUBB6 PE=1 SV=1	TBB6_HUMAN	50 kDa	TRUE	.680724659	0.9	128.2	74.065	86.779	70.051	105.89	87.4
1677	TRUE	Empty	Tubulin-folding cofactor B OS=Homo sapiens GN=TBCB PE=1 SV=2	TBCB_HUMAN	?		0.864004896	1.2	0	0	1.49	1.04	0	0.93978
1678	TRUE	Empty	Tubulin-specific chaperone A OS=Homo sapiens GN=TBCA PE=1 SV=3	TBCA_HUMAN	?		0.450034554	0.6	4.06	8.54	18.952	8.26	5.99	5.87
1679	TRUE	Empty	Tubulin-specific chaperone D OS=Homo sapiens GN=TBCD PE=1 SV=2	TBCD_HUMAN	?		0.011102006	8.685447892	0	0	1.49	7.22	4.57	5.87
1680	TRUE	Empty	Tubulin-specific chaperone E OS=Homo sapiens GN=TBCE PE=1 SV=1	TBCE_HUMAN	?		0.384988624	3.7	0	1.0432	0	2.09	0.84713	0
1681	TRUE	Empty	Tubulin--tyrosine ligase-like protein 12 OS=Homo sapiens GN=TTLL12 PE=1 SV=2	TTL12_HUMAN	74 kDa	TRUE	.119086248	INF	0	0	0	2.57	0	1.96
1682	TRUE	Empty	Tumor protein D52 OS=Homo sapiens GN=TPD52 PE=1 SV=2	TPD52_HUMAN	?	TRUE	.503208195	0.6	0	20.863	11.969	5.65	7.42	6.85
1683	TRUE	Empty	Tumor protein D53 OS=Homo sapiens GN=TPD52L1 PE=1 SV=1	TPD53_HUMAN	?	TRUE	.453013153	0.5	0	14.604	6.22	3.61	2.14	4.89
1684	TRUE	Empty	Tumor protein D54 OS=Homo sapiens GN=TPD52L2 PE=1 SV=2	TPD54_HUMAN	?		0.9484979	1	13.262	35.468	19.949	17.885	28.803	23.495
1685	TRUE	Empty	Tumor-associated calcium signal transducer 2 OS=Homo sapiens GN=TACSTD2 PE=1 SV=3	TACD2_HUMAN	36 kDa	TRUE	.009521936	4.227868787	0.99746	0	0	1.04	0.84713	1.96
1686	TRUE	Empty	Twinfilin-1 OS=Homo sapiens GN=TWF1 PE=1 SV=3	TWF1_HUMAN	?	TRUE	.017661144	6.775710304	0	0.99746	0	1.04	3.85	1.96
1687	TRUE	Empty	Twinfilin-2 OS=Homo sapiens GN=TWF2 PE=1 SV=2	TWF2_HUMAN	40 kDa	TRUE	.943595292	0.9	0	1.0432	0.99746	0	0	1.96
1688	TRUE	Empty	Type 1 phosphatidylinositol 4,5-bisphosphate 4-phosphatase OS=Homo sapiens GN=TMEM55B PE=1 SV=1	TM55B_HUMAN	?		0.080922552	INF	0	0	0	0.74522	1.43	3.91
1689	TRUE	Empty	Type II inositol 3,4-bisphosphate 4-phosphatase OS=Homo sapiens GN=INPP4B PE=2 SV=4	INP4B_HUMAN	?	TRUE	)000160132	16.76565425	0	1.0432	0.99746	11.923	11.013	11.277
1690	TRUE	Empty	Tyrosine-protein phosphatase non-receptor type 1 OS=Homo sapiens GN=PTPN1 PE=1 SV=1	PTN1_HUMAN	50 kDa		0.143783106	3.9	2.03	0	0	1.04	2.14	4.89
1691	TRUE	Empty	Tyrosine-protein phosphatase non-receptor type 11 OS=Homo sapiens GN=PTPN11 PE=1 SV=2	PTN11_HUMAN	?	TRUE	.231711402	INF	0	0	0	0.74522	0	2.94
1692	TRUE	Empty	Tyrosine--tRNA ligase, cytoplasmic OS=Homo sapiens GN=YARS PE=1 SV=4	SYYC_HUMAN	59 kDa	TRUE	0.16325979	1.7	2.03	4.1727	7.97	6.707	7.42	10.338
1693	TRUE	Empty	U1 small nuclear ribonucleoprotein 70 kDa OS=Homo sapiens GN=SNRNP70 PE=1 SV=2	RU17_HUMAN	?		0.556868603	0.6	4.06	4.1727	0.99746	1.04	0	4.89
1694	TRUE	Empty	U2 small nuclear ribonucleoprotein A' OS=Homo sapiens GN=SNRPA1 PE=1 SV=2	RU2A_HUMAN	28 kDa		0.791336004	1.2	0	9.86	8.71	3.61	10.166	7.83
1695	TRUE	Empty	U2 small nuclear ribonucleoprotein B'' OS=Homo sapiens GN=SNRPB2 PE=1 SV=1	RU2B_HUMAN	25 kDa	TRUE	.212051675	3.4	0	1.0432	0.99746	0.74522	4.57	1.96
1696	TRUE	Empty	U2 snRNP-associated SURP motif-containing protein OS=Homo sapiens GN=U2SURP PE=1 SV=2	SR140_HUMAN	?	TRUE	.000114504	5.077196078	0	0.99746	0	1.04	1.43	1.96
1697	TRUE	Empty	U4/U6 small nuclear ribonucleoprotein Prp31 OS=Homo sapiens GN=PRPF31 PE=1 SV=2	PRP31_HUMAN	?	TRUE	.105819574	INF	0	0	0	0.74522	2.14	5.87
1698	TRUE	Empty	U4/U6 small nuclear ribonucleoprotein Prp4 OS=Homo sapiens GN=PRPF4 PE=1 SV=2	PRP4_HUMAN	?	TRUE	.017276814	4.37728617	0	2.0863	0.99746	4.13	3.85	5.87
1699	TRUE	Empty	U4/U6.U5 tri-snRNP-associated protein 1 OS=Homo sapiens GN=SART1 PE=1 SV=1	SNUT1_HUMAN	90 kDa	TRUE	.963269819	1	0	5.59	4.73	4.13	1.43	3.91

1700	TRUE	Empty	U4/U6.U5 tri-snRNP-associated protein 2 OS=Homo sapiens GN=USP39 PE=1 SV=2	SNUT2_HUMAN	?		0.601935698	2.5	0	0	0.99746	0	2.14	0
1701	TRUE	Empty	U5 small nuclear ribonucleoprotein 200 kDa helicase OS=Homo sapiens GN=SNRNP200 PE=1 SV=2	U520_HUMAN	?	TRUE	.034946445	6.036216394	8.13	0	0	24.592	11.86	16.916
1702	TRUE	Empty	U5 small nuclear ribonucleoprotein 40 kDa protein OS=Homo sapiens GN=SNRNP40 PE=1 SV=1	SNR40_HUMAN	?		0.10952434	2.8	0	1.0432	0.99746	2.57	2.14	0.93978
1703	TRUE	Empty	U6 snRNA-associated Sm-like protein LSm2 OS=Homo sapiens GN=LSM2 PE=1 SV=1	LSM2_HUMAN	11 kDa		0.196672229	0.4	2.03	2.0863	0.99746	0	0	1.96
1704	TRUE	Empty	U6 snRNA-associated Sm-like protein LSm8 OS=Homo sapiens GN=LSM8 PE=1 SV=3	LSM8_HUMAN	10 kDa		0.229657678	0.2	4.06	0	2.24	1.04	0	0
1705	TRUE	Empty	Ubiquilin-1 OS=Homo sapiens GN=UBQLN1 PE=1 SV=2	UBQL1_HUMAN	?	TRUE	.175169842	0.5	8.13	30.252	21.944	11.178	11.013	7.83
1706	TRUE	Empty	Ubiquilin-4 OS=Homo sapiens GN=UBQLN4 PE=1 SV=2	UBQL4_HUMAN	?	TRUE	.218811624	0.5	4.06	11.475	13.964	8.74	2.14	4.89
1707	TRUE	Empty	Ubiquitin carboxyl-terminal hydrolase 10 OS=Homo sapiens GN=USP10 PE=1 SV=2	UBP10_HUMAN	?	TRUE	.000114504	5.077196078	0	0	0.99746	1.04	1.43	1.96
1708	TRUE	Empty	Ubiquitin carboxyl-terminal hydrolase 14 OS=Homo sapiens GN=USP14 PE=1 SV=3	UBP14_HUMAN	?	TRUE	.096081604	2.2	4.06	1.0432	2.24	5.17	4.57	8.81
1709	TRUE	Empty	Ubiquitin carboxyl-terminal hydrolase 15 OS=Homo sapiens GN=USP15 PE=1 SV=3	UBP15_HUMAN	?	TRUE	.137246924	3.1	0	1.0432	0	1.04	0.84713	0.93978
1710	TRUE	Empty	Ubiquitin carboxyl-terminal hydrolase 5 OS=Homo sapiens GN=USP5 PE=1 SV=2	UBP5_HUMAN	?	TRUE	.026849742	2.686749288	2.03	3.1295	1.49	4.13	6.71	8.81
1711	TRUE	Empty	Ubiquitin carboxyl-terminal hydrolase 7 OS=Homo sapiens GN=USP7 PE=1 SV=2	UBP7_HUMAN	?		0.030399489	15.53806669	0.99746	0	0	2.57	7.42	5.87
1712	TRUE	Empty	Ubiquitin carboxyl-terminal hydrolase isozyme L3 OS=Homo sapiens GN=UCHL3 PE=1 SV=1	UCHL3_HUMAN	26 kDa		0.898254512	1.1	0	1.0432	1.49	1.04	0	1.96
1713	TRUE	Empty	Ubiquitin conjugation factor E4 A OS=Homo sapiens GN=UBE4A PE=1 SV=2	UBE4A_HUMAN	?		0.031963678	6.664357468	0	0	0.99746	2.09	0.84713	2.94
1714	TRUE	Empty	Ubiquitin conjugation factor E4 B OS=Homo sapiens GN=UBE4B PE=1 SV=1	UBE4B_HUMAN	?		0.124952625	INF	0	0	0	0	2.14	1.96
1715	TRUE	Empty	Ubiquitin domain-containing protein UBFD1 OS=Homo sapiens GN=UBFD1 PE=1 SV=2	UBFD1_HUMAN	33 kDa		0.925926432	0.9	0	5.59	0.99746	2.57	2.14	0.93978
1716	TRUE	Empty	Ubiquitin fusion degradation protein 1 homolog OS=Homo sapiens GN=UFD1L PE=1 SV=3	UFD1_HUMAN	?		0.862139202	1.1	0	5.59	4.73	1.04	3.85	6.85
1717	TRUE	Empty	Ubiquitin thioesterase OTUB1 OS=Homo sapiens GN=OTUB1 PE=1 SV=2	OTUB1_HUMAN	?		0.059167149	2.2	6.631	1.0432	4.73	10.433	9.85	7.83
1718	TRUE	Empty	Ubiquitin-40S ribosomal protein S27a OS=Homo sapiens GN=RPS27A PE=1 SV=2	RS27A_HUMAN	18 kDa	TRUE	.527101643	0.6	50.837	13.561	23.939	42.477	5.99	5.87
1719	TRUE	Empty	Ubiquitin-associated protein 2 OS=Homo sapiens GN=UBAP2 PE=1 SV=1	UBAP2_HUMAN	?	TRUE	0.15359	0	0	1.0432	1.49	0	0	0
1720	TRUE	Empty	Ubiquitin-associated protein 2-like OS=Homo sapiens GN=UBAP2L PE=1 SV=2	UBP2L_HUMAN	?	TRUE	.859924228	0.9	2.03	6.259	8.71	3.61	5.99	6.85
1721	TRUE	Empty	Ubiquitin-conjugating enzyme E2 A OS=Homo sapiens GN=UBE2A PE=1 SV=2	UBE2A_HUMAN	?	TRUE	0.63309427	1.5	0	1.0432	1.49	0.74522	0.84713	2.94
1722	TRUE	Empty	Ubiquitin-conjugating enzyme E2 D3 OS=Homo sapiens GN=UBE2D3 PE=1 SV=1	UB2D3_HUMAN	?		0.132510102	4.1	0	2.0863	0	2.57	1.43	4.89
1723	TRUE	Empty	Ubiquitin-conjugating enzyme E2 K OS=Homo sapiens GN=UBE2K PE=1 SV=3	UBE2K_HUMAN	?		0.098502269	2.7	2.03	5.59	0	7.22	4.57	8.81
1724	TRUE	Empty	Ubiquitin-conjugating enzyme E2 L3 OS=Homo sapiens GN=UBE2L3 PE=1 SV=1	UB2L3_HUMAN	?		0.036904884	0.632915255	11.052	15.648	11.969	7.22	7.42	9.78
1725	TRUE	Empty	Ubiquitin-conjugating enzyme E2 N OS=Homo sapiens GN=UBE2N PE=1 SV=1	UBE2N_HUMAN	17 kDa		0.062348734	0.6	11.052	9.86	10.972	4.13	9.85	5.87
1726	TRUE	Empty	Ubiquitin-conjugating enzyme E2 variant 1 OS=Homo sapiens GN=UBE2V1 PE=1 SV=2	UB2V1_HUMAN	?	TRUE	.754889106	1.1	4.06	10.432	12.967	8.74	6.71	16.916

1727	TRUE	Empty	Ubiquitin-fold modifier 1 OS=Homo sapiens GN=UFM1 PE=1 SV=1	UFM1_HUMAN	?		0.985338155	1	0	5.59	5.47	4.13	5.99	0.93978
1728	TRUE	Empty	Ubiquitin-like modifier-activating enzyme 1 OS=Homo sapiens GN=UBA1 PE=1 SV=3	UBA1_HUMAN	?	TRUE	.037461083	2.26131865	35.365	9.86	14.962	41.732	49.134	44.17
1729	TRUE	Empty	Ubiquitin-like protein ISG15 OS=Homo sapiens GN=ISG15 PE=1 SV=5	ISG15_HUMAN	18 kDa		0.857764027	0.8	35.365	3.1295	0	14.159	9.85	8.81
1730	TRUE	Empty	Ubiquitin-protein ligase E3A OS=Homo sapiens GN=UBE3A PE=1 SV=4	UBE3A_HUMAN	?	TRUE	.169759736	INF	0	0	0	0	0.84713	1.96
1731	TRUE	Empty	UBP8_HUMAN-DECOY	UBP8_HUMAN-DEC	?	TRUE	.373900966	INF	0	0	0	0	1.43	0
1732	TRUE	Empty	UBX domain-containing protein 7 OS=Homo sapiens GN=UBXN7 PE=1 SV=2	UBXN7_HUMAN	55 kDa		0.92881698	0.9	0	0	1.49	0	0.84713	0.93978
1733	TRUE	Empty	UDP-glucose 6-dehydrogenase OS=Homo sapiens GN=UGDH PE=1 SV=1	UGDH_HUMAN	?		0.241076102	0.8	132.62	154.39	102.74	114.02	115.21	90.219
1734	TRUE	Empty	UDP-glucose:glycoprotein glucosyltransferase 1 OS=Homo sapiens GN=UGGT1 PE=1 SV=3	UGGG1_HUMAN	?	TRUE	.136603418	3.1	8.13	0	0	12.669	7.42	7.83
1735	TRUE	Empty	UDP-N-acetylhexosamine pyrophosphorylase-like protein 1 OS=Homo sapiens GN=UAP1L1 PE=1 SV=2	UAP1L_HUMAN	?	TRUE	.094905451	INF	0	0	0	0.74522	0.84713	2.94
1736	TRUE	Empty	UMP-CMP kinase OS=Homo sapiens GN=CMPK1 PE=1 SV=3	KCY_HUMAN	?		0.572371231	0.7	2.03	16.691	6.22	2.57	7.42	7.83
1737	TRUE	Empty	Unconventional myosin-Ib OS=Homo sapiens GN=MYO1B PE=1 SV=3	MYO1B_HUMAN	?	TRUE	.005155173	3.720367683	6.631	3.1295	6.22	17.14	25.414	19.735
1738	TRUE	Empty	Unconventional myosin-Ic OS=Homo sapiens GN=MYO1C PE=1 SV=4	MYO1C_HUMAN	?	TRUE	.036752639	2.880033302	2.03	1.0432	2.24	7.22	6.71	3.91
1739	TRUE	Empty	Unconventional myosin-Id OS=Homo sapiens GN=MYO1D PE=1 SV=2	MYO1D_HUMAN	116 kDa	TRUE	.017663121	3.387905279	0	0	0.99746	0.74522	1.43	0.93978
1740	TRUE	Empty	Unconventional myosin-Vc OS=Homo sapiens GN=MYO5C PE=1 SV=2	MYO5C_HUMAN	?	TRUE	.118904525	6.2	0	1.0432	0	3.61	0.84713	1.96
1741	TRUE	Empty	Unconventional myosin-VI OS=Homo sapiens GN=MYO6 PE=1 SV=4	MYO6_HUMAN	?	TRUE	.081266401	4.8	2.03	0	0.99746	8.74	4.57	2.94
1742	TRUE	Empty	UPF0160 protein MYG1, mitochondrial OS=Homo sapiens GN=C12orf10 PE=1 SV=2	MYG1_HUMAN	42 kDa		0.583741844	0.6	0	12.518	4.73	2.09	4.57	3.91
1743	TRUE	Empty	UPF0568 protein C14orf166 OS=Homo sapiens GN=C14orf166 PE=1 SV=1	CN166_HUMAN	28 kDa	TRUE	.751050258	0.9	13.262	18.777	10.972	14.159	8.13	16.916
1744	TRUE	Empty	Up-regulated during skeletal muscle growth protein 5 OS=Homo sapiens GN=USMG5 PE=1 SV=1	USMG5_HUMAN	6 kDa		0.140218517	INF	0	0	0	0	1.43	2.94
1745	TRUE	Empty	Uridine 5'-monophosphate synthase OS=Homo sapiens GN=UMPS PE=1 SV=1	UMPS_HUMAN	?		0.017662632	22.17883424	0	0	0.99746	8.74	10.166	3.91
1746	TRUE	Empty	UTP--glucose-1-phosphate uridylyltransferase OS=Homo sapiens GN=UGP2 PE=1 SV=5	UGPA_HUMAN	?	TRUE	.022551025	11.66564417	0	1.0432	0	5.17	3.85	2.94
1747	TRUE	Empty	UV excision repair protein RAD23 homolog A OS=Homo sapiens GN=RAD23A PE=1 SV=1	RD23A_HUMAN	?	TRUE	.823046031	0.8	0	7.22	0.99746	2.57	2.14	1.96
1748	TRUE	Empty	UV excision repair protein RAD23 homolog B OS=Homo sapiens GN=RAD23B PE=1 SV=1	RD23B_HUMAN	?	TRUE	.194505642	0.3	22.103	91.799	30.921	14.904	14.401	13.157
1749	TRUE	Empty	Vacuolar protein sorting-associated protein 26A OS=Homo sapiens GN=VPS26A PE=1 SV=2	VP26A_HUMAN	?	TRUE	.303769855	1.8	0	4.1727	1.49	3.61	2.14	4.89
1750	TRUE	Empty	Vacuolar protein sorting-associated protein 29 OS=Homo sapiens GN=VPS29 PE=1 SV=1	VPS29_HUMAN	?		0.194576287	3.2	0	2.0863	0	2.09	0.84713	2.94
1751	TRUE	Empty	Vacuolar protein sorting-associated protein 35 OS=Homo sapiens GN=VPS35 PE=1 SV=2	VPS35_HUMAN	92 kDa	TRUE	.000234508	19.91859323	0.99746	0	0	7.22	6.71	5.87
1752	TRUE	Empty	Vacuolar protein sorting-associated protein 45 OS=Homo sapiens GN=VPS45 PE=1 SV=1	VPS45_HUMAN	?		0.022682884	24.374411	0	0.99746	0	4.13	7.42	12.217
1753	TRUE	Empty	Vacuolar protein sorting-associated protein 4A OS=Homo sapiens GN=VPS4A PE=1 SV=1	VPS4A_HUMAN	49 kDa	TRUE	0.82243974	0.7	0	0	3.98	0	0	2.94

1754	TRUE	Empty	Valine--tRNA ligase OS=Homo sapiens GN=VARS PE=1 SV=4	SYVC_HUMAN	?		0.009093586	4.729429522	4.06	0	4.73	18.63	12.707	13.157
1755	TRUE	Empty	Vasodilator-stimulated phosphoprotein OS=Homo sapiens GN=VASP PE=1 SV=3	VASP_HUMAN	40 kDa		0.971364092	1	2.03	8.54	2.24	9.78	2.14	0.93978
1756	TRUE	Empty	Very long-chain specific acyl-CoA dehydrogenase, mitochondrial OS=Homo sapiens GN=ACADVL PE=1 SV=1	ACADV_HUMAN	?		0.007855217	2.001247569	8.13	10.432	7.97	20.866	18.637	15.037
1757	TRUE	Empty	Very-long-chain (3R)-3-hydroxyacyl-CoA dehydratase 3 OS=Homo sapiens GN=HACD3 PE=1 SV=2	HACD3_HUMAN	?		0.140218517	INF	0	0	0	0	1.43	2.94
1758	TRUE	Empty	Very-long-chain enoyl-CoA reductase OS=Homo sapiens GN=TECR PE=1 SV=1	TECR_HUMAN	?		0.197570015	2.6	6.631	0	0	6.707	4.57	6.85
1759	TRUE	Empty	Vesicle-associated membrane protein 7 OS=Homo sapiens GN=VAMP7 PE=1 SV=3	VAMP7_HUMAN	?		0.094905451	INF	0	0	0	0.74522	0.84713	2.94
1760	TRUE	Empty	Vesicle-associated membrane protein 8 OS=Homo sapiens GN=VAMP8 PE=1 SV=1	VAMP8_HUMAN	11 kDa		0.281714709	INF	0	0	0	5.17	0	0.93978
1761	TRUE	Empty	Vesicle-associated membrane protein-associated protein B/C OS=Homo sapiens GN=VAPB PE=1 SV=3	VAPB_HUMAN	?	TRUE	.009163088	5.318766828	6.631	0	0.99746	15.65	12.707	12.217
1762	TRUE	Empty	Vesicle-fusing ATPase OS=Homo sapiens GN=NSF PE=1 SV=3	NSF_HUMAN	?	TRUE	.003617465	23.78050248	0	0.99746	0	7.22	5.99	10.338
1763	TRUE	Empty	Vesicle-trafficking protein SEC22b OS=Homo sapiens GN=SEC22B PE=1 SV=4	SC22B_HUMAN	25 kDa		0.665592284	1.5	4.06	0	0	2.09	1.43	1.96
1764	TRUE	Empty	Vesicular integral-membrane protein VIP36 OS=Homo sapiens GN=LMAN2 PE=1 SV=1	LMAN2_HUMAN	40 kDa		0.080775509	INF	0	0	0	5.65	2.14	0.93978
1765	TRUE	Empty	Vigilin OS=Homo sapiens GN=HDLBP PE=1 SV=2	VIGLN_HUMAN	?		0.332263297	1.8	2.03	0	5.47	6.707	3.85	4.89
1766	TRUE	Empty	Vimentin OS=Homo sapiens GN=VIM PE=1 SV=4	VIME_HUMAN	54 kDa	TRUE	.785762157	0.8	2.03	0	4.73	1.04	2.14	1.96
1767	TRUE	Empty	Vinculin OS=Homo sapiens GN=VCL PE=1 SV=4	VINC_HUMAN	?		0.890700939	1.1	0	23.993	30.921	16.395	22.025	20.675
1768	TRUE	Empty	Voltage-dependent anion-selective channel protein 1 OS=Homo sapiens GN=VDAC1 PE=1 SV=2	VDAC1_HUMAN	31 kDa	TRUE	.589113084	1.4	4.06	26.079	5.47	13.414	15.248	20.675
1769	TRUE	Empty	Voltage-dependent anion-selective channel protein 2 OS=Homo sapiens GN=VDAC2 PE=1 SV=2	VDAC2_HUMAN	?	TRUE	.925278289	1	6.631	4.1727	1.49	5.65	3.85	3.91
1770	TRUE	Empty	Voltage-dependent anion-selective channel protein 3 OS=Homo sapiens GN=VDAC3 PE=1 SV=1	VDAC3_HUMAN	?	TRUE	.017349763	4.427541894	0	4.1727	0.99746	5.17	8.13	8.81
1771	TRUE	Empty	von Willebrand factor A domain-containing protein 9 OS=Homo sapiens GN=VWA9 PE=1 SV=2	VWA9_HUMAN	?		0.000114504	5.077196078	0	0.99746	0	1.04	1.43	1.96
1772	TRUE	Empty	V-type proton ATPase catalytic subunit A OS=Homo sapiens GN=ATP6V1A PE=1 SV=2	VATA_HUMAN	?	TRUE	.011499868	5.27858634	0	1.0432	0.99746	4.13	2.14	3.91
1773	TRUE	Empty	V-type proton ATPase subunit B, brain isoform OS=Homo sapiens GN=ATP6V1B2 PE=1 SV=3	VATB2_HUMAN	57 kDa	TRUE	.052200604	INF	0	0	0	5.17	1.43	2.94
1774	TRUE	Empty	V-type proton ATPase subunit d 1 OS=Homo sapiens GN=ATP6V0D1 PE=1 SV=1	VA0D1_HUMAN	40 kDa		0.005538615	3.285655565	0.99746	0	0	1.04	0.84713	0.93978
1775	TRUE	Empty	V-type proton ATPase subunit G 1 OS=Homo sapiens GN=ATP6V1G1 PE=1 SV=3	VATG1_HUMAN	14 kDa		0.990696861	1	0	6.259	2.24	3.61	1.43	3.91
1776	TRUE	Empty	V-type proton ATPase subunit H OS=Homo sapiens GN=ATP6V1H PE=1 SV=1	VATH_HUMAN	?	TRUE	0.00025994	5.824393961	0.99746	0	0	2.57	1.43	1.96
1777	TRUE	Empty	WASH complex subunit FAM21C OS=Homo sapiens GN=FAM21C PE=1 SV=3	FA21C_HUMAN	?	TRUE	.534861273	0.5	0	1.0432	1.49	1.04	0	0
1778	TRUE	Empty	WASH complex subunit strumpellin OS=Homo sapiens GN=KIAA0196 PE=1 SV=1	STRUM_HUMAN	134 kDa		0.06853503	INF	0	0	0	0.74522	2.14	0.93978
1779	TRUE	Empty	WD repeat-containing protein 1 OS=Homo sapiens GN=WDR1 PE=1 SV=4	WDR1_HUMAN	?		0.025625731	2.985886677	0	4.1727	3.98	8.74	6.71	9.78
1780	TRUE	Empty	WD repeat-containing protein 18 OS=Homo sapiens GN=WDR18 PE=1 SV=2	WDR18_HUMAN	47 kDa		0.250282168	3.1	0	1.0432	1.49	5.17	2.14	0.93978
1781	TRUE	Empty	WD repeat-containing protein 26 OS=Homo sapiens GN=WDR26 PE=1 SV=3	WDR26_HUMAN	?		0.147672906	INF	0	0	0	0	1.43	0.93978

1782	TRUE	Empty	WD repeat-containing protein 5 OS=Homo sapiens GN=WDR5 PE=1 SV=1	WDR5_HUMAN	37 kDa	TRUE	0.52907428	0.4	0	5.59	0.99746	0	0.84713	1.96
1783	TRUE	Empty	WD40 repeat-containing protein SMU1 OS=Homo sapiens GN=SMU1 PE=1 SV=2	SMU1_HUMAN	?		0.131893048	INF	0	0	0	0	4.57	2.94
1784	TRUE	Empty	WW domain-binding protein 11 OS=Homo sapiens GN=WBP11 PE=1 SV=1	WBP11_HUMAN	70 kDa	TRUE	.693507807	1.6	0	0	1.49	2.57	0	0.93978
1785	TRUE	Empty	WW domain-binding protein 2 OS=Homo sapiens GN=WBP2 PE=1 SV=1	WBP2_HUMAN	28 kDa		0.2341866	0.2	0	4.1727	1.49	0	0	0.93978
1786	TRUE	Empty	Xaa-Pro aminopeptidase 1 OS=Homo sapiens GN=XPNPEP1 PE=1 SV=3	XPP1_HUMAN	?		0.098928091	3.4	0	0	1.49	2.57	1.43	2.94
1787	TRUE	Empty	Xaa-Pro dipeptidase OS=Homo sapiens GN=PEPD PE=1 SV=3	PEPD_HUMAN	?		0.875156665	1.1	0	2.0863	2.24	2.09	1.43	0.93978
1788	TRUE	Empty	X-ray repair cross-complementing protein 5 OS=Homo sapiens GN=XRCC5 PE=1 SV=3	XRCC5_HUMAN	83 kDa		0.112146708	3.2	6.631	1.0432	2.24	17.885	5.99	10.338
1789	TRUE	Empty	X-ray repair cross-complementing protein 6 OS=Homo sapiens GN=XRCC6 PE=1 SV=2	XRCC6_HUMAN	?		0.074706697	2.5	15.472	2.0863	3.98	17.885	22.025	15.037
1790	TRUE	Empty	Y-box-binding protein 3 OS=Homo sapiens GN=YBX3 PE=1 SV=4	YBOX3_HUMAN	?	TRUE	.803807751	1.1	13.262	9.86	18.952	20.866	12.707	11.277
1791	TRUE	Empty	YLP motif-containing protein 1 OS=Homo sapiens GN=YLPM1 PE=1 SV=3	YLPM1_HUMAN	?	TRUE	.373900966	0	0	0	1.49	0	0	0
1792	TRUE	Empty	YTH domain-containing family protein 1 OS=Homo sapiens GN=YTHDF1 PE=1 SV=1	YTHD1_HUMAN	?	TRUE	.209754271	3.9	0	2.0863	0.99746	7.22	0.84713	3.91
1793	TRUE	Empty	YTH domain-containing family protein 2 OS=Homo sapiens GN=YTHDF2 PE=1 SV=2	YTHD2_HUMAN	?	TRUE	.534861273	0.5	0	1.0432	1.49	1.04	0	0
1794	TRUE	Empty	YTH domain-containing family protein 3 OS=Homo sapiens GN=YTHDF3 PE=1 SV=1	YTHD3_HUMAN	64 kDa	TRUE	.264790325	7.4	0	1.0432	0	5.17	0.84713	0.93978
1795	TRUE	Empty	ZC3H1_HUMAN-DECOY	ZC3H1_HUMAN-DE(	?	TRUE	.373900966	INF	0	0	0	0	0	1.96
1796	TRUE	Empty	Zinc finger CCCH domain-containing protein 14 OS=Homo sapiens GN=ZC3H14 PE=1 SV=1	ZC3HE_HUMAN	?		0.169759736	INF	0	0	0	0	0.84713	1.96
1797	TRUE	Empty	Zinc finger CCCH domain-containing protein 15 OS=Homo sapiens GN=ZC3H15 PE=1 SV=1	ZC3HF_HUMAN	?	TRUE	.110753917	3.1	0	0	2.24	2.09	3.85	2.94
1798	TRUE	Empty	Zinc finger CCCH domain-containing protein 4 OS=Homo sapiens GN=ZC3H4 PE=1 SV=3	ZC3H4_HUMAN	140 kDa	TRUE	.588834218	0.4	0	0	3.98	1.04	0	0
1799	TRUE	Empty	Zinc finger protein 106 OS=Homo sapiens GN=ZNF106 PE=1 SV=1	ZN106_HUMAN	?	TRUE	.253272622	2.5	0	2.0863	0.99746	0.74522	4.57	2.94
1800	TRUE	Empty	Zinc finger protein 185 OS=Homo sapiens GN=ZNF185 PE=1 SV=3	ZN185_HUMAN	?		0.174882284	0.4	4.06	11.475	17.954	6.707	3.85	3.91
1801	TRUE	Empty	Zinc finger protein 428 OS=Homo sapiens GN=ZNF428 PE=1 SV=2	ZN428_HUMAN	20 kDa		0.555391835	0.6	0	2.0863	0.99746	0	0.84713	0.93978
1802	TRUE	Empty	Zinc phosphodiesterase ELAC protein 2 OS=Homo sapiens GN=ELAC2 PE=1 SV=2	RNZ2_HUMAN	?		0.935347937	1.1	2.03	0	0	0.74522	1.43	0
1803	TRUE	Empty	Zyxin OS=Homo sapiens GN=ZYX PE=1 SV=1	ZYX_HUMAN	?		0.198161774	0.2	2.03	21.907	7.97	1.04	1.43	1.96

END OF FILE
